# A synthetic circuit for buffering gene dosage variation between individual mammalian cells

**DOI:** 10.1038/s41467-021-23889-0

**Published:** 2021-07-05

**Authors:** Jin Yang, Jihwan Lee, Michelle A. Land, Shujuan Lai, Oleg A. Igoshin, François St-Pierre

**Affiliations:** 1 Department of Bioengineering, Rice University, Houston, TX USA; 2 Systems, Synthetic, and Physical Biology Program, Rice University, Houston, TX USA; 3 Department of Neuroscience, Baylor College of Medicine, Houston, TX USA; 4 Department of Biosciences, Rice University, Houston, TX USA; 5 Department of Chemistry, Rice University, Houston, TX USA; 6 Department of Electrical and Computer Engineering, Rice University, Houston, TX USA; 7 Department of Biochemistry and Molecular Biology, Baylor College of Medicine, Houston, TX USA; 8 Present Address: Department of Biological Engineering, Massachusetts Institute of Technology, Cambridge, MA USA

**Keywords:** Genetic vectors, Synthetic biology

## Abstract

Precise control of gene expression is critical for biological research and biotechnology. However, transient plasmid transfections in mammalian cells produce a wide distribution of copy numbers per cell, and consequently, high expression heterogeneity. Here, we report plasmid-based synthetic circuits – Equalizers – that buffer copy-number variation at the single-cell level. Equalizers couple a transcriptional negative feedback loop with post-transcriptional incoherent feedforward control. Computational modeling suggests that the combination of these two topologies enables Equalizers to operate over a wide range of plasmid copy numbers. We demonstrate experimentally that Equalizers outperform other gene dosage compensation topologies and produce as low cell-to-cell variation as chromosomally integrated genes. We also show that episome-encoded Equalizers enable the rapid generation of extrachromosomal cell lines with stable and uniform expression. Overall, Equalizers are simple and versatile devices for homogeneous gene expression and can facilitate the engineering of synthetic circuits that function reliably in every cell.

## Abstract (web summary)

Transient plasmid transfection produces a wide distribution of copy numbers and expression heterogeneity. Here the authors present synthetic circuits called Equalizers that buffer copy number variation at the single-cell level and minimize cell-to-cell expression variability.

## Introduction

Expressing genes of interest from synthetic cassettes are critical for studying natural proteins, producing reagents of commercial interest, and constructing synthetic biological circuits. Uniform expression among individual cells is needed when expressing genes whose properties depend on their concentration^1^. For example, many natural and engineered proteins can be nonfunctional or undetectable at low concentrations, and aggregate, mislocalize, or display aberrant function at high-expression levels^2–7^. Expression homogeneity would also facilitate the development of synthetic biological circuits with predictable behavior at the single-cell level^8,9^.

An important challenge to achieving uniform expression levels is the large variability in copy numbers observed after transfection of plasmids in mammalian cells^10^. While expression from the chromosome is widely used to reduce cell-to-cell variation in gene expression, it has multiple disadvantages compared with expression from plasmids. First, because experiments using plasmids can be conducted as soon as 1–3 days after transfection, the functions of new genes or circuits can be rapidly evaluated. In contrast, the creation of new cell lines via chromosomal integration typically takes several weeks because of the need to select stable integrants with the desired expression level. Second, plasmids can be more easily and rapidly deployed across a wide array of cell types. Meanwhile, chromosomal expression requires genomic integration to be repeated and validated for each cell type.

**Fig. 1:**
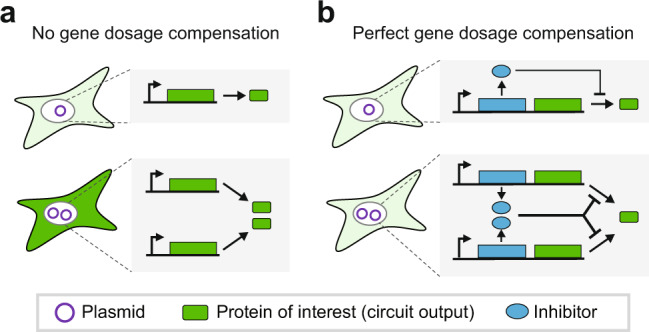
Gene expression from ideal dosage compensation circuits does not vary with plasmid copy number. **a** Expression from promoters with no control circuitry (i.e. open-loop circuits) is proportional to the plasmid copy number. **b** An ideal dosage compensation system uses control mechanisms to tune the per-copy expression rate, thereby maintaining constant expression regardless of copy number.

The limitations of classical expression methods have motivated the development of plasmid-based gene dosage compensation circuits—synthetic circuits that buffer plasmid copy-number variation. In an ideal compensation circuit, the per-plasmid expression rate is inversely proportional to the copy number; the total protein expression thus remains constant (Fig. 1). These circuits promise to combine the versatility and convenience of plasmids with the lower cell-to-cell variability of chromosomal expression. A variety of gene dosage compensation circuits have been theorized or tested in bacterial^11^ and mammalian cells^12–16^. However, existing mammalian circuits buffer gene dosage variation across a limited range of plasmid copy numbers. Moreover, their ability to reduce cell-to-cell expression variability within a transfected population has not been demonstrated or has been incompletely quantified.

Here, we report a series of engineered circuits—named Equalizers—that robustly buffer expression heterogeneity due to plasmid dosage variation between individual mammalian cells. We also describe computational models that guided circuit design and propose mechanistic explanations for their improved performance. We experimentally demonstrate that Equalizers function in multiple cell types and outperform other compensation circuits at the single-cell level. Finally, we show that when incorporated into replicating plasmids, Equalizers enable long-term gene expression with cell-to-cell variation comparable to chromosomal expression.

## Results

### Modeling suggests that negative feedback (NF) and incoherent feedforward (IFF) circuits can function synergistically for dosage compensation

**Fig. 2:**
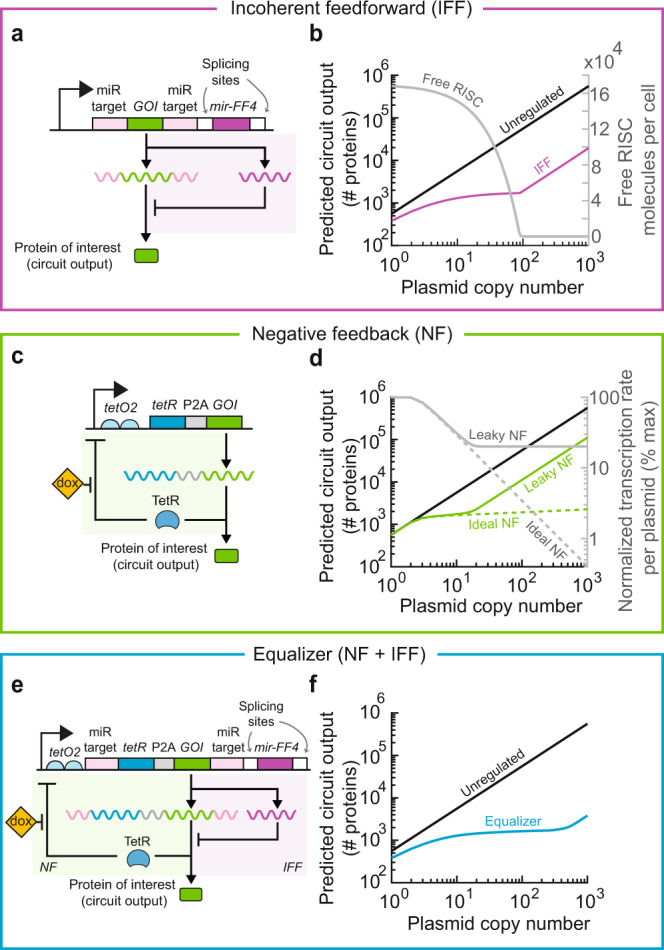
Combining incoherent feedforward (IFF) and negative feedback (NF) loops is predicted to widen the copy number range with efficient dosage compensation. **a** Schematic of the miRNA-based post-transcriptional IFF circuit. **b** The depletion of free RISC (gray trace) at high plasmid copy numbers abolishes dosage compensation by the IFF circuit (purple trace). **c** Schematic of the transcriptional NF circuit. **d** Incomplete repression by TetR results in a higher baseline transcription rate per plasmid (solid gray trace) that narrows the compensation range (leaky vs. ideal NF). Simulations were run with a doxycycline concentration that resulted in a wide compensation range of the NF circuit (1 ng/mL for both the ideal and leaky NF loops; see Supplementary Fig. 2). **e** Schematic of the Equalizer circuit, which combines transcriptional NF (green shaded area) with post-transcriptional IFF (purple shaded area). **f** Equalizer has the potential for compensating for a wider range of plasmid copy numbers than NF and IFF circuits alone. Simulated doxycycline concentration, 1 ng/mL.

To guide the development of a more effective gene dosage compensation system, we modeled different control topologies and quantified how their circuit output varied as a function of plasmid copy number. An important performance metric we considered was the range of plasmid copy numbers with effective gene dosage compensation, herein termed the compensation range. We first evaluated type I incoherent feedforward (IFF) circuits (Supplementary Fig. 1a) because they have been shown to buffer gene dosage variation in both natural^17–20^ and synthetic circuits^12,14–16^. Inspired by previous studies, we focused on IFF circuits where inhibition is mediated by microRNA (miRNA)-based RNA interference^12,14,15^. We modeled a representative implementation of a circuit containing miR-FF4 (a synthetic miRNA with a strong affinity to its target sites^21–23^), miR-FF4 target sites, and the gene of interest (*GOI*) on the same transcript (Fig. 2a). Following transcription, miRNAs are spliced out of a newly made precursor messenger RNA and incorporated into RNA-induced silencing complexes (RISC). The miRNA acts as a template for RISC to recognize and cleave mRNA molecules with miRNA-target sites^24^.

In agreement with previous studies, our deterministic simulations predicted that this IFF topology could compensate for gene dosage (Fig. 2b, purple vs. black curves; see Supplementary Note 1 for model description). However, the model also predicted that RISC availability decreases sharply at high plasmid concentrations (Fig. 2b, gray curve), consistent with previous reports of RISC saturation in the presence of high miRNA levels^25^. The absence of free RISC renders the IFF circuit inoperative, thereby limiting gene dosage compensation at high plasmid copy-numbers (Fig. 2b, purple curve at copy numbers ≳10^2^).

To identify a topology that would compensate gene dosage across a broader range of plasmid copy numbers than miRNA-based IFF circuits, we next considered that negative feedback (NF, Supplementary Fig. 1b) was also predicted to enable gene dosage compensation^12^. In one such NF circuit, the tetracycline repressor protein (TetR) is co-expressed from the same promoter as the *GOI* using a 2A ribosome-skipping sequence from porcine teschovirus-1 (Fig. 2c, refs. ^26,27^). By binding onto its cognate operator sites (*tetO2*) on the promoter, TetR represses both its own transcription and that of the *GOI*^28–30^.

Our model predicted that the NF circuit could effectively buffer gene dosage variation (Fig. 2d, ideal NF curves; Supplementary Note 2). Simulations further suggested that varying inducer (doxycycline) concentration regulates the dependence of dosage compensation on plasmid copy number (Supplementary Fig. 2a). For example, increasing doxycycline from 1 to 5 ng/mL improved dosage compensation at copy numbers ≳30 at the expense of reduced performance at lower plasmid concentrations (Supplementary Fig. 2a). Overall, several inducer concentrations produced effective dosage compensation over a wide range of plasmid copy numbers. However, this circuit has been reported to deviate from ideal behavior due to incomplete repression at high TetR concentration when no inducer is present, leading to “leaky” expression^29,31–33^ (Supplementary Fig. 3). Simulations predicted that this incomplete repression narrows the compensation range (Fig. 2d, leaky NF curves; Supplementary Fig. 2b).

We hypothesized that combining miRNA-based IFF and leaky TetR-based NF would widen the compensation range and simulated this combined architecture at different doxycycline concentrations (see Supplementary Note 1 for model description). The model predicted that this combination circuit could provide dosage compensation over 2–3 logs of plasmid copy numbers, outperforming both standalone IFF and NF topologies (Fig. 2e, f and Supplementary Fig. 4a). Simulations further suggested that this combination circuit would provide improved dosage compensation compared with the IFF subcircuit over a wide range of RISC concentrations (Supplementary Fig. 4b, c) and binding affinity between miRNAs and their targets (Supplementary Fig. 4d, e). We named this promising circuit “Equalizer” given its intended function to reduce gene expression variability between individual cells.

### Equalizer-L achieves as low cell-to-cell expression variability as stable cell lines

We next conducted a series of experiments to demonstrate that the Equalizer topology can effectively compensate for variability in gene expression caused by differences in plasmid copy number among transfected cells. Modeling results predicted that tuning the binding affinity of the miRNA to its target sites could change the Equalizer’s compensation performance (Supplementary Fig. 4e). Therefore, we constructed two Equalizer variants with different miRNA/target pairs: Equalizer-M uses miR-FF4 while Equalizer-H uses miR-FF3, a miRNA with lower affinity to its target than miR-FF4^22^ (Supplementary Fig. 5a, b). We also constructed Equalizer-L, which encodes miR-FF4 like Equalizer-M but incorporates a second miRNA-target site upstream of the start codon (Supplementary Figs. 5c and 2e), an arrangement that can increase miRNA-based inhibition^23^. The Equalizer variants and control plasmids were constructed with the enhanced green fluorescent protein (EGFP) as the circuit output reporter.

Cell-to-cell variability in plasmid copy number naturally arises during transient transfection as plasmid uptake is stochastic^34^. We identified cells that were successfully transfected with a spectrally compatible red fluorescent reporter (i.e., mCherry) expressed from a co-transfected plasmid (Supplementary Fig. 5k) or a separate cassette on the same plasmid as the circuit (e.g., Supplementary Fig. 5d). When present on the same plasmid, mCherry also served as a gene-dosage reporter: the mCherry fluorescence values were used to approximate the relative number of actively expressing plasmids.

We transfected HEK293 cells with Equalizer plasmids and measured single-cell fluorescence using flow cytometry. We used the coefficient of variation (CV) of EGFP fluorescence in mCherry^+^ cells to measure cell-to-cell variability in circuit output levels. Flow cytometry and microscopy produced similar CVs of circuit output, demonstrating that the CV is robust to differences in the method used to quantify single-cell fluorescence (Supplementary Fig. 6). We used flow cytometry in ensuing experiments, given the high throughput of this technique.

**Fig. 3:**
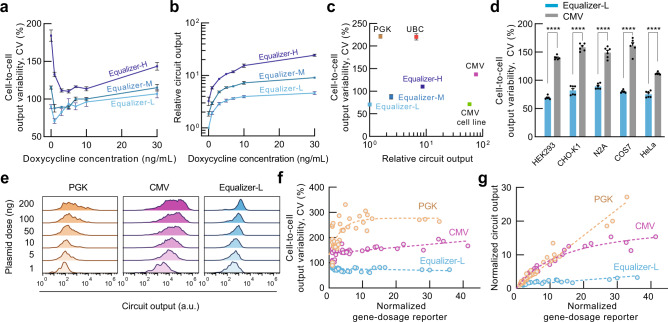
Equalizers demonstrate robust gene dosage compensation at the single-cell and population levels. Circuit output variability (**a**) and relative mean circuit output levels (**b**) of HEK293 cells transfected with the Equalizer plasmids and cultured under different inducer concentrations. Output levels are relative to that of uninduced Equalizer-L. Mean values ± SEM are shown. *n* = 8 (Equalizer-L) or 3 (-M/-H) independent transfections. Here and for **c**, **d**, 100 ng of circuit plasmids were used per transfection. **c** Equalizer plasmids produced lower cell-to-cell expression variability than plasmids with unregulated promoters. *p* < 0.01 for all pairs in Tukey’s multiple comparison tests. Cells transfected with Equalizer-L produced similar variability as cells with a chromosomally integrated unregulated CMV cassette (CMV cell line); *p* > 0.99, Tukey’s multiple comparison test. Circuit output values were relative to that of Equalizer-L. Mean ± SEM are shown; some error bars are too small to be seen. *n* = 3 (unregulated circuits and Equalizer-M & -H) or 8 (Equalizer-L) independent transfections. *n* = 3 independent cell cultures (CMV cell line). Equalizer-L was induced with 1 ng/mL of doxycycline. **d** Equalizer-L produced lower cell-to-cell variability than the CMV promoter in five cell lines. The black circles are independent transfections. Equalizer-L was induced with 1 ng/mL of doxycycline. Mean ± SEM are shown; some error bars are too small to be seen. *n* = 6 independent transfections per circuit. ****p < 0.0001; Sidak’s multiple comparison test. **e** Representative output-level histograms. Each histogram was normalized to its peak. For **e**–**g**, Equalizer-L was induced with 1 ng/mL of doxycycline. **f** Equalizer-L produced lower cell-to-cell variability than unregulated promoters at different gene dosage levels. The circles represent independent transfections. *n* = 36 per circuit (6 per dose and 6 doses per circuit). The dashed lines indicate trend lines (linear for Equalizer-L and CMV; exponential for PGK). The gene-dosage reporter values were normalized to those obtained when transfecting 1 ng of plasmid. **g** The mean Equalizer-L output is robust to increases in gene dosage. The gene-dosage and circuit output values were normalized to those obtained when transfecting 1 ng of plasmid. The circles and sample sizes are as in (**f**). The dashed lines indicate trend lines (linear for Equalizer-L and PGK, hyperbolic for CMV). Source data are provided as a Source Data file.

Our model predicted that doxycycline could be used to tune Equalizers’ gene dosage compensation range and profile (Supplementary Fig. 4a), as previously shown with NF circuits (Supplementary Fig. 2). To identify the optimal inducer concentration for each Equalizer variant, we quantified the cell-to-cell output variability at several doxycycline concentrations from 0 to 30 ng/mL (Fig. 3a). We observed that the shape of the CV dependence on inducer concentration was non-monotonic, with intermediate concentrations producing the lowest expression variability. At their respective inducer concentration, Equalizer-L produced the lowest cell-to-cell variation (CV = 71%), followed by Equalizer-M (CV = 88%) and Equalizer-H (CV = 110%). We also quantified relative expression levels over the same range of inducer concentrations (Fig. 3b). Induction increased expression of all three Equalizers by a maximum of four- to eight-fold. Equalizer-L produced lower expression variability but also lower expression. For example, Equalizer-H produced 5.2 and 22 times higher fluorescence than Equalizer-L at 0 and 30 ng/mL, respectively.

We compared the expression variability obtained above with Equalizers to the variability produced by commonly used promoters without control circuitry (i.e., open-loop), hereafter referred to as unregulated promoters. As expected, we observed higher variation with all unregulated promoters tested: the phosphoglycerate kinase (PGK, CV = 221%), the ubiquitin C (UBC, CV = 220%), and the cytomegalovirus (CMV, CV = 137%) promoters (Fig. 3c). The greater variability produced by the PGK and UBC promoters compared with the CMV promoter may be due to increased burstiness of the weaker PGK and UBC promoters^35^, saturation of gene expression due to limited cellular resources when using the strong CMV promoter, or both.

We next determined to what extent the cell-to-cell variability observed with Equalizer circuits was due to residual dependence on plasmid copy number rather than other sources of variation such as intrinsic noise^35^, differences in expression capacity^36^, or measurement noise. We, therefore, created a condition without copy-number variation by chromosomally integrating an EGFP expression cassette with the same promoter (CMV) as the Equalizer circuit. The variation produced by Equalizer-L and the CMV cell line were both similar (CV ~ 71%), demonstrating the potency of Equalizer-L at buffering plasmid copy number variability (Fig. 3c). We conducted subsequent experiments solely with Equalizer-L because it was the most effective of the three circuit variants at buffering gene dosage. Henceforth, all experiments with Equalizer-L were performed at the doxycycline concentration producing the lowest cell-to-cell variation (1 ng/ml), unless otherwise noted.

To evaluate whether Equalizer-L’s gene dosage compensation circuitry is functional in multiple cell types, we tested Equalizer-L in multiple commonly used mammalian cell lines derived from different species. Compared with the unregulated CMV promoter, Equalizer-L achieved lower cell-to-cell variability in all the cell types tested, including Neuro2A, a line of mouse neuroblasts; CHO-K1, a line of Chinese hamster ovarian cells; COS-7, a line of African green monkey kidney cells; and HeLa, a line of human cervical adenocarcinoma cells (Fig. 3d).

To confirm that our results were robust to the dose of transfected plasmids, we quantified expression heterogeneity following transfection with 1–200 ng of plasmids. The lower cell-to-cell variability of Equalizer-L was maintained across the entire range of plasmid doses (Fig. 3e, f). The mean gene-dosage reporter values did not increase linearly with the plasmid dose, leading to a smaller range of gene-dosage reporter values (Supplementary Fig. 7a). The mean circuit output level of Equalizer-L was intermediate between those of the PGK and CMV promoters for five of the six plasmid doses (Supplementary Fig. 7b). Therefore, the lower output variability produced by Equalizer-L is not simply due to its weaker expression compared with the CMV promoter. For each plasmid dose, the mean values and the overall distribution of gene-dosage reporter levels were similar between all three circuits, demonstrating that our results were not due to differences in transfection efficiency or expression capacity (Supplementary Fig. 8a).

We computed the mean circuit output and the mean gene-dosage reporter values for each plasmid dose. As expected, the resulting transfer curves showed that Equalizer-L compensates for increases in plasmid copy number at the population level. For example, over a 20-fold change in mean gene dosage, the mean circuit output levels of the Equalizer-L only increased 2.7-fold, compared with 15.7 and 10.4 for the PGK and CMV promoters, respectively (Fig. 3g).

Because our data were acquired by measuring individual cells, we could quantify circuit output over a wider range of gene dosages than when only considering population means. We pooled the single-cell data from experiments with each plasmid dose and quantified the mean circuit output of each 5-percentile bin of gene-dosage reporter values. In response to a 200-fold change in gene dosage, Equalizer-L circuit output increased ~4-fold compared with ~90-fold for PGK and ~50-fold for CMV (Supplementary Fig. 8b). Taken together, our experiments demonstrate that the Equalizer-L robustly buffers plasmid copy-number variation at both population and single-cell levels and produces output variation similar to chromosomal expression.

### NF and IFF loops act synergistically to widen the gene dosage range of effective compensation

**Fig. 4:**
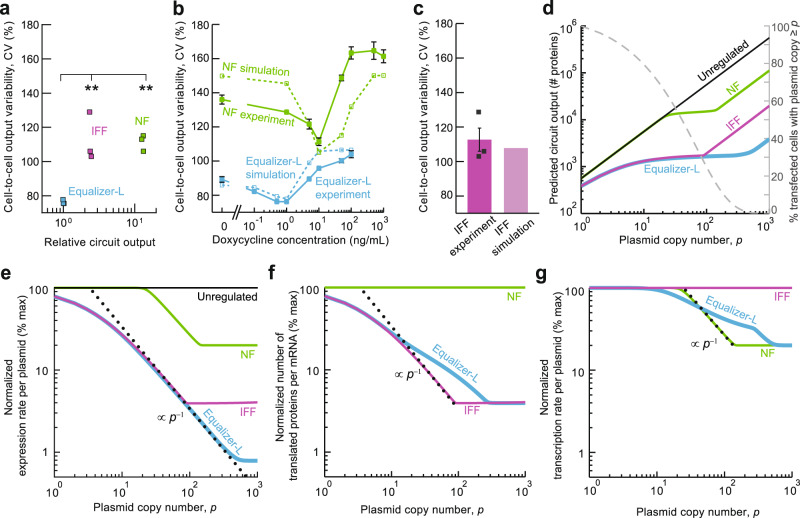
Equalizer-L combines NF and IFF circuitry to increase the range of gene dosage compensation. **a** Equalizer-L produced lower cell-to-cell variability than either NF or IFF circuits alone. In this experiment and in simulations (**d–g**), doxycycline was used at the concentration producing the lowest cell-to-cell variability for each circuit (Equalizer-L, 1 ng/mL; NF, 10 ng/mL). **p < 0.01 (Tukey’s multiple comparison test). Square markers indicate *n* = 3 independent transfections. Simulation results closely matched the experimentally determined cell-to-cell output variability of the NF (**b**) and IFF (**c**) circuits. For **b**, the filled markers indicate the mean of *n* = 3 independent transfections per construct. For **c**, the square markers indicate independent transfections. The error bars are the SEM. Each simulation datapoint (open markers) was computed from 10,000 cells whose plasmid copy number was sampled from the estimated plasmid copy number distribution. See Supplementary Notes 1, 3, and 5 for simulation models and methods. **d** Deterministic simulations predicted that Equalizer-L has a wider compensation range than standalone NF and IFF circuits. The dashed gray curve (right axis) illustrates the estimated plasmid copy number distribution. Simulated overall expression rate (**e**), number of proteins translated per mRNA (**f**), and transcription rate per plasmid (**g**) for each topology. The dotted lines indicate the slopes corresponding to perfect dosage compensation. Source data are provided as a Source Data file.

Having established Equalizer-L as an effective gene dosage compensation circuit, we experimentally confirmed that it outperforms the standalone NF and IFF subcircuits (Fig. 4a and Supplementary Figs. 9, 10), as originally predicted (Fig. 2 and Supplementary Figs. 2, 4). In this experiment, both the Equalizer-L and the NF circuit were induced with doxycycline at the concentration producing the lowest cell-to-cell variation (1 and 10 ng/ml for Equalizer-L and NF, respectively; Fig. 4b). The same inducer concentrations were also used in the ensuing simulations.

We next used computational modeling to understand how the NF and IFF subcircuits interplay within the Equalizer architecture. To this end, we first refined our initial models by using our experimental results. Using the distribution of expression for unregulated circuits and mean expression levels of NF circuit, we estimated the distribution of plasmid copy number following transient transfection and the leakage parameter of TetR repression. Comparing simulated and measured mean expression for the cells expressing the Equalizer and the NF circuits, we estimated the miRNA affinity to its target sites (Supplementary Notes 3 and 4). To account for the contribution of intrinsic noise^35^ to cell-to-cell variation, we assumed that the expression variability of the CMV cell line (Fig. 3d) was due solely to intrinsic noise. We further assumed identical intrinsic noise for the different circuits across all doxycycline concentrations (Supplementary Note 5). With these constraints in place, we could predict the cell-to-cell output variability with no free parameters.

Our simulation results closely matched the trends in cell-to-cell variability determined experimentally for the NF and Equalizer-L circuits in response to doxycycline (Fig. 4b). Simulations also accurately approximated the circuit output heterogeneity observed with the IFF circuit (Fig. 4c). This close agreement between simulated and experimental values was consistent with our claim that the Equalizer-L reduces cell-to-cell output variability primarily by buffering gene dosage variation rather than by reducing intrinsic noise. More generally, the close agreement between experimental results and simulations suggested that our model was suitable to study the gene dosage compensation properties of Equalizer-L.

We next used the model to predict the range of plasmid copy numbers over which Equalizer-L outperforms the NF and IFF subcircuits. We first overlaid the predicted plasmid copy number distribution with the predicted circuit output as a function of the plasmid copy number for the IFF, NF, and Equalizer-L circuits (Fig. 4d). Our model predicted that the variability in plasmid copy number between cells was wide, with ~99% of transfected cells harboring between 1 and 432 plasmids (Fig. 4d, gray curve). Equalizer-L was effective at buffering copy-number variation across the entire range, although with reduced potency at very low (≲5) and very high (≳500) plasmid copy numbers (Fig. 4d and Supplementary Fig. 11). In contrast, the NF circuit was limited by poor dosage compensation at both low and high plasmid copy numbers, while the IFF’s gene dosage compensation was predominantly impaired at high plasmid copy numbers. The shape of the predicted NF circuit output is different from that shown in Fig. 2d (Leaky NF) due to a difference in inducer concentration. Figure 4b was simulated with the inducer concentration producing the lowest cell-to-cell output variability of the (leaky) NF circuit (10 ng/mL), while Fig. 2d was generated using a doxycycline concentration optimized for the ideal NF circuit (1 ng/mL).

In an ideal plasmid dosage compensation circuit, protein expression per plasmid is inversely proportional to the plasmid copy number. In log–log plots, this ideal scaling corresponds to a straight line parallel to the dotted lines depicted in Fig. 4e–g. Equalizer-L compensated for gene dosage at or near this theoretical ideal across a wider range of plasmid copy numbers than the NF and IFF circuits (Fig. 4e). To determine which Equalizer-L subcircuit was responsible for gene dosage compensation in different plasmid copy number regimes, we plotted how the predicted post-transcriptional and transcriptional rates varied across the estimated range of plasmid copy numbers. At low copy numbers (≲10), dosage compensation was primarily provided by the IFF subcircuit of Equalizer-L (Fig. 4f), with negligible contributions from the NF subcircuit (Fig. 4g). At both intermediate (10^1^–10^2^) and high (>10^2^) plasmid copy numbers, the IFF and NF loops acted synergistically to provide overall dosage compensation close to the theoretical ideal.

The improved dosage compensation of the Equalizer-L at high plasmid copy numbers was due to stretching of both transcriptional and post-transcriptional dosage compensation curves compared with those of the standalone NF and IFF circuits, respectively (Fig. 4f, g). The predicted change in the post-transcriptional curve (Fig. 4f) is consistent with the NF subcircuit reducing transcription of miRNAs and their targets, thereby delaying saturation of RISC until higher copy numbers (Supplementary Fig. 12). The shallower but wider transcriptional dosage compensation curve of Equalizer-L compared with the standalone NF circuit (Fig. 4g) is consistent with the IFF subcircuit reducing TetR concentrations: TetR levels at which leakiness dominates are, therefore, only reached at higher copy numbers.

### Gene-dosage compensation of Equalizer-L is superior to an alternative circuit that combines miRNA-based NF and IFF topologies

**Fig. 5:**
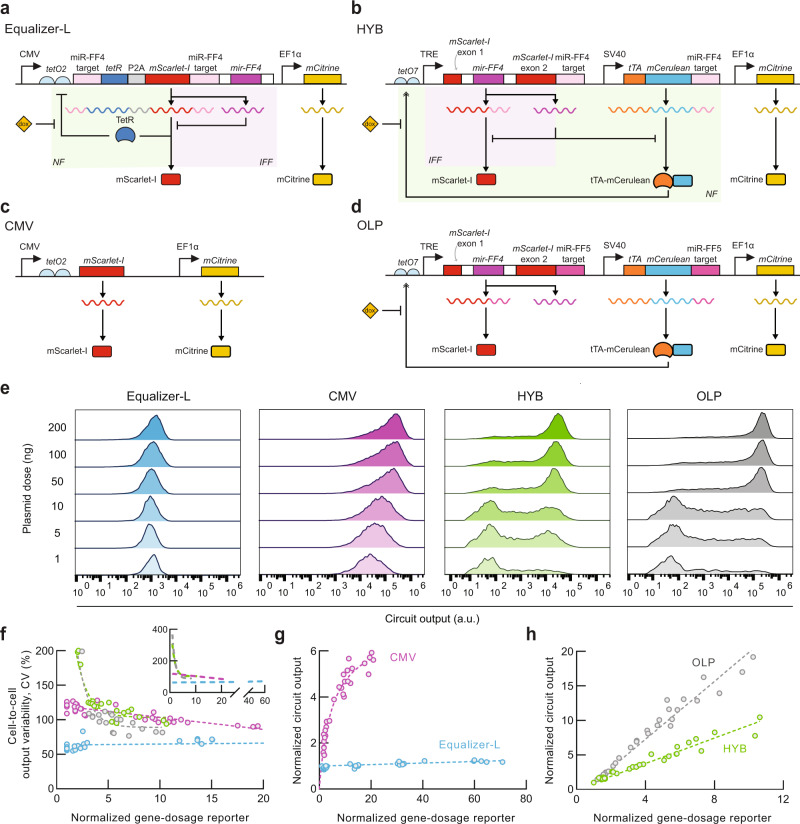
Equalizer-L has superior gene dosage compensation than an alternative circuit that combines post-transcriptional NF and IFF motifs. **a**–**d** Circuit schematics. mScarlet-I (RFP) and mCitrine (YFP) are reporters of circuit output and gene dosage, respectively. CMV (**c**) and OLP (**d**) do not have dosage compensation circuitry and are used as controls for Equalizer-L (**a**) and HYB (**b**), respectively. **e** Representative circuit output histograms. For all experiments (**e**–**h**), Equalizer-L was induced with 1 ng/mL of doxycycline. **f** HYB produces high cell-to-cell circuit output variability. The gene-dosage values were normalized to those obtained when transfecting 1 ng of plasmid. The circles represent independent transfections. *n* = 36 per circuit (6 per dose and 6 doses per circuit). The dashed lines are trend lines (linear for Equalizer-L and CMV; exponential for HYB and OLP). The inset shows the trend lines for the entire range of gene-dosage reporter levels. See Supplementary Statistics for statistical comparisons. Equalizer-L (**g**) showed superior gene dosage compensation than HYB (**h**) at the population level. The gene-dosage and circuit output values were normalized to those obtained when transfecting 1 ng of plasmid. Dashed lines indicate trend lines (hyperbolic for CMV and linear otherwise). Sample sizes are as in (**f**). *p* < 0.0001 for the two-sided Welch’s *t*-test comparing the trendline slopes of Equalizer-L and HYB. Source data are provided as a Source Data file.

While we were developing and characterizing Equalizer circuits, another NF-IFF hybrid circuit was reported^15^. This system, called HYB, was also proposed to compensate for plasmid copy-number variation. Although both HYB and Equalizer-L combine NF and IFF topologies, there are important differences in their implementation (Fig. 5a, b). First, while Equalizer-L expresses all the circuit components using a single promoter, HYB utilizes two promoters. Second, while Equalizer-L uses miRNAs solely in its IFF subcircuit, HYB utilizes miRNAs to regulate both its NF and IFF subcircuits. Third, the implementation of NF differed between the two circuits. In Equalizer-L’s NF loop, TetR directly represses the expression of its own gene and the circuit output. In contrast, HYB’s NF loop is mediated by miRNA-based repression of a transactivator that increases the expression of the output protein and the miRNA itself. Finally, the HYB circuit also includes a coherent feedforward loop since both the circuit output and its transactivator are encoded on the same plasmid. The Equalizer-L plasmid neither encodes a transactivator nor incorporates a coherent feedforward loop.

The differences between these two circuits presented a unique opportunity to evaluate how gene dosage performance could be affected by circuit design choices. We noticed that the two systems used different fluorescent protein reporters, thereby complicating their comparison. Therefore, we modified the Equalizer-L and HYB plasmids to express the same reporter: the red fluorescent protein mScarlet-I^37^ as the reporter of circuit output, and the yellow fluorescent protein mCitrine^38^ as the reporter of plasmid dosage. We chose these fluorescent proteins because of their monomericity, high brightness, and fast maturation time^37–41^. We also applied the same modifications to the unregulated controls: CMV for Equalizer-L and OLP for HYB (Fig. 5c, d). We conducted the following experiments in HEK293T cells because this cell line was used in the original study that reported HYB^15^.

We first confirmed that the above modifications did not impact the gene dosage capacity of Equalizer-L and that the optimal gene dosage compensation was still achieved with 1 ng/mL of doxycycline (Supplementary Fig. 13a). We also evaluated how the cell-to-cell variability and circuit output produced by the HYB plasmid varied with doxycycline concentration. We found that HYB produced the lowest cell-to-cell variability in the absence of inducer (Supplementary Fig. 13b). Therefore, we did not use doxycycline in subsequent experiments with HYB and OLP. HYB also produced the highest circuit expression when no inducer was added, as expected for a system that is repressed by doxycycline (Fig. 5b).

In contrast with expression from Equalizer-L and CMV, expression from HYB and OLP was largely bimodal and more steeply dependent on the plasmid dosage (Fig. 5e). Increasing the plasmid dose increased the proportion of cells in the high-expression peak. The distributions of gene-dosage reporter levels were not bimodal and, therefore, could not explain the bimodality of the HYB and OLP circuit output distributions (Supplementary Fig. 14a, b). Instead, the observed bimodality may have occurred because the tetracycline transactivator is encoded on the same plasmid as its cognate promoter (TRE). Since the TRE promoter used in both OLP and HYB is highly sensitive to transactivator level (Hill coefficient ~3.2 - see ref. ^32^), a modest change in plasmid concentration could enable cells to cross the threshold necessary for TRE activation.

We transfected different plasmid doses and quantified expression heterogeneity. Equalizer-L reduced cell-to-cell variability to similar levels as previously observed when using EGFP as the circuit output reporter (Figs. 5f and 3). However, HYB produced similar expression variability as OLP. We also quantified the resulting mean circuit output levels of populations of cells as a function of mean gene-dosage reporter levels. Equalizer-L showed excellent gene compensation, with only ~1.2-fold increase in the mean circuit output in response to a ~60-fold increase in apparent gene dosage (Fig. 5g). In contrast, while the mean output of HYB had a weaker dependence on gene dosage than OLP, HYB’s mean output levels remained nearly proportional to the change in gene-dosage reporter levels (Fig. 5h).

We also quantified gene dosage compensation at the single-cell level. HYB produced a ~180-fold change in expression in response to a ~100-fold increase in plasmid dosage, lower than the 320-fold change observed with OLP (Supplementary Fig. 14c). In comparison, over the same range, Equalizer-L increased only by approximately threefold. CMV increased by ~14-fold, producing a non-linear response to gene-dosage reporter values.

We conducted several control experiments and analyses to strengthen our claim that Equalizer-L provides superior dosage compensation than HYB. To determine whether the poor dosage compensation of HYB is due to the lower-expressing subpopulation in its bimodal distribution, we reanalyzed results considering only higher-expressing cells from experiments with HYB or OLP. We obtained similar results as above for both population and single-cell assays (Supplementary Fig. 14d–f). We also obtained similar cell-to-cell variability with the original (unmodified) HYB and OLP plasmids, demonstrating that our results are not due to changing the output protein from DsRed-Express to mScarlet-I (Supplementary Fig. 14g). HYB and OLP had comparable expression heterogeneity in CHO-K1 cells, showing that our results extend to other cell types than HEK293T (Supplementary Fig. 14g).

We replicated the previously reported finding that HYB has a lower Fano factor than its corresponding unregulated promoter, OLP (Supplementary Fig. 14h)^15^. However, Fano factors are not easily interpretable when comparing distributions with different means (Supplementary Note 7). For example, the PGK promoter produced a lower Fano factor than the CMV promoter (Supplementary Fig. 14i) despite producing larger CV values in our evaluations of cell-to-cell variability (Fig. 3f) and a nearly linear dependence on gene dosage (Fig. 3g). We also replicated the finding that HYB produces a flatter curve than OLP when these circuits are evaluated by plotting unnormalized circuit output values on a linear axis^15^ (Supplementary Fig. 14j, left). However, evaluation of gene dosage compensation using unnormalized values can be misleading, as weaker promoters will also produce flatter curves when plotted in this manner. For example, the dependence of circuit output on gene dosage appeared similar between Equalizer-L and the (weak) PGK promoter, despite PGK not compensating for gene dosage (Supplementary fig. 14j, right). Taken together, our results strongly suggest that Equalizer-L has superior gene dosage compensation capacity at the population and single-cell levels compared with HYB.

### A replicating variant of Equalizer-L enables simple, rapid, and versatile development of extra-chromosomal cell lines with low cell-to-cell expression variability

Transient transfection with most expression plasmids is only suitable for experiments lasting up to a few days: expression levels and the proportion of expressing cells peak on day 2 or 3 post-transfection and are substantially reduced by days 5 and 6^42^. However, some plasmids—called episomes—can replicate in mammalian cells. Episomes enable persistent gene expression and are compatible with many cell types^43^. However, episomes are expected to suffer from high cell-to-cell variability in circuit output as they undergo the same transfection process as nonreplicating plasmids. We reasoned that incorporating Equalizer-L in an episome would combine the simplicity and versatility of plasmid expression with the potential for long-term experiments with low expression heterogeneity that normally requires chromosomal expression.

To develop an episomal version of Equalizer, we repurposed plasmids that are derived from the Epstein–Barr virus and that replicate synchronously with the cell cycle^44^ at a copy number between 1 and 100, depending on the cell type^44,45^. Plasmid replication depends solely on two viral sequences: an origin of replication called *oriP* and the *oriP*-binding nuclear protein EBNA-1^44,46^. *oriP*-bound EBNA-1 also tethers plasmids to chromosomes, both to prevent plasmid loss during mitosis^47^ and to promote replication^48^. We, therefore, constructed an Equalizer episome by subcloning Equalizer-L and our gene-dosage reporter onto a plasmid with *oriP* and EBNA-1 (Supplementary Fig. 16).

Next, we evaluated the ability of the Equalizer-L episome to maintain constant gene expression and low cell-to-cell expression variability for multiple weeks. We transfected episomes in HEK293 cells and grew the cells for 2 months. We quantified the fluorescence of individual cells every 1–2 weeks using flow cytometry. The boundaries between expressing and nonexpressing cells were defined using untransfected cells and control cultures expressing a single fluorescent protein (Supplementary Fig. 17). We also took representative images of the cells under fluorescence microscopy (Supplementary Fig. 18). In the absence of selection, plasmid loss is reported to be between 2 and 5% per generation^44^. The episome expresses a hygromycin B resistance gene, and we prevented the emergence of plasmid-free cells by using growth media with antibiotics starting 1 day after transfection.

**Fig. 6:**
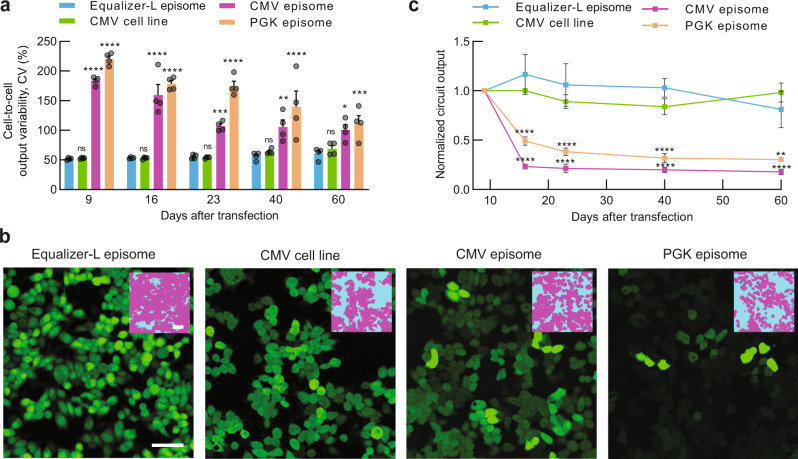
A replicating variant of Equalizer-L enables the development of extra-chromosomal cell lines that have stable gene expression with low cell-to-cell variation. **a** Over the course of >8 weeks, the Equalizer-L episome produced similar cell-to-cell variation as a chromosomally integrated CMV cassette (CMV cell line) and lower variation than episomes with unregulated promoters. HEK293 cells were used. For all experiments **a**–**c**, Equalizer-L was induced with 1-ng/mL doxycycyline. The error bars represent SEM. *n* = 4 independent trials. Tukey’s multiple comparison test was used to compare the Equalizer-L episome with the other conditions. ns not significant; *p < 0.05; **p < 0.01; ***p < 0.001; ****p < 0.0001. **b** Representative images of HEK293 cells expressing EGFP from episomes or the chromosome at 23 days post transfection. Each image is displayed with a linear lookup table with the minimum set to 0 and the maximum set to the sum of the mean intensity value and three standard deviations (see Methods). This approach enables a qualitative comparison of the cell-to-cell expression variability despite large differences in mean circuit output. Insets, binary masks to help identify regions of the images that correspond to cells (magenta region). Scale bar, 50 μm. See Supplementary Fig. 18 for images acquired on other days. **c** The circuit output levels from the Equalizer-L episome were stable for >50 days, whereas episomes with unregulated promoters displayed pronounced declines. Each circuit’s mean output levels were normalized to levels at 9 days post transfection. Induction, sample sizes, error bars, and statistical tests are as in (**a**). Source data are provided as a Source Data file.

For the entire duration of the 2-month experiment, cells expressing the Equalizer-L episome had indistinguishable cell-to-cell variability from a cell line expressing a chromosomally integrated CMV expression cassette (Fig. 6a, b and Supplementary Fig. 18). The average circuit output also remained relatively constant, similar to what we observed with the (chromosomal) CMV cell line (Fig. 6c and Supplementary Fig. 20a). The cell-to-cell variability observed with episomes expressing the CMV or PGK promoters (Supplementary Fig. 16b, c) was higher throughout the experiment. From day 9 to day 60 post transfection, the CMV and PGK episomes also produced 70% and 82% decreases in gene expression, respectively (Fig. 6c). Expression from the gene-dosage reporter also decreased by 53–81% between day 9 and day 60 for all plasmids (Supplementary Fig. 20b, c). These changes likely reflect a decrease in copy number due to imperfect plasmid replication and segregation: while antibiotic selection prevents the growth of plasmid-free cells, a reduction in the number of plasmids per cell can occur. These presumed changes in the copy number distribution may explain why the cell-to-cell output variability of the CMV and PGK episomes decreased over the 2 months of the experiment (Fig. 6a). However, because gene-dosage reporter values were low on several days, we could not accurately quantify changes in plasmid copy number distributions. As predicted, the Equalizer-L episome buffered these fluctuations, producing circuit output that remained largely invariant over the same timescale (Fig. 6c).

Despite the presence of an RFP gene-dosage reporter on all episomes, all cultures showed a significant fraction of cells with detectable circuit output (GFP^+^) but undetectable gene-dosage reporter values (RFP^−^) (Supplementary Fig. 19). These apparent GFP^+^ RFP^−^ cells may result from imperfect detectability of the RFP (mCherry) at lower plasmid concentrations, from silencing of the EF1-*α* promoter driving the gene-dosage reporter^49^ or, less likely, from genomic instability^50^. The fraction of GFP^+^ RFP^−^ cells was particularly high in the CMV episome cultures, where they accounted for 32–71% of GFP^+^ cells. We hypothesize that the strong CMV promoter created stronger selective pressure for lower plasmid concentrations, reduced available cellular machinery available for expressing the gene-dosage reporter, or both. Consistent with these explanations, the mean RFP expression of RFP^+^ cells with the CMV episome was ~1.7–4.4-fold lower than that observed with the weaker-expressing PGK and Equalizer-L episomes (Supplementary Fig. 20b). While only RFP^+^ cells were analyzed above (Fig. 6a, c and Supplementary Fig. 20a–c), including GFP^+^ RFP^−^ cells in our analyses resulted in similar trends in cell-to-cell expression variability and mean circuit output (Supplementary Fig. 20d, e). Regardless of the emergence of these subpopulations, most cells robustly expressed Equalizer-L for 60 days.

## Discussion

A critical goal of synthetic biology is to design systems with predictable functions^8^. However, uniform expression of even single genes across a population of mammalian cells remains challenging. As variations in plasmid copy number is a key factor driving expression heterogeneity following transient transfection, approaches to reduce the dependence of expression on the abundance of encoding genetic material are needed^8,9^. To this end, this study reports new mammalian genetic circuits, called Equalizers, that buffer circuit output from variation in plasmid copy number. Cell-to-cell expression variability with Equalizers is equivalent to that observed in cell lines that harbor chromosomally integrated reporters and are thus not subject to plasmid copy-number variation (Figs. 3c and 6a). Robust gene dosage compensation was displayed at both the population and single-cell levels, in multiple cell lines (Fig. 3d), and across a wide range of transfected plasmid doses (Figs. 3e–g and 5).

We also reported that, when encoded on an episome, Equalizer-L enables stable expression over multiple weeks of growth with as low cell-to-cell variability as chromosomal cell lines (Fig. 6). Episomal cell lines can be generated by simple transfections, followed by a short period of antibiotic selection, and are compatible with a wide array of cell types^51–54^. Therefore, this method is rapid, versatile, and accessible to all labs without specialized skills in chromosomal integration techniques. After ~40 (PGK) or ~60 (Equalizer-L) days post transfection, we observed the emergence of cells that were no longer expressing the reporter of circuit output (EGFP) despite continued expression of mCherry, our reporter of plasmid dosage (Supplementary Fig. 19). This decrease in circuit output may be due to silencing of the CMV promoter used in Equalizer-L, as previously reported^55^. Alternative promoters or CMV variants^56^ that are less prone to silencing should be evaluated for experiments lasting longer than 1.5–2 months.

Our results highlight how the nonideal behavior of circuit components must be considered when designing gene circuits. For example, incomplete repression of gene expression at high TetR concentration was predicted to strongly impair gene dosage compensation of the NF-only circuit at high plasmid copy numbers (Fig. 2d and Supplementary Fig. 2). Limitations in cellular resources are another design consideration; for example, simulations suggest that miRNA-based IFF circuits are limited at high plasmid copy numbers due to low availability of free RISC (Fig. 2b and Supplementary Fig. 12). The Equalizer circuits achieve robust performance by combining two imperfect subcircuits, each with distinct limitations and complementary gene dosage compensation ranges (Fig. 4d–g).

Our results also illustrate that simply incorporating NF and/or IFF loops is insufficient to reduce expression heterogeneity following transient transfection. For example, despite encoding both an NF and an IFF subcircuit, the HYB plasmid^15^ did not reduce overall cell-to-cell variability (Fig. 5f and Supplementary Fig. 14f, g). A contributing factor may be the dependence of expression on a transactivator located on the same plasmid. The resulting coherent feedforward loop is expected to amplify the existing dependence of gene expression on copy number. Consistent with this hypothesis, the mean circuit output from cells with the OLP plasmid increased faster than the change in apparent gene dosage (Fig. 5h). A second factor may be that HYB and OLP express their circuit components using two promoters, a configuration that is predicted to increase intrinsic noise^57,58^. Consistent with this explanation, a variant of the Equalizer-L where TetR, the miRNA, and the reporter gene are expressed from separate promoters produced higher cell-to-cell variation despite deterministic simulations predicting identical gene-dosage compensation capacity ((Supplementary Fig. 15; Supplementary Note 6). Finally, additional cell-to-cell variation may have been caused by plasmid replication: we found that the SV40 promoter encoded by HYB and OLP also includes the SV40 origin of replication. This origin is thought to be mediate plasmid replication uncoupled from the cell cycle^43^ in HEK293T—the cell line used here^59^ and in the original report of HYB^15^. The vectors used for transient transfection of Equalizer-L or CMV are not replication-competent, and those used for stable transfection can replicate synchronously with the cell cycle^44^.

While Equalizers are promising circuits for uniform expression at the single-cell level, further improvements and characterization would be desirable. First, future efforts should focus on increasing the expression levels. The topologies deployed here are inhibitory: an ideal circuit reduces the expression level of cells with multiple plasmids to match the level with a single plasmid. For example, Equalizer-L produced up to 50 times lower expression than the unregulated CMV promoter (Supplementary Fig. 7b). As expected, the gap narrowed down to ~7-fold at the lowest plasmid dose tested. A second design goal is the development of circuits that do not modify the sequence of the protein of interest. In Equalizers, the 2A ribosome-skipping sequence (Fig. 2e) adds a proline to the N terminus of the protein of interest. Moreover, a small amount of read-through can occur, producing fusions with TetR^27^. 2A could be substituted with an Internal Ribosome Entry Site, although with increased intrinsic noise^58^ and lower expression^60^. Finally, it would be useful to investigate the Equalizers’ ability to adapt to changes in cellular resources, akin to circuits recently reported^16,61^.

In natural systems, chromosomal replication and volume changes during cell growth create variation in gene copy numbers or concentration. Some gene networks must buffer this variation to remain functional and conserve their properties^17–20,62,63^. For example, through the cross-regulation of its component proteins, the yeast galactose (GAL) pathway remains similarly inducible after halving the dosage of the entire GAL network^17,19^. The GAL dosage compensation topology also reduces cell-to-cell variation in pathway activity^18^. Equalizers employ typologies similar to the GAL system and other natural networks. A valuable future direction would be to adapt the mechanisms of natural systems and Equalizers to compensate for dosage variation of multigene synthetic systems.

In summary, we have developed synthetic circuits that near-perfectly buffer variation in plasmid copy number between individual mammalian cells. We anticipate that Equalizers will be rapidly adopted by the biomedical and synthetic biology communities, providing a simple-to-use, robust, and versatile solution to achieving uniform gene expression at the single-cell level.

## Methods

### Plasmid construction

All new plasmids were generated using standard molecular biology methods and were verified by sequencing. Plasmids used in this study are available from Addgene (169367, 169731–169735, 169737–169748, 170041) and their sequences are available from GenBank (MW962296–MW962297, MW987521–MW987522, MW987525-MW987527, MW987529-MW987537, MZ099631, MZ220609-MZ220611). pDN-D2ir_mCherry_P2A_TetR:EGFP was obtained from D. Nevozhay & G. Balázsi and was used to amplify *tetR* and its cognate *tetO2* binding site. pTRE-Tight-BI-DsRed-miR-FF3/tgt-FF3-AmCyan-FF3^14^ were used to amplify miR-FF3 and their binding sites. miR-FF4 was cloned with miR-FF3 as template and using two long primers 5′-ACATCTGTGGCTTCACTATTTAATTAAAGACTTCAAGCGGCGCTCACTGTCAACAGCAC-3′ and 5′-TGAAGCCACAGATGTATTTAATTAAAGACTTCAAGCGGTGCCTACTGCCTCGGAGAATT-3′ that modified the core sense/antisense sequence from miR-FF3 to miR-FF4^22^. pCEP4-CXCR4 was obtained from Addgene (Plasmid #98944) and was used to subclone the episome plasmids. HYB (pGLM127) and OLP (pGLM130) plasmids are described in ref. ^15^ and were obtained from Dr M. Khammash. We noticed that the miR-FF4 used in HYB and OLP circuits had several nucleotide differences to the miR-FF4^22^, which we used as a reference to build our circuit plasmids. The mutations were c.1T>A; 4A>T; 5G>A; 35C>T. Some of the unregulated promoter constructs have different 5′ and 3′ UTRs. We have shown that these differences have minimal impact on cell-to-cell variation (Supplementary Fig. 21). Schematics of plasmid constructs are in Supplementary Figs. 5 and 16 and the entire list of plasmids used in this study is in Supplementary Table 4.

### Cell lines

The Flp-In^TM^ 293 (RRID:CVCL_U421, Thermo Fisher Scientific) cell line was primarily used in our study. In the text, we call this cell line as HEK293 for simplicity. Other mammalian cell lines used in this study were HEK293A (RRID:CVCL_6910, Thermo Fisher Scientific), CHO-K1 (CCL-61, ATCC), HeLa cells (CCL-2, ATCC), HEK293T (CRL-3216, ATCC), COS-7 (CRL-1651, ATCC), and N2A (CCL-131, ATCC). These cell lines were free of mycoplasma contamination. All the cell lines, except CHO-K1 cells, were maintained in high-glucose Dulbecco’s Modified Eagle Medium (DMEM, D1145, Sigma-Aldrich) supplemented with 10% fetal bovine serum (FBS, F2442, Sigma-Aldrich), 2-mM glutamine (G7513, Sigma-Aldrich), and 100-unit/mL penicillin-streptomycin (P4333, Sigma-Aldrich) at 37 ^∘^C in air with 5% CO_2_. We call the growth media described above as fully supplemented DMEM hereafter. We confirmed with the manufacturer that the FBS did not contain any residual doxycycline. For the culture media for HEK293 cells, we added Zeocin^TM^ (100 μg/mL, R25005, Thermo Fisher Scientific) to the fully supplemented media. CHO-K1 cells were cultured using DMEM/Nutrient Mixture F-12 (11320033, Thermo Fisher Scientific) supplemented with 10% FBS, 2-mM glutamine, and 100-unit/mL penicillin-streptomycin. The creation and maintenance of episomal cell lines are described in a separate section below.

To generate the cell line that expressed the reporter of circuit output (i.e., EGFP) from the chromosome, we used the Flp-In^TM^ system. Flp-In^TM^ 293 cells (i.e., HEK293 cells) were plated in a six-well plate for a confluence of 70% 1 h before transfection. Cells were then co-transfected (using 6:1 mass ratio, respectively) with pOG44 plasmid (V600520, Thermo Fisher Scientific) and an unregulated CMV expression plasmid encoding EGFP, hygromycin B resistance gene (*hph*), and a FRT site. A total 4.5 μg of plasmid DNA was added to each well with 155 μL of Opti-MEM^TM^ (11058021, Thermo Fisher Scientific) and 13.5 μL of FuGene HD (E2311, Promega, Madison, WI). Twenty-four hours after transfection, fully supplemented medium in each well was replaced to reduce the possible cytotoxicty caused by the transfection reagents. Forty-eight hours after transfection, medium was removed from each well and replenished with fresh medium containing 100-ng/μL hygromycin B (10687010, Thermo Fisher Scientific). Same medium was replaced every 2–3 days until attached colonies could be identified and grew to 70–80% confluency. Cells were then passaged to a 10-cm culture dish or stored in liquid nitrogen for future use. The EGFP expression plasmid used for the genome integration had the same promoter and 5′ UTR as those of the Equalizer, the IFF, and the NF plasmids.

### Transient transfection

Transfections were carried out using FuGene HD according to the manufacturer’s instructions (0.6-μL reagent:200-ng DNA per well for 96-well plates).

For flow cytometry experiments, cells were transfected in glass-bottom 96-well plates (P96-1.5H-N, Cellvis). Three hours before transfection, the plates were coated with 60 μL per well of 0.1 mg/mL of poly-L-lysine and incubated for an hour. After removing the poly-L-lysine, the wells were washed with 1x Dulbecco’s phosphate-buffered saline (DPBS) without calcium and magnesium (21-031-CV, Corning). Seventy microliters of cells in fully supplemented DMEM were then plated in each well to achieve ~60% confluency. The plates were incubated a 37 ^∘^C with 5% CO_2_ air for 1–2 h to promote cellular attachment prior to transfection. Among the 200 ng of plasmid DNA transfected per well, 100 ng were circuit plasmids, and the other 100 ng were transfection dosage control plasmid that encoded a fluorescent protein with minimal overlap (i.e., mCherry) with the reporter fluorescent protein (EGFP) encoded on the circuit plasmids. This control plasmid does not contain any *TetO* binding sites or miRNA targets sites and thus expression of the mCherry is not under control of the Equalizer. For most experiments, the mCherry expression cassette was cloned into the circuit plasmids. In this case, 100 ng of circuit plasmid with the onboard mCherry expression cassette and 100 ng of empty vector plasmid (that did not encode any genes) were used per well. For experiments, which we varied the transfecting plasmid doses, we used 1–200 ng of circuit plasmids. Appropriate amount of empty vector plasmid was added so that the total transfecting plasmid amount was 200 ng per well.

For each well, plasmid DNA was mixed with Fugene (with 200-ng to 0.6-μL ratio) in 12.5-μL Opti-MEM. After incubating the mixture at room temperature for 6–8 min, 27 μL per well of fully supplemented DMEM was added. Thirty microliters of the resulting mixture was added to the wells that had 70 μL of cell suspension. The plate was gently shaken to ensure that the reagents were well mixed. For inducible constructs (Equalizers or NF circuit), 2–4 h after transfection, 50 μL of doxycycline diluted in fully supplemented DMEM was added to achieve the desired inducer concentrations. Fifty microliters of fully supplemented media without doxycycline was added to wells that did not require induction.

### Transfection of episomal plasmids and cell culture of episomal cell lines

HEK293 cells were plated in a six-well plate at a confluency of 70% an hour before transfection. For each well, a transfection mixture was prepared by mixing 2000 ng of episomal plasmid (Equalizer or unregulated promoter) with 50 μL of Opti-MEM. Then, 6 μL of Fugene was added to the mixture and incubated at room temperature for 6–8 min. After the incubation, the transfection mixture was added to the plated cells. Twenty-four hours after transfection, the transfected cells on the 6-well plate were detached and replated on two 96-well plates: one for imaging and another for flow cytometry. For the imaging plate, cells were plated at a confluency of 20–30% and for the flow cytometry plate, cells were plated at a confluency of 40–50%. The cells in the six-well plate were also passaged to another six-well plate at a confluency of 30% to maintain the episome cell cultures. The episome cell cultures were grown and maintained as described above for the entire duration of the experiment. Cells were passaged twice per week, and during every passage, fresh hygromycin B (50 ng/μL) was replenished to select for and maintain the cells transfected with the episomal plasmids. Note that the epsisomal plasmids express a hygromcyin B resistance gene (*hph*). Among the cells that were plated in 96-well plates, cells that were transfected with the Equalizer-L episome were induced with 1-ng/mL doxycyline at 2–4 h after plating. Forty-eight hours after induction, culture medium in the imaging plate was replaced with Hanks’ Balanced Salt Solution (HBSS, H8264, Sigma-Aldrich) and cells were imaged using two-photon microscopy (2PM) setup described in the “Fluorescence microscopy” below. The cells in the flow cytometry plate were prepared and analyzed as described in the “Flow cytometry” below. Every week or 2 weeks for 2 months, the episome harboring cells were plated on the 96-well plates for imaging or flow cytometry.

### Flow cytometry

Thirty-six to forty-eight hours after transfection, cells were detached using trypsin (T3924, Sigma-Aldrich) and washed twice with 1x DPBS without calcium and magnesium. Detached cells were resuspended in 1x DPBS without calcium and magnesium and deposited into 96-well deep well plates. Attune NxT Acoustic Focusing Cytometer with the Autosampler (Thermo Fisher Scientific) was used to measure the fluorescence of individual cells. The following lasers and emission filters were used: for mCerulean, a 405-nm laser and a 440/50-nm emission filter; for EGFP and mCitrine, a 488-nm laser and a 530/30-nm emission filter; for mCherry, DsRed-Express, and mScarlet-I a 561-nm laser and a 620/15-nm emission filter. For each sample, 5000–10,000 cells were typically measured. Cells expressing one type of FP (single-FP controls) were prepared to compensate for bleed-through between the color channels. For the episomal Equalizer-L experiment (Fig. 6) that involved sampling of cells on multiple days for a 2-month period, we measured stable fluorescent beads (RFP-38-5, Spherotech) to ensure that the optical setup of the flow cytometer was the same throughout the entire duration of the experiment.

### Microscopy

Thirty-six to forty-eight hours after transfection, cells were washed once with 1x DPBS without calcium and magnesium. The media was then switched to 100 μL/well of HBSS supplemented with 10-mM HEPES. Cells were then imaged with an A1R MP+ microscope (Nikon Instruments) fitted with a 20x 0.75-NA dry objective and driven by the software NIS-Elements version 4.6 (Nikon Instruments). 2PM was used to image cells with a shallower depth of focus, to reduce apparent variation in fluorescence due to height differences between cells. 2PM experiments used a galvanometric mirrors to steer a titanium:sapphire Chameleon Ultra II laser (Coherent). GFP was excited with 920-nm light. The emission light was filtered by a 525/50-nm filter and collected using a gallium arsenide phosphide detector. For two-photon laser-scanning experiments, laser power and gain were adjusted for different constructs so that the brightest pixels were below pixel saturation. For Supplementary Fig. 18, the replicating Equalizer-L image was acquired with 5% gain, 30% power, 6.2-μs dwell time, and 2× averaging; the open-loop images were taken with 1% gain, 5% power, 6.2-μs dwell time, and 2× averaging.

For wide-field one-photon microscopy experiments (Supplementary Fig. 3b), GFP was excited with 470/20-nm light (SpectraX, Lumencore). Emission light was collected by a scientific CMOS camera (Flash4 v2+, Hamamatsu) after passing through a Multiband Filter (SpectraX, 77074159).

### Data processing

Flow cytometry data were collected using Attune NxT Acoustic Focusing Cytometer Software (version 4.0.1445.0, Thermo Fisher Scientific), and analyzed using FlowJo (version 10.6.1, BD). Forward and side scatters were used to gate singlet cells. Among the singlet cells, only the transfected cells were used for analysis unless mentioned otherwise. Circuit output levels of individual cells were evaluated using the fluorescence levels of reporter fluorescent proteins (EGFP or mScarlet-I) expressed by the circuit plasmids. mCherry or mCitrine expressed from an independent expression cassette was used to determine transfected cells by gating for cells that show higher mCherry or mCitrine fluorescence than the baseline non-transfected cells. mCherry and mCitrine fluorescence levels of individual cells were also used to estimate the active-plasmid copy number (i.e., gene dosage) inside the transfected cells. The CV values of EGFP or mScarlet-I fluorescence distributions were used to measure the cell-to-cell variability in circuit output. CV was calculated by dividing the SD of fluorescence values by the mean fluorescence value of the transfected cells. To evaluate gene dosage compensation at a population level, we transfected cells with different plasmid doses to vary the average plasmid copy number inside the transfected cells. We then determined how the mean circuit output levels varied as a function of mean active-plasmid copy number. We also evaluated population-level gene dosage compensation by pooling the single-cell data points of cells transfected with different plasmid doses (e.g., Supplementary Fig. 14c). Pooled data were divided into 20 bins with equal data points. For each bin, we computed the mean fluorescence and normalized each mean value to that of the first bin. This approach was used for both axes. Normalization of mean values was conducted when appropriate and the details of normalization are noted in the figure captions.

MATLAB (version r2019b, MathWorks) was used for quantitative (e.g., Supplementary Fig. 6) and qualitative (e.g., Fig. 6b) assessment of images. For quantitative analysis, image segmentation was conducted using ilastik^64^ to distinguish the cells from the background. Smoothing and background subtraction were applied on the raw images. Segmentation masks were then used to evaluate the fluorescent protein intensity of individual cells. The mean, standard deviation, and CV values of fluorescent protein intensities of segmented cells were calculated. Each field of view had 200–1000 cells. Fields of view with saturated pixels were removed from analysis. For qualitative analysis of fluorescence images, we first conducted image segmentation, as mentioned above, to obtain the means and standard deviations of fluorescence intensities of cells in the fields of view. We then systematically set the lookup table boundary for each field of view so that the boundary was centered around the mean fluorescence intensity of the cells in the field of view. More specifically, for each field of view, we set the lower bound to zero and the upper bound to mean fluorescence value plus three times the standard deviation value. The masks shown in Fig. 6b and Supplementary Fig. 18 were generated by thresholding. Note that these masks were not used for image segmentation, but simply to visualize regions of the images that corresponded to cells.

MATLAB and Prism (version 9.0.1, GraphPad) were used to conduct basic calculations, generate plots, and conduct statistical analysis.

### Statistical analysis

Statistical analysis was conducted to compare the mean cell-to-cell variability or circuit output values of *n* = 3–9 independent transfections. When comparing the means of two groups, we performed the unpaired two-sided *t*-test. For experiments that compared the means of more than two groups, we used the ANOVA. Prior to the *t*-test, one-way and two-way ANOVA, we conducted the F-test, Brown–Forsythe test, and Spearman’s test, respectively, to compare the variances of the groups. When the variances were statistically different, the Welch’s correction was applied when appropriate. Because normality tests have low power when the sample size (*n*) is small^65^, we did not conduct normality tests and assumed normality. For one-way and two-way ANOVAs, we conducted post hoc multiple comparison tests (Tukey, Sidak, or Dunnett). In the figures, *p* values are annotated as: *p < 0.05, **p < 0.01, ***p < 0.001, ****p < 0.0001. The details of all statistical analysis can be found in Supplementary Statistics.

### Computational modeling

MATLAB and MATLAB Simbiology were used for modeling and deterministic simulations of the study (see Supplementary Note 1). Stochkit2^66^ was used for stochastic simulation (see Supplementary Note 6). Model reactions and assumptions are listed in these Supplementary Notes. Simulation parameters are included in Supplementary Tables 1–3. Simulations in Fig. 2 used 1-ng/mL doxycycline for the ideal NF circuit, the leaky NF circuit, and Equalizer. Simulations in Fig. 4d–g used 10 ng/mL of doxycycline for the NF circuit and 1 ng/mL for Equalizer-L. Figure 2b, f and Supplementary Fig. 4 used miRNA dissociation rate constant of 0.3 s^−1^ for the IFF circuit and Equalizer as an in silico proof of concept, before Equalizer was experimentally tested. Unless specified otherwise, parameter values listed in Supplementary Tables 1–3 were used in the simulations.

Gene-dosage compensation was predicted using the inverse of log sensitivity of steady-state protein concentration to DNA copy number by varying the copy number by ±1 plasmid. In other words, this measure is the ratio of relative changes in gene dosage and relative change in gene expression. The higher this number, the better the circuit can maintain the same protein expression with changes in gene dosage. For instance, dosage compensation of 4 implies that a 100% (i.e., 2×) increase in gene dosage will lead to ~100%/4 = 25% increase in expression. To calculate the log sensitivity, each Simbiology circuit model was ran to steady state at individual copy number, and the log sensitivity at each copy number was calculated using numerical differentiation with second-order schemes (keeping values of DNA copy number, ${\rm{CN}}$, integer). For the copy number 1, a second-order forward finite difference was used to approximate the local log sensitivity ([POI] denotes steady-state protein concentration)

$${\left.\frac{\partial {\rm{log}}[{\rm{POI}}]}{\partial {\rm{log}}{\rm{CN}}}\right|}_{{\rm{CN}} = 1}={\left.\frac{{\rm{CN}}}{[{\rm{POI}}]}\frac{\partial [{\rm{POI}}]}{\partial {\rm{CN}}}\right|}_{{\rm{CN}} = 1}\approx \frac{-{[{\rm{POI}}]}_{{\rm{CN = 3}}}+4{[{\rm{POI}}]}_{{\rm{CN}} = 2}-3{[{\rm{POI}}]}_{{\rm{CN}} = 1}}{2{[{\rm{POI}}]}_{{\rm{CN}} = 1}}$$

For the highest copy number simulated (CN = *n*), a second-order backward finite difference was used to approximate the local log sensitivity

$${\left.\frac{\partial {\rm{log}}[{\rm{POI}}]}{\partial {\rm{log}}{\rm{CN}}}\right|}_{{\rm{CN}} = n}={\left.\frac{{\rm{CN}}}{[{\rm{POI}}]}\frac{\partial [{\rm{POI}}]}{\partial {\rm{CN}}}\right|}_{{\rm{CN}} = n}\approx \frac{n(-3{[{\rm{POI}}]}_{{\rm{CN}} = n}-4{[{\rm{POI}}]}_{{\rm{CN}} = n-1}+{[{\rm{POI}}]}_{{\rm{CN}} = n-2})}{2{[{\rm{POI}}]}_{{\rm{CN}} = n}}$$

For intermediate copy numbers (${\rm{CN}}=i$), a centered finite difference was used to approximate the local log sensitivity

$${\left.\frac{\partial {\rm{log}}[{\rm{POI}}]}{\partial {\rm{log}}{\rm{CN}}}\right|}_{{\rm{CN}} = i}={\left.\frac{{\rm{CN}}}{[{\rm{POI}}]}\frac{\partial [{\rm{POI}}]}{\delta {\rm{CN}}}\right|}_{{\rm{CN}} = i}\approx \frac{i({[{\rm{POI}}]}_{{\rm{CN}} = i+1}-{[{\rm{POI}}]}_{{\rm{CN}} = i-1})}{2{[{\rm{POI}}]}_{{\rm{CN}} = i}}$$

All computational and experimental data regarding NF topology shown in our study refer to the Equalizer (-L, -M, or -H) without the miRNA, its flanking splice sites and its target(s). For modeling the Equalizer and NF circuits, different inducer concentrations were supplied in the initial conditions to identify the optimal inducer concentration that produces the lowest log sensitivity.

While leakiness of each inducible construct can be conceptualized as the ratio of the expression level when no inducer was added to the maximum expression achieved by adding a saturated amount of inducers, it depends on the number of plasmids in a cell, because cells with different plasmid copy number will have different TetR concentrations without inducers, leading to different basal transcription rate per plasmid. Leakiness is considered in our modeling as the leakage parameter, as described in the end of Supplementary Note 1. See Supplementary Note 3 for the estimation of leakage value.

To approximate the miRNA-target affinity used in the models, the mean expression level of ten thousand cells with a fitted plasmid copy number distribution (Supplementary Note 3) was simulated with the Equalizer model (topology 4 in Supplementary Note 1) and the NF model (topology 2 in Supplementary Note 1) across doxycycline concentration of 0, 1, 5, 10, 50, 100 ng/mL. MATLAB’s fminsearch function was used to find the miRNA-target affinity that produces smallest mean squared error of the simulated mean expression ratio of the Equalizer model and the NF model compared with experimental data (see Supplementary Note 4 for details).

### Reporting summary

Further information on research design is available in the Nature Research Reporting Summary linked to this article.

## Supplementary information

Supplementary Information

Reporting Summary

## Data Availability

Annotated plasmid sequences have been deposited in GenBank with the accession codes: MW962296, MW962297, MW987521, MW987522, MW987525, MW987526, MW987527, MW987529, MW987530, MW987531, MW987532, MW987533, MW987534, MW987535, MW987536, MW987537, MZ099631, MZ220609, MZ220610, MZ220611. Plasmids used in this study can be obtained from Addgene (#169367, 169731–169735, 169737–169748, 170041). Detailed information on statistical tests is available in Supplementary Statistics. They are also available on GitHub: https://github.com/stpierrelab/Equalizer/tree/main/ExperimentData. Raw microscopy image files for Fig. 6 and Supplementary Figs. 3, 18 are provided with this paper. All other data are available upon reasonable request. Source data are provided with this paper.

## Source data

Source Data

## Code availability

MATLAB SimBiology models of gene dosage compensation topologies are available through the Equalizer GitHub repository at: 10.5281/zenodo.4741005^67^.
