# Supplementary material for: A synthetic circuit for buffering gene dosage variation between individual mammalian cells
Source: Nat Commun. 2021 Jul 5;12:4132. doi: 10.1038/s41467-021-23889-0 (PMC8257781; doi:10.1038/s41467-021-23889-0)
Supplement: Supplementary file 1 — Supplementary Information [file 41467_2021_23889_MOESM1_ESM.pdf]

Supplementary Information for:

A synthetic circuit for buffering gene dosage variability between individual mammalian cells

Jin Yang[1,†,‡], Jihwan Lee[2,†], Michelle A. Land[3], Shujuan Lai [3], Oleg A. Igoshin[1,2,4,5], François St-Pierre[2,3,6,7,*]
1 Department of Bioengineering, Rice University, 6100 Main St., Houston, 77005, TX, USA
2 Systems, Synthetic, and Physical Biology Program, Rice University, 6100 Main St., Houston, 77005, TX, USA
3 Department of Neuroscience, Baylor College of Medicine, 1 Baylor Plaza, Houston, 77030, TX, USA
4 Department of Biosciences, Rice University, 6100 Main St., Houston, 77005, TX, USA
5 Department of Chemistry, Rice University, 6100 Main St., Houston, 77005, TX, USA
6 Department of Electrical and Computer Engineering, Rice University, 6100 Main St., Houston, 77005, TX, USA
7 Department of Biochemistry and Molecular Biology, Baylor College of Medicine, 1 Baylor Plaza, Houston, 77030, TX, USA

† These authors contributed equally to this work.
∗ Correspondence and requests for materials should be addressed to F.S.-P. (email: stpierre@bcm.edu)
‡Current Address: Department of Biological Engineering, Massachusetts Institute of Technology

Contents

Supplementary Note 1: Model description of four key topologies utilized to predict gene dosage compensation. **2**

Supplementary Note 2: Mathematical modeling of a transcriptional negative feedback circuit **9**

Supplementary Note 3: Estimating the distribution of plasmid copy numbers in transfected cells **10**

Supplementary Note 4: Estimating miRNA affinity **13**

Supplementary Note 5: Predicting cell-to-cell variability **14**

Supplementary Note 6: Comparing intrinsic noise between Equalizer-L and multi-promoter Equalizer-L **15**

Supplementary Note 7: Comparing the coefficient of variation and the Fano factor as measures for quantifying cell-to-cell variability in our experiments. **17**

Supplementary Figures **19**

Supplementary Tables **42**

Supplementary Note 1: Model description of four key topologies utilized to predict gene dosage compensation.

We used deterministic simulations to predict the gene dosage compensation abilities of the five circuit topologies: (1) unregulated (i.e., open-loop constitutive expression) topology, (2) TetR-based negative feedback topology, (3) miRNA-based incoherent feedforward topology, and (4) Equalizer circuit which is a hybrid of negative feedback and incoherent feedforward topologies. (5) Equalizer circuit that separately expresses the circuit components using multiple promoters. Parts of the models were adapted from Nevozhay et al. [1] and Bleris et al. [2]. All reactions are listed below and follow mass action kinetics unless otherwise stated; the reactions that do not follow the mass action kinetics are underlined. The kinetics of these reactions are described by Equations 5-6 at the end of this note.

Topology 1: Unregulated (open-loop) topology

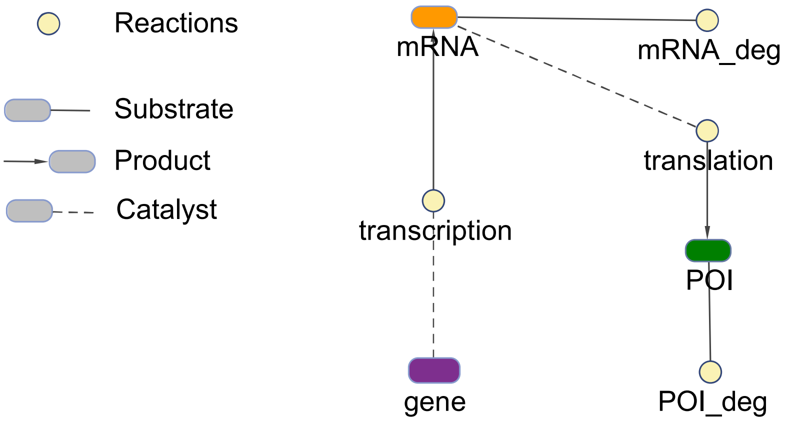

Reactions:

$$\begin{aligned} \text{gene} &\rightarrow \text{mRNA} + \text{gene} \\ \text{mRNA} &\rightarrow \text{POI} \\ \text{POI} &\rightarrow \text{null} \\ \text{mRNA} &\rightarrow \text{null} \end{aligned}$$

Topology 2: Negative feedback (NF) topology implemented with TetR

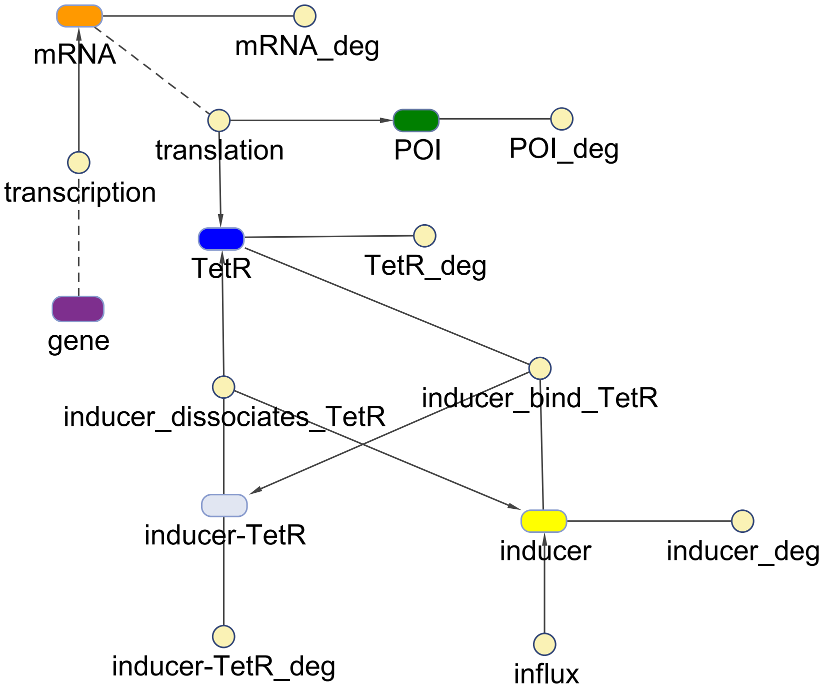

Reactions:

$$
\begin{aligned}
\underline{\text{gene} \rightarrow \text{mRNA} + \text{gene}} \\
\text{mRNA} &\rightarrow \text{POI} + \text{TetR} + \text{mRNA} \\
\text{POI} &\rightarrow \text{null} \\
\text{TetR} + \text{Inducer} &\rightarrow \text{Inducer-TetR} \\
\text{Inducer} &\rightarrow \text{null} \\
\text{null} &\rightarrow \text{Inducer} \\
\text{mRNA} &\rightarrow \text{null} \\
\text{TetR} &\rightarrow \text{null} \\
\text{Inducer-TetR} &\rightarrow \text{null} \\
\text{Inducer-TetR} &\rightarrow \text{TetR} + \text{Inducer}
\end{aligned}
$$

Topology 3: Incoherent feedforward (IFF) topology implemented with a miRNA

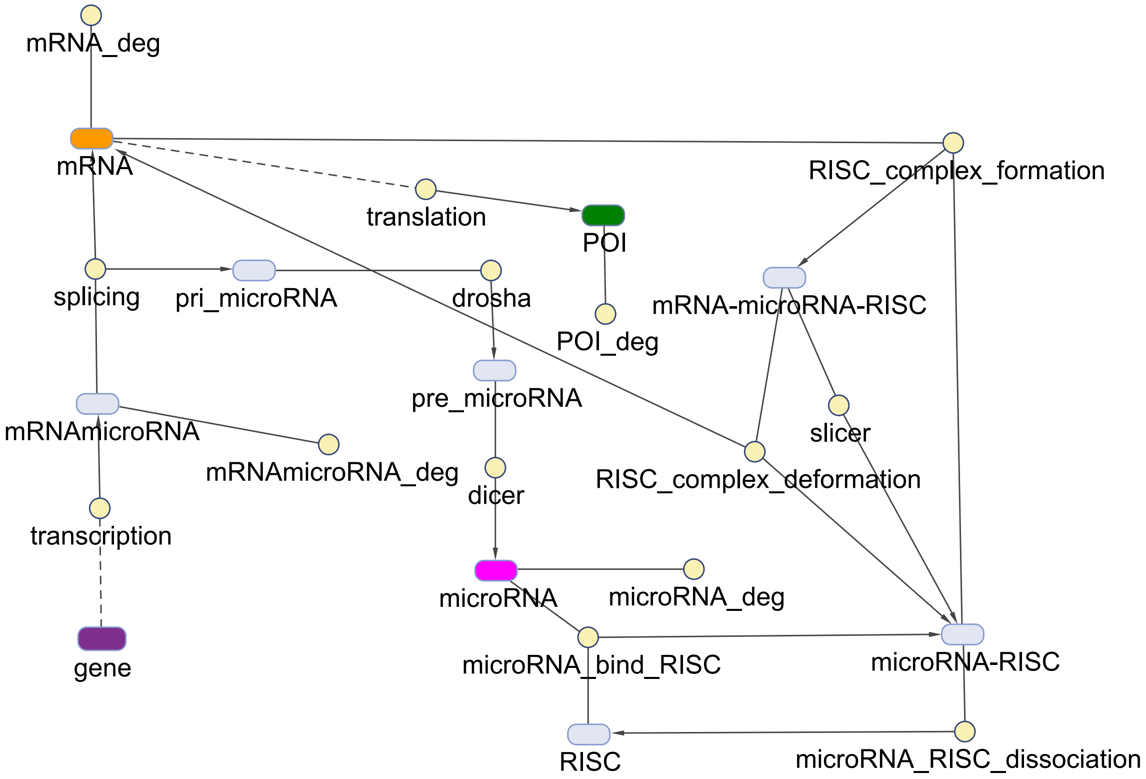

Reactions:

$$\begin{aligned}
\text{gene} &\rightarrow \text{mRNAmicroRNA} + \text{gene} \\
\text{mRNAmicroRNA} &\rightarrow \text{mRNA} + \text{pri_microRNA} \\
\text{mRNA} &\rightarrow \text{POI} + \text{mRNA} \\
\text{pri_microRNA} &\rightarrow \text{pre_microRNA} \\
\text{pre_microRNA} &\rightarrow \text{microRNA} \\
\text{microRNA} + \text{RISC} &\rightarrow \text{microRNA-RISC} \\
\text{microRNA} &\rightarrow \text{null} \\
\text{POI} &\rightarrow \text{null} \\
\text{mRNA} &\rightarrow \text{null} \\
\text{mRNA-microRNA-RISC} &\rightarrow \text{mRNA} + \text{microRNA-RISC} \\
\text{microRNA-RISC} &\rightarrow \text{RISC} \\
\text{mRNA-microRNA-RISC} &\rightarrow \text{microRNA-RISC} \\
\text{mRNAmicroRNA} &\rightarrow \text{null} \\
\text{mRNA} + \text{microRNA-RISC} &\rightarrow \text{mRNA-microRNA-RISC}
\end{aligned}$$

Topology 4: Equalizer topology

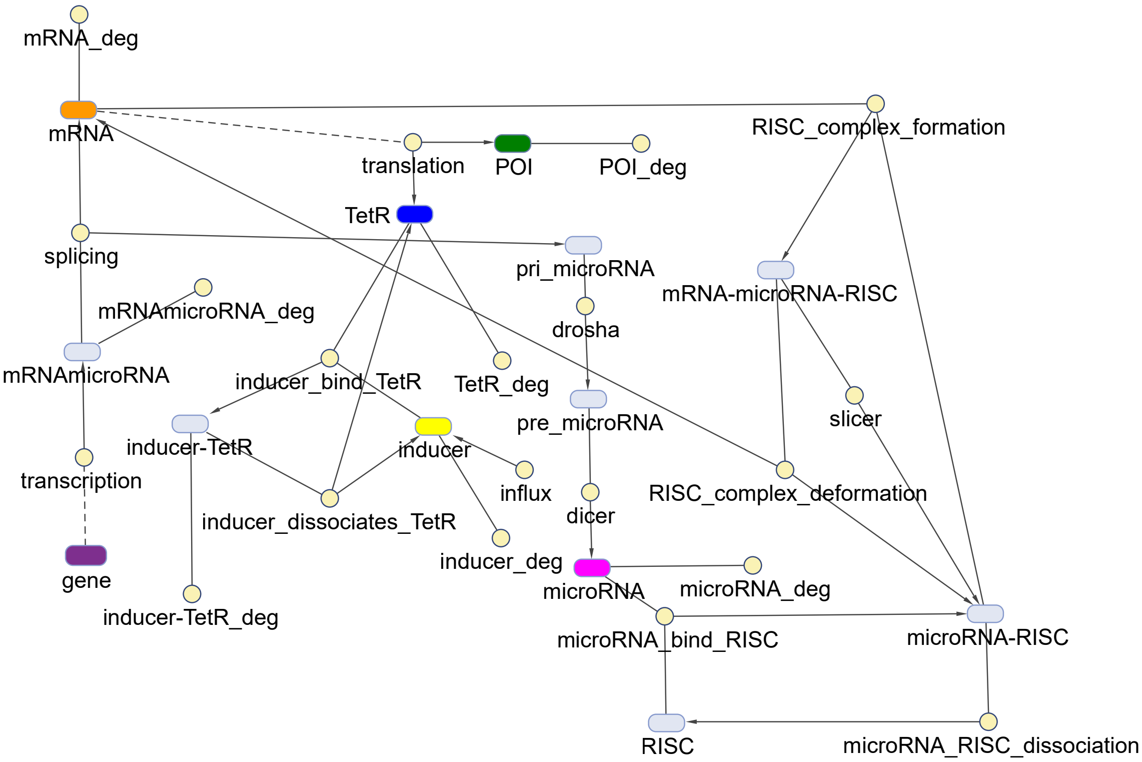

Reactions:

$$\underline{\text{gene} \rightarrow \text{mRNAmicroRNA} + \text{gene}}$$
$$\text{mRNAmicroRNA} \rightarrow \text{mRNA} + \text{pri\_microRNA}$$
$$\text{mRNA} \rightarrow \text{POI} + \text{TetR} + \text{mRNA}$$
$$\text{pri\_microRNA} \rightarrow \text{pre\_microRNA}$$
$$\text{pre\_microRNA} \rightarrow \text{microRNA}$$
$$\text{microRNA} + \text{RISC} \rightarrow \text{microRNA-RISC}$$
$$\text{microRNA} \rightarrow \text{null}$$
$$\text{POI} \rightarrow \text{null}$$
$$\text{TetR} + \text{Inducer} \rightarrow \text{Inducer-TetR}$$
$$\text{Inducer} \rightarrow \text{null}$$
$$\text{null} \rightarrow \text{Inducer}$$
$$\text{mRNA} \rightarrow \text{null}$$
$$\text{mRNA-microRNA-RISC} \rightarrow \text{mRNA} + \text{microRNA-RISC}$$
$$\text{microRNA-RISC} \rightarrow \text{RISC}$$
$$\text{mRNA-microRNA-RISC} \rightarrow \text{microRNA-RISC}$$
$$\text{TetR} \rightarrow \text{null}$$
$$\text{Inducer-TetR} \rightarrow \text{null}$$
$$\text{mRNAmicroRNA} \rightarrow \text{null}$$
$$\text{mRNA} + \text{microRNA-RISC} \rightarrow \text{mRNA-microRNA-RISC}$$
$$\text{Inducer-TetR} \rightarrow \text{TetR} + \text{Inducer}$$

Topology 5: Equalizer circuit implemented with multiple promoters (Multi-promoter Equalizer-L)

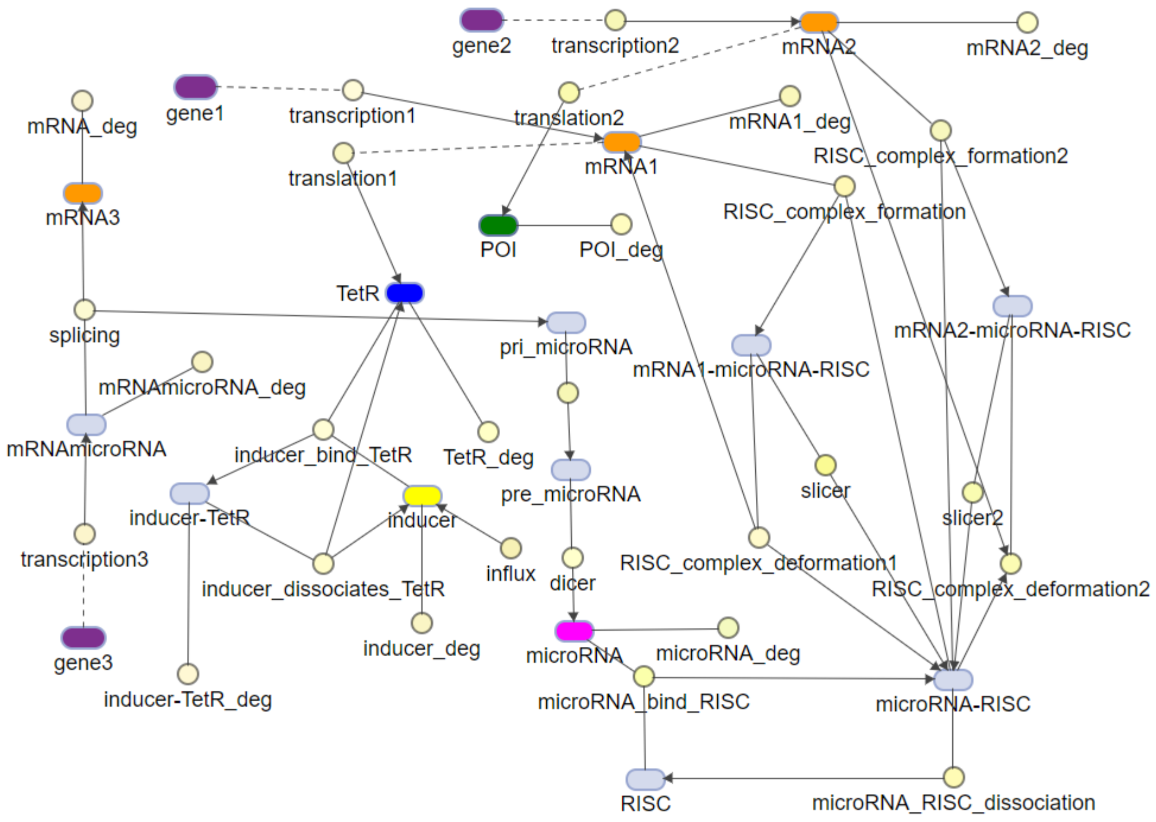

Reactions:

$$\underline{\text{gene1} \rightarrow \text{mRNA1} + \text{gene1}}$$
$$\underline{\text{gene2} \rightarrow \text{mRNA2} + \text{gene2}}$$
$$\underline{\text{gene3} \rightarrow \text{mRNAmicroRNA} + \text{gene3}}$$
$$\begin{aligned}
\text{mRNAmicroRNA} &\rightarrow \text{mRNA3} + \text{pri_microRNA} \\
\text{mRNA1} &\rightarrow \text{TetR} + \text{mRNA1} \\
\text{mRNA2} &\rightarrow \text{POI} + \text{mRNA2} \\
\text{pri_microRNA} &\rightarrow \text{pre_microRNA} \\
\text{pre_microRNA} &\rightarrow \text{microRNA} \\
\text{microRNA} + \text{RISC} &\rightarrow \text{microRNA-RISC} \\
\text{microRNA} &\rightarrow \text{null} \\
\text{POI} &\rightarrow \text{null} \\
\text{TetR} + \text{Inducer} &\rightarrow \text{Inducer-TetR} \\
\text{Inducer} &\rightarrow \text{null} \\
\text{null} &\rightarrow \text{Inducer} \\
\text{mRNA1} &\rightarrow \text{null} \\
\text{mRNA2} &\rightarrow \text{null} \\
\text{mRNA3} &\rightarrow \text{null} \\
\text{mRNA1-microRNA-RISC} &\rightarrow \text{mRNA1} + \text{microRNA-RISC} \\
\text{mRNA2-microRNA-RISC} &\rightarrow \text{mRNA2} + \text{microRNA-RISC} \\
\text{microRNA-RISC} &\rightarrow \text{RISC} \\
\text{mRNA1-microRNA-RISC} &\rightarrow \text{microRNA-RISC} \\
\text{mRNA2-microRNA-RISC} &\rightarrow \text{microRNA-RISC} \\
\text{TetR} &\rightarrow \text{null} \\
\text{Inducer-TetR} &\rightarrow \text{null} \\
\text{mRNAmicroRNA} &\rightarrow \text{null} \\
\text{mRNA1} + \text{microRNA-RISC} &\rightarrow \text{mRNA1-microRNA-RISC} \\
\text{mRNA2} + \text{microRNA-RISC} &\rightarrow \text{mRNA2-microRNA-RISC} \\
\text{Inducer-TetR} &\rightarrow \text{TetR} + \text{Inducer}
\end{aligned}$$

The parameters for mass action kinetics are listed in **Supplementary Table 1**. The transcription reaction of topologies 2, 4, and 5 (as seen in **Supplementary Note 1**) did not follow mass action kinetics, but instead followed the reactions listed below:

$$\mathrm{rate\_of\_reaction} = \mathrm{k\_transcription\_max} \times \mathrm{CN} \times \mathrm{F([TetR])} \tag{1}$$

$$\mathrm{F([TetR])} = (\frac{K_{50}^4}{K_{50}^4 + [\mathrm{TetR}]^4} + \mathrm{leakage})/(1 + \mathrm{leakage})\,. \tag{2}$$

As shown in Equation 2, we used a similar mathematical formulation as Siciliano et al. [3] to describe leakiness of the Tet system. Note that the $\mathrm{leakage}$ parameter cannot be directly calculated by the fold change between mean expression with and without induction, as cells with different plasmid copy number will have different TetR concentrations without induction, and the assumption that $[\mathrm{TetR}]$ is much larger than the half-saturation constant K_{50} may not always hold (so $\frac{K_{50}^4}{K_{50}^4+[\mathrm{TetR}]^4}$ may not be zero when there is no induction for cells with low plasmid copy number). See **Supplementary Note 3** for the estimation of the $\mathrm{leakage}$ parameter.

Supplementary Note 2: Mathematical modeling of a transcriptional negative feedback circuit

We built a mathematical model of an negative feedback (NF) circuit based on repression by a transcription factor called the Tetracycline Repressor (TetR). The two primary states of this circuit are illustrated in the figure below. TetR can bind to its cognate operator sites (*tetO*), thereby inhibiting transcription of its own gene and that of the gene of interest. The two genes are separated by a ribosome-skipping sequence (P2A) that enables multi-cistronic gene expression in eukaryotic systems [4]. In the presence of saturating amounts of the doxycycline inducer (dox), TetR is unable to bind to (*tetO2*) and gene expression can proceed.

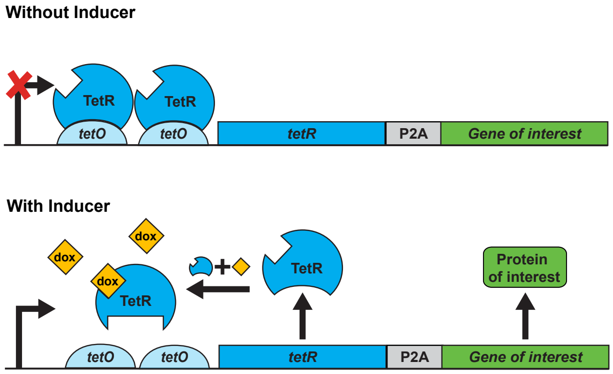

Negative feedback circuit with tetracycline repressor

To model this negative feedback circuit, we modified an existing TetR-based NF model [1] by adding plasmid copy number (CN) as an additional parameter. We used the following set of differential equations based on mass action kinetics:

$$\frac{d[\text{POI}]}{dt} = a \cdot \mathrm{CN} \cdot \mathrm{F}([\text{TetR}]) - d \cdot [\text{P}] \,, \tag{3}$$

$$\frac{d[\text{TetR}]}{dt} = a \cdot \mathrm{CN} \times \mathrm{F}([\text{TetR}]) - b \cdot [\text{TetR}] \cdot [\text{Dox}] - d \cdot [\text{TetR}] \,, \tag{4}$$

$$\frac{d[\text{Dox}]}{dt} = c \cdot [\text{Dox}]_{\mathrm{ext}} - b \cdot [\text{TetR}] \cdot [\text{Dox}] - f \cdot [\text{Dox}] \,, \tag{5}$$

where CN is the plasmid copy number, "[ ]" notation denotes intracellular concentration, and "$[\ ]_{\mathrm{ext}}$" indicates extracellular concentration. Dox is the inducer molecule (i.e. doxycycline). a is the production rate per plasmid. $\mathrm{F}([\mathrm{TetR}])$ is the inhibition function with intracellular TetR concentration as the independent variable. This inhibition function is shown at the end of **Supplementary Note 1**. b is the association rate of TetR and Dox, and d is the protein decay rate, c is the diffusion constant of Dox, and f is the inducer decay rate. The decay terms $d \cdot [\mathrm{TetR}]$ and $f \cdot [\mathrm{Dox}]$ are small compared with the association term $b \cdot [\mathrm{TetR}] \cdot [\mathrm{Dox}]$ [1]. If $d \cdot [\mathrm{TetR}]$ and $f \cdot [\mathrm{Dox}]$ are approximated as zero, the steady-state concentration of POI, $[\text{POI}]_{\mathrm{ss}}$, can be written as:

$$[\text{POI}]_{\mathrm{ss}} = \frac{c \cdot [\text{Dox}]_{\mathrm{ext}}}{d} \,. \tag{6}$$

This shows that at steady-state, the concentration of POI is independent of the copy number, CN. If the approximation above does not hold, $[\text{POI}]_{\mathrm{ss}}$ becomes modestly dependent on plasmid copy number, as shown in the main text (**Fig. 2d**, Ideal Negative Feedback curve).

Supplementary Note 3: Estimating the distribution of plasmid copy numbers in transfected cells

Plasmid copy number distribution in transiently transfected cells was an important parameter used in our model to predict the cell-to-cell variability in gene expression caused by the variability in plasmid copy number. While most of the other parameter values used in our model were determined based on the prior work (see **Supplementary Table 1 and 2**), plasmid copy number distribution is specific to experimental conditions such as transfecting cell type, transfection reagent, amount of transfecting plasmid. Therefore, we estimated this parameter based on our experimental results. The plasmid copy number distribution was estimated using the EGFP expression distribution of HEK293 cells expressing EGFP from the transfected unregulated promoter plasmid and the NF circuit plasmid (see **Methods** for details). For the unregulated promoter plasmids, we observed that expression levels were approximately linearly proportional to the plasmid copy number (**Fig. 3g**). Therefore, the expression level distribution that can be empirically determined could be a proxy for the plasmid copy number distribution. Of note, here, we defined plasmid copy number as "active" plasmid copy number that was actively transcribed. In agreement with previous studies [5], we observed the EGFP expression of cells transfected with the unregulated plasmid roughly followed the gamma distribution whose cumulative distribution function (CDF), $F(x)$, followed:

$$\mathrm{F}(x) = \frac{1}{\Gamma(k)}\gamma(k, \frac{x}{\theta})\,, \tag{7}$$

where x is the effective plasmid copy number, k is the shape parameter, θ is the scale parameter, $\Gamma(k)$ is the gamma function evaluated at k, and $\gamma(k, \frac{x}{\theta})$ is the lower incomplete gamma function. Depicted below is the CDF histogram of EGFP fluorescence of transfected cells that were analyzed by flow cytometry. The red line in the figure below is the fitted CDF curve of a gamma distribution, which has a shape parameter k of 0.57 and a scale parameter θ of 1.7×10^5. Therefore, because the EGFP expression distribution fitted a gamma distribution, we could conclude that the underlying plasmid copy number should also have a gamma distribution, assuming the EGFP expression random variable is the plasmid copy number random variable scaled by some positive constant factor.

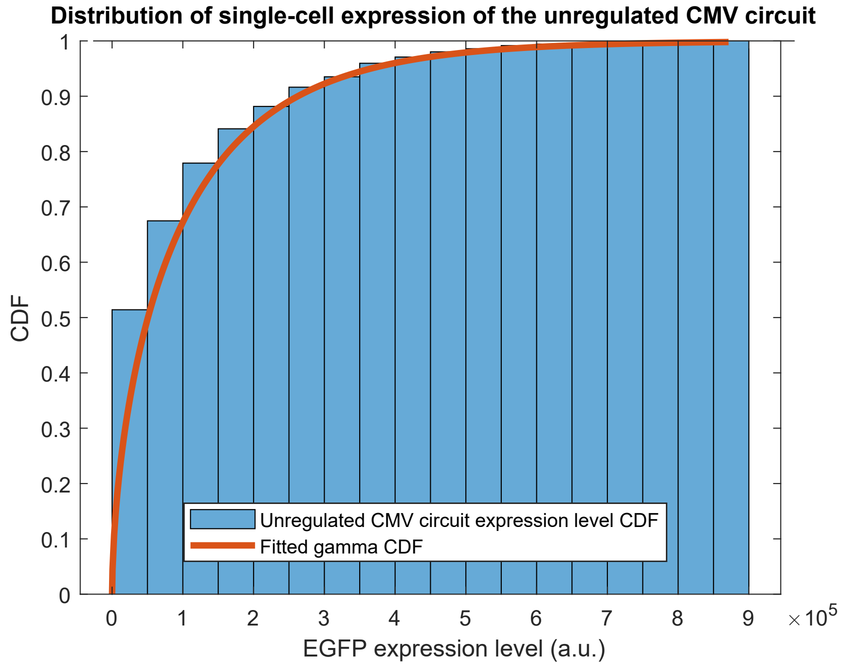

Fitting the shape parameter k of the plasmid copy number distribution

After establishing that the plasmid copy number distribution fits a gamma distribution, we first set out to determine the shape parameter k of the plasmid copy number distribution. To this end, we used the flow-cytometry-acquired single-cell EGFP expression distributions of HEK293 cells transfected with the unregulated CMV plasmid.

As a property of the gamma distribution, the shape parameter k remains constant if the random variable is scaled by some positive constant. Thus we can use any unregulated plasmid to estimate the shape parameter k of the plasmid copy number distribution. Single-cell unregulated CMV plasmid data (depicted in the first figure) were used

to estimate the shape parameter k of the plasmid copy number distribution to be 0.57 (see MATLAB script in the Equalizer GitHub repository: *SuppNote3_CopyNumberDistShapeParameterEstimate.m*)

Further validation confirmed that expression levels scale linearly with plasmid copy numbers. This was accomplished by fitting the shape parameter k using the single-cell unregulated PGK plasmid data. Due to the lower expression of EGFP from the PGK promoter than CMV promoter, this promoter was chosen to demonstrate that absolute expression levels would not impact fitting of k. Using the same shape fitting technique as was done for the unregulated CMV circuit, the unregulated PGK circuit had a similar fit with an estimated k value of 0.56. These data further support the assumption that expression levels scale linearly with plasmid copy number and suggests only minimal non-linearity.

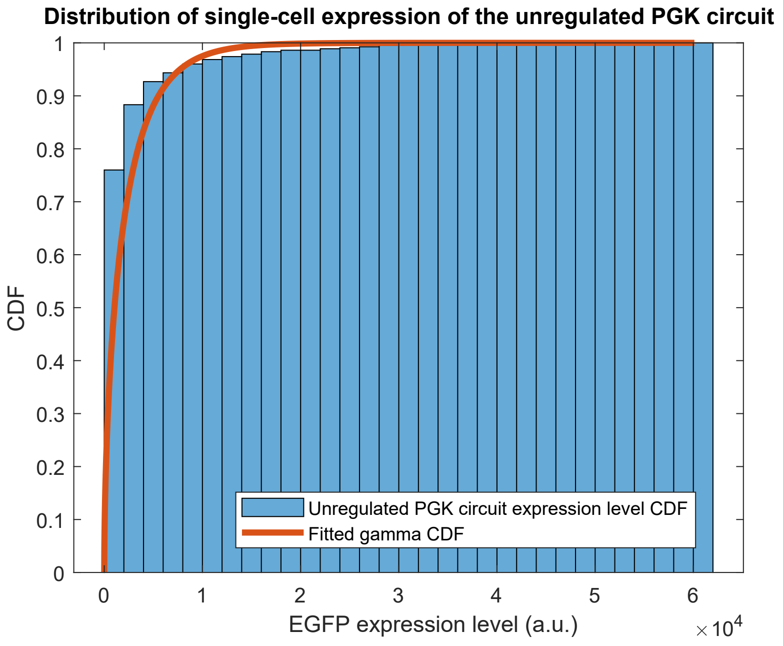

Fitting the scale parameter θ of the plasmid copy number distribution and the leakage of the NF circuit

Having determined the shape parameter k, we can now use the experimentally determined mean expression of the NF circuit at different doxycycline concentrations to estimate the scale parameter θ of the plasmid copy number distribution and the $\mathrm{leakage}$ parameter. In contrast to determining k, the distribution of single-cell expression from unregulated circuits does not constrain θ. The additional constraints imposed by the mean expression of the NF circuit at different doxycycline concentrations allowed for estimation of θ and $\mathrm{leakage}$ at the same time, since the plasmid copy number distribution and the leakage parameter both affect the expression induction curve of the NF circuit. We scanned across different leakage and scale parameter θ combinations to minimize the mean squared error (MSE) of the simulated mean expression of the NF circuit at 8 different inducer concentrations (0, 1, 5, 10, 50, 100, 500, 1000 ng/mL) compared with experimental data. All experimental data were normalized to the lowest expression level so that the simulated expression vs. inducer curve demonstrated a similar trend to what is experimentally observed. As shown in the plot below, where the z-axis shows the negative MSE, there is a sharp gradient along the leakage axis. The smallest deviation between the simulated and experimental data (i.e., MSE closest to zero) is achieved at the leakage value of 0.25.

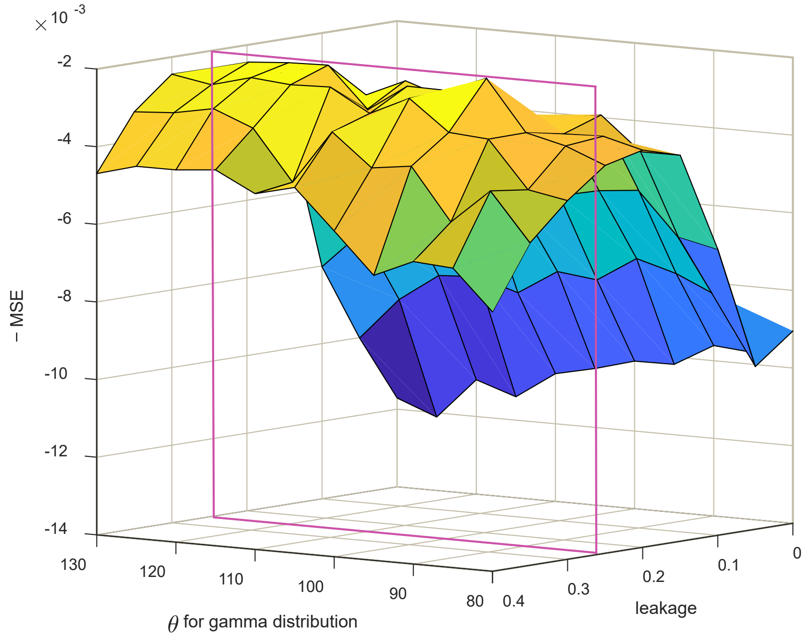

See: SuppNote3_CopyNumberDistScaleParameter_Leakage_Estimate.m.

Given the small gradient along the scale parameter θ axis, we did a finer scan of the scale parameter with leakage fixed at 0.25 and with a larger range of θ from 60 to 200. This is equivalent to taking a slice along $\mathrm{leakage}$ = 0.25 and looking at MSE versus θ, as indicated by the pink bounding box in the figure above. The MSE is closest to zero between a scale parameter θ of 100 to 120. θ of 120 is used for the simulations in this work. See **Supplementary Note 5** for how sensitive the simulation results are to the choice of θ.

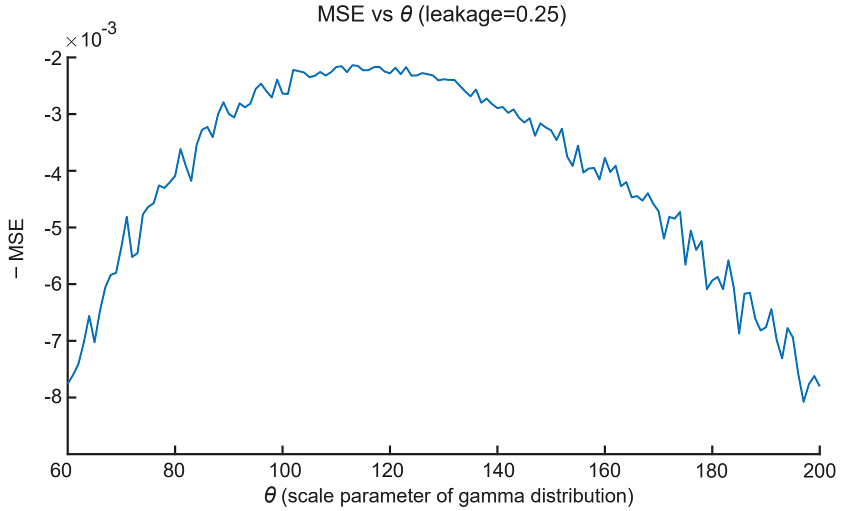

See: SuppNote3_CopyNumberDistScaleParameter_1dScan.m.

Using the defined shape and scale parameters, the plasmid copy number distribution can be used to predict the cell-to-cell variability caused by plasmid copy number variability for any given circuit model.

Supplementary Note 4: Estimating miRNA affinity

miRNA is an integral part of several of the circuits used in this study, thus to properly model and predict protein of interest cell-to-cell variation for these circuits, miRNA affinity was estimated. miRNA affinity to its target depends on the specific sequences of the miRNA and target, the number of target sites on the transcript, and location of target sites on the transcript [6]. From the modeling standpoint, miRNA affinity can be described by the ratio of the dissociation rate constant and the association rate constant. Previous studies found that miRNA affinities are modulated by the dissociation rate constant [7,8]. Thus, we fixed the association rate constant ($\mathrm{kf\_RISC\_complex\_formation}$ in the model) and used the change in dissociation rate constant ($\mathrm{kf\_RISC\_complex\_deformation}$ in the model) to model the change in miRNA affinity to its target. MATLAB's fminsearch function was used to fit the values of dissociation rate constant by minimizing the mean squared difference between the simulated and experimental ratio of the mean expression of 10 thousand cells expressing the Equalizer circuit and the NF circuit at 6 different inducer concentrations (0, 1, 5, 10, 50, 100 ng/mL). Equalizer and the NF circuits were included in this analysis so that direct comparisons between a circuit with and without miRNA, respectively, can be done. The plasmid copy number distribution was estimated from the distribution obtained from Supplementary Note 3. To test the simulation accuracy, different initial guesses of the dissociation rate constant of miRNA to its target were used and converged to the same estimated dissociation rate constant of 0.303 second^{-1}. See: SuppNote4_microRNAaffinity_Estimate.m.

Having now defined the parameters for miRNA affinity and plasmid copy number distribution, the fully specified models for Equalizer and the IFF circuit can be used to predict cell-to-cell variability of cells expressing the circuits.

Supplementary Note 5: Predicting cell-to-cell variability

Our modeling can predict the cell-to-cell variability originating from plasmid copy number variability (extrinsic noise). However, the experimentally observed cell-to-cell variability is caused by intrinsic noises and extrinsic noise. To compare our prediction with experimental data, we made the following assumptions: (1) plasmid copy number variability is the major source of extrinsic noise, and extrinsic noise from other sources is negligible in comparison; (2) intrinsic noise is constant across Equalizer, IFF circuit, NF circuit, and unregulated CMV circuit. Based on the second assumption, the intrinsic noise can then be approximated as the experimentally observed cell-to-cell variability of the CMV cell line, which integrated the unregulated CMV circuit to the genome and thus, the noise should be mostly intrinsic noise since it has minimal DNA copy number variability. To predict the total noise (coefficient of variation), we utilized the relationship that the square of total noise equals the sum of squares of extrinsic noise and intrinsic noise [9], i.e., $\mathrm{CV}^2_{\mathrm{total}} = \mathrm{CV}^2_{\mathrm{intrinsic}} + \mathrm{CV}^2_{\mathrm{extrinsic}}$.

Given the relatively large range of the scale parameter (θ) to produce a low MSE (θ = 100 to 120, **Supplementary Note 3**) and the effect θ has on estimated miRNA affinity, we tested how different θ values would affect the predicted cell-to-cell variability. Shown below is the predicted cell-to-cell variability by simulating 10 thousand cells expressing the Equalizer-L and NF circuits versus the experimentally observed cell-to-cell variability induced with different concentrations of doxycycline (also shown in **Fig. 3d**). Comparing the simulated to the experimental values demonstrates that the simulations could predict the relative shapes for each circuit. Additionally, the predicted CV is largely unaffected by the change of θ from 120 to 100, as seen by only small changes in the simulated curves between the two different θ values. These data suggest that using a θ of 120 and a miRNA dissociation rate constant of 0.303 second^{-1} in our simulations produces accurate predictions and thus, we used these values in all other modeling done in this work unless otherwise specified.

a

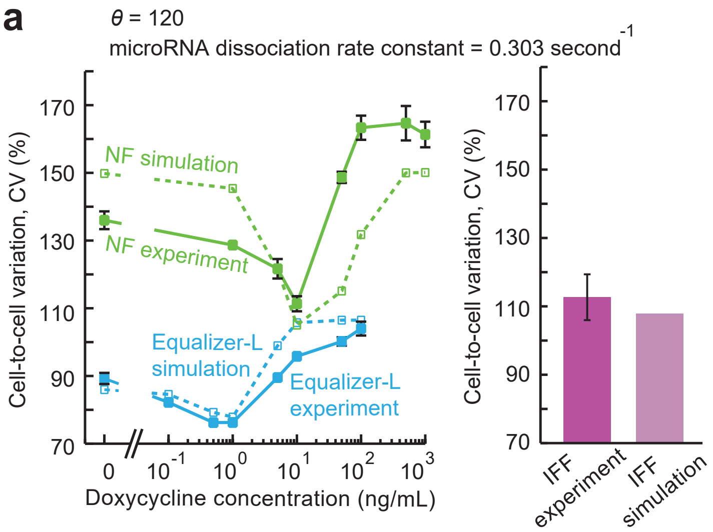

b

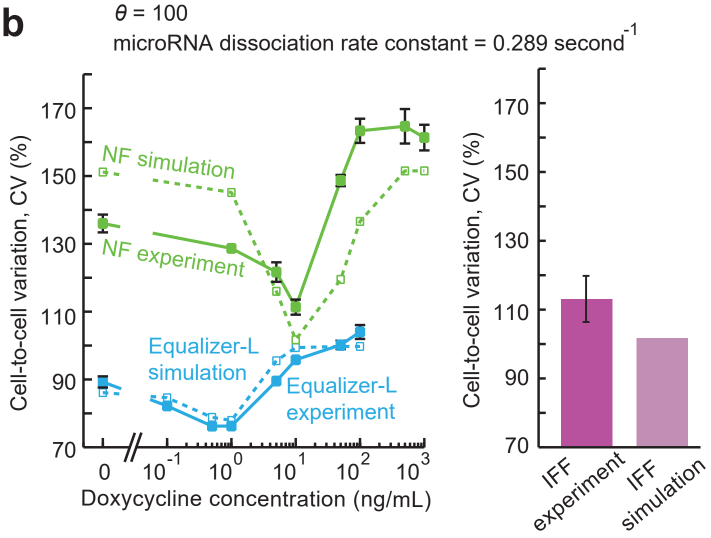

Supplementary Note 6: Comparing intrinsic noise between Equalizer-L and multi-promoter Equalizer-L

To evaluate the impact of having multiple promoters on the intrinsic noise of expression from Equalizer, we performed stochastic simulations to compute the intrinsic noise across different transcriptional bursting parameter space. To model transcriptional bursting, we added an off-state to the gene of interest to the deterministic models, which is a standard assumption in the field that has been supported by in vivo observation [10]. To minimize redundancy, here we only show the additional reactions on top of the reactions listed in **Supplementary Note 1**:

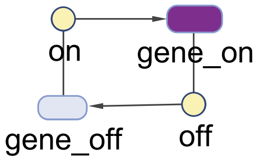

The switching between on-state and off-state follows mass action kinetics parameters $\mathrm{k\_on}$ and $\mathrm{k\_off}$ (as shown in **Supplementary Table 1**).
Here we focus on the comparison between Equalizer and the multi-promoter version of Equalizer (**Supplementary Fig. 15a**), which has the same regulatory elements as the Equalizer circuit but expresses each regulatory element from separate promoters as opposed to a single promoter in the Equalizer circuit:

Stochastic simulation setup

We performed stochastic simulations using Stochkit2 [11], stand-alone stochastic simulation software that allows defining custom propensity functions. For each topology, a first simulation is run for 10^6 seconds to reach a steady-state. Then, auto-correlation is computed for the Equalizer circuit (topology 4) to determine the time τ required for the system to "forget" its initial state at time 0 as shown below. τ of $5\times\ 10^6$ seconds was chosen since auto-correlation is well within the approximate upper and lower auto-correlation confidence bounds shown as blue horizontal lines.

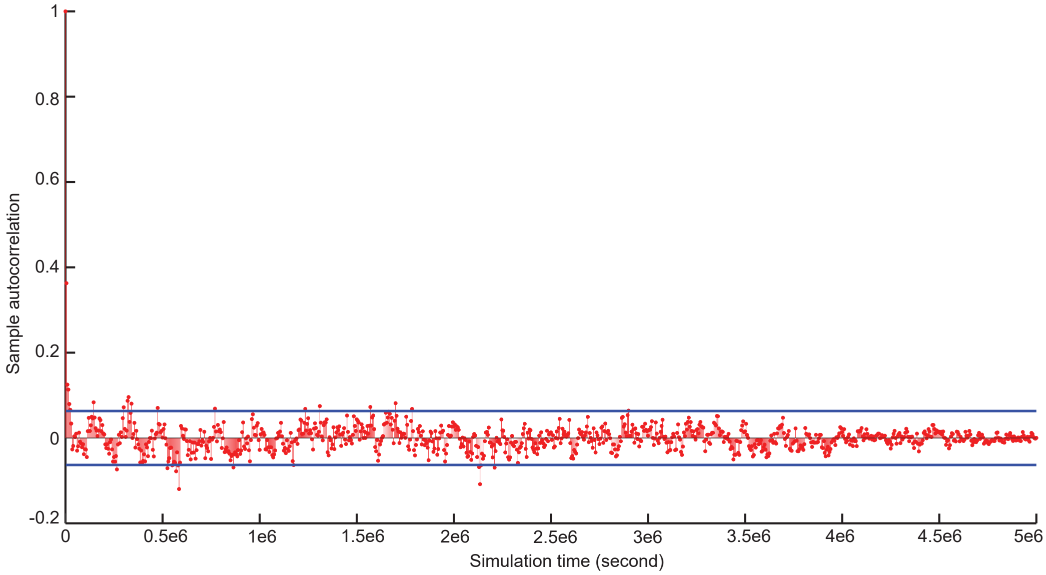

Then, using the end states of the first 10^6-second simulation for each topology as the initial states, 1000 simulations of length τ were run. The mean and standard deviation of the POI molecule numbers of the 1000 simulations are used to calculate the simulated cell-to-cell variability. The initial condition for all topologies assumes 10 copies of the gene in the off-state, the inducer at a steady-state determined by the influx rate and the degradation rate shown in Supplementary Table 1 with an extracellular inducer of 0.5 ng/mL, and RISC molecule count is set to the value mentioned above (1.7e5 molecules/cell); the initial molecule count for the rest of the species is zero.
In the first set of stochastic simulations, we used the kinetic parameters listed in **Supplementary Table 1** for the Equalizer circuit (Topology 4 in **Supplementary Note 1**). We modified the kinetic parameters for the multiple pro-

moter Equalizer circuit (Topology 5 in **Supplementary Note 1**) so that the steady-state mRNA amount and steady-state POI amount remains the same as the Equalizer circuit. The following table shows the modified parameters to achieve the same steady-state amount of mRNA and POI:

Stochastic simulation set 1: original parameters

Parameter	Equalizer	Multiple promoter Equalizer
k_transcription (1/second)	4.67e-2	3.70e-2 (same for all three genes)
k_translation (1/second)	3.33e-4	3.33e-4 (same for all three genes)
k_on (1/second)	3.10e-4	3.10e-4 (same for all three genes)
k_off (1/second)	4.18e-4	4.18e-4 (same for all three genes)

The second set of stochastic simulations test how each topology reacts to amplified translation bursting, for which the steady-state mRNA amount in each topology is reduced 50 fold compared with set 1 while maintaining steady-state POI and TetR amount with the following modified parameters:

Stochastic simulation set 2: amplified translational bursting

Parameter	Equalizer	Multiple promoter Equalizer
k_transcription (1/second)	5.10e-4	4.40e-4 (same for all three genes)
k_translation (1/second)	1.66e-2	1.66e-2 (same for all three genes)
k_on (1/second)	3.10e-4	3.10e-4 (same for all three genes)
k_off (1/second)	4.18e-4	4.18e-4 (same for all three genes)

The third set of stochastic simulations test how each topology reacts to amplified transcriptional bursting, for which the transcription bursting parameters k_on and k_off are decreased by 10 fold compared with set 1:

Stochastic simulation set 3: amplified transcriptional bursting

Parameter	Equalizer	Multiple promoter Equalizer
k_transcription (1/second)	4.67e-2	3.70e-2 (same for all three genes)
k_translation (1/second)	3.33e-4	3.33e-4 (same for all three genes)
k_on (1/second)	3.10e-5	3.10e-5 (same for all three genes)
k_off (1/second)	4.18e-5	4.18e-5 (same for all three genes)

Stochastic simulations results are shown in **Supplementary Fig. 15**.

Supplementary Note 7: Comparing the coefficient of variation and the Fano factor as measures for quantifying cell-to-cell variability in our experiments.

Heterogeneity of a population not only arises between cells of different genetic backgrounds but also occurs in populations that arise from genetically identical cells through differences in intrinsic and extrinsic noise [12–15]. There are two main measures to describe population variation, coefficient of variation (CV) [16] and Fano factor [17]. Consider a population distribution with a mean (μ) and a standard deviation (σ) defined as:

$$\mu = \frac{1}{N}\sum_{i=1}^{N} x_i \,, \tag{8}$$

$$\sigma = \sqrt{\frac{\sum_{i=1}^{N}(x_i-\mu)^2}{N}} \,, \tag{9}$$

where N is the size of the population and x_i is each value of the population.
Then CV is defined as:

$$\mathrm{CV} = \frac{\sigma}{\mu} \,, \tag{10}$$

and Fano factor is defined as:

$$F = \frac{\sigma^2}{\mu} \,. \tag{11}$$

CV is a dimensionless quantity that is invariant to proportional scaling. When the population distribution x is scaled with a factor α, then the mean of the scaled distribution, μ_{scaled} is:

$$\mu_{\mathrm{scaled}} = \frac{1}{N}\sum_{i=1}^{N} \alpha x_i \,, \tag{12}$$

$$= \alpha\mu \,, \tag{13}$$

and the standard deviation of the scaled distribution σ_{scaled}, is:

$$\sigma_{\mathrm{scaled}} = \sqrt{\frac{\sum_{i=1}^{N}(\alpha x_i-\alpha\mu)^2}{N}} \,, \tag{14}$$

$$= \sqrt{\frac{\sum_{i=1}^{N}\alpha^2(x_i-\mu)^2}{N}} \,, \tag{15}$$

$$= \alpha\sqrt{\frac{\sum_{i=1}^{N}(x_i-\mu)^2}{N}} \,, \tag{16}$$

$$= \alpha\sigma \,. \tag{17}$$

The CV of the scaled distribution, $\mathrm{CV}_{\mathrm{scaled}}$ is:

$$\mathrm{CV}_{\mathrm{scaled}} = \frac{\sigma_{\mathrm{scaled}}}{\mu_{\mathrm{scaled}}} \,, \tag{18}$$

$$= \frac{\alpha\sigma}{\alpha\mu} \,, \tag{19}$$

$$= \frac{\sigma}{\mu} \,, \tag{20}$$

$$= \mathrm{CV} \,. \tag{21}$$

Unlike CV, the Fano factor changes when the population distribution is scaled with a factor α:

$$F_{\text{scaled}} = \frac{\sigma^2_{\text{scaled}}}{\mu_{\text{scaled}}}\,, \tag{22}$$

$$= \frac{(\alpha\sigma)^2}{\alpha\mu}\,, \tag{23}$$

$$= \frac{\alpha^2\sigma^2}{\alpha\mu}\,, \tag{24}$$

$$= \frac{\alpha\sigma^2}{\mu}\,, \tag{25}$$

$$= \alpha F\,. \tag{26}$$

The experiments conducted in this work produce quantities in arbitrary units where the signal is proportional to the molecule number with unknown proportionality. In such cases, the Fano factor has major disadvantages. Not only would the application of the Fano factor metric have unclear units, but its exact value would scale with the changes in the unknown proportionality coefficient as Eq. (26). As a result, the Fano factor does not stay invariant under proportional scaling, unlike CV. For example, the Fano factor cannot be compared between experiments that employ measurements of molecule count (e.g., flow cytometry, microscopy) with different devices, cameras, or amplification gain settings as these are expected to affect the proportionality coefficient. Additionally, the Fano factor compares the spread of probability distribution relative to a Poisson distribution with the same mean. Thus, the Fano factor metric struggles to deal with comparisons between conditions when the mean value of each condition is very different (**Supplementary Fig. 14i**). For example, if two conditions have the same mean, but one has a greater Fano factor value, then there is a clear difference in dispersion. However, if the conditions have different means, it is difficult to interpret what the difference in their Fano Factor values means in terms of fold change since there is no proportional scaling.

The proportional scaling of CV allows for comparing the variability between systems with different sized means and units. Additionally, differences in data acquisition can be tolerated as long as the measurement values differ by a scaling factor. CV can also be expressed in percentage (as we chose to do here) by multiplying the value given by Eq. (10) by 100. For these reasons, CV was chosen to quantify cell-to-cell variability.

Supplementary Figures

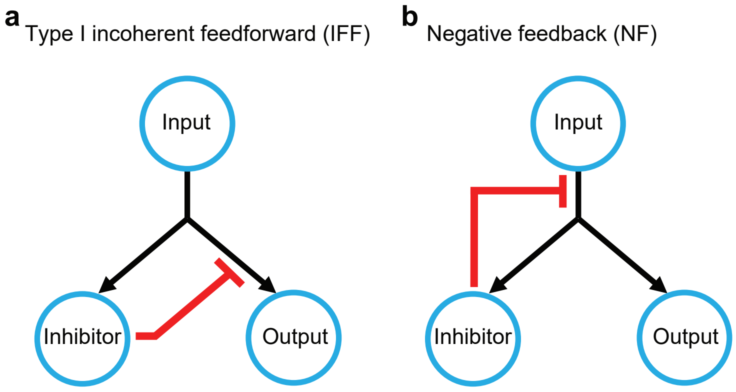

Supplementary Fig. 1 | Negative feedback and type I incoherent feedforward topologies of genetic circuits. **(a)** In type I incoherent feedforward circuits, a common input (e.g., plasmid) drives the expression (black arrows) of an inhibitor (e.g., miRNA) and an output protein (e.g., EGFP). The inhibitor only represses (red blunted arrow) the output protein production and does not repress its own expression. **(b)** In negative feedback circuits, a common input (e.g., plasmid) drives the expression of an inhibitor (e.g., Tet repressor protein) and an output protein (e.g., EGFP). Unlike incoherent feedforward circuits, the inhibitor represses both its own production and that of the output protein.

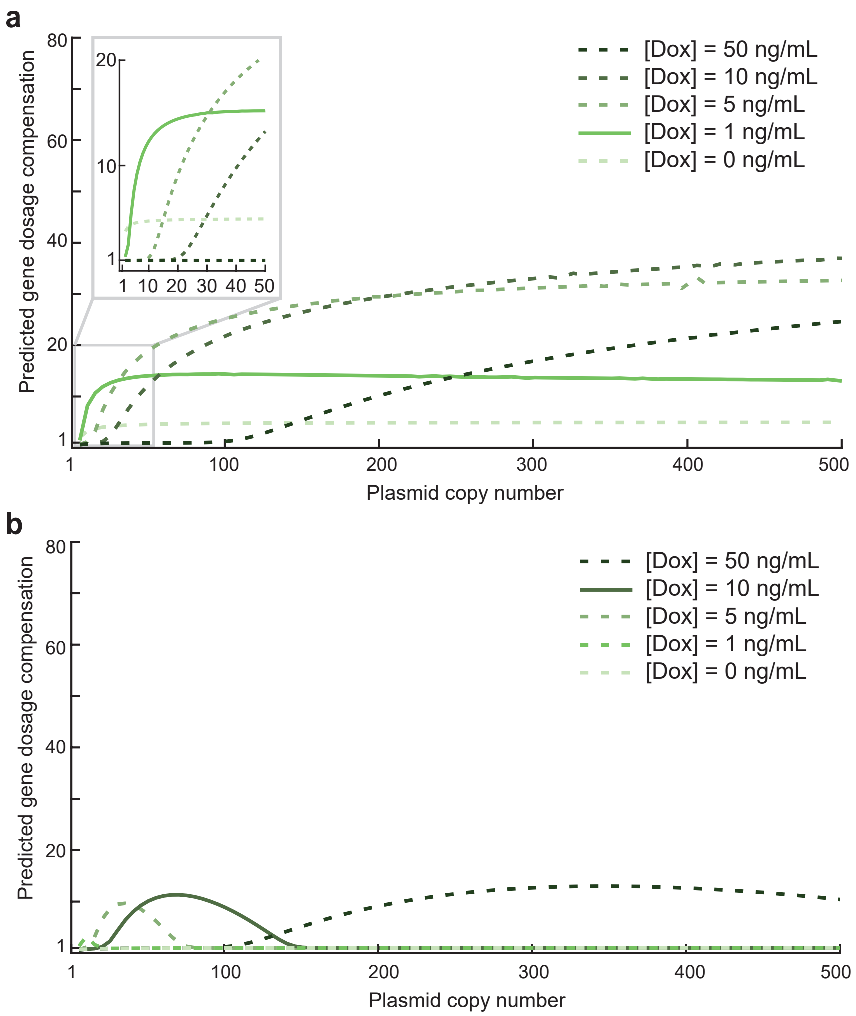

Supplementary Fig. 2 | The gene dosage compensation profiles of NF circuits can be tuned by changing the doxycycline concentration. Predicted gene dosage compensation at different doxycycline concentrations for (**a**) an ideal NF circuit or (**b**) an NF circuit that is ”leaky”. The leaky NF circuit has incomplete repression at saturating concentrations of TetR. The solid lines correspond to the doxycycline concentrations that predicted the lowest CV with the fitted plasmid copy number distribution described in **Supplementary Note 3**, 1ng/mL for the ideal NF and 10ng/mL for leaky NF. The calculation of the predicted gene dosage compensation score is described in the **Methods**. See **Supplementary Note 1** for the model description and **Supplementary Tables 1 and 2** for other parameters used in the simulations.

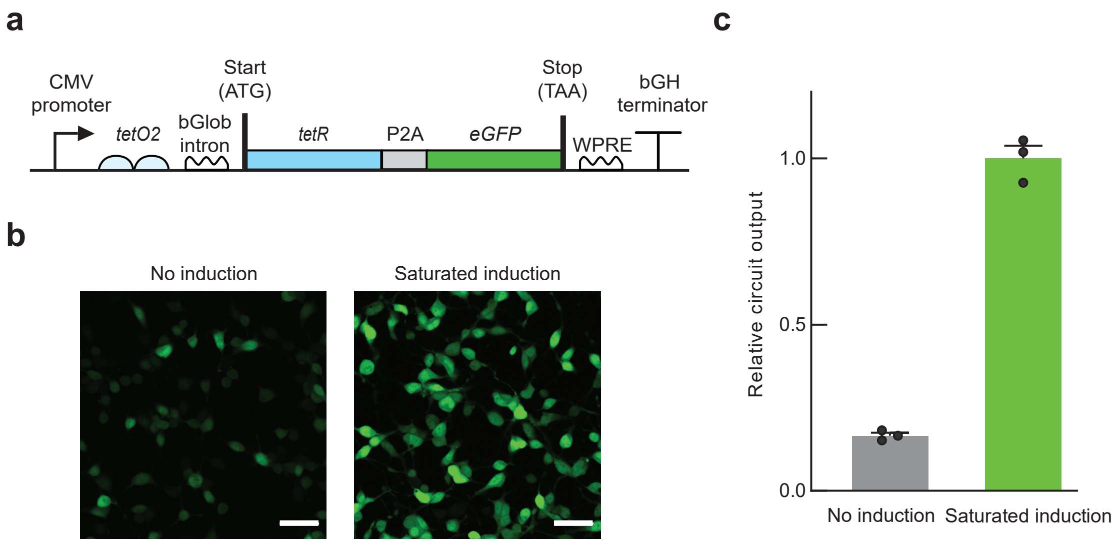

Supplementary Fig. 3 | A TetR-based negative feedback circuit shows incomplete repression when uninduced. (**a**) Schematic of an EGFP expression cassette with negative feedback (NF) control. (**b-c**) Uninduced NF circuits produce substantial fluorescence. **(b)** Representative images of HEK293A cells transfected with plasmids encoding the NF circuit without inducer (left) and under saturating amounts of inducer (1000 ng/mL doxycycline, right). Images are shown with the same lookup table. Scale bars, 50 μm. (**c**) The mean circuit output levels of the uninduced NF is 17 $\pm$ 1.1 % of the circuit output levels obtained under fully induced NF. The mean output levels were relative to that of the fully induced NF circuit. Error bars indicate SEM. The black circles represent n = 3 independent transfections.

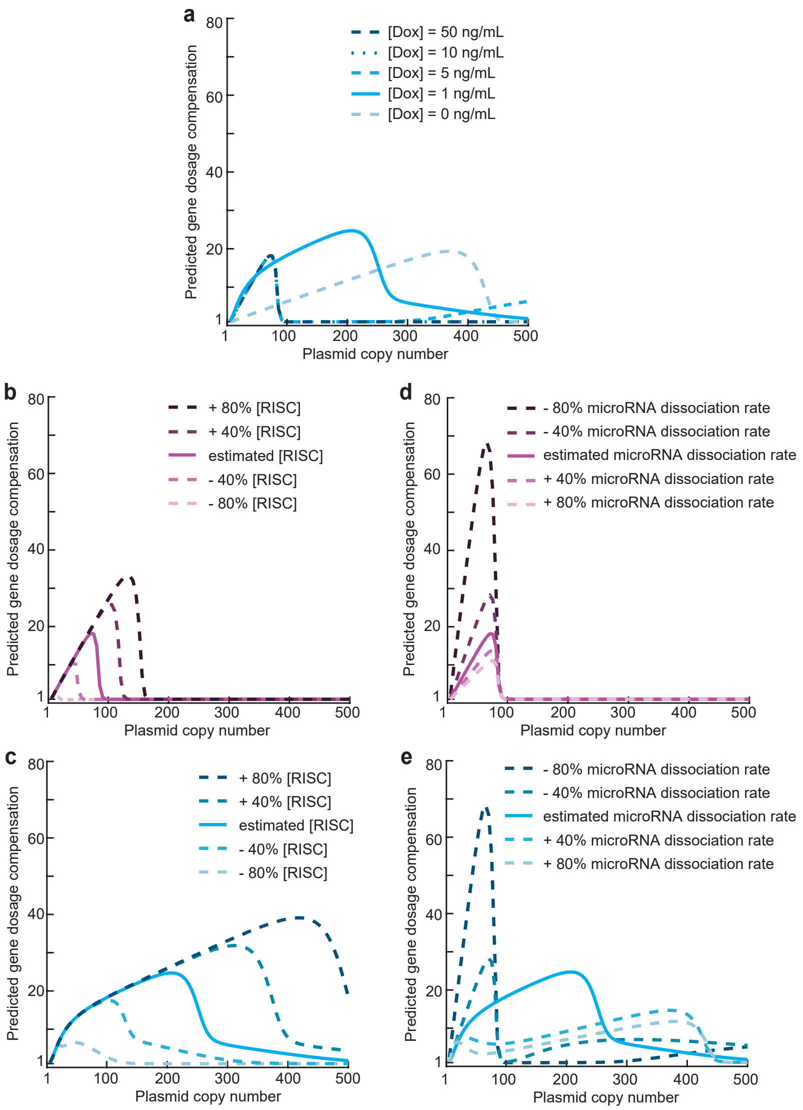

Supplementary Fig. 4 | **Simulating the gene dosage compensation profiles of Equalizer and IFF circuits using different model parameters.** (**a**) The gene dosage compensation profile of the Equalizer can be tuned by changing the doxycycline induction level. The curves for doxycycline concentrations of 5, 10, and 50 ng/mL show substantial overlap. The mathematical definition of predicted gene dosage compensation is described in **Methods**. (**b-e**) The Equalizer circuit is predicted to display improved gene dosage compensation compared with its incoherent feedforward (IFF) subcircuit over a wide range of RISC concentrations and miRNA-target binding affinities. (**b & c**) Predicted dosage compensation as a function of plasmid copy number for (**b**) the IFF subcircuit and (**c**) the Equalizer. For both panels, RISC abundance was varied 9-fold (from 0.2 to 1.8-fold). The solid lines corresponds to 1.7e+05 RISC complexes/cell. The simulated doxycycline concentration was 1 ng/mL. (**d & e**) Predicted dosage compensation as a function of plasmid copy number for (**d**) the IFF subcircuit and (**e**) the Equalizer. For both panels, the miRNA affinity was tuned in the model by changing the miRNA dissociation rate constant while keeping the miRNA association rate constant unchanged. For this *in silico* proof of concept, before any parameter fitting, the miRNA dissociation rate was first set to 0.3 second^{-1} (solid line) and was varied 9-fold (from 0.2 to 1.8-fold, dashed lines). The simulated doxycycline concentration was 1 ng/mL. See **Supplementary Note 1** for model description and **Supplementary Tables 1 and 2** for other parameters used in the simulations.

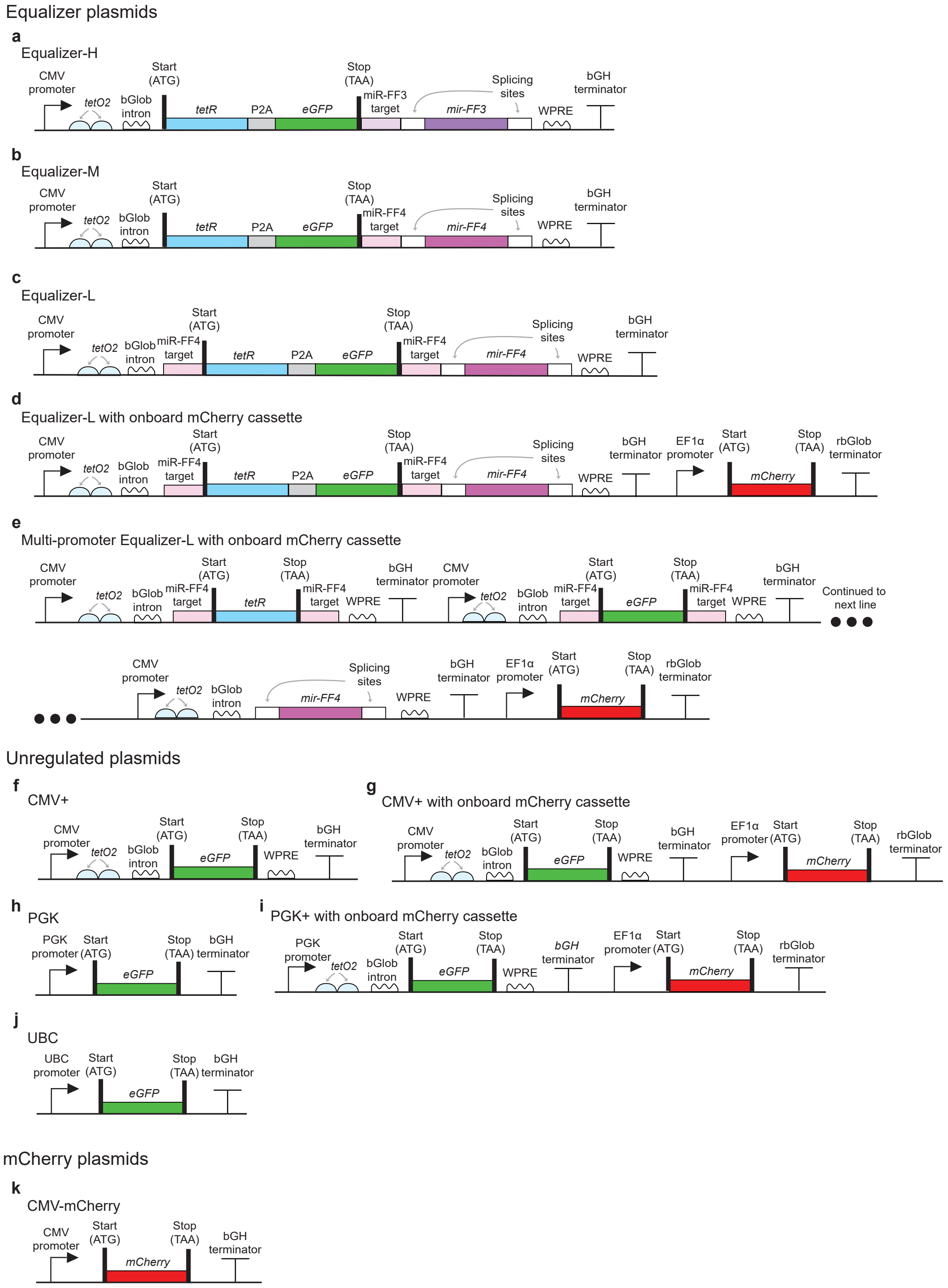
Equalizer plasmids
a
Equalizer-H
CMV promoter
tetO2
bGlob intron
Start (ATG)
tetR
P2A
eGFP
Stop (TAA)
miR-FF3 target
Splicing sites
mir-FF3
WPRE
bGH terminator
b
Equalizer-M
CMV promoter
tetO2
bGlob intron
Start (ATG)
tetR
P2A
eGFP
Stop (TAA)
miR-FF4 target
Splicing sites
mir-FF4
WPRE
bGH terminator
c
Equalizer-L
CMV promoter
tetO2
bGlob intron
miR-FF4 target
Start (ATG)
tetR
P2A
eGFP
Stop (TAA)
miR-FF4 target
Splicing sites
mir-FF4
WPRE
bGH terminator
d
Equalizer-L with onboard mCherry cassette
CMV promoter
tetO2
bGlob intron
miR-FF4 target
Start (ATG)
tetR
P2A
eGFP
Stop (TAA)
miR-FF4 target
Splicing sites
mir-FF4
WPRE
bGH terminator
EF1α promoter
Start (ATG)
mCherry
Stop (TAA)
rbGlob terminator
e
Multi-promoter Equalizer-L with onboard mCherry cassette
CMV promoter
tetO2
bGlob intron
miR-FF4 target
Start (ATG)
tetR
Stop (TAA)
miR-FF4 target
WPRE
bGH terminator
CMV promoter
tetO2
bGlob intron
miR-FF4 target
Start (ATG)
eGFP
Stop (TAA)
miR-FF4 target
WPRE
bGH terminator
Continued to next line
CMV promoter
tetO2
bGlob intron
Splicing sites
mir-FF4
WPRE
bGH terminator
EF1α promoter
Start (ATG)
mCherry
Stop (TAA)
rbGlob terminator
Unregulated plasmids
f
CMV+
CMV promoter
tetO2
bGlob intron
Start (ATG)
eGFP
Stop (TAA)
WPRE
bGH terminator
g
CMV+ with onboard mCherry cassette
CMV promoter
tetO2
bGlob intron
Start (ATG)
eGFP
Stop (TAA)
WPRE
bGH terminator
EF1α promoter
Start (ATG)
mCherry
Stop (TAA)
rbGlob terminator
h
PGK
PGK promoter
Start (ATG)
eGFP
Stop (TAA)
bGH terminator
i
PGK+ with onboard mCherry cassette
PGK promoter
tetO2
bGlob intron
Start (ATG)
eGFP
Stop (TAA)
WPRE
bGH terminator
EF1α promoter
Start (ATG)
mCherry
Stop (TAA)
rbGlob terminator
j
UBC
UBC promoter
Start (ATG)
eGFP
Stop (TAA)
bGH terminator
mCherry plasmids
k
CMV-mCherry
CMV promoter
Start (ATG)
mCherry
Stop (TAA)
bGH terminator

Supplementary Fig. 5 | **Schematics of Equalizer and unregulated expression plasmids**. (**a-e**) Schematics of Equalizer plasmids. bGlob intron is the rabbit β-*globin* intron II. WPRE is the woodchuck hepatitis virus post-transcriptional regulatory element. bGlob intron and WPRE were placed at the 5' and 3' UTRs, respectively, to increase the overall gene expression levels. (**f-j**) Schematics of unregulated expression plasmids. For the CMV and PGK unregulated promoters, we made additional variants with *tetO2*, bGlob intron, and WPRE for closer similarity to the Equalizer circuits. These elements did not impact cell-to-cell variability (**Supplementary Fig. 21**). For simplicity, those variants are also referred to as CMV and PGK, as appropriate. **Supplementary Table 4** lists the experiments in which each plasmid was used. (**k**) Schematic of unregulated mCherry expression plasmid. The CMV-mCherry plasmid was co-transfected with the circuit plasmids that do not have the onboard mCherry cassette. This plasmid was used to quantify plasmid uptake and distinguish transfected cells from the non-transfected cells. See **Supplementary Table 4** for the complete list of plasmids and their usage in this study.

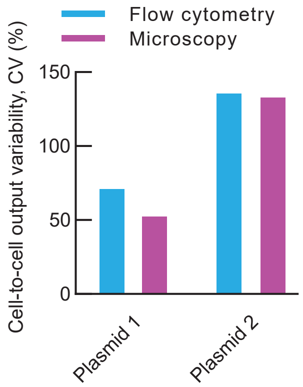

Supplementary Fig. 6 | Cell-to-cell variability in circuit output level is similar when quantified by flow cytometry or microscopy. HEK293 cells were transfected with Plasmid 1 (Equalizer-L episome) or Plasmid 2 (Unregulated CMV episome). Cells transfected with the Equalizer-L episome were induced with 1 ng/mL doxycycline. Two days after transfection, cells were analyzed with either flow cytometry or microscopy.

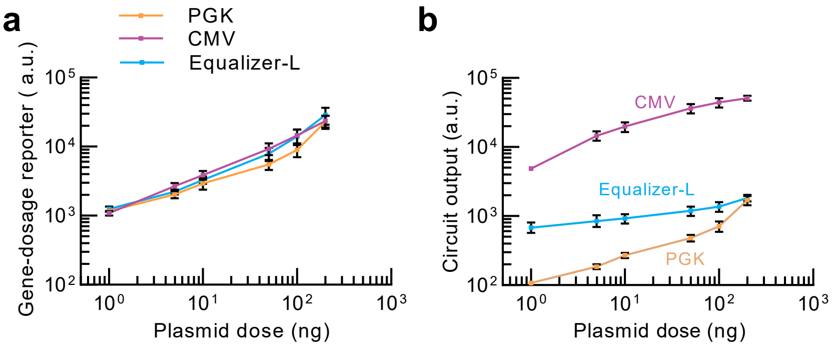

Supplementary Fig. 7 | **Mean gene-dosage reporter and mean circuit output levels of Equalizer-L and unregulated promoter circuits at different transfection plasmid doses.** (**a & b**) Data is from the same experiments as **Fig. 3e-g**. HEK293 cells were transfected with plasmids encoding Equalizer-L, unregulated CMV, or unregulated PGK circuits (see **Supplementary Fig. 5d, g &i**) and analyzed using flow cytometry. Each plasmid also expressed mCherry RFP from the constitutive EF1α promoter. Gene-dosage reporter levels of individual cells were quantified as red fluorescence. (**a**) Mean gene-dosage reporter levels at different doses of transfected plasmids. At each dose, the mean gene-dosage reporter levels of the three circuits were similar. Mean values $\pm$ SEM are shown. n = 6 independent transfections. (**b**) Mean circuit output levels at different doses of transfected plasmids. Mean values $\pm$ SEM are shown. n = 6 independent transfections per circuit.

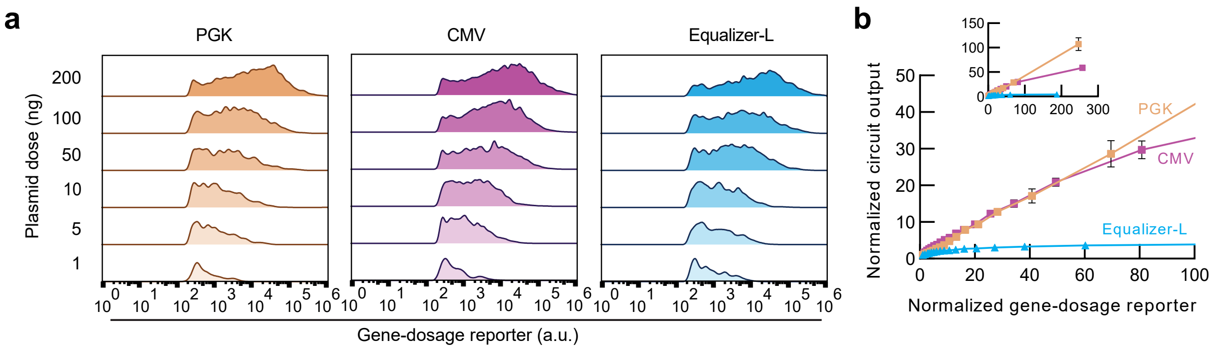

Supplementary Fig. 8 | Equalizer-L buffers circuit output changes resulting from different doses of transfected plasmid. (**a & b**) Data is from the same experiments as **Fig. 3e-g**. HEK293 cells were transfected with plasmids encoding Equalizer-L, unregulated CMV, or unregulated PGK circuits (**Supplementary Fig. 5d, g &i**) and analyzed using flow cytometry. Each plasmid also expressed mCherry RFP from the constitutive EF1α promoter. Gene-dosage reporter levels of individual cells were quantified as red fluorescence. (**a**) Representative histograms of gene-dosage reporter levels. At each plasmid dose, the gene-dosage reporter distributions of the three circuits were similar. (**b**) For each circuit, single-cell flow cytometry data used to generate **Fig. 3e** was pooled and divided into bins with an equal number of data points per bin. For each bin, we computed the mean fluorescence and normalized each mean value to that of the first bin. This approach was used for both axes. The top panel shows the entire range of gene-dosage reporter levels. The bottom panel shows a narrower range of gene-dosage reporter levels. n = 3 independent transfections. Error bars indicate SEM.

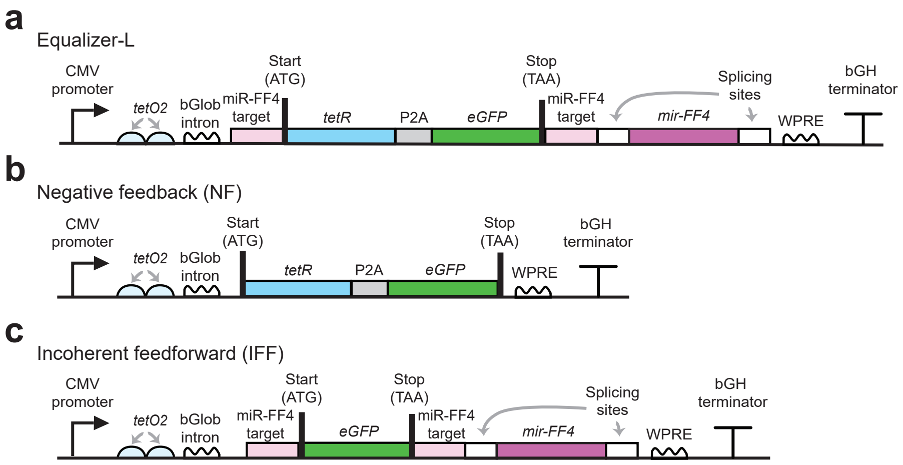

Supplementary Fig. 9 | **Schematics of Equalizer-L and the standalone NF and IFF circuits** (**a**) Equalizer-L (**b**) the negative feedback (NF) circuit (**c**) the incoherent feedforward (IFF) circuit. bGlob intron is the rabbit β-*globin* intron II. WPRE is the woodchuck hepatitis virus post-transcriptional regulatory element. bGlob intron and WPRE were placed at the 5' and 3' UTRs, respectively, to increase the overall gene expression levels.

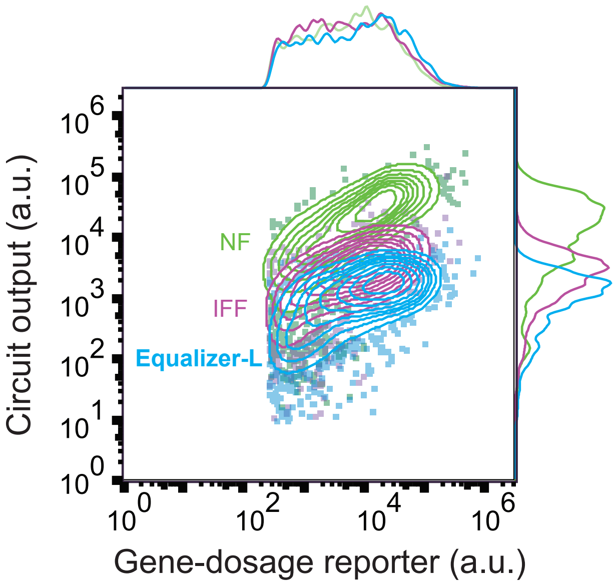

Supplementary Fig. 10 | Equalizer-L produces a narrower circuit output distribution than the standalone NF or IFF circuits in transfected cells. Representative contour plots of flow cytometry data used in **Fig. 4b & c** are shown. The inducer was used at the concentration providing the lowest cell-to-cell output variability: 0.5 ng/mL for the Equalizer-L and 10 ng/mL for the NF circuit. The plasmid expressing mCherry (i.e., an RFP) from the constitutive promoter CMV (**Supplementary Figure 5l**) was co-transfected with the circuit plasmids. Gene-dosage reporter levels were estimated as red fluorescence. Histograms of gene-dosage reporter (*top edge*) and circuit output (*right edge*) are shown. The dots indicate data points that were outside the lowest contour lines (i.e., 10% line)

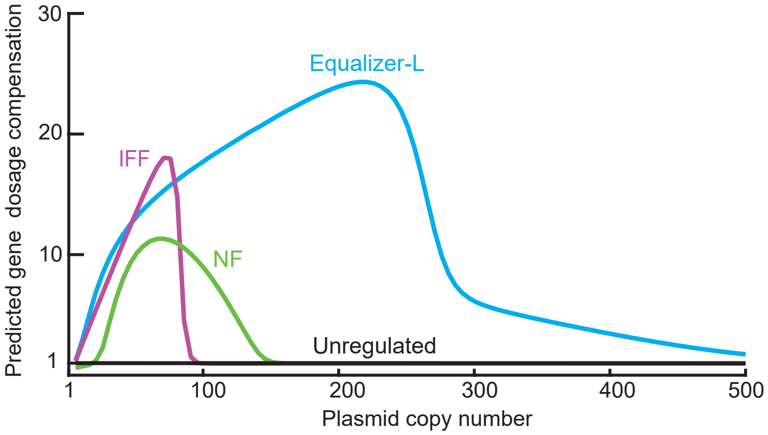

Supplementary Fig. 11 | **Equalizer-L is predicted to show superior dosage compensation compared with IFF and NF circuits.** Equalizer-L and NF circuit were simulated with doxycycline concentrations that were predicted to give the best gene dosage compensation (1 ng/mL for Equalizer-L and 10 ng/mL for NF; see **Fig. 4b**). The predicted gene dosage compensation is defined in **Methods**. See **Supplementary Note 1** for the model description and **Supplementary Tables 1 and 2** for other parameters used in the simulations.

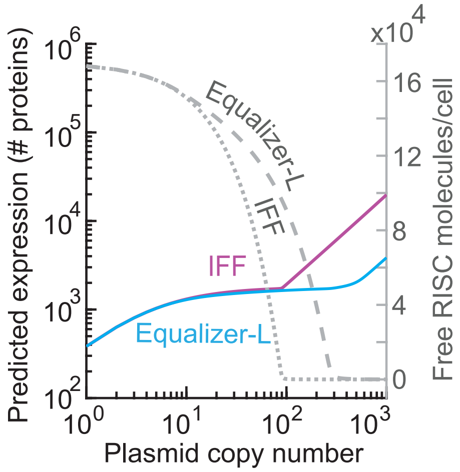

Supplementary Fig. 12 | **Equalizer-L shifts the saturation of RISC to higher plasmid copy numbers.** The solid lines show the predicted output protein levels. Dashed lines show the abundance of free RNA-induced silencing complex (RISC). Because of the shift in RISC saturation, Equalizers can more effectively buffer gene dosage at higher plasmid copy numbers. See **Supplementary Note 1** for model description and **Supplementary Tables 1 and 2** for other parameters used in the simulations.

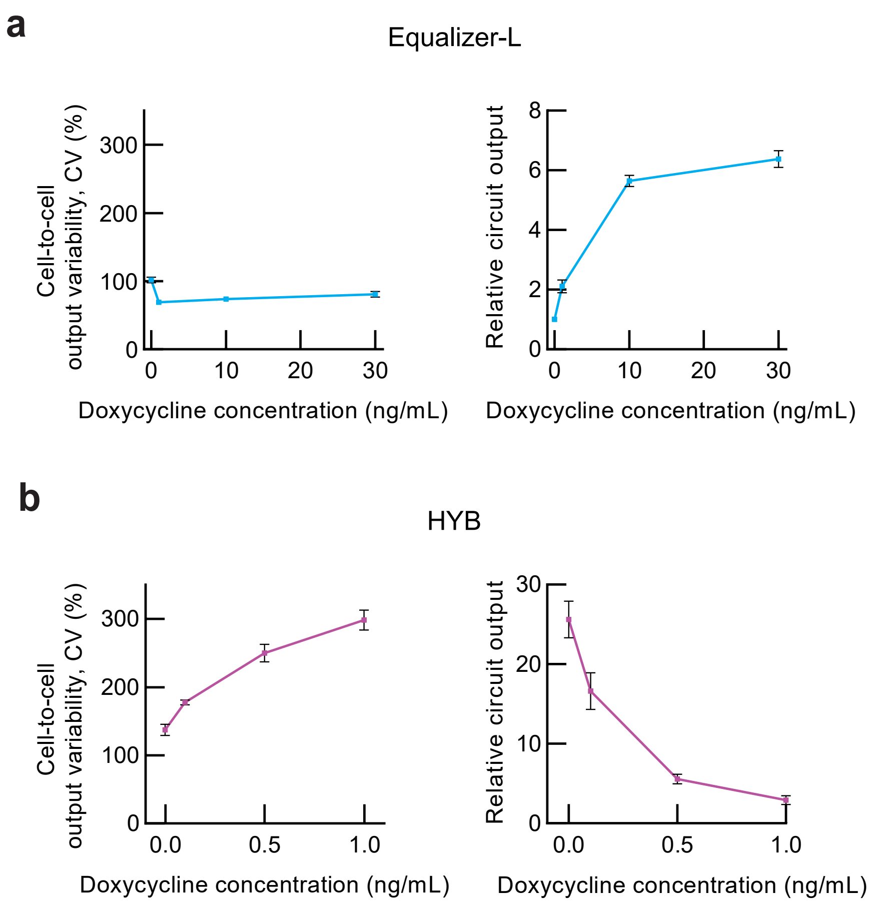

Supplementary Fig. 13 | Determination of the optimal inducer concentrations for the Equalizer-L variant and an alternative NF-IFF hybrid circuit (HYB) to achieve the lowest cell-to-cell output variability. Cell-to-cell output variability (*left*) and mean circuit output levels (*right*) of HEK293T cells harboring (**a**) Equalizer-L or (**b**) HYB and cultured with different inducer concentrations. For the schematics of the gene circuits, see **Fig. 5a & b**. This experiment is similar to that of **Fig. 3a-b** but uses the Equalizer-L circuit variant with mScarlet-I (i.e., an RFP) as a reporter of circuit output and mCitrine (i.e., YFP) as a marker of gene dosage. Mean values $\pm$ SEM are shown. The mean expression values are relative to that of uninduced Equalizer-L. n = 6 independent transfections per circuit.

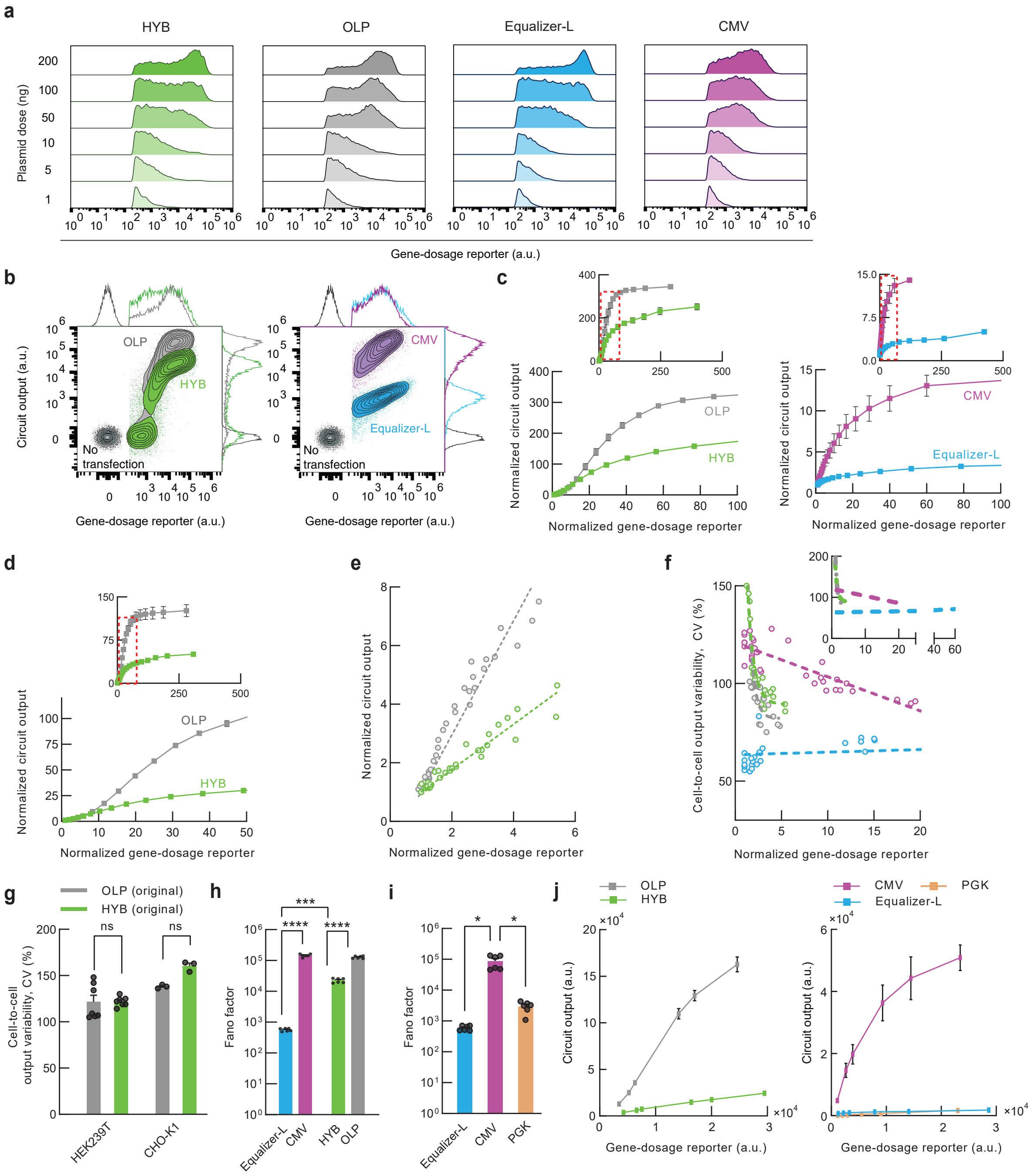

a
HYB
OLP
Equalizer-L
CMV
Plasmid dose (ng)
200
100
50
10
5
1
Gene-dosage reporter (a.u.)
b
Circuit output (a.u.)
OLP
HYB
No transfection
CMV
Equalizer-L
Gene-dosage reporter (a.u.)
c
Normalized circuit output
Normalized gene-dosage reporter
d
e
f
Cell-to-cell output variability, CV (%)
g
OLP (original)
HYB (original)
Cell-to-cell output variability, CV (%)
ns
HEK239T
CHO-K1
h
Fano factor
***
****
i
*
PGK
j
Circuit output (a.u.)

Supplementary Fig. 14 | Equalizer-L has superior gene dosage compensation than HYB, an alternative circuit that combines NF and IFF regulations. (**a**) Representative gene-dosage reporter histograms at the different plasmid doses.(**b**) HYB-transfected cells (*left*) produce a bimodal and wide circuit output distribution while Equalizer-L-transfected cells (*right*) produce a narrow unimodal distribution. Representative contour plots of flow cytometry data. The plasmid dose was 50 ng. Histograms of gene-dosage reporter (top edge) and circuit output (right edge) are shown. The non-transfected cell population is included as a reference to show baseline fluorescence levels. The dots indicate data points outside the lowest contour lines (i.e., 10% line). (**c**) For each circuit, all single-cell flow cytometry data used to generate **Fig. 5e-h** was divided into 20 bins and analyzed using the binning method described in the methods. The top panels show the entire range of gene-dosage reporter values. The bottom panels show a narrower range of gene-dosage reporter (inside red dashed boxes). n = 3 independent transfections. (**d-f**) Data reanalysis after excluding transfected cells (mCitrine$^+$ cells) with no detectable circuit output levels (see panel b). (**d**) Reanalysis of panel c. (**e**) Reanalysis of Fig. 5h. (**f**) Reanalysis of Fig. 5f. (**g**) Cell-to-cell variability in circuit output levels of the original HYB and OLP plasmids in two different cell types. These cell types and plasmids were used in the original study that reported the two circuits [18]. The two plasmids have DsRed-Express rather than mScarlet-I as circuit output reporter. $p > 0.99$ for HEK239T and $p = 0.08$ for CHO-K1 (Sidak's multiple comparison test). n = 7 independent transfections for HEK293T and n = 3 independent transfections for CHO-K1. 50 ng of circuit plasmid was used per transfection reaction. (**h**) The Fano factor of circuit output levels. ***, $p < 0.001$; ****, $p < 0.0001$ (Dunnett's T3 multiple comparison test). n = 6 independent transfections. 50 ng circuit plasmid was used per transfection reaction. This panel was created to reproduce data from a previous study [18]. (**i**) The Fano factor of circuit output levels of circuit plasmids used in Fig. 3c (50 ng transfection dose). n = 6 independent transfections. *, $p < 0.05$ (Dunnett's T3 multiple comparison test) (**j**) When plotting unnormalized circuit output values on a graph with a linear y-axis, flat curves may be due to gene dosage compensation, low circuit output, or both. Data from Fig. 5 (*right*) and Fig. 3 (*left*) are shown for a side-by-side comparison. The unregulated PGK promoter, which has weak expression but does not compensate for gene dosage, produces a flat curve similar to that of Equalizer-L (*right).* For all panels, error bars indicate SEM.

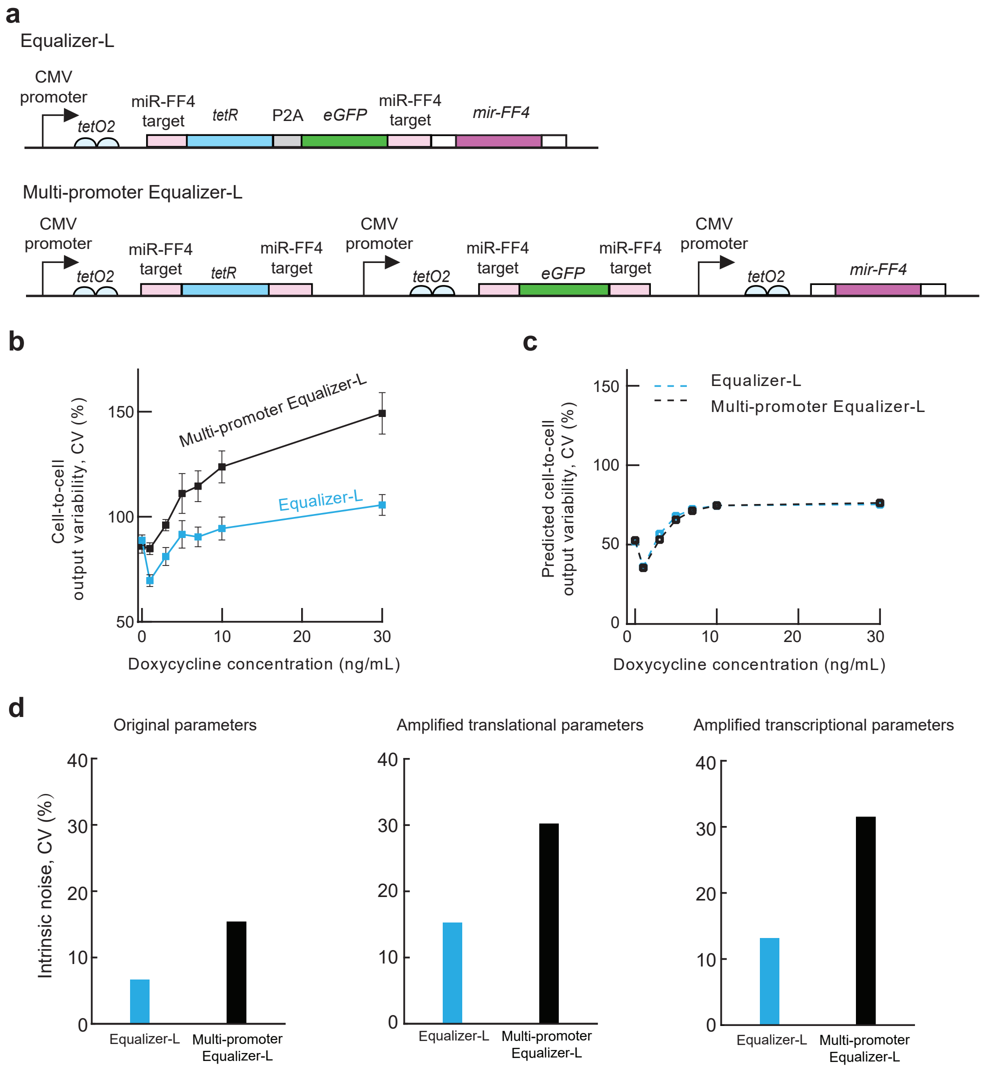

Supplementary Fig. 15 | The multi-promoter variant of the Equalizer-L showed impaired dosage compensation versus the original Equalizer-L. (**a**) Simplified schematics of the original Equalizer-L (*top*) and a multiple promoter implementation (*bottom*). More detailed schematics are shown in **Supplementary Fig. 5d-e**. (**b**) The multi-promoter Equalizer-L produces greater cell-to-cell variability in circuit output levels at most inducer concentrations compared than single-promoter Equalizer-L. Mean values ± SEM are shown. n = 6 transfections/condition. p = 0.038 (two-sided t-test comparing the areas under the curve). (**c**) Deterministic simulations predicted that the two Equalizer-L variants would produce similar cell-to-cell variability across the tested range of inducer concentrations. (**d**) The multi-promoter Equalizer-L is predicted to produce ∼ 2-fold greater intrinsic noise than the original Equalizer-L. Three different sets of translational and transcriptional parameters produce similar results. This greater intrinsic noise may explain why the multi-promoter Equalizer-L produced higher cell-to-cell variability experimentally (panel b) but not in deterministic simulations that do not account for intrinsic noise (panel c). The simulated doxycycline concentration was 0.5 ng/mL. Addition information is provided in **Supplementary Note 6**.

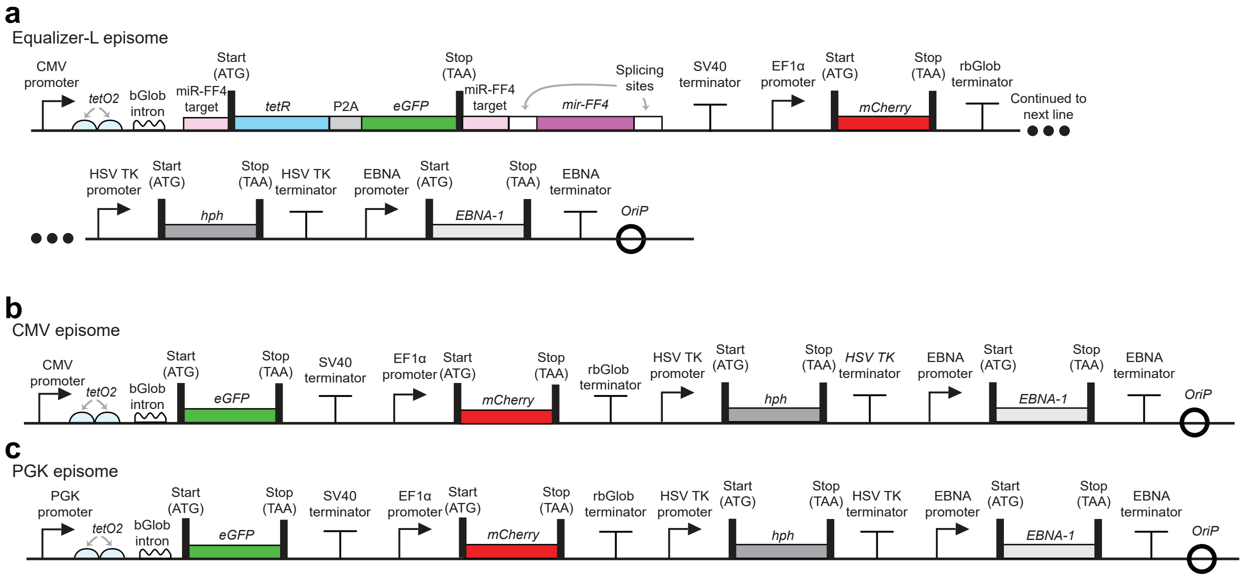

Supplementary Fig. 16 | Schematics of episomal Equalizer-L and unregulated plasmids used to characterize gene dosage compensation of Equalizer-L over a two-month period. (**a-c**) bGlob intron is the rabbit β*-globin* intron II. bGlob intron was placed at the 5' UTR to increase the overall gene expression levels. *Hph* gene encodes the hygromycin-B-phosphotransferase protein, which confers resistance to Hygromycin-B. EBNA-1 is the Epstein-Barr nuclear antigen-1. OriP is the origin of replication of the Epstein-Barr virus. EBNA-1 and OriP are necessary to maintain episomal plasmids in transfected cells.

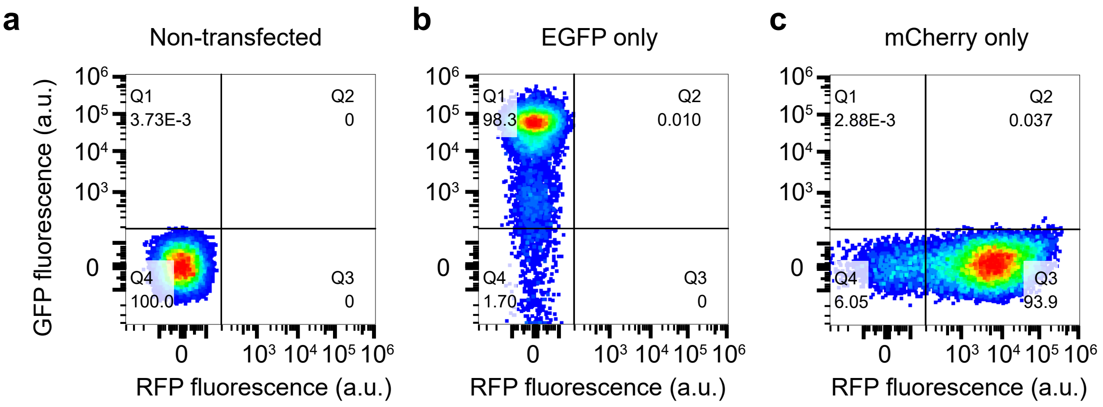

Supplementary Fig. 17 | Determination of fluorescence thresholds between expressing and non-expressing cells in flow cytometry experiments with episomes. The boundaries between the quadrants (Q1-4) were set to minimize false positives using cellular fluorescence distributions from (**a**) untransfected (GFP^- and RFP^-) cells, (**b**) cells stably expressing EGFP from the genome, and (**c**) episomes encoding an EF1α-mCherry expression cassette. The number inside each quadrant (Q1-4) represents the number of cells in the quadrant as a percentage of total cells.

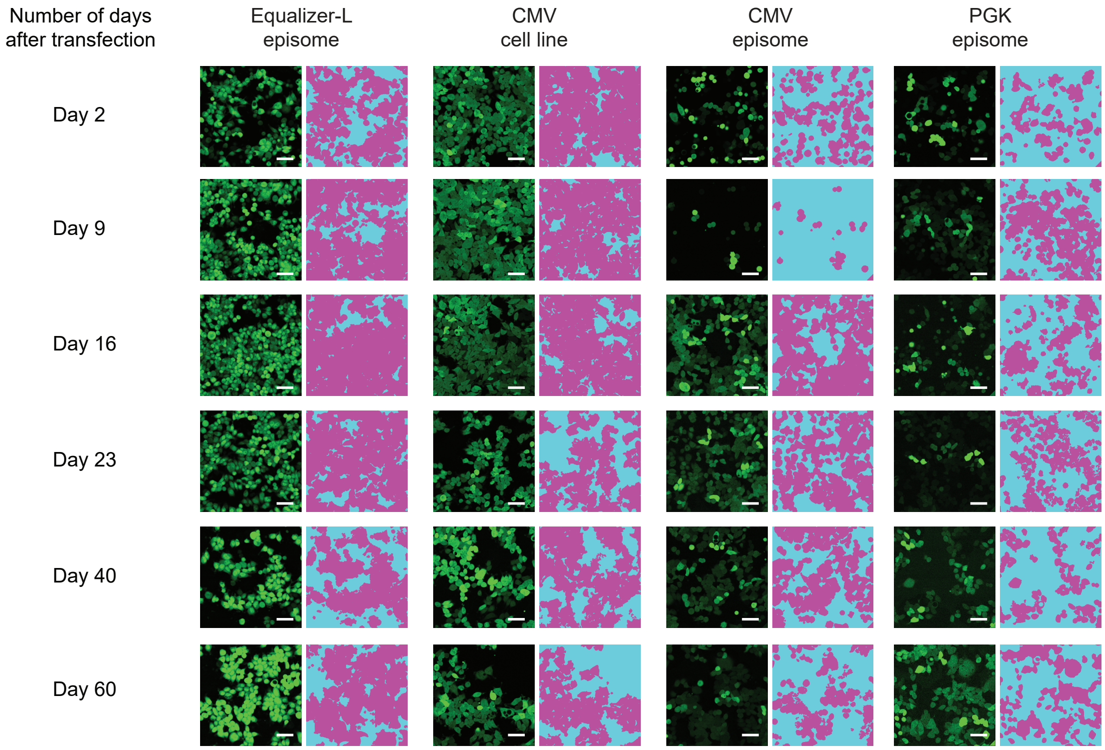

Supplementary Fig. 18 | **Representative fluorescence images of episome-carrying cells over a two-month period.** The same cell populations analyzed by flow cytometry (**Supplementary Fig. 19**) were also imaged by fluorescence microscopy. Representative images of n = 4 independent trials are shown. To help distinguish dim cells from the background, we also show binary masks beside each fluorescent image. Magenta denotes cells, and blue denotes background. We used two-photon microscopy to produce optical sectioning, thereby reducing variations in brightness due to differences in cell thickness. Scale bars, 50 μm.

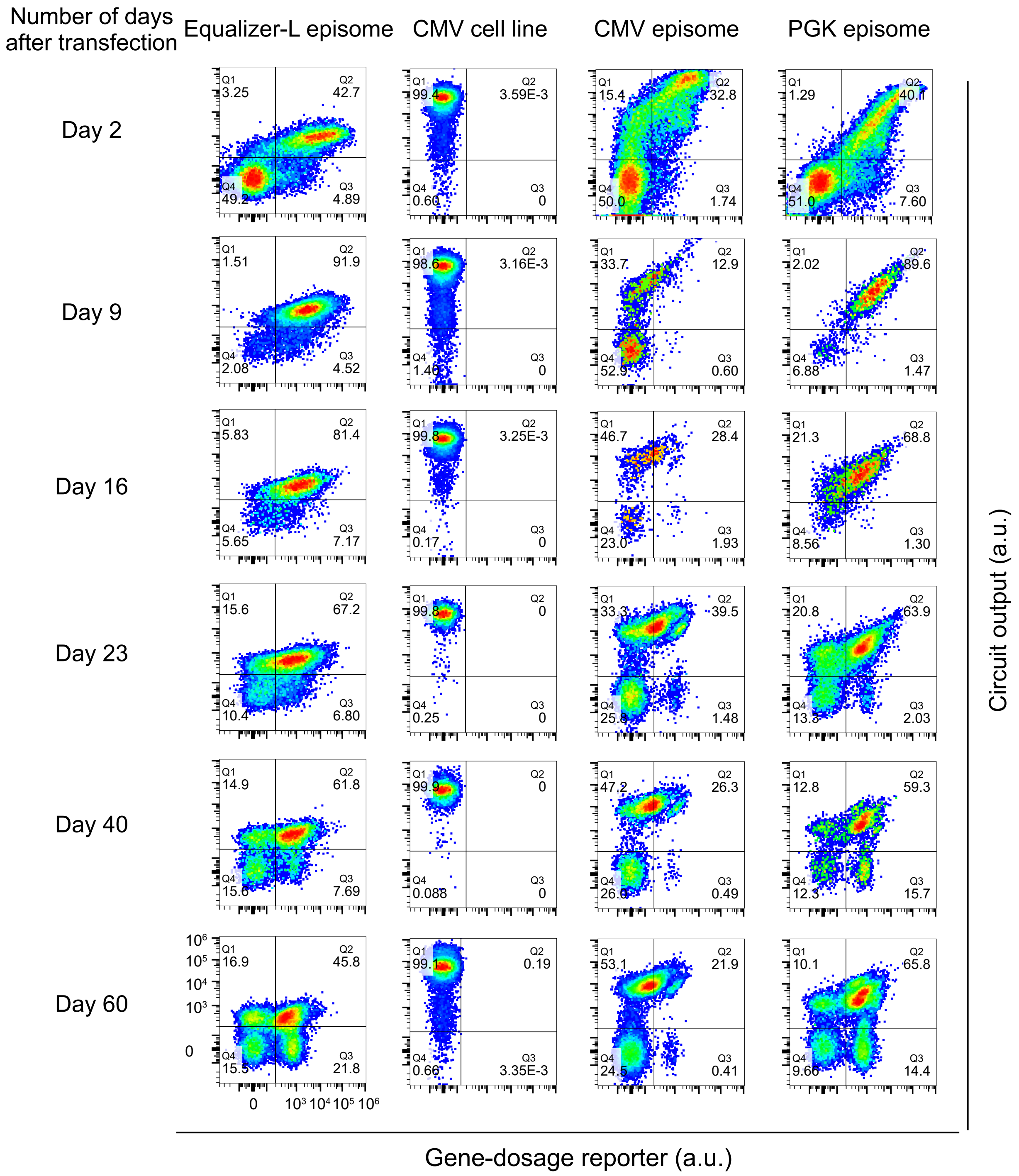

Supplementary Fig. 19 | Circuit expression distributions from populations of episome-carrying cells over a two-month period. The fluorescence of individual HEK293 cells was determined by flow cytometry. As a control, we also grew and analyzed cells from a line containing a chromosomally-integrated CMV-EGFP expression cassette (*column 2*). This line was not transfected with episomes, but passaged and analyzed on the same days as other episome-transfected cells. Schematics of episome plasmids are shown in **Supplementary Fig. 16**. Each episome plasmid expressed mCherry (i.e., an RFP) from a constitutive EF1α promoter. Gene-dosage reporter level of individual cells was quantified as red fluorescence of the cells. Circuit output level was defined as EGFP fluorescence. The thresholds between GFP$^+$ and GFP$^-$ cells and between RFP$^+$ and RFP$^-$ cells were defined using control cell populations (**Supplementary Fig. 17**). Flow cytometry data are presented on biexponential axes. The number inside each quadrant (Q1-4) represents the number of cells in the quadrant as a percentage of total cells. The axes numbers shown in the bottom left plot apply to the rest of the plots.

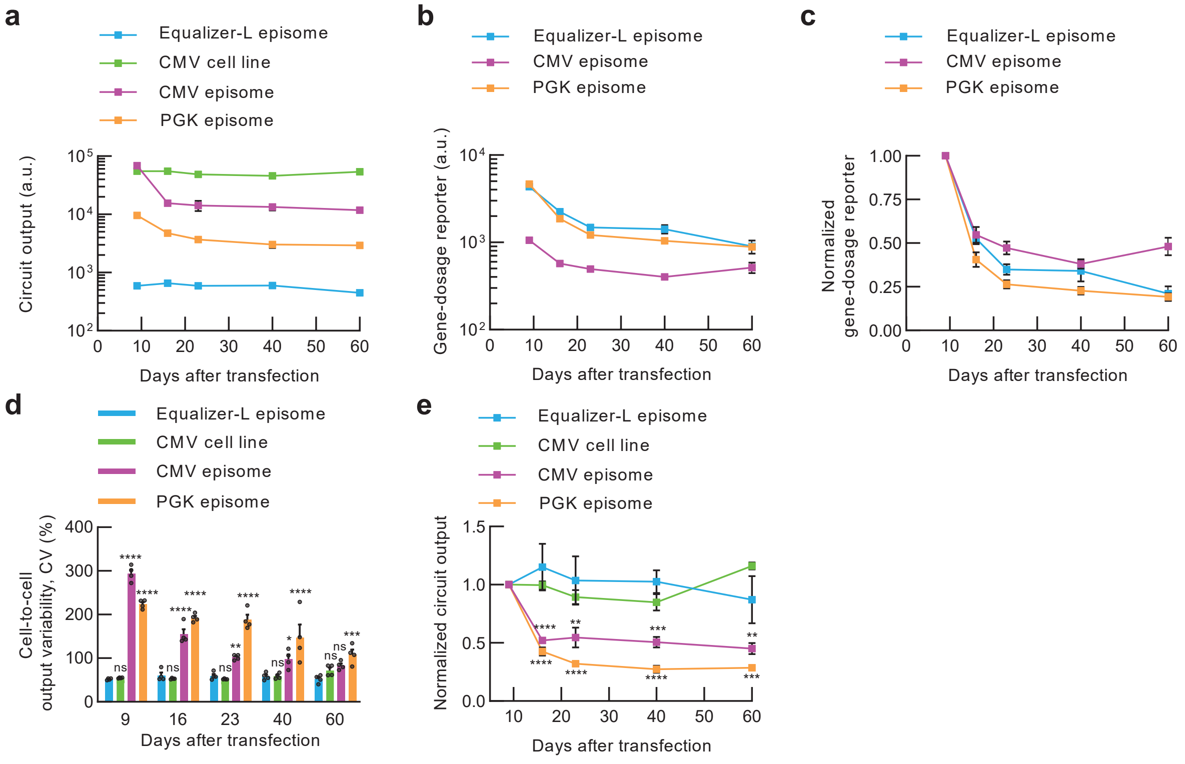

Supplementary Fig. 20 | Circuit output and gene-dosage marker levels from Day 9 to Day 60 after transfection with episomal vectors. (**a**) Circuit output. (**b**) Gene dosage marker, defined as the red fluorescence from the EF1α-mCherry cassette encoded on each circuit plasmid (see **Supplementary Fig. 16** for plasmid schematics). (**c**) Mean gene-dosage marker values from panel b were normalized to the mean at 9 days after transfection. (**d-e**) Reanalysis of (**d**) cell-to-cell output variability and (**e**) circuit output levels shown in **Fig. 6a & c**, respectively. Reanalysis was conducted after including GFP$^+$ and RFP$^-$ cells (see **Supplementary Fig. 17**). ns, not significant; *, $p < 0.05$; **, $p < 0.01$; ***, $p < 0.001$; ****, $p < 0.0001$ (Tukey's multiple comparison test). For all panels, mean values $\pm$ SEM are shown. n = 4 independent trials. The data shown here is from the same experiments as illustrated in **Fig. 6**.

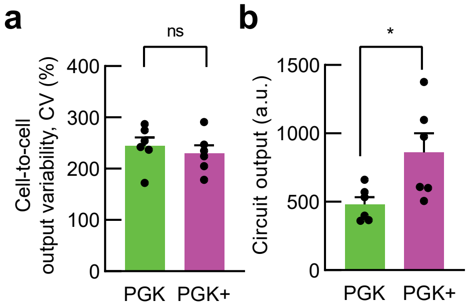

Supplementary Fig. 21 | Addition of the *tetO2* site in the promoter region, the rabbit β-globin intron II sequence in the 5'UTR, and WPRE sequence in the 3' UTR has minimal effect on cell-to-cell output variability. (**a-b**) Unregulated PGK plasmid or Unregulated PGK+ plasmid, a variant with additional sequences, were transfected in HEK293 cells. The fluorescence of single cells was quantified by flow cytometry. Schematics of PGK and PGK+ plasmids can be found in **Supplementary Fig. 5h and i**, respectively. (**a**) Cell-to-cell output variability. p = 0.54 (two-sided unpaired t-test). (**b**) Circuit output levels. p = 0.043 (two-sided unpaired t-test). For both panels, mean values ± SEM are shown. Black circles represent n = 6 independent transfections.

Supplementary Tables

Supplementary Table 1: mass action kinetic parameters

Parameter	Value	Unit	Reference
k_transcription	4.67e-2	1/second	[19]
k_dicer	1e-3	1/second	[20]
k_drosha	1e-2	1/second	[2]
k_Inducer_bind_TetR	1.0e-5	1/(molecule*second)	[21]
k_Inducer_dissociates_TetR	2.0e-8	1/second	[21]
k_inducer_deg	3.33e-4	1/second	[1]
k_influx[a]	0.156	molecule/second	[1]
k_microRNA_bind_RISC	1.0e-5	1/(molecule*second)	[20]
k_microRNA_deg	2.88e-4	1/second	[19]
k_microRNA_RISC_dissociation	2.16e-5	1/second	[20]
k_mRNA_deg	2.88e-4	1/second	[19]
k_mRNAmicroRNA_deg	2.88e-4	1/second	[19]
k_POI_deg	9.67e-5	1/second	[19]
k_RISC_complex_deformation	0.303	1/second	estimated
k_RISC_complex_formation	1.84e-6	1/(molecule*second)	[20]
k_slicer	7.0e-3	1/second	[20]
k_splicing	2.0e-3	1/second	[2]
k_TetR_deg	9.67e-5	1/second	[19]
k_Inducer-TetR_deg	9.67e-5	1/second	[19]
k_translation	3.33e-4	1/second	[19] [20]
k_on	3.10e-4	1/second	[23]
k_off	4.18e-4	1/second	[23]
RISC_initial	1.7e+5	molecules	[24]

[a] 0.156 molecule/second corresponds to a extracellular doxycycline concentration of 1 ng/ml, assuming a nucleus volume of 690 μm^3 [22]; this influx rate linearly scales with extracellular doxycycline concentration

See **Supplementary Note 4** for methods used for estimation of the k_RISC_complex_formation parameter.

Supplementary Table 2: non-mass action kinetic parameters

Parameter	Value	Unit	Reference
k_transcription_max	4.67e-2	1/second	[19]
K_{50}	183	molecule	[1]
leakage	0.25	dimensionless	estimated

See **Supplementary Note 3** for methods used for estimation of the leakage parameter.

Supplementary Table 3: gamma-distributed plasmid copy number parameters

Parameter	Value	Unit	Reference
shape parameter k	0.57	dimensionless	estimated
scale parameter θ	120	dimensionless	estimated

See **Supplementary Note 3** for methods used for the estimation of k and θ.

Supplementary Table 4: plasmids used in this study

Name	Description	Usage	Schematic	Addgene; GenBank
Equalizer-H plasmid	CMV_*tetO2*_promoter-bGlob_intron-*tetR*-P2A-*eGFP*-MiR(FF3)_target-*mir(FF3)*-WPRE-bGH_terminator	Fig. 3a-c	Supp. Fig. 5a	169367; MW962297
Equalizer-M plasmid	CMV_*tetO2*_promoter-bGlob_intron-*tetR*-P2A-*eGFP*-MiR(FF4)_target-*mir(FF4)*-WPRE-bGH_terminator	Fig. 3a-c	Supp. Fig. 5b	169731; MW987521
Equalizer-L plasmid	CMV_*tetO2*_promoter-bGlob_intron-MiR(FF4)_target-*tetR*-P2A-*eGFP*-MiR(FF4)_target-*mir(FF4)*-WPRE-bGH_terminator	Fig. 3a-c; Fig. 4a-b; Supp. Fig. 9	Supp. Fig. 5c	169732; MW987522
Equalizer-L plasmid with onboard mCherry cassette	CMV_*tetO2*_promoter-bGlob_intron-MiR(FF4)_target-*tetR*-P2A-*eGFP*-MiR(FF4)_target-*mir(FF4)*-WPRE-bGH_terminator-*EF1*α_promoter-*mCherry*-rbGlob_terminator	Fig. 3d-g; Supp. Fig. 7-8; Supp. Fig. 14i-j; Supp. Fig. 15b	Supp. Fig. 5d	169735; MW987525
Multi-promoter Equalizer-L plasmid with onboard mCherry cassette	CMV_*tetO2*_promoter-bGlob_intron-MiR(FF4)_target-*eGFP*_MiR(FF4)_target-WPRE-bGH_terminator-CMV_*tetO2*_promoter-bGlob_intron-MiR(FF4)_target-*tetR*-MiR(FF4)_target-WPRE-bGH_terminator-CMV_*tetO2*_promoter-bGlob_intron-*mir(FF4)*-WPRE-bGH_terminator-*EF1*α_promoter-*mCherry*-rbGlob_terminator	Supp. Fig. 15b	Supp. Fig. 5e	N/A; MZ099631
Equalizer-L (mScarlet-I) plasmid with onboard mCitrine cassette	CMV_*tetO2*_promoter-bGlob_intron-MiR(FF4)_target-*tetR*-P2A-*mScarletI*-MiR(FF4)_target-*mir(FF4)*-WPRE-bGH_terminator-*EF1*α_promoter-*mCitrine*-SV40_terminator-bGH_terminator	Fig. 5; Supp. Fig. 13a; Supp. Fig. 14a-c,f,h	Fig. 5a	169737; MW987526

Continued on next page

Equalizer-L episome	CMV_*tetO2*_promoter-bGlob_intron-MiR(FF4)_target-*tetR*-P2A-*eGFP*-MiR(FF4)_target-*mir(FF4)*-SV40_terminator-*EF1*α_promoter-*mCherry*-rbGlob_terminator-HSV_*TK*_promoter-*hph*-HSV_*TK*_terminator-*EBNA*_promoter-*EBNA1*-*EBNA*_terminator-*OriP*	Fig. 6; Supp. Fig. 6; Supp. Fig. 18-20	Supp. Fig. 16a	169738; MW987527
CMV+ plasmid	CMV_*tetO2*_promoter-bGlob_intron-*eGFP*-WPRE-bGH_terminator	Fig. 3c	Supp. Fig. 5f	169740; MW987529
CMV+ plasmid with onboard mCherry cassette	CMV_*tetO2*_promoter-bGlob_intron-*eGFP*-WPRE-bGH_terminator-*EF1*α_promoter-*mCherry*-rbGlob_terminator	Fig. 3d-g; Supp. Fig. 7-8; Supp. Fig. 14i-j	Supp. Fig. 5g	169741; MW987530
CMV episome	CMV_*tetO2*_promoter-bGlob_intron-*eGFP*-WPRE-bGH_terminator-*EF1*α_promoter-*mCherry*-rbGlob_terminator-HSV_*TK*_promoter-*hph*-HSV_*TK*_terminator-*EBNA*_promoter-*EBNA1*-*EBNA*_terminator-*OriP*	Fig. 6; Supp. Fig. 6; Supp. Fig. 18-20	Supp. Fig. 16b	169742; MW987531
CMV+ (mScarlet-I) plasmid with onboard mCitrine cassette	CMV_*tetO2*_promoter-bGlob_intron-*mScarletI*-WPRE-bGH_terminator-*EF1*α_promoter-*mCitrine*-rbGlob_terminator	Fig. 5; Supp. Fig. 14a-c,f,h	Fig. 5c	169743; MW987532
PGK plasmid	*PGK*_promoter-*eGFP*-bGH_terminator	Fig. 3c; Supp. Fig. 22	Supp. Fig. 5h	169744; MW987533
PGK+ plasmid with onboard mCherry cassette	*PGK*_*tetO2*_promoter-bGlob_intron-*eGFP*-WPRE-bGH_terminator-*EF1*α_promoter-*mCherry*-rbGlob_terminator	Fig. 3e-g; Supp. Fig. 7-8; Supp. Fig. 14i-j; Supp Fig. 22	Supp. Fig. 5i	169745; MW987534

Continued on next page

PGK episome with onboard mCherry cassette	PGK_tetO2_promoter-bGlob_intron-eGFP-WPRE-bGH_terminator-EF1α_promoter-mCherry-rbGlob_terminator-HSV_TK_promoter-hph-HSV_TK_terminator-EBNA_promoter-EBNA1-EBNA_terminator-OriP	Fig. 6; Supp. Fig. 18-20	Supp. Fig. 16c	170041; MW962296
UBC plasmid	UBC_promoter-eGFP-bGH_terminator	Fig. 3c	Supp. Fig. 5j	169746; MW987535
CMV mCherry plasmid	CMV_promoter-mCherry-bGH_terminator	Fig. 3a-c; Fig. 4a-c; Supp. Fig. 3; Supp. Fig. 10; Supp. Fig. 22	Supp. Fig. 5k	MZ220611
Negative feedback (NF) plasmid	CMV_tetO2_promoter-bGlob_intron-tetR-P2A-eGFP-WPRE-bGH_terminator	Fig. 4a-b; Supp. Fig. 3; Supp. Fig. 10	Supp. Fig. 9b	169747; MW987536
Incoherent feedforward (IFF) plasmid	CMV_tetO2_promoter-bGlob_intron-MiR(FF4)_target-eGFP-MiR(FF4)_target-mir(FF4)-WPRE-bGH_terminator	Fig. 4c; Supp. Fig. 10	Supp. Fig. 9c	169748; MW987537
HYB plasmid	EF1α_promoter-mCitrine-SV40_terminator-bGH_terminator-SV40_promoter-tTA::Cerulean-MiR(FF4)_targetx3-SV40_terminator-bGH_terminator-TRE_promoter-mScarletI(mir_FF4)-MiR(FF4)_targetx3-SV40_terminator	Fig. 5; Supp. Fig. 13b; Supp. Fig. 14a-f,h,j	Fig. 5b	MZ220609
HYB (original) plasmid	EF1α_promoter-mCitrine-SV40_terminator-bGH_terminator-SV40_promoter-tTA::Cerulean-MiR(FF4)_targetx3-SV40_terminator-bGH_terminator-TRE_promoter-DsRed(mir_FF4)-MiR(FF4)_targetx3-SV40_terminator	Supp. Fig. 14g	ref. [18]	Contact authors of ref. [18] for the sequence

Continued on next page

OLP plasmid	*EF1α*_promoter-*mCitrine*-SV40_terminator-bGH_terminator-SV40_promoter-*tTA::Cerulean*-MiR(FF5)_targetx3-SV40_terminator-bGH_terminator-TRE_promoter-*mScarletI(mir_FF4)*-MiR(FF5)_targetx3-SV40_terminator	Fig. 5;<br>Supp. Fig. 14a-f,h,j	Fig. 5d	MZ220610
OLP (original) plasmid	*EF1α*_promoter-*mCitrine*-SV40_terminator-bGH_terminator-SV40_promoter-*tTA::Cerulean*-MiR(FF5)_targetx3-SV40_terminator-bGH_terminator-TRE_promoter-*DsRed(mir_FF4)*-MiR(FF5)_targetx3-SV40_terminator	Supp. Fig. 14g	ref. [18]	Contact authors of ref. [18] for the sequence

References

[1] Nevozhay, D., Adams, R. M., Murphy, K. F., Josić, K. & Balázsi, G. Negative autoregulation linearizes the dose–response and suppresses the heterogeneity of gene expression. *PNAS* **106**, 5123–5128 (2009).

[2] Bleris, L. *et al.* Synthetic incoherent feedforward circuits show adaptation to the amount of their genetic template. *Mol. Syst. Biol.* **7**, 519–519 (2011).

[3] Siciliano, V. *et al.* Construction and modelling of an inducible positive feedback loop stably integrated in a mammalian cell-line. *PLoS Comput. Biol* **7**, e1002074 (2011).

[4] Kim, J. H. *et al.* High cleavage efficiency of a 2A peptide derived from porcine teschovirus-1 in human cell lines, zebrafish and mice. *Plos One* **6**, e18556 (2011).

[5] Friedman, N., Cai, L. & Xie, X. S. Linking stochastic dynamics to population distribution: An analytical framework of gene expression. *Phys. Rev. Lett.* **97**, 168302 (2006).

[6] Schreiber, J., Arter, M., Lapique, N., Haefliger, B. & Benenson, Y. Model-guided combinatorial optimization of complex synthetic gene networks. *Mol. Syst. Biol.* **12**, 899 (2016).

[7] Salomon, W., Jolly, S., Moore, M., Zamore, P. & Serebrov, V. Single-molecule imaging reveals that argonaute reshapes the binding properties of its nucleic acid guides. *Cell* **162**, 84–95 (2015).

[8] Denzler, R. *et al.* Impact of microrna levels, target-site complementarity, and cooperativity on competing endogenous rna-regulated gene expression. *Mol. Cell.* **64**, 565–579 (2016).

[9] Swain, P. S., Elowitz, M. B. & Siggia, E. D. Intrinsic and extrinsic contributions to stochasticity in gene expression. *PNAS* **99**, 12795–12800 (2002).

[10] Sepúlveda, L. A., Xu, H., Zhang, J., Wang, M. & Golding, I. Measurement of gene regulation in individual cells reveals rapid switching between promoter states. *Science* **351**, 1218–1222 (2016).

[11] Sanft, K. R. *et al.* Stochkit2: software for discrete stochastic simulation of biochemical systems with events. *Bioinformatics* **27**, 2457–2458 (2011).

[12] Sanchez, A., Choubey, S. & Kondev, J. Regulation of noise in gene expression. *Annu Rev Biophys* **42**, 469–491 (2013).

[13] Paulsson, J. Summing up the noise in gene networks. *Nature* **427**, 415–418 (2004).

[14] Swain, P. S., Elowitz, M. B. & Siggia, E. D. Intrinsic and extrinsic contributions to stochasticity in gene expression. *PNAS* **99**, 12795–12800 (2002).

[15] Fu, A. Q. & Pachter, L. Estimating intrinsic and extrinsic noise from single-cell gene expression measurements. *Stat Appl Genet Mol Biol* **15**, 447–471 (2016).

[16] Barker, P. *The logic of scientific design*. Encyclopedia of Research Design (SAGE Publications, Inc., 2010).

[17] Hortsch, S. K. & Kremling, A. *Stochastic models for studying the role of cellular noise and heterogeneity*, 34–44 (Elsevier, 2021).

[18] Lillacci, G., Benenson, Y. & Khammash, M. Synthetic control systems for high performance gene expression in mammalian cells. *Nucleic Acids Res* **46**, 9855–9863 (2018).

[19] Tigges, M., Marquez-Lago, T. T., Stelling, J. & Fussenegger, M. A tunable synthetic mammalian oscillator. *Nature* **457**, 309 (2009). Citation for protein requiring narrow concentration range.

[20] Tigges, M., Dénervaud, N., Greber, D., Stelling, J. & Fussenegger, M. A synthetic low-frequency mammalian oscillator. *Nucleic Acid Res* **38**, 2702–2711 (2010).

[21] To, T.-L. & Maheshri, N. Noise can induce bimodality in positive transcriptional feedback loops without bistability. *Science* **327**, 1142–1145 (2010).

[22] Monier, K., Armas, J. C. G., Etteldorf, S., Ghazal, P. & Sullivan, K. F. Annexation of the interchromosomal space during viral infection. *Nat. Cell Biol.* **2**, 661 (2000).

[23] Molina, N. *et al.* Stimulus-induced modulation of transcriptional bursting in a single mammalian gene. *PNAS* **110**, 20563–20568 (2013).

[24] Wang, D. *et al.* Quantitative functions of argonaute proteins in mammalian development. *Genes Dev* **26**, 693–704 (2012).
