# Supplementary material for: A synthetic circuit for buffering gene dosage variation between individual mammalian cells
Source: Nat Commun. 2021 Jul 5;12:4132. doi: 10.1038/s41467-021-23889-0 (PMC8257781; doi:10.1038/s41467-021-23889-0)
Supplement: Supplementary file 2 — Reporting Summary [file 41467_2021_23889_MOESM2_ESM.pdf]

nature research

Corresponding author(s): St-Pierre, François
Last updated by author(s): May 3, 2021

Reporting Summary

Nature Research wishes to improve the reproducibility of the work that we publish. This form provides structure for consistency and transparency in reporting. For further information on Nature Research policies, see our Editorial Policies and the Editorial Policy Checklist.

Statistics

For all statistical analyses, confirm that the following items are present in the figure legend, table legend, main text, or Methods section.

n/a	Confirmed	
☐	☒	The exact sample size (*n*) for each experimental group/condition, given as a discrete number and unit of measurement
☐	☒	A statement on whether measurements were taken from distinct samples or whether the same sample was measured repeatedly
☐	☒	The statistical test(s) used AND whether they are one- or two-sided<br>*Only common tests should be described solely by name; describe more complex techniques in the Methods section.*
☒	☐	A description of all covariates tested
☐	☒	A description of any assumptions or corrections, such as tests of normality and adjustment for multiple comparisons
☐	☒	A full description of the statistical parameters including central tendency (e.g. means) or other basic estimates (e.g. regression coefficient) AND variation (e.g. standard deviation) or associated estimates of uncertainty (e.g. confidence intervals)
☐	☒	For null hypothesis testing, the test statistic (e.g. *F*, *t*, *r*) with confidence intervals, effect sizes, degrees of freedom and *P* value noted<br>*Give P values as exact values whenever suitable.*
☒	☐	For Bayesian analysis, information on the choice of priors and Markov chain Monte Carlo settings
☒	☐	For hierarchical and complex designs, identification of the appropriate level for tests and full reporting of outcomes
☒	☐	Estimates of effect sizes (e.g. Cohen's *d*, Pearson's *r*), indicating how they were calculated

Our web collection on statistics for biologists contains articles on many of the points above.

Software and code

Policy information about availability of computer code

Data collection	The software used for data collection is mentioned in Methods. Briefly, microscopy data was collected using NIS-Elements AR (version 4.60, Nikon Instruments). Flow cytometry data was collected using Attune NxT Acoustic Focusing Cytometer Software (version 4.0.1445.0).
Data analysis	All the software used for data analysis is mentioned in Methods. Briefly, flow cytometry data was analyzed using FlowJo (version 10.6.1, FlowJo LLC). Automated image analysis was conducted using MATLAB (version r2019b, MathWorks); image segmentation was conducted using ILASTIK (version 1.3.2) and MATLAB. Average values and standard errors of the mean output levels and coefficient of variation (CV) of output levels for the transfection replicates were calculated using MATLAB or Prism (version 9.0.1, GraphPad). All MATLAB code used in the simulations is available on GitHub: https://github.com/stpierrelab/Equalizer

For manuscripts utilizing custom algorithms or software that are central to the research but not yet described in published literature, software must be made available to editors and reviewers. We strongly encourage code deposition in a community repository (e.g. GitHub). See the Nature Research guidelines for submitting code & software for further information.

Data

Policy information about availability of data

All manuscripts must include a data availability statement. This statement should provide the following information, where applicable:
- Accession codes, unique identifiers, or web links for publicly available datasets
- A list of figures that have associated raw data
- A description of any restrictions on data availability

Annotated plasmid sequences are available from GenBank (Accession numbers: MW962296-MW962297, MW987521-MW987537, MZ099631). Detailed information on statistical tests are available in Supplementary Statistics. Source data for the main figures are provided with this paper as supplementary files. They are also available on GitHub: https://github.com/stpierrelab/Equalizer/tree/main/ExperimentData. All other data are available upon reasonable request.

Field-specific reporting

Please select the one below that is the best fit for your research. If you are not sure, read the appropriate sections before making your selection.

- [x] Life sciences
- [ ] Behavioural & social sciences
- [ ] Ecological, evolutionary & environmental sciences

For a reference copy of the document with all sections, see nature.com/documents/nr-reporting-summary-flat.pdf

Life sciences study design

All studies must disclose on these points even when the disclosure is negative.

Sample size	Biological replicates were defined as independent transfections or cell cultures. Sample sizes of n = 3 to 9 per condition were used for experiments because these sample sizes are standard practice in the field and because variations between replicates were low compared with the effect size observed and reported here. For each replicate, hundreds to thousands of cells were analyzed. Statistical analyses to determine optimal sample sizes were not conducted.
Data exclusions	We excluded non-transfected cells from analysis. The method used to distinguish transfected cells from non-transfected is described below in the flow cytometry section. We excluded a minute number of obvious outlier values (e.g. ~1 cell / 10,000 cells with a z-score of > ~50). These outlier values are likely cells from previous samples that stuck to the walls of the flow cytometer capillaries and were not removed in the wash steps between samples.
Replication	Three to nine independent transfections (n=3 to 9) per condition were used for all the experiments. For most experiments, replicates were conducted once on a separate day and successfully showed the same trends.
Randomization	Randomization was not critical in this study as there was no need to allocate cells into experimental groups.
Blinding	Aspects of blinding and other measures were used to minimize potential bias. (1) All sample groups were prepared and assayed using the same predefined protocols. (2) Samples were assayed automatically by the Attune NxT Flow Cytometer Autosampler (Thermo Fisher Scientific) with no interference from the experimenter after starting sample acquisition. (3) The flow cytometer named data files using well numbers instead of sample names. The experimenter mapped the sample identities to well numbers only at the final analysis stage. (4) Identical analysis was done on all samples using predefined routines. (5) Only quantitative data were used to compare different conditions. (6) Qualitative data (e.g., fluorescence images) were only used to confirm the conclusions derived using quantitative data. (7) For microscopy, fields of view were randomly selected to acquire images.

Reporting for specific materials, systems and methods

We require information from authors about some types of materials, experimental systems and methods used in many studies. Here, indicate whether each material, system or method listed is relevant to your study. If you are not sure if a list item applies to your research, read the appropriate section before selecting a response.

Materials & experimental systems

n/a	Involved in the study	
[x]	[ ]	Antibodies
[ ]	[x]	Eukaryotic cell lines
[x]	[ ]	Palaeontology and archaeology
[x]	[ ]	Animals and other organisms
[x]	[ ]	Human research participants
[x]	[ ]	Clinical data
[x]	[ ]	Dual use research of concern

Methods

n/a	Involved in the study	
[x]	[ ]	ChIP-seq
[ ]	[x]	Flow cytometry
[x]	[ ]	MRI-based neuroimaging

Eukaryotic cell lines

Policy information about cell lines

Cell line source(s)	HEK293A and Flp-In 293 were obtained from Thermo Fisher Scientific. Neuro2A was obtained from ATCC. HeLa, COS-7, and CHO-K1 cells was obtained from Cell-Based Assay Screening Service core (Baylor College of Medicine). The core purchased these cell lines from ATCC. HEK293T was a gift from Michael Lin Lab (Stanford University). Lin lab obtained the line from ATCC.
Authentication	All human cell lines were authenticated by the Cytogenetics and Cell Authentication core (MD Anderson). The core used the Short Tandem Repeat (STR) analysis for cell authentication.
Mycoplasma contamination	Mammalian cell cultures were tested free of mycoplasma contamination using the Mycoplasma Detection Kit (Lonza).
Commonly misidentified lines (See ICLAC register)	No commonly misidentified lines were used.

Flow Cytometry

Plots

Confirm that:

- [x] The axis labels state the marker and fluorochrome used (e.g. CD4-FITC).
- [x] The axis scales are clearly visible. Include numbers along axes only for bottom left plot of group (a 'group' is an analysis of identical markers).
- [x] All plots are contour plots with outliers or pseudocolor plots.
- [x] A numerical value for number of cells or percentage (with statistics) is provided.

Methodology

Sample preparation	Additional notes on the checklist above:<br>(1) This is not fully pertinent to our study. All our axes do describe the quantity being measured. We used fluorescent proteins (FPs) rather than dyes. To keep axes labeling consistent across the paper, we have not mentioned the FPs in the axes labels. However, the few FPs used are described in the text. All main figures except 1 (Fig. 5) used the same FPs. For Fig. 5, we have also indicated the FPs used in the caption.<br>(2) All axis scales are clearly visible. For panels with only 2-3 plots, we showed axes numbers for each plot as removing numbers caused confusion by readers who thought the numbers were missing. For panels with a greater number of plots, we have followed the suggested strategy.<br>(3) Most of the flow cytometry plots presented in our study are contour plots with outliers or pseudocolor plots. Histograms are used to present the distribution of a single variable.<br><br>Commonly used cell lines were transfected with plasmids using FuGene HD (Promega). 36-48 hrs after transfection, cells were detached and washed in DPBS. Detached cells were resuspended in DPBS and analyzed using FACS.
Instrument	Attune NxT Acoustic Focusing Cytometer (Thermo Fisher Scientific) was used for flow cytometry experiments.
Software	Attune NxT Acoustic Focusing Cytometer Software (version 4.0.1445.0 , Thermo Fisher Scientific) was used for data acquisition. Flow cytometry data was analyzed using FlowJo (version 10.6.1, FlowJo LLC), MATLAB (version r2019b, MathWorks), and Prism (version 9.0.2, GraphPad)
Cell population abundance	Only the transfected cells were used for analysis unless mentioned otherwise. We determined the transfected cells by using fluorescence protein (FP) markers that were spectrally compatible with fluorescent protein reporters of circuit expression. The transfection FP markers were expressed either by co-transfecting a separate FP-expressing plasmid or using an “onboard” FP expression cassette placed on the same plasmid as with the genetic circuits. We typically achieved 20% - 50% transfection efficiency which on average corresponded several thousand cells per well of a 96 microwell plate.
Gating strategy	The standard approach of using FSC-A and FSC-H was used to gate for single cells. Using the non-transfected cells as a reference for baseline fluorescence, we gated for and analyzed the cells that showed higher fluorescence protein reporter levels than the non-transfected cells. Example gating strategy is presented in the supplementary information for experiments that involved the quantification of the proportion of cells inside each gate.

- [x] Tick this box to confirm that a figure exemplifying the gating strategy is provided in the Supplementary Information.
