# Supplementary material for: A synthetic circuit for buffering gene dosage variation between individual mammalian cells
Source: Nat Commun. 2021 Jul 5;12:4132. doi: 10.1038/s41467-021-23889-0 (PMC8257781; doi:10.1038/s41467-021-23889-0)
Supplement: Supplementary file 3 — Source Data [file 41467_2021_23889_MOESM3_ESM.zip › Supplementary statistics.pdf]

Supplementary statistic for

A synthetic circuit for buffering gene dosage variation between individual mammalian cells

Table of contents

General comments ... 3

Fig. 3a ... 4

Fig. 3b ... 5

Fig. 3c ... 7

Cell-to-cell variability, CV ... 7

Relative circuit output ... 9

Fig. 3d ... 10

Fig. 3f ... 12

Fig. 3g ... 15

Fig. 4a ... 18

Fig. 4b & c ... 19

Fig. 5f ... 19

Fig. 5g ... 26

Fig. 5h ... 19

Fig 6a. ... 29

Fig 6c. ... 31

Supp. Fig. 3c ... 33

Supp. Fig. 7a ... 34

Supp. Fig. 7b ... 37

Supp. Fig. 8b ... 34

Supp. Fig. 13 ... 37

Supp. Fig. 14c ... 38

Supp. Fig. 14c - continued ... 38

Supp. Fig. 14d ... 39

Supp. Fig. 14e ... 39

Supp. Fig. 14f ... 42

Supp. Fig. 14g 46
Supp. Fig. 14h 47
Supp. Fig. 14i 48
Supp. Fig. 14j 49
Supp. Fig. 14j - continued 50
Supp. Fig. 15b 52
Supp. Fig. 20a 53
Supp. Fig. 20b 55
Supp. Fig. 20c 57
Supp. Fig. 20d 59
Supp. Fig. 20e 61
Supp. Fig. 22a 63
Supp. Fig. 22b 64

General comments

For each experiment, we have indicated the sample size (n), the type(s) of statistical analysis performed, and their corresponding results. For all statistical analyses, a significance level (α) of 0.05 was used. CV stands for coefficient of variation. NF stands for negative feedback. IFF stands for incoherent feedforward. Normality tests were not conducted because they have low power when the sample size is small.

Fig. 3a

Sample size (independent transfections per dox. concentration)

	Equalizer-H	Equalizer-M	Equalizer-L
**n**	3	3	8

Normality test

Normality was assumed.

Spearman's test for heteroscedasticity

Spearman's test for heteroscedasticity	
**Rs of predicted Y vs. |residual|**	-0.2248
**P value (one tailed)**	0.0130
**Passed (P > 0.05)?**	No

Two-way ANOVA

Two-way ANOVA	Ordinary				
**Alpha**	0.05				
**Source of Variation**	% of total variation	P value	P value summary	Significant?	
**Interaction**	18.66	<0.0001	****	Yes	
**Doxycycline concentration**	22.20	<0.0001	****	Yes	
**Equalizer variant**	43.28	<0.0001	****	Yes	
**ANOVA table**	SS (Type III)	DF	MS	F (DFn, DFd)	P value
**Interaction**	10691	12	890.9	F (12, 77) = 6.281	P<0.0001
**Doxycycline concentration**	12721	6	2120	F (6, 77) = 14.95	P<0.0001
**Equalizer variant**	24803	2	12402	F (2, 77) = 87.43	P<0.0001
**Residual**	10922	77	141.8		
**Data summary**					
**Number of columns (Equalizer variant)**	3				
**Number of rows (Doxycycline concentration)**	7				
**Number of values**	98				

Post- hoc Tukey tests

Tukey's multiple comparisons test	Predicted (LS) mean diff.	95.00% CI of diff.	Below threshold?	Summary	Adjusted P Value
**Row 1 (0 ng/mL dox.)**					

**Equalizer-H vs. Equalizer-M**	69.00	45.76 to 92.24	Yes	****	<0.0001
**Equalizer-H vs. Equalizer-L**	94.42	75.15 to 113.7	Yes	****	<0.0001
**Equalizer-M vs. Equalizer-L**	25.42	6.152 to 44.69	Yes	**	0.0064
**Row 2 (1 ng/mL dox.)**					
**Equalizer-H vs. Equalizer-M**	44.83	21.59 to 68.07	Yes	****	<0.0001
**Equalizer-H vs. Equalizer-L**	61.93	42.66 to 81.20	Yes	****	<0.0001
**Equalizer-M vs. Equalizer-L**	17.10	-2.173 to 36.36	No	ns	0.0924
**Row 3 (3 ng/mL dox.)**					
**Equalizer-H vs. Equalizer-M**	21.80	-1.439 to 45.04	No	ns	0.0706
**Equalizer-H vs. Equalizer-L**	28.75	9.477 to 48.01	Yes	**	0.0018
**Equalizer-M vs. Equalizer-L**	6.946	-12.32 to 26.21	No	ns	0.6661
**Row 4 (5 ng/mL dox.)**					
**Equalizer-H vs. Equalizer-M**	21.30	-1.939 to 44.54	No	ns	0.0792
**Equalizer-H vs. Equalizer-L**	16.90	-2.373 to 36.16	No	ns	0.0974
**Equalizer-M vs. Equalizer-L**	-4.404	-23.67 to 14.86	No	ns	0.8487
**Row 5 (7 ng/mL dox.)**					
**Equalizer-H vs. Equalizer-M**	16.30	-6.939 to 39.54	No	ns	0.2208
**Equalizer-H vs. Equalizer-L**	25.92	6.652 to 45.19	Yes	**	0.0054
**Equalizer-M vs. Equalizer-L**	9.621	-9.648 to 28.89	No	ns	0.4608
**Row 6 (10 ng/mL dox.)**					
**Equalizer-H vs. Equalizer-M**	13.50	-9.739 to 36.74	No	ns	0.3520
**Equalizer-H vs. Equalizer-L**	17.60	-1.665 to 36.87	No	ns	0.0805
**Equalizer-M vs. Equalizer-L**	4.104	-15.16 to 23.37	No	ns	0.8672
**Row 7 (30 ng/mL dox.)**					
**Equalizer-H vs. Equalizer-M**	28.33	5.094 to 51.57	Yes	*	0.0128
**Equalizer-H vs. Equalizer-L**	36.38	17.11 to 55.65	Yes	****	<0.0001
**Equalizer-M vs. Equalizer-L**	8.050	-11.22 to 27.32	No	ns	0.5801

Fig. 3b

Sample size (independent transfections per dox. induction concentration)

	Equalizer-H	Equalizer-M	Equalizer-L
**n**	3	3	8

Normality test

Normality was assumed.

Spearman's test for heteroscedasticity

**Spearman's test for heteroscedasticity**	
**Rs of predicted Y vs. |residual|**	0.4081
**P value (one tailed)**	<0.0001
**Passed (P > 0.05)?**	No

Two-way ANOVA

**Two-way ANOVA**	**Ordinary**				
**Alpha**	0.05				
**Source of Variation**	% of total variation	P value	P value summary	Significant?	
**Interaction**	20.82	<0.0001	****	Yes	
**Doxycycline concentration**	38.93	<0.0001	****	Yes	
**Equalizer variant**	49.42	<0.0001	****	Yes	
**ANOVA table**	SS (Type III)	DF	MS	F (DFn, DFd)	P value
**Interaction**	446.9	12	37.24	F (12, 77) = 76.86	P<0.0001
**Doxycycline concentration**	835.5	6	139.3	F (6, 77) = 287.4	P<0.0001
**Equalizer variant**	1061	2	530.4	F (2, 77) = 1095	P<0.0001
**Residual**	37.31	77	0.4845		
**Data summary**					
**Number of columns (Equalizer variant)**	3				
**Number of rows (Doxycycline concentration)**	7				
**Number of values**	98				

Post- hoc Tukey tests

**Tukey's multiple comparisons test**	**Predicted (LS) mean diff.**	**95.00% CI of diff.**	**Below threshold?**	**Summary**	**Adjusted P Value**
**Row 1 (0 ng/mL)**					
**Equalizer-H vs. Equalizer-M**	1.393	0.03476 to 2.751	Yes	*	0.0431
**Equalizer-H vs. Equalizer-L**	2.252	1.125 to 3.378	Yes	****	<0.0001

**Equalizer-M vs. Equalizer-L**	0.8587	-0.2675 to 1.985	No	ns	0.1692
**Row 2 (1 ng/mL)**					
**Equalizer-H vs. Equalizer-M**	2.857	1.499 to 4.216	Yes	****	<0.0001
**Equalizer-H vs. Equalizer-L**	3.884	2.758 to 5.010	Yes	****	<0.0001
**Equalizer-M vs. Equalizer-L**	1.027	-0.09951 to 2.153	No	ns	0.0813
**Row 3 (3 ng/mL)**					
**Equalizer-H vs. Equalizer-M**	3.734	2.376 to 5.093	Yes	****	<0.0001
**Equalizer-H vs. Equalizer-L**	5.674	4.547 to 6.800	Yes	****	<0.0001
**Equalizer-M vs. Equalizer-L**	1.939	0.8130 to 3.065	Yes	***	0.0003
**Row 4 (5 ng/mL)**					
**Equalizer-H vs. Equalizer-M**	4.761	3.402 to 6.119	Yes	****	<0.0001
**Equalizer-H vs. Equalizer-L**	7.313	6.187 to 8.439	Yes	****	<0.0001
**Equalizer-M vs. Equalizer-L**	2.552	1.426 to 3.678	Yes	****	<0.0001
**Row 5 (7 ng/mL)**					
**Equalizer-H vs. Equalizer-M**	5.554	4.196 to 6.912	Yes	****	<0.0001
**Equalizer-H vs. Equalizer-L**	8.116	6.990 to 9.242	Yes	****	<0.0001
**Equalizer-M vs. Equalizer-L**	2.562	1.436 to 3.688	Yes	****	<0.0001
**Row 6 (10 ng/mL)**					
**Equalizer-H vs. Equalizer-M**	8.153	6.795 to 9.511	Yes	****	<0.0001
**Equalizer-H vs. Equalizer-L**	11.42	10.29 to 12.54	Yes	****	<0.0001
**Equalizer-M vs. Equalizer-L**	3.264	2.138 to 4.390	Yes	****	<0.0001
**Row 7 (30 ng/mL)**					
**Equalizer-H vs. Equalizer-M**	15.26	13.90 to 16.62	Yes	****	<0.0001
**Equalizer-H vs. Equalizer-L**	19.68	18.55 to 20.81	Yes	****	<0.0001
**Equalizer-M vs. Equalizer-L**	4.424	3.297 to 5.550	Yes	****	<0.0001

Fig. 3c

Cell-to-cell variability, CV

Sample size (independent transfections or cell culture)

	Equalizer-H	Equalizer-M	Equalizer-L	CMV	PGK	UBC	CMV cell line
**n**	3	3	8	3	3	3	3

Normality test

Normality was assumed.

Brown-Forsythe test

Brown-Forsythe test	
F (DFn, DFd)	0.5049 (6, 19)
P value	0.7970
P value summary	ns
Are SDs significantly different (P < 0.05)?	No

One-way ANOVA

ANOVA summary	
F	249.2
P value	<0.0001
P value summary	****
Significant diff. among means (P < 0.05)?	Yes
R squared	0.9875

ANOVA table	SS	DF	MS	F (DFn, DFd)	P value
Treatment (between columns)	91779	6	15297	F (6, 19) = 249.2	P<0.0001
Residual (within columns)	1166	19	61.37		
Total	92945	25			
Data summary					
Number of treatments (columns)	7				
Number of values (total)	26				

Post- hoc Tukey tests

Tukey's multiple comparisons test	Mean Diff.	95.00% CI of diff.	Below threshold?	Summary	Adjusted P Value
Equalizer-H vs. Equalizer-M	22.50	1.491 to 43.51	Yes	*	0.0311
Equalizer-H vs. Equalizer-L	39.60	22.18 to 57.02	Yes	****	<0.0001
Equalizer-H vs. CMV	-27.00	-48.01 to -5.991	Yes	**	0.0070
Equalizer-H vs. PGK	-111.0	-132.0 to -89.99	Yes	****	<0.0001
Equalizer-H vs. UBC	-110.0	-131.0 to -88.99	Yes	****	<0.0001
Equalizer-H vs. CMV cell line	38.90	17.89 to 59.91	Yes	***	0.0001
Equalizer-M vs. Equalizer-L	17.10	-0.3243 to 34.52	No	ns	0.0565
Equalizer-M vs. CMV	-49.50	-70.51 to -28.49	Yes	****	<0.0001
Equalizer-M vs. PGK	-133.5	-154.5 to -112.5	Yes	****	<0.0001

**Equalizer-M vs. UBC**	-132.5	-153.5 to -111.5	Yes	****	<0.0001
**Equalizer-M vs. CMV cell line**	16.40	-4.609 to 37.41	No	ns	0.1921
**Equalizer-L vs. CMV**	-66.60	-84.02 to -49.18	Yes	****	<0.0001
**Equalizer-L vs. PGK**	-150.6	-168.0 to -133.2	Yes	****	<0.0001
**Equalizer-L vs. UBC**	-149.6	-167.0 to -132.2	Yes	****	<0.0001
**Equalizer-L vs. CMV cell line**	-0.6958	-18.12 to 16.72	No	ns	>0.9999
**CMV vs. PGK**	-84.00	-105.0 to -62.99	Yes	****	<0.0001
**CMV vs. UBC**	-83.00	-104.0 to -61.99	Yes	****	<0.0001
**CMV vs. CMV cell line**	65.90	44.89 to 86.91	Yes	****	<0.0001
**PGK vs. UBC**	1.000	-20.01 to 22.01	No	ns	>0.9999
**PGK vs. CMV cell line**	149.9	128.9 to 170.9	Yes	****	<0.0001
**UBC vs. CMV cell line**	148.9	127.9 to 169.9	Yes	****	<0.0001

Relative circuit output

Sample size (independent transfections or cell culture)

	**Equalizer-H**	**Equalizer-M**	**Equalizer-L**	**CMV**	**PGK**	**UBC**	**CMV cell line**
**n**	3	3	8	3	3	3	3

Normality test

Normality was assumed.

Brown-Forsythe test

**Brown-Forsythe test**	
**F (DFn, DFd)**	1.539 (6, 19)
**P value**	0.2191
**P value summary**	ns
**Are SDs significantly different (P < 0.05)?**	No

One-way ANOVA

**ANOVA summary**	
**F**	249.2
**P value**	<0.0001
**P value summary**	****
**Significant diff. among means (P < 0.05)?**	Yes
**R squared**	0.9875

ANOVA table	SS	DF	MS	F (DFn, DFd)	P value
Treatment (between columns)	19534	6	3256	F (6, 19) = 27150	P<0.0001
Residual (within columns)	2.278	19	0.1199		
Total	19536	25			
Data summary					
Number of treatments (columns)	7				
Number of values (total)	26				

Post- hoc Tukey tests

Tukey's multiple comparisons test	Mean Diff.	95.00% CI of diff.	Below threshold?	Summary	Adjusted P Value
Equalizer-H vs. Equalizer-M	7.671	6.742 to 8.599	Yes	****	<0.0001
Equalizer-H vs. Equalizer-L	9.621	8.851 to 10.39	Yes	****	<0.0001
Equalizer-H vs. CMV	-65.67	-66.60 to -64.74	Yes	****	<0.0001
Equalizer-H vs. PGK	9.062	8.134 to 9.991	Yes	****	<0.0001
Equalizer-H vs. UBC	3.928	2.999 to 4.857	Yes	****	<0.0001
Equalizer-H vs. CMV cell line	-48.18	-49.11 to -47.26	Yes	****	<0.0001
Equalizer-M vs. Equalizer-L	1.950	1.180 to 2.720	Yes	****	<0.0001
Equalizer-M vs. CMV	-73.34	-74.27 to -72.41	Yes	****	<0.0001
Equalizer-M vs. PGK	1.392	0.4631 to 2.320	Yes	**	0.0015
Equalizer-M vs. UBC	-3.743	-4.671 to -2.814	Yes	****	<0.0001
Equalizer-M vs. CMV cell line	-55.85	-56.78 to -54.93	Yes	****	<0.0001
Equalizer-L vs. CMV	-75.29	-76.06 to -74.52	Yes	****	<0.0001
Equalizer-L vs. PGK	-0.5587	-1.329 to 0.2113	No	ns	0.2582
Equalizer-L vs. UBC	-5.693	-6.463 to -4.923	Yes	****	<0.0001
Equalizer-L vs. CMV cell line	-57.81	-58.58 to -57.04	Yes	****	<0.0001
CMV vs. PGK	74.73	73.81 to 75.66	Yes	****	<0.0001
CMV vs. UBC	69.60	68.67 to 70.53	Yes	****	<0.0001
CMV vs. CMV cell line	17.49	16.56 to 18.42	Yes	****	<0.0001
PGK vs. UBC	-5.134	-6.063 to -4.206	Yes	****	<0.0001
PGK vs. CMV cell line	-57.25	-58.18 to -56.32	Yes	****	<0.0001
UBC vs. CMV cell line	-52.11	-53.04 to -51.18	Yes	****	<0.0001

Fig. 3d

Sample size (independent transfections)

	N2A	CHO-K1	COS7	HELA

**CMV**	6	6	6	6
**Equalizer-L**	6	6	6	6

Normality test

Normality was assumed.

Spearman's test for heteroscedasticity

**Spearman's test for heteroscedasticity**	
**Rs of predicted Y vs. |residual|**	0.2410
**P value (one tailed)**	0.0318
**Passed (P > 0.05)?**	No

Two-way ANOVA

**Two-way ANOVA**	**Ordinary**				
**Alpha**	0.05				
**Source of Variation**	% of total variation	P value	P value summary	Significant?	
**Interaction**	4.895	<0.0001	****	Yes	
**Row Factor**	9.170	<0.0001	****	Yes	
**Column Factor**	82.97	<0.0001	****	Yes	
**ANOVA table**	SS	DF	MS	F (DFn, DFd)	P value
**Interaction**	3738	4	934.4	F (4, 50) = 20.64	P<0.0001
**Row Factor**	7002	4	1750	F (4, 50) = 38.66	P<0.0001
**Column Factor**	63349	1	63349	F (1, 50) = 1399	P<0.0001
**Residual**	2264	50	45.28		
**Difference between column means**					
**Mean of Equalizer-L**	79.15				
**Mean of CMV plasmid**	144.1				
**Difference between means**	-64.99				
**SE of difference**	1.737				
**95% CI of difference**	-68.48 to -61.50				

Post- hoc Sidak tests

**Šídák's multiple comparisons test**	**Mean Diff.**	**95.00% CI of diff.**	**Below threshold?**	**Summary**	**Adjusted P Value**
**Equalizer-L - CMV plasmid**					
**HEK293**	-70.77	-81.14 to -60.39	Yes	****	<0.0001
**CHO-K1**	-76.38	-86.76 to -66.01	Yes	****	<0.0001

**N2A**	-59.97	-70.34 to -49.59	Yes	****	<0.0001
**COS-7**	-81.08	-91.46 to -70.71	Yes	****	<0.0001
**HeLa**	-36.73	-47.11 to -26.36	Yes	****	<0.0001

Fig. 3f

Sample size (independent transfections)

	CMV	PGK	Equalizer-L
**1 ng**	6	6	6
**5 ng**	6	6	6
**10 ng**	6	6	6
**50 ng**	6	6	6
**100 ng**	6	6	6
**200 ng**	6	6	6

Trendlines

Trendlines were determined after pooling all the data obtained at different transfection doses (n = 36 per circuit).

CMV

**Best-fit values**	
**Slope**	1.211
**Y-intercept**	137.0
**X-intercept**	-113.1
**1/slope**	0.8255
**Std. Error**	
**Slope**	0.2574
**Y-intercept**	3.580
**95% Confidence Intervals**	
**Slope**	0.6884 to 1.734
**Y-intercept**	129.7 to 144.3
**X-intercept**	-206.2 to -76.01
**Goodness of Fit**	
**R squared**	0.3946
**Sy.x**	16.13
**Is slope significantly non-zero?**	
**F**	22.16
**DFn, DFd**	1, 34
**P value**	<0.0001
**Deviation from zero?**	Significant
**Equation**	Y = 1.211*X + 137.0
**Data**	
**Number of X values**	36
**Maximum number of Y replicates**	1
**Total number of values**	36

**Number of missing values**	0

PGK

**One-phase association**	
**Best-fit values**	
**Y0**	132.5
**Plateau**	277.3
**K**	0.2905
**Tau**	3.443
**Half-time**	2.386
**Span**	144.9
**95% CI (profile likelihood)**	
**Y0**	54.67 to 179.2
**Plateau**	247.9 to 318.7
**K**	0.1076 to 0.6592
**Tau**	1.517 to 9.290
**Half-time**	1.051 to 6.439
**Goodness of Fit**	
**Degrees of Freedom**	33
**R squared**	0.4859
**Sum of Squares**	59087
**Sy.x**	42.31
**Constraints**	
**K**	K > 0
**Number of points**	
**# of X values**	36
**# Y values analyzed**	36

Equalizer-L

**Best-fit values**	
**Slope**	-0.2354
**Y-intercept**	77.29
**X-intercept**	328.3
**1/slope**	-4.248
**Std. Error**	
**Slope**	0.1745
**Y-intercept**	1.938
**95% Confidence Intervals**	
**Slope**	-0.5900 to 0.1192
**Y-intercept**	73.36 to 81.23
**X-intercept**	135.2 to +infinity
**Goodness of Fit**	
**R squared**	0.05082
**Sy.x**	8.869
**Is slope significantly non-zero?**	
**F**	1.821
**DFn, DFd**	1, 34
**P value**	0.1862
**Deviation from zero?**	Not Significant
**Equation**	Y = -0.2354*X + 77.29

Data	
Number of X values	36
Maximum number of Y replicates	1
Total number of values	36
Number of missing values	0

Normality test

Normality was assumed.

Spearman's test for heteroscedasticity

Spearman's test for heteroscedasticity	
Rs of predicted Y vs. |residual|	0.5254
P value (one tailed)	<0.0001
Passed (P > 0.05)?	No

Two-way ANOVA

Two-way ANOVA	Ordinary				
Alpha	0.05				
Source of Variation	% of total variation	P value	P value summary	Significant?	
Interaction	6.922	<0.0001	****	Yes	
Plasmid dose	4.331	0.0002	***	Yes	
Circuit type	74.20	<0.0001	****	Yes	
ANOVA table	SS	DF	MS	F (DFn, DFd)	P value
Interaction	35508	10	3551	F (10, 90) = 4.282	P<0.0001
Plasmid dose	22214	5	4443	F (5, 90) = 5.358	P=0.0002
Circuit type	380600	2	190300	F (2, 90) = 229.5	P<0.0001
Residual	74625	90	829.2		
Data summary					
Number of columns (Circuit type)	3				
Number of rows (Plasmid dose)	6				
Number of values	108				

Post- hoc Tukey tests

Tukey's multiple comparisons test	Mean Diff.	95.00% CI of diff.	Below threshold?	Summary	Adjusted P Value
Row 1 (1 ng)					
CMV vs. PGK	-43.92	-83.54 to -4.298	Yes	*	0.0261
CMV vs. Equalizer-L	44.08	4.464 to 83.70	Yes	*	0.0254
PGK vs. Equalizer-L	88.00	48.38 to 127.6	Yes	****	<0.0001

**Row 2 (5 ng)**					
**CMV vs. PGK**	-36.33	-75.95 to 3.286	No	ns	0.0792
**CMV vs. Equalizer-L**	67.05	27.43 to 106.7	Yes	***	0.0003
**PGK vs. Equalizer-L**	103.4	63.76 to 143.0	Yes	****	<0.0001
**Row 3 (10 ng)**					
**CMV vs. PGK**	-59.50	-99.12 to -19.88	Yes	**	0.0016
**CMV vs. Equalizer-L**	67.65	28.03 to 107.3	Yes	***	0.0003
**PGK vs. Equalizer-L**	127.2	87.53 to 166.8	Yes	****	<0.0001
**Row 4 (50 ng)**					
**CMV vs. PGK**	-92.17	-131.8 to -52.55	Yes	****	<0.0001
**CMV vs. Equalizer-L**	79.65	40.03 to 119.3	Yes	****	<0.0001
**PGK vs. Equalizer-L**	171.8	132.2 to 211.4	Yes	****	<0.0001
**Row 5 (100 ng)**					
**CMV vs. PGK**	-91.33	-131.0 to -51.71	Yes	****	<0.0001
**CMV vs. Equalizer-L**	76.83	37.21 to 116.5	Yes	****	<0.0001
**PGK vs. Equalizer-L**	168.2	128.5 to 207.8	Yes	****	<0.0001
**Row 6 (200 ng)**					
**CMV vs. PGK**	-114.2	-153.8 to -74.55	Yes	****	<0.0001
**CMV vs. Equalizer-L**	99.78	60.16 to 139.4	Yes	****	<0.0001
**PGK vs. Equalizer-L**	214.0	174.3 to 253.6	Yes	****	<0.0001

Fig. 3g

Sample size (independent transfections)

	**CMV**	**PGK**	**Equalizer-L**
**1 ng**	6	6	6
**5 ng**	6	6	6
**10 ng**	6	6	6
**50 ng**	6	6	6
**100 ng**	6	6	6
**200 ng**	6	6	6

Trendlines

Trendlines were determined after pooling all the data obtained at different transfection doses (n = 36 per circuit).

CMV

**One site -- Specific binding**	
**Best-fit values**	
**Bmax**	20.53
**Kd**	14.49
**95% CI (profile likelihood)**	
**Bmax**	18.45 to 23.05

**Kd**	11.61 to 18.24
**Goodness of Fit**	
**Degrees of Freedom**	34
**R squared**	0.9629
**Sum of Squares**	26.89
**Sy.x**	0.8893
**Number of points**	
**# of X values**	36
**# Y values analyzed**	36

PGK

**Best-fit values**	
**Slope**	0.7760
**Y-intercept**	0.2240
**X-intercept**	-0.2886
**1/slope**	1.289
**Std. Error**	
**Slope**	0.02189
**Y-intercept**	0.02189
**95% Confidence Intervals**	
**Slope**	0.7316 to 0.8205
**Y-intercept**	0.1795 to 0.2684
**X-intercept**	-0.9753 to 0.3612
**Goodness of Fit**	
**Sy.x**	1.243
**Is slope significantly non-zero?**	
**F**	1257
**DFn, DFd**	1, 35
**P value**	<0.0001
**Deviation from zero?**	Significant
**Equation**	Y = 0.7760*X + 0.2240
**Data**	
**Number of X values**	36
**Maximum number of Y replicates**	1
**Total number of values**	36
**Number of missing values**	0

Equalizer-L

**Best-fit values**	
**Slope**	0.08044
**Y-intercept**	0.9196
**X-intercept**	-11.43
**1/slope**	12.43
**Std. Error**	
**Slope**	0.006434
**Y-intercept**	0.006434
**95% Confidence Intervals**	
**Slope**	0.06738 to 0.09350
**Y-intercept**	0.9065 to 0.9326
**X-intercept**	-15.94 to -7.881

Goodness of Fit	
Sy.x	0.4050
Is slope significantly non-zero?	
F	156.3
DFn, DFd	1, 35
P value	<0.0001
Deviation from zero?	Significant
Equation	Y = 0.08044*X + 0.9196
Data	
Number of X values	36
Maximum number of Y replicates	1
Total number of values	36
Number of missing values	0

Normality test

Normality was assumed.

Spearman's test for heteroscedasticity

Spearman's test for heteroscedasticity	
Rs of predicted Y vs. |residual|	0.8991
P value (one tailed)	<0.0001
Passed (P > 0.05)?	No

Two-way ANOVA

Two-way ANOVA	Ordinary				
Alpha	0.05				
Source of Variation	% of total variation	P value	P value summary	Significant?	
Interaction	19.93	<0.0001	****	Yes	
Row Factor	41.87	<0.0001	****	Yes	
Column Factor	17.18	<0.0001	****	Yes	
ANOVA table	SS	DF	MS	F (DFn, DFd)	P value
Interaction	428.8	10	42.88	F (10, 90) = 8.539	P<0.0001
Row Factor	900.7	5	180.1	F (5, 90) = 35.87	P<0.0001
Column Factor	369.6	2	184.8	F (2, 90) = 36.80	P<0.0001
Residual	452.0	90	5.022		
Data summary					
Number of columns (Column Factor)	3				
Number of rows (Row Factor)	6				
Number of values	108				

Post- hoc Tukey tests

Tukey's multiple comparisons test	Mean Diff.	95.00% CI of diff.	Below threshold?	Summary	Adjusted P Value
**Row 1 (1 ng)**					
**CMV vs. PGK**	0.000	-3.083 to 3.083	No	ns	>0.9999
**CMV vs. Equalizer-L**	0.000	-3.083 to 3.083	No	ns	>0.9999
**PGK vs. Equalizer-L**	0.000	-3.083 to 3.083	No	ns	>0.9999
**Row 2 (5 ng)**					
**CMV vs. PGK**	1.269	-1.814 to 4.352	No	ns	0.5908
**CMV vs. Equalizer-L**	1.736	-1.347 to 4.819	No	ns	0.3760
**PGK vs. Equalizer-L**	0.4669	-2.616 to 3.550	No	ns	0.9308
**Row 3 (10 ng)**					
**CMV vs. PGK**	1.549	-1.534 to 4.633	No	ns	0.4577
**CMV vs. Equalizer-L**	2.677	-0.4068 to 5.760	No	ns	0.1022
**PGK vs. Equalizer-L**	1.127	-1.956 to 4.210	No	ns	0.6598
**Row 4 (50 ng)**					
**CMV vs. PGK**	2.984	-0.09919 to 6.067	No	ns	0.0600
**CMV vs. Equalizer-L**	5.682	2.599 to 8.766	Yes	****	<0.0001
**PGK vs. Equalizer-L**	2.698	-0.3849 to 5.782	No	ns	0.0985
**Row 5 (100 ng)**					
**CMV vs. PGK**	2.461	-0.6221 to 5.544	No	ns	0.1439
**CMV vs. Equalizer-L**	7.059	3.975 to 10.14	Yes	****	<0.0001
**PGK vs. Equalizer-L**	4.597	1.514 to 7.681	Yes	**	0.0017
**Row 6 (200 ng)**					
**CMV vs. PGK**	-5.312	-8.395 to -2.229	Yes	***	0.0003
**CMV vs. Equalizer-L**	7.729	4.646 to 10.81	Yes	****	<0.0001
**PGK vs. Equalizer-L**	13.04	9.958 to 16.12	Yes	****	<0.0001

Fig. 4a

Sample size (independent transfections)

	Equalizer-L (1 ng/mL)	NF (10 ng/mL)	IFF
**n**	3	3	3

Normality test

Normality was assumed.

Brown-Forsythe test

Brown-Forsythe test	
F (DFn, DFd)	0.8802 (2, 6)
P value	0.4622
P value summary	ns
Are SDs significantly different (P < 0.05)?	No

One-way ANOVA

ANOVA summary	
F	16.95
P value	0.0034
P value summary	**
Significant diff. among means (P < 0.05)?	Yes
R squared	0.8496

ANOVA table	SS	DF	MS	F (DFn, DFd)	P value
Treatment (between columns)	2556	2	1278	F (2, 6) = 16.95	P=0.0034
Residual (within columns)	452.4	6	75.40		
Total	3009	8			
Data summary					
Number of treatments (columns)	3				
Number of values (total)	9				

Post- hoc Tukey tests

Dunnett's multiple comparisons test	Mean Diff.	95.00% CI of diff.	Below threshold?	Summary	Adjusted P Value
Equalizer-L (1 ng/mL) vs. NF (10 ng/mL)	-35.07	-55.37 to -14.77	Yes	**	0.0047
Equalizer-L (1 ng/mL) vs. IFF	-36.40	-56.70 to -16.10	Yes	**	0.0039

Fig. 4b & c

Statistical analysis was not conducted.

Fig. 5f

Sample size (independent transfections)

	CMV	Equalizer-L	OLP	HYB
1 ng	6	6	6	6
5 ng	6	6	6	6
10 ng	6	6	6	6
50 ng	6	6	6	6
100 ng	6	6	6	6
200 ng	6	6	6	6

Trendlines

Trendlines were determined after pooling all the data obtained at different transfection doses (n = 36 per circuit).

CMV

**Best-fit values**	
**Slope**	-1.742
**Y-intercept**	120.9
**X-intercept**	69.38
**1/slope**	-0.5740
**Std. Error**	
**Slope**	0.1526
**Y-intercept**	1.433
**95% Confidence Intervals**	
**Slope**	-2.052 to -1.432
**Y-intercept**	118.0 to 123.8
**X-intercept**	59.89 to 82.97
**Goodness of Fit**	
**R squared**	0.7930
**Sy.x**	5.782
**Is slope significantly non-zero?**	
**F**	130.3
**DFn, DFd**	1, 34
**P value**	<0.0001
**Deviation from zero?**	Significant
**Equation**	Y = -1.742*X + 120.9
**Data**	
**Number of X values**	36
**Maximum number of Y replicates**	1
**Total number of values**	36
**Number of missing values**	0

Equalizer-L

**Best-fit values**	
**Slope**	0.1340
**Y-intercept**	63.53
**X-intercept**	-474.2
**1/slope**	7.464
**Std. Error**	
**Slope**	0.04810
**Y-intercept**	1.368
**95% Confidence Intervals**	
**Slope**	0.03622 to 0.2317
**Y-intercept**	60.75 to 66.31
**X-intercept**	-1804 to -266.0
**Goodness of Fit**	
**R squared**	0.1858
**Sy.x**	6.253
**Is slope significantly non-zero?**	
**F**	7.758
**DFn, DFd**	1, 34

**P value**	0.0087
**Deviation from zero?**	Significant
**Equation**	Y = 0.1340*X + 63.53
**Data**	
**Number of X values**	36
**Maximum number of Y replicates**	1
**Total number of values**	36
**Number of missing values**	0

OLP

**One phase decay**	
**Best-fit values**	
**Y0**	782.9
**Plateau**	88.97
**K**	0.9154
**Half Life**	0.7572
**Tau**	1.092
**Span**	693.9
**95% CI (profile likelihood)**	
**Y0**	614.0 to 1046
**Plateau**	68.82 to 107.1
**K**	0.6758 to 1.212
**Half Life**	0.5717 to 1.026
**Tau**	0.8248 to 1.480
**Goodness of Fit**	
**Degrees of Freedom**	33
**R squared**	0.9246
**Sum of Squares**	31578
**Sy.x**	30.93
**Constraints**	
**K**	K > 0
**Number of points**	
**# of X values**	36
**# Y values analyzed**	36

HYB

**One phase decay**	
**Best-fit values**	
**Y0**	585.1
**Plateau**	103.4
**K**	0.8607
**Half Life**	0.8053
**Tau**	1.162
**Span**	481.6
**95% CI (profile likelihood)**	
**Y0**	506.5 to 688.5
**Plateau**	93.27 to 112.9
**K**	0.7081 to 1.036
**Half Life**	0.6690 to 0.9789
**Tau**	0.9651 to 1.412

**Goodness of Fit**	
**Degrees of Freedom**	33
**R squared**	0.9636
**Sum of Squares**	7660
**Sy.x**	15.24
**Constraints**	
**K**	K > 0
**Number of points**	
**# of X values**	36
**# Y values analyzed**	36

Normality test

Normality was assumed.

Spearman's test for heteroscedasticity

**Spearman's test for heteroscedasticity**	
**Rs of predicted Y vs. |residual|**	0.5164
**P value (one tailed)**	<0.0001
**Passed (P > 0.05)?**	No

Two-way ANOVA

**Two-way ANOVA**	**Ordinary**				
**Alpha**	0.05				
**Source of Variation**	% of total variation	P value	P value summary	Significant?	
**Interaction**	29.95	<0.0001	****	Yes	
**Plasmid dose**	30.55	<0.0001	****	Yes	
**Circuit type**	37.09	<0.0001	****	Yes	
**ANOVA table**	SS	DF	MS	F (DFn, DFd)	P value
**Interaction**	302945	15	20196	F (15, 120) = 99.20	P<0.0001
**Plasmid dose**	309020	5	61804	F (5, 120) = 303.6	P<0.0001
**Circuit type**	375189	3	125063	F (3, 120) = 614.3	P<0.0001
**Residual**	24432	120	203.6		
**Data summary**					
**Number of columns (Circuit type)**	4				
**Number of rows (Plasmid dose)**	6				
**Number of values**	144				

Post- hoc Tukey tests

Tukey's multiple comparisons test	Mean Diff.	95.00% CI of diff.	Below threshold?	Summary	Adjusted P Value
**Row 1 (1 ng)**					
**TRE vs. HYB**	68.17	46.70 to 89.63	Yes	****	<0.0001
**TRE vs. CMV**	259.3	237.9 to 280.8	Yes	****	<0.0001
**TRE vs. Equalizer-L**	320.5	299.0 to 341.9	Yes	****	<0.0001
**HYB vs. CMV**	191.2	169.7 to 212.6	Yes	****	<0.0001
**HYB vs. Equalizer-L**	252.3	230.8 to 273.7	Yes	****	<0.0001
**CMV vs. Equalizer-L**	61.12	39.65 to 82.58	Yes	****	<0.0001
**Row 2 (5 ng)**					
**TRE vs. HYB**	22.67	1.203 to 44.13	Yes	*	0.0342
**TRE vs. CMV**	133.0	111.5 to 154.5	Yes	****	<0.0001
**TRE vs. Equalizer-L**	192.5	171.1 to 214.0	Yes	****	<0.0001
**HYB vs. CMV**	110.3	88.87 to 131.8	Yes	****	<0.0001
**HYB vs. Equalizer-L**	169.9	148.4 to 191.3	Yes	****	<0.0001
**CMV vs. Equalizer-L**	59.52	38.05 to 80.98	Yes	****	<0.0001
**Row 3 (10 ng)**					
**TRE vs. HYB**	9.333	-12.13 to 30.80	No	ns	0.6700
**TRE vs. CMV**	100.5	79.04 to 122.0	Yes	****	<0.0001
**TRE vs. Equalizer-L**	147.8	126.3 to 169.3	Yes	****	<0.0001
**HYB vs. CMV**	91.17	69.70 to 112.6	Yes	****	<0.0001
**HYB vs. Equalizer-L**	138.5	117.0 to 159.9	Yes	****	<0.0001
**CMV vs. Equalizer-L**	47.30	25.84 to 68.76	Yes	****	<0.0001
**Row 4 (50 ng)**					
**TRE vs. HYB**	-14.67	-36.13 to 6.797	No	ns	0.2878
**TRE vs. CMV**	2.767	-18.70 to 24.23	No	ns	0.9869
**TRE vs. Equalizer-L**	38.18	16.72 to 59.65	Yes	****	<0.0001
**HYB vs. CMV**	17.43	-4.030 to 38.90	No	ns	0.1539
**HYB vs. Equalizer-L**	52.85	31.39 to 74.31	Yes	****	<0.0001
**CMV vs. Equalizer-L**	35.42	13.95 to 56.88	Yes	***	0.0002
**Row 5 (100 ng)**					
**TRE vs. HYB**	-17.57	-39.03 to 3.897	No	ns	0.1488
**TRE vs. CMV**	-3.317	-24.78 to 18.15	No	ns	0.9778
**TRE vs. Equalizer-L**	25.12	3.653 to 46.58	Yes	*	0.0148
**HYB vs. CMV**	14.25	-7.214 to 35.71	No	ns	0.3129
**HYB vs. Equalizer-L**	42.68	21.22 to 64.15	Yes	****	<0.0001
**CMV vs. Equalizer-L**	28.43	6.970 to 49.90	Yes	**	0.0042
**Row 6 (200 ng)**					
**TRE vs. HYB**	-16.73	-38.20 to 4.730	No	ns	0.1824
**TRE vs. CMV**	-11.12	-32.58 to 10.35	No	ns	0.5335
**TRE vs. Equalizer-L**	13.77	-7.697 to 35.23	No	ns	0.3435
**HYB vs. CMV**	5.617	-15.85 to 27.08	No	ns	0.9038
**HYB vs. Equalizer-L**	30.50	9.036 to 51.96	Yes	**	0.0018

**CMV vs. Equalizer-L**	24.88	3.420 to 46.35	Yes	*	0.0161

Fig. 5g

Sample size (independent transfections)

	CMV	Equalizer-L
**1 ng**	6	6
**5 ng**	6	6
**10 ng**	6	6
**50 ng**	6	6
**100 ng**	6	6
**200 ng**	6	6

Trendlines

Trendlines were determined after pooling all the data obtained at different transfection doses (n = 36 per circuit). Slopes of Equalizer-L and HYB are compared in the next section.

CMV

Hyperbola (X is concentration)	
**Best-fit values**	
**Bmax**	7.045
**Kd**	5.015
**95% CI (profile likelihood)**	
**Bmax**	6.587 to 7.570
**Kd**	4.194 to 6.031
**Goodness of Fit**	
**Degrees of Freedom**	34
**R squared**	0.9659
**Sum of Squares**	3.475
**Sy.x**	0.3197
**Number of points**	
**# of X values**	36
**# Y values analyzed**	36

Equalizer-L

Best-fit values	
**Slope**	0.003320
**Y-intercept**	0.9967
**X-intercept**	-300.2
**1/slope**	301.2
**Std. Error**	
**Slope**	0.0004330
**Y-intercept**	0.0004330
**95% Confidence Intervals**	
**Slope**	0.002441 to 0.004199

**Y-intercept**	0.9958 to 0.9976
**X-intercept**	-440.5 to -207.8
**Goodness of Fit**	
**Sy.x**	0.07223
**Is slope significantly non-zero?**	
**F**	58.79
**DFn, DFd**	1, 35
**P value**	<0.0001
**Deviation from zero?**	Significant
**Equation**	Y = 0.003320*X + 0.9967
**Data**	
**Number of X values**	36
**Maximum number of Y replicates**	1
**Total number of values**	36
**Number of missing values**	0

Normality test

Normality was assumed.

Spearman's test for heteroscedasticity

**Spearman's test for heteroscedasticity**	
**Rs of predicted Y vs. |residual|**	0.6708
**P value (one tailed)**	<0.0001
**Passed (P > 0.05)?**	No

Two-way ANOVA

**Two-way ANOVA**	**Ordinary**				
**Alpha**	0.05				
**Source of Variation**	% of total variation	P value	P value summary	Significant?	
**Interaction**	21.55	<0.0001	****	Yes	
**Plasmid dose**	26.58	<0.0001	****	Yes	
**Circuit type**	44.70	<0.0001	****	Yes	
**ANOVA table**	SS	DF	MS	F (DFn, DFd)	P value
**Interaction**	44.03	5	8.806	F (5, 60) = 36.10	P<0.0001
**Plasmid dose**	54.31	5	10.86	F (5, 60) = 44.52	P<0.0001
**Circuit type**	91.31	1	91.31	F (1, 60) = 374.3	P<0.0001
**Residual**	14.64	60	0.2440		
**Data summary**					
**Number of columns (Circuit type)**	2				
**Number of rows (Plasmid dose)**	6				

Number of values	72				

Post- hoc Sidak tests

Šídák's multiple comparisons test	Mean Diff.	95.00% CI of diff.	Below threshold?	Summary	Adjusted P Value
CMV - Equalizer-L					
Row 1 (1 ng)	6.661e-016	-0.7758 to 0.7758	No	ns	>0.9999
Row 2 (5 ng)	0.8054	0.02960 to 1.581	Yes	*	0.0379
Row 3 (10 ng)	1.563	0.7876 to 2.339	Yes	****	<0.0001
Row 4 (50 ng)	3.131	2.355 to 3.907	Yes	****	<0.0001
Row 5 (100 ng)	3.775	2.999 to 4.551	Yes	****	<0.0001
Row 6 (200 ng)	4.239	3.463 to 5.014	Yes	****	<0.0001

Fig. 5h

Sample size (independent transfections)

	OLP	HYB
1 ng	6	6
5 ng	6	6
10 ng	6	6
50 ng	6	6
100 ng	6	6
200 ng	6	6

Trendlines

Trendlines were determined after pooling all the data obtained at different transfection doses (n = 36 per circuit).

OLP

Best-fit values	
Slope	2.093
Y-intercept	-1.093
X-intercept	0.5223
1/slope	0.4777
Std. Error	
Slope	0.04493
Y-intercept	0.04493
95% Confidence Intervals	
Slope	2.002 to 2.185
Y-intercept	-1.185 to -1.002
X-intercept	0.3097 to 0.7268
Goodness of Fit	
Sy.x	0.9955
Is slope significantly non-zero?	

**F**	2171
**DFn, DFd**	1, 35
**P value**	<0.0001
**Deviation from zero?**	Significant
**Equation**	Y = 2.093*X - 1.093
**Data**	
**Number of X values**	36
**Maximum number of Y replicates**	1
**Total number of values**	36
**Number of missing values**	0

HYB

**Best-fit values**	
**Slope**	0.9216
**Y-intercept**	0.07841
**X-intercept**	-0.08508
**1/slope**	1.085
**Std. Error**	
**Slope**	0.02416
**Y-intercept**	0.02416
**95% Confidence Intervals**	
**Slope**	0.8725 to 0.9706
**Y-intercept**	0.02937 to 0.1275
**X-intercept**	-0.3737 to 0.1883
**Goodness of Fit**	
**Sy.x**	0.5375
**Is slope significantly non-zero?**	
**F**	1455
**DFn, DFd**	1, 35
**P value**	<0.0001
**Deviation from zero?**	Significant
**Equation**	Y = 0.9216*X + 0.07841
**Data**	
**Number of X values**	36
**Maximum number of Y replicates**	1
**Total number of values**	36
**Number of missing values**	0

Unpaired t-test to compare the trendline slopes of Equalizer-L and HYB

**Unpaired t test with Welch's correction**	
**P value**	<0.0001
**P value summary**	****
**Significantly different (P < 0.05)?**	Yes
**One- or two-tailed P value?**	Two-tailed
**Welch-corrected t, df**	t=38.00, df=5.003
**How big is the difference?**	
**Mean of column A**	0.003320
**Mean of column B**	0.9216
**Difference between means (B - A) ± SEM**	0.9183 ± 0.02416
**95% confidence interval**	0.8562 to 0.9804

**R squared (eta squared)**	0.9965
**F test to compare variances**	
**F, DFn, Dfd**	3113, 5, 5
**P value**	<0.0001
**P value summary**	****
**Significantly different (P < 0.05)?**	Yes
**Data analyzed**	
**Sample size, column A**	6
**Sample size, column B**	6

Normality test

Normality was assumed.

Spearman's test for heteroscedasticity

**Spearman's test for heteroscedasticity**	
**Rs of predicted Y vs. |residual|**	0.4203
**P value (one tailed)**	0.0001
**Passed (P > 0.05)?**	No

Two-way ANOVA

**Two-way ANOVA**	**Ordinary**				
**Alpha**	0.05				
**Source of Variation**	% of total variation	P value	P value summary	Significant?	
**Interaction**	11.94	<0.0001	****	Yes	
**Plasmid dose**	69.67	<0.0001	****	Yes	
**Circuit type**	15.44	<0.0001	****	Yes	
**ANOVA table**	SS	DF	MS	F (DFn, DFd)	P value
**Interaction**	121.5	5	24.31	F (5, 60) = 48.48	P<0.0001
**Plasmid dose**	709.5	5	141.9	F (5, 60) = 283.0	P<0.0001
**Circuit type**	157.2	1	157.2	F (1, 60) = 313.5	P<0.0001
**Residual**	30.09	60	0.5014		
**Data summary**					
**Number of columns (Circuit type)**	2				
**Number of rows (Plasmid dose)**	6				
**Number of values**	72				

Post- hoc Sidak tests

**Šídák's multiple comparisons test**	**Mean Diff.**	**95.00% CI of diff.**	**Below threshold?**	**Summary**	**Adjusted P Value**

OLP - HYB					
**Row 1 (1 ng)**	-1.998e-015	-1.112 to 1.112	No	ns	>0.9999
**Row 2 (5 ng)**	0.3694	-0.7429 to 1.482	No	ns	0.9374
**Row 3 (10 ng)**	0.8717	-0.2406 to 1.984	No	ns	0.2029
**Row 4 (50 ng)**	4.665	3.553 to 5.778	Yes	****	<0.0001
**Row 5 (100 ng)**	5.498	4.386 to 6.610	Yes	****	<0.0001
**Row 6 (200 ng)**	6.326	5.214 to 7.438	Yes	****	<0.0001

Fig 6a.

Sample size (independent transfections or cell culture)

	CMV episome	PGK episome	Equalizer-L episome	CMV cell line
**n**	4	4	4	4

Normality test

Normality was assumed.

Spearman's test for heteroscedasticity

Spearman's test for heteroscedasticity	
**Rs of predicted Y vs. |residual|**	0.6458
**P value (one tailed)**	<0.0001
**Passed (P > 0.05)?**	No

Two-way ANOVA

Two-way ANOVA	Ordinary				
**Alpha**	0.05				
**Source of Variation**	% of total variation	P value	P value summary	Significant?	
**Interaction**	13.44	<0.0001	****	Yes	
**Row Factor**	7.152	<0.0001	****	Yes	
**Column Factor**	71.64	<0.0001	****	Yes	
**ANOVA table**	SS	DF	MS	F (DFn, DFd)	P value
**Interaction**	33281	12	2773	F (12, 60) = 8.655	P<0.0001
**Row Factor**	17706	4	4426	F (4, 60) = 13.81	P<0.0001
**Column Factor**	177363	3	59121	F (3, 60) = 184.5	P<0.0001
**Residual**	19225	60	320.4		

Data summary					
Number of columns (Column Factor)	4				
Number of rows (Row Factor)	5				
Number of values	80				

Post- hoc Tukey tests

Tukey's multiple comparisons test	Mean Diff.	95.00% CI of diff.	Below threshold?	Summary	Adjusted P Value
**Row 1 (Day 9)**					
**CMV episome vs. PGK episome**	-36.75	-70.20 to -3.302	Yes	*	0.0258
**CMV episome vs. Equalizer-L episome**	131.8	98.33 to 165.2	Yes	****	<0.0001
**CMV episome vs. CMV cell line**	130.6	97.15 to 164.0	Yes	****	<0.0001
**PGK episome vs. Equalizer-L episome**	168.5	135.1 to 202.0	Yes	****	<0.0001
**PGK episome vs. CMV cell line**	167.4	133.9 to 200.8	Yes	****	<0.0001
**Equalizer-L episome vs. CMV cell line**	-1.175	-34.62 to 32.27	No	ns	0.9997
**Row 2 (Day 16)**					
**CMV episome vs. PGK episome**	-18.88	-52.32 to 14.57	No	ns	0.4491
**CMV episome vs. Equalizer-L episome**	106.1	72.69 to 139.6	Yes	****	<0.0001
**CMV episome vs. CMV cell line**	106.6	73.18 to 140.1	Yes	****	<0.0001
**PGK episome vs. Equalizer-L episome**	125.0	91.56 to 158.5	Yes	****	<0.0001
**PGK episome vs. CMV cell line**	125.5	92.05 to 158.9	Yes	****	<0.0001
**Equalizer-L episome vs. CMV cell line**	0.4875	-32.96 to 33.94	No	ns	>0.9999
**Row 3 (Day 23)**					
**CMV episome vs. PGK episome**	-67.51	-101.0 to -34.06	Yes	****	<0.0001
**CMV episome vs. Equalizer-L episome**	51.54	18.09 to 84.99	Yes	***	0.0008
**CMV episome vs. CMV cell line**	53.13	19.68 to 86.57	Yes	***	0.0005
**PGK episome vs. Equalizer-L episome**	119.1	85.60 to 152.5	Yes	****	<0.0001
**PGK episome vs. CMV cell line**	120.6	87.19 to 154.1	Yes	****	<0.0001
**Equalizer-L episome vs. CMV cell line**	1.588	-31.86 to 35.04	No	ns	0.9993
**Row 4 (Day 40)**					
**CMV episome vs. PGK episome**	-33.95	-67.40 to -0.5023	Yes	*	0.0454

**CMV episome vs. Equalizer-L episome**	49.41	15.96 to 82.86	Yes	**	0.0014
**CMV episome vs. CMV cell line**	41.88	8.427 to 75.32	Yes	**	0.0084
**PGK episome vs. Equalizer-L episome**	83.36	49.91 to 116.8	Yes	****	<0.0001
**PGK episome vs. CMV cell line**	75.83	42.38 to 109.3	Yes	****	<0.0001
**Equalizer-L episome vs. CMV cell line**	-7.538	-40.99 to 25.91	No	ns	0.9331
**Row 5 (Day 60)**					
**CMV episome vs. PGK episome**	-10.88	-44.32 to 22.57	No	ns	0.8257
**CMV episome vs. Equalizer-L episome**	39.95	6.502 to 73.40	Yes	*	0.0130
**CMV episome vs. CMV cell line**	32.03	-1.423 to 65.47	No	ns	0.0654
**PGK episome vs. Equalizer-L episome**	50.83	17.38 to 84.27	Yes	***	0.0009
**PGK episome vs. CMV cell line**	42.90	9.452 to 76.35	Yes	**	0.0066
**Equalizer-L episome vs. CMV cell line**	-7.925	-41.37 to 25.52	No	ns	0.9233

Fig 6c.

Sample size (independent transfections or culture)

	**CMV episome**	**PGK episome**	**Equalizer-L episome**	**CMV cell line**
**n**	4	4	4	4

Normality test

Normality was assumed.

Spearman's test for heteroscedasticity

**Spearman's test for heteroscedasticity**	
**Rs of predicted Y vs. |residual|**	0.09763
**P value (one tailed)**	0.1945
**Passed (P > 0.05)?**	Yes

Two-way ANOVA

**Two-way ANOVA**	**Ordinary**				
**Alpha**	0.05				
**Source of Variation**	% of total variation	P value	P value summary	Significant?	
**Interaction**	14.89	<0.0001	****	Yes	
**Row Factor**	16.48	<0.0001	****	Yes	

**Column Factor**	52.25	<0.0001	****	Yes	

**ANOVA table**	SS	DF	MS	F (DFn, DFd)	P value
**Interaction**	1.766	12	0.1472	F (12, 60) = 4.544	P<0.0001
**Row Factor**	1.955	4	0.4889	F (4, 60) = 15.09	P<0.0001
**Column Factor**	6.198	3	2.066	F (3, 60) = 63.79	P<0.0001
**Residual**	1.943	60	0.03239		

Post- hoc Tukey tests

**Tukey's multiple comparisons test**	**Mean Diff.**	**95.00% CI of diff.**	**Below threshold?**	**Summary**	**Adjusted P Value**
**Row 1 (Day 9)**					
**CMVepisome vs. PGKepisome**	0.000	-0.3363 to 0.3363	No	ns	>0.9999
**CMVepisome vs. Equalizer-Lepisome**	0.000	-0.3363 to 0.3363	No	ns	>0.9999
**CMVepisome vs. CMVcellline**	0.000	-0.3363 to 0.3363	No	ns	>0.9999
**PGKepisome vs. Equalizer-Lepisome**	0.000	-0.3363 to 0.3363	No	ns	>0.9999
**PGKepisome vs. CMVcellline**	0.000	-0.3363 to 0.3363	No	ns	>0.9999
**Equalizer-Lepisome vs. CMVcellline**	0.000	-0.3363 to 0.3363	No	ns	>0.9999
**Row 2 (Day 16)**					
**CMVepisome vs. PGKepisome**	-0.2600	-0.5962 to 0.07629	No	ns	0.1840
**CMVepisome vs. Equalizer-Lepisome**	-0.9350	-1.271 to -0.5987	Yes	****	<0.0001
**CMVepisome vs. CMVcellline**	-0.7701	-1.106 to -0.4338	Yes	****	<0.0001
**PGKepisome vs. Equalizer-Lepisome**	-0.6750	-1.011 to -0.3387	Yes	****	<0.0001
**PGKepisome vs. CMVcellline**	-0.5101	-0.8464 to -0.1739	Yes	***	0.0010
**Equalizer-Lepisome vs. CMVcellline**	0.1649	-0.1714 to 0.5011	No	ns	0.5692
**Row 3 (Day 23)**					
**CMVepisome vs. PGKepisome**	-0.1694	-0.5057 to 0.1669	No	ns	0.5470
**CMVepisome vs. Equalizer-Lepisome**	-0.8450	-1.181 to -0.5087	Yes	****	<0.0001
**CMVepisome vs. CMVcellline**	-0.6765	-1.013 to -0.3402	Yes	****	<0.0001
**PGKepisome vs. Equalizer-Lepisome**	-0.6756	-1.012 to -0.3393	Yes	****	<0.0001
**PGKepisome vs. CMVcellline**	-0.5071	-0.8434 to -0.1708	Yes	**	0.0010

**Equalizer-Lepisome vs. CMVcellline**	0.1685	-0.1678 to 0.5047	No	ns	0.5515
**Row 4 (Day 30)**					
**CMVepisome vs. PGKepisome**	-0.1184	-0.4546 to 0.2179	No	ns	0.7888
**CMVepisome vs. Equalizer-Lepisome**	-0.8318	-1.168 to -0.4955	Yes	****	<0.0001
**CMVepisome vs. CMVcellline**	-0.6399	-0.9761 to -0.3036	Yes	****	<0.0001
**PGKepisome vs. Equalizer-Lepisome**	-0.7134	-1.050 to -0.3772	Yes	****	<0.0001
**PGKepisome vs. CMVcellline**	-0.5215	-0.8578 to -0.1852	Yes	***	0.0007
**Equalizer-Lepisome vs. CMVcellline**	0.1919	-0.1443 to 0.5282	No	ns	0.4391
**Row 5 (Day 60)**					
**CMVepisome vs. PGKepisome**	-0.1250	-0.4613 to 0.2113	No	ns	0.7600
**CMVepisome vs. Equalizer-Lepisome**	-0.6323	-0.9686 to -0.2960	Yes	****	<0.0001
**CMVepisome vs. CMVcellline**	-0.8042	-1.140 to -0.4680	Yes	****	<0.0001
**PGKepisome vs. Equalizer-Lepisome**	-0.5073	-0.8435 to -0.1710	Yes	**	0.0010
**PGKepisome vs. CMVcellline**	-0.6792	-1.015 to -0.3429	Yes	****	<0.0001
**Equalizer-Lepisome vs. CMVcellline**	-0.1719	-0.5082 to 0.1643	No	ns	0.5346

Supp. Fig. 3c

Sample size (independent transfections)

	**No induction**	**Saturated induction**
**n**	3	3

Normality test

Normality was assumed.

F-test

**F test to compare variances**	
**F, DFn, Dfd**	24.83, 2, 2
**P value**	0.0774
**P value summary**	ns
**Significantly different (P < 0.05)?**	No

Unpaired t-test

Unpaired t test	
P value	0.0001
P value summary	***
Significantly different (P < 0.05)?	Yes
One- or two-tailed P value?	Two-tailed
t, df	t=15.23, df=4

Supp. Fig. 7a

Sample size (independent transfections per transfection dose)

	CMV	PGK	Equalizer-L
n	6	6	6

Normality test

Normality was assumed.

Spearman's test for heteroscedasticity

Spearman's test for heteroscedasticity	
Rs of predicted Y vs. |residual|	0.9648
P value (one tailed)	<0.0001
Passed (P > 0.05)?	No

Two-way ANOVA

Two-way ANOVA	Ordinary				
Alpha	0.05				
Source of Variation	% of total variation	P value	P value summary	Significant?	
Interaction	1.449	0.9582	ns	No	
Plasmid dose	62.00	<0.0001	****	Yes	
Circuit type	0.9034	0.3243	ns	No	
ANOVA table	SS	DF	MS	F (DFn, DFd)	P value
Interaction	172022553	10	17202255	F (10, 90) = 0.3659	P=0.9582
Plasmid dose	7359321248	5	1471864250	F (5, 90) = 31.30	P<0.0001
Circuit type	107232430	2	53616215	F (2, 90) = 1.140	P=0.3243
Residual	4231530864	90	47017010		
Data summary					
Number of columns (Circuit type)	3				
Number of rows (Plasmid dose)	6				
Number of values	108				

Post- hoc Tukey tests

Tukey's multiple comparisons test	Mean Diff.	95.00% CI of diff.	Below threshold?	Summary	Adjusted P Value
**Row 1 (1 ng)**					
**CMV vs. PGK**	-79.00	-9513 to 9355	No	ns	0.9998
**CMV vs. Equalizer-L**	-165.0	-9599 to 9269	No	ns	0.9990
**PGK vs. Equalizer-L**	-86.00	-9520 to 9348	No	ns	0.9997
**Row 2 (5 ng)**					
**CMV vs. PGK**	631.7	-8803 to 10066	No	ns	0.9861
**CMV vs. Equalizer-L**	448.8	-8985 to 9883	No	ns	0.9929
**PGK vs. Equalizer-L**	-182.8	-9617 to 9251	No	ns	0.9988
**Row 3 (10 ng)**					
**CMV vs. PGK**	954.7	-8480 to 10389	No	ns	0.9685
**CMV vs. Equalizer-L**	606.2	-8828 to 10040	No	ns	0.9872
**PGK vs. Equalizer-L**	-348.5	-9783 to 9086	No	ns	0.9957
**Row 4 (50 ng)**					
**CMV vs. PGK**	3804	-5630 to 13238	No	ns	0.6034
**CMV vs. Equalizer-L**	1429	-8006 to 10863	No	ns	0.9308
**PGK vs. Equalizer-L**	-2375	-11810 to 7059	No	ns	0.8205
**Row 5 (100 ng)**					
**CMV vs. PGK**	5482	-3953 to 14916	No	ns	0.3532
**CMV vs. Equalizer-L**	369.0	-9065 to 9803	No	ns	0.9952
**PGK vs. Equalizer-L**	-5113	-14547 to 4322	No	ns	0.4037
**Row 6 (200 ng)**					
**CMV vs. PGK**	447.2	-8987 to 9881	No	ns	0.9930
**CMV vs. Equalizer-L**	-5198	-14632 to 4237	No	ns	0.3917
**PGK vs. Equalizer-L**	-5645	-15079 to 3789	No	ns	0.3320

Supp. Fig. 7b

Sample size (independent transfections per dose)

	CMV	PGK	Equalizer-L
**n**	6	6	6

Normality test

Normality was assumed.

Spearman's test for heteroscedasticity

Spearman's test for heteroscedasticity	
**Rs of predicted Y vs. |residual|**	0.9633

**P value (one tailed)**	<0.0001
**Passed (P > 0.05)?**	No

Two-way ANOVA

**Two-way ANOVA**	**Ordinary**				
**Alpha**	0.05				
**Source of Variation**	% of total variation	P value	P value summary	Significant?	
**Interaction**	19.79	<0.0001	****	Yes	
**Plasmid dose**	11.48	<0.0001	****	Yes	
**Circuit type**	57.98	<0.0001	****	Yes	
**ANOVA table**	SS	DF	MS	F (DFn, DFd)	P value
**Interaction**	6240406186	10	624040619	F (10, 90) = 16.59	P<0.0001
**Plasmid dose**	3620390594	5	724078119	F (5, 90) = 19.24	P<0.0001
**Circuit type**	18280470365	2	9140235183	F (2, 90) = 242.9	P<0.0001
**Residual**	3386189801	90	37624331		
**Data summary**					
**Number of columns (Circuit type)**	3				
**Number of rows (Plasmid dose)**	6				
**Number of values**	108				

Post- hoc Tukey tests

**Tukey's multiple comparisons test**	**Mean Diff.**	**95.00% CI of diff.**	**Below threshold?**	**Summary**	**Adjusted P Value**
**Row 1 (1 ng)**					
**CMV vs. PGK**	4770	-3669 to 13210	No	ns	0.3732
**CMV vs. Equalizer-L**	4202	-4238 to 12641	No	ns	0.4643
**PGK vs. Equalizer-L**	-568.7	-9008 to 7871	No	ns	0.9859
**Row 2 (5 ng)**					
**CMV vs. PGK**	14392	5953 to 22831	Yes	***	0.0003
**CMV vs. Equalizer-L**	13730	5291 to 22170	Yes	***	0.0006
**PGK vs. Equalizer-L**	-661.7	-9101 to 7778	No	ns	0.9809
**Row 3 (10 ng)**					
**CMV vs. PGK**	19477	11038 to 27917	Yes	****	<0.0001
**CMV vs. Equalizer-L**	18819	10380 to 27259	Yes	****	<0.0001
**PGK vs. Equalizer-L**	-658.2	-9098 to 7781	No	ns	0.9811
**Row 4 (50 ng)**					

**CMV vs. PGK**	35840	27400 to 44279	Yes	****	<0.0001
**CMV vs. Equalizer-L**	35129	26689 to 43568	Yes	****	<0.0001
**PGK vs. Equalizer-L**	-711.2	-9151 to 7728	No	ns	0.9780
**Row 5 (100 ng)**					
**CMV vs. PGK**	43573	35134 to 52013	Yes	****	<0.0001
**CMV vs. Equalizer-L**	42921	34482 to 51360	Yes	****	<0.0001
**PGK vs. Equalizer-L**	-652.2	-9092 to 7787	No	ns	0.9815
**Row 6 (200 ng)**					
**CMV vs. PGK**	49204	40765 to 57644	Yes	****	<0.0001
**CMV vs. Equalizer-L**	49074	40635 to 57514	Yes	****	<0.0001
**PGK vs. Equalizer-L**	-130.0	-8569 to 8309	No	ns	0.9993

Supp. Fig. 8b

Sample size (independent transfections)

	CMV	PGK	Equalizer-L
**n**	3	3	3

Area under the curve (AUC) for each circuit was determined using data values between 1 to 100 normalized gene-dosage marker values. ANOVA was conducted using the mean AUC values.

One-way ANOVA

**ANOVA summary**					
**F**	24.63				
**P value**	0.0013				
**P value summary**	**				
**Significant diff. among means (P < 0.05)?**	Yes				
**R squared**	0.8914				
**ANOVA table**	SS	DF	MS	F (DFn, DFd)	P value
**Treatment (between columns)**	241382505	2	120691252	F (2, 6) = 24.63	P=0.0013
**Residual (within columns)**	29402522	6	4900420		
**Total**	270785027	8			
**Data summary**					
**Number of treatments (columns)**	3				
**Number of values (total)**	9				

Post-hoc Tukey tests

Tukey's multiple comparisons test	Mean Diff.	95.00% CI of diff.	Below threshold?	Summary	Adjusted P Value

**CMV vs. PGK**	-3916	-9462 to 1630	No	ns	0.1562
**CMV vs. Equalizer-L**	8491	2946 to 14037	Yes	**	0.0080
**PGK vs. Equalizer-L**	12407	6862 to 17953	Yes	**	0.0011

Supp. Fig. 13

Statistical analysis was not conducted.

Supp. Fig. 14c

Sample size (independent transfections)

	OLP	HYB
**n**	3	3

For each curve, the area-under-the-curve was calculated. Statistical analysis was conducted to compare the mean area-under-the-curve of the constructs.

Normality test

Normality was assumed.

F-test

F test to compare variances	
**F, DFn, Dfd**	2.296, 2, 2
**P value**	0.6069
**P value summary**	ns
**Significantly different (P < 0.05)?**	No

Unpaired t-test

Unpaired t test	
**P value**	<0.0001
**P value summary**	****
**Significantly different (P < 0.05)?**	Yes
**One- or two-tailed P value?**	Two-tailed
**t, df**	t=17.45, df=4

Supp. Fig. 14c - continued

Sample size (independent transfections)

	CMV	Equalizer-L
**n**	3	3

For each curve, the area-under-the-curve was calculated. Statistical analysis was conducted to compare the mean area-under-the-curve of the constructs.

Normality test

Normality was assumed.

F-test

F test to compare variances	
F, DFn, Dfd	3234, 2, 2
P value	0.0006
P value summary	***
Significantly different (P < 0.05)?	Yes

Unpaired t-test

Unpaired t test with Welch's correction	
P value	0.0100
P value summary	*
Significantly different (P < 0.05)?	Yes
One- or two-tailed P value?	Two-tailed
Welch-corrected t, df	t=9.903, df=2.001

Supp. Fig. 14d

Sample size (independent transfections)

	OLP	HYB
n	3	3

For each curve, the area-under-the-curve was calculated. Statistical analysis was conducted to compare the mean area-under-the-curve of the constructs.

Normality test

Normality was assumed.

F-test

F test to compare variances	
F, DFn, Dfd	8.380, 2, 2
P value	0.2132
P value summary	ns
Significantly different (P < 0.05)?	No

Unpaired t-test

Unpaired t test	
P value	<0.0001
P value summary	****
Significantly different (P < 0.05)?	Yes
One- or two-tailed P value?	Two-tailed
t, df	t=43.00, df=4

Supp. Fig. 14e

Sample size (independent transfections)

	OLP	HYB
1 ng	6	6
5 ng	6	6

**10 ng**	6	6
**50 ng**	6	6
**100 ng**	6	6
**200 ng**	6	6

Trendlines

Trendlines were determined after pooling all the data obtained at different transfection doses (n = 36 per circuit).

OLP

**Best-fit values**	
**Slope**	1.949
**Y-intercept**	-0.9485
**X-intercept**	0.4868
**1/slope**	0.5132
**Std. Error**	
**Slope**	0.06033
**Y-intercept**	0.06033
**95% Confidence Intervals**	
**Slope**	1.826 to 2.071
**Y-intercept**	-1.071 to -0.8260
**X-intercept**	0.3807 to 0.5887
**Goodness of Fit**	
**Sy.x**	0.5366
**Is slope significantly non-zero?**	
**F**	1043
**DFn, DFd**	1, 35
**P value**	<0.0001
**Deviation from zero?**	Significant
**Equation**	Y = 1.949*X - 0.9485
**Data**	
**Number of X values**	36
**Maximum number of Y replicates**	1
**Total number of values**	36
**Number of missing values**	0

HYB

**Best-fit values**	
**Slope**	0.7711
**Y-intercept**	0.2289
**X-intercept**	-0.2968
**1/slope**	1.297
**Std. Error**	
**Slope**	0.02506
**Y-intercept**	0.02506
**95% Confidence Intervals**	
**Slope**	0.7202 to 0.8220
**Y-intercept**	0.1780 to 0.2798
**X-intercept**	-0.4758 to -0.1305

**Goodness of Fit**	
**Sy.x**	0.2536
**Is slope significantly non-zero?**	
**F**	946.6
**DFn, DFd**	1, 35
**P value**	<0.0001
**Deviation from zero?**	Significant
**Equation**	Y = 0.7711*X + 0.2289
**Data**	
**Number of X values**	36
**Maximum number of Y replicates**	1
**Total number of values**	36
**Number of missing values**	0

Normality test

Normality was assumed.

Spearman's test for heteroscedasticity

**Spearman's test for heteroscedasticity**	
**Rs of predicted Y vs. |residual|**	0.09454
**P value (one tailed)**	0.2148
**Passed (P > 0.05)?**	Yes

Two-way ANOVA

**Two-way ANOVA**	**Ordinary**				
**Alpha**	0.05				
**Source of Variation**	% of total variation	P value	P value summary	Significant?	
**Interaction**	11.57	<0.0001	****	Yes	
**Plasmid dose**	67.82	<0.0001	****	Yes	
**Circuit type**	16.31	<0.0001	****	Yes	
**ANOVA table**	SS	DF	MS	F (DFn, DFd)	P value
**Interaction**	17.33	5	3.465	F (5, 60) = 32.33	P<0.0001
**Plasmid dose**	101.5	5	20.31	F (5, 60) = 189.4	P<0.0001
**Circuit type**	24.42	1	24.42	F (1, 60) = 227.8	P<0.0001
**Residual**	6.432	60	0.1072		
**Data summary**					
**Number of columns (Circuit type)**	2				
**Number of rows (Plasmid dose)**	6				
**Number of values**	72				

Post- hoc Tukey tests

Šídák's multiple comparisons test	Mean Diff.	95.00% CI of diff.	Below threshold?	Summary	Adjusted P Value
**OLP - HYB**					
**Row 1 (1 ng)**	0.000	-0.5143 to 0.5143	No	ns	>0.9999
**Row 2 (5 ng)**	0.2013	-0.3130 to 0.7156	No	ns	0.8731
**Row 3 (10 ng)**	0.4078	-0.1065 to 0.9221	No	ns	0.1924
**Row 4 (50 ng)**	1.856	1.341 to 2.370	Yes	****	<0.0001
**Row 5 (100 ng)**	2.130	1.616 to 2.644	Yes	****	<0.0001
**Row 6 (200 ng)**	2.393	1.879 to 2.907	Yes	****	<0.0001

Supp. Fig. 14f

Sample size (independent transfections)

	CMV	Equalizer-L	OLP	HYB
**1 ng**	6	6	6	6
**5 ng**	6	6	6	6
**10 ng**	6	6	6	6
**50 ng**	6	6	6	6
**100 ng**	6	6	6	6
**200 ng**	6	6	6	6

Trendlines

Trendlines were determined after pooling all the data obtained at different transfection doses (n = 36 per circuit).

CMV

Best-fit values	
**Slope**	-1.742
**Y-intercept**	120.9
**X-intercept**	69.38
**1/slope**	-0.5740
**Std. Error**	
**Slope**	0.1526
**Y-intercept**	1.433
**95% Confidence Intervals**	
**Slope**	-2.052 to -1.432
**Y-intercept**	118.0 to 123.8
**X-intercept**	59.89 to 82.97
**Goodness of Fit**	
**R squared**	0.7930
**Sy.x**	5.782
**Is slope significantly non-zero?**	

**F**	130.3
**DFn, DFd**	1, 34
**P value**	<0.0001
**Deviation from zero?**	Significant
**Equation**	Y = -1.742*X + 120.9
**Data**	
**Number of X values**	36
**Maximum number of Y replicates**	1
**Total number of values**	36
**Number of missing values**	0

Equalizer-L

**Best-fit values**	
**Slope**	0.1340
**Y-intercept**	63.53
**X-intercept**	-474.2
**1/slope**	7.464
**Std. Error**	
**Slope**	0.04810
**Y-intercept**	1.368
**95% Confidence Intervals**	
**Slope**	0.03622 to 0.2317
**Y-intercept**	60.75 to 66.31
**X-intercept**	-1804 to -266.0
**Goodness of Fit**	
**R squared**	0.1858
**Sy.x**	6.253
**Is slope significantly non-zero?**	
**F**	7.758
**DFn, DFd**	1, 34
**P value**	0.0087
**Deviation from zero?**	Significant
**Equation**	Y = 0.1340*X + 63.53
**Data**	
**Number of X values**	36
**Maximum number of Y replicates**	1
**Total number of values**	36
**Number of missing values**	0

OLP

**One phase decay**	
**Best-fit values**	
**Y0**	1086
**Plateau**	82.14
**K**	1.926
**Half Life**	0.3599
**Tau**	0.5192
**Span**	1004
**95% CI (profile likelihood)**	
**Y0**	658.6 to 2072
**Plateau**	68.81 to 93.74

**K**	1.384 to 2.609
**Half Life**	0.2657 to 0.5010
**Tau**	0.3833 to 0.7228
**Goodness of Fit**	
**Degrees of Freedom**	31
**R squared**	0.9089
**Sum of Squares**	10116
**Sy.x**	18.06
**Constraints**	
**K**	K > 0
**Number of points**	
**# of X values**	34
**# Y values analyzed**	34

HYB

**One phase decay**	
**Best-fit values**	
**Y0**	524.1
**Plateau**	89.34
**K**	1.485
**Half Life**	0.4666
**Tau**	0.6732
**Span**	434.7
**95% CI (profile likelihood)**	
**Y0**	386.4 to 775.8
**Plateau**	80.60 to 96.98
**K**	1.113 to 1.944
**Half Life**	0.3566 to 0.6227
**Tau**	0.5145 to 0.8983
**Goodness of Fit**	
**Degrees of Freedom**	33
**R squared**	0.9230
**Sum of Squares**	4000
**Sy.x**	11.01
**Constraints**	
**K**	K > 0
**Number of points**	
**# of X values**	36
**# Y values analyzed**	36

Normality test

Normality was assumed.

Spearman's test for heteroscedasticity

**Spearman's test for heteroscedasticity**	
**Rs of predicted Y vs. |residual|**	0.5871
**P value (one tailed)**	<0.0001
**Passed (P > 0.05)?**	No

Two-way ANOVA

Two-way ANOVA	Ordinary				
**Alpha**	0.05				
**Source of Variation**	% of total variation	P value	P value summary	Significant?	
**Interaction**	24.67	<0.0001	****	Yes	
**Plasmid dose**	28.88	<0.0001	****	Yes	
**Circuit type**	42.92	<0.0001	****	Yes	
**ANOVA table**	SS	DF	MS	F (DFn, DFd)	P value
**Interaction**	75262	15	5017	F (15, 120) = 55.80	P<0.0001
**Plasmid dose**	88111	5	17622	F (5, 120) = 196.0	P<0.0001
**Circuit type**	130946	3	43649	F (3, 120) = 485.4	P<0.0001
**Residual**	10791	120	89.92		
**Data summary**					
**Number of columns (Circuit type)**	4				
**Number of rows (Plasmid dose)**	6				
**Number of values**	144				

Post- hoc Tukey tests

Tukey's multiple comparisons test	Mean Diff.	95.00% CI of diff.	Below threshold?	Summary	Adjusted P Value
**Row 1**					
**TRE vs. HYB**	39.33	25.07 to 53.60	Yes	****	<0.0001
**TRE vs. CMV**	104.0	89.74 to 118.3	Yes	****	<0.0001
**TRE vs. Equalizer-L**	172.8	158.5 to 187.1	Yes	****	<0.0001
**HYB vs. CMV**	64.67	50.40 to 78.93	Yes	****	<0.0001
**HYB vs. Equalizer-L**	133.5	119.2 to 147.7	Yes	****	<0.0001
**CMV vs. Equalizer-L**	68.80	54.54 to 83.06	Yes	****	<0.0001
**Row 2**					
**TRE vs. HYB**	19.83	5.569 to 34.10	Yes	**	0.0024
**TRE vs. CMV**	57.17	42.90 to 71.43	Yes	****	<0.0001
**TRE vs. Equalizer-L**	122.8	108.6 to 137.1	Yes	****	<0.0001
**HYB vs. CMV**	37.33	23.07 to 51.60	Yes	****	<0.0001
**HYB vs. Equalizer-L**	103.0	88.74 to 117.3	Yes	****	<0.0001
**CMV vs. Equalizer-L**	65.67	51.40 to 79.93	Yes	****	<0.0001
**Row 3**					
**TRE vs. HYB**	15.17	0.9023 to 29.43	Yes	*	0.0325
**TRE vs. CMV**	45.50	31.24 to 59.76	Yes	****	<0.0001
**TRE vs. Equalizer-L**	101.6	87.30 to 115.8	Yes	****	<0.0001
**HYB vs. CMV**	30.33	16.07 to 44.60	Yes	****	<0.0001
**HYB vs. Equalizer-L**	86.40	72.14 to 100.7	Yes	****	<0.0001
**CMV vs. Equalizer-L**	56.07	41.80 to 70.33	Yes	****	<0.0001

**Row 4**					
**TRE vs. HYB**	-4.900	-19.16 to 9.364	No	ns	0.8075
**TRE vs. CMV**	-9.633	-23.90 to 4.631	No	ns	0.2980
**TRE vs. Equalizer-L**	31.92	17.65 to 46.18	Yes	****	<0.0001
**HYB vs. CMV**	-4.733	-19.00 to 9.531	No	ns	0.8231
**HYB vs. Equalizer-L**	36.82	22.55 to 51.08	Yes	****	<0.0001
**CMV vs. Equalizer-L**	41.55	27.29 to 55.81	Yes	****	<0.0001
**Row 5**					
**TRE vs. HYB**	-7.533	-21.80 to 6.731	No	ns	0.5168
**TRE vs. CMV**	-12.53	-26.80 to 1.731	No	ns	0.1063
**TRE vs. Equalizer-L**	22.32	8.052 to 36.58	Yes	***	0.0005
**HYB vs. CMV**	-5.000	-19.26 to 9.264	No	ns	0.7978
**HYB vs. Equalizer-L**	29.85	15.59 to 44.11	Yes	****	<0.0001
**CMV vs. Equalizer-L**	34.85	20.59 to 49.11	Yes	****	<0.0001
**Row 6**					
**TRE vs. HYB**	-10.33	-24.60 to 3.931	No	ns	0.2389
**TRE vs. CMV**	-15.70	-29.96 to -1.436	Yes	*	0.0249
**TRE vs. Equalizer-L**	15.27	1.002 to 29.53	Yes	*	0.0309
**HYB vs. CMV**	-5.367	-19.63 to 8.898	No	ns	0.7610
**HYB vs. Equalizer-L**	25.60	11.34 to 39.86	Yes	****	<0.0001
**CMV vs. Equalizer-L**	30.97	16.70 to 45.23	Yes	****	<0.0001

Supp. Fig. 14g

Sample size (independent transfections)

	**OLP (original)**	**HYB (original)**
**HEK293T**	7	7
**CHO-K1**	3	3

Normality test

Normality was assumed.

Spearman's test for heteroscedasticity

**Spearman's test for heteroscedasticity**	
**Rs of predicted Y vs. |residual|**	-0.7447
**P value (one tailed)**	<0.0001
**Passed (P > 0.05)?**	No

Two-way ANOVA

Two-way ANOVA	Ordinary				
**Alpha**	0.05				
**Source of Variation**	% of total variation	P value	P value summary	Significant?	
**Interaction**	8.103	0.0828	ns	No	
**Cell line**	50.25	0.0003	***	Yes	
**Circuit type**	8.528	0.0758	ns	No	
**ANOVA table**	SS (Type III)	DF	MS	F (DFn, DFd)	P value
**Interaction**	510.4	1	510.4	F (1, 16) = 3.424	P=0.0828
**Cell line**	3165	1	3165	F (1, 16) = 21.23	P=0.0003
**Circuit type**	537.2	1	537.2	F (1, 16) = 3.604	P=0.0758
**Residual**	2385	16	149.1		
**Data summary**					
**Number of columns (Circuit type)**	2				
**Number of rows (Cell line)**	2				
**Number of values**	20				

Post-hoc Sidak tests

Šídák's multiple comparisons test	Predicted (LS) mean diff.	95.00% CI of diff.	Below threshold?	Summary	Adjusted P Value
**OLP (original) - HYB (original)**					
**HEK239T**	-0.2857	-16.38 to 15.81	No	ns	0.9988
**CHO-K1**	-22.33	-46.92 to 2.256	No	ns	0.0777

Supp. Fig. 14h

Sample size (independent transfections)

	OLP	HYB	CMV	Equalizer-L
**n**	6	6	6	6

Normality test

Normality was assumed.

Brown-Forsythe test

Brown-Forsythe test	
**F (DFn, DFd)**	6.447 (3, 20)
**P value**	0.0031
**P value summary**	**

**Are SDs significantly different (P < 0.05)?**	Yes

Welch's ANOVA test

**Welch's ANOVA test**	
**W (DFn, DFd)**	411.9 (3.000, 8.335)
**P value**	<0.0001
**P value summary**	****
**Significant diff. among means (P < 0.05)?**	Yes
**Data summary**	
**Number of treatments (columns)**	4
**Number of values (total)**	24

Post- hoc Dunnett's tests

**Dunnett's T3 multiple comparisons test**	**Mean Diff.**	**95.00% CI of diff.**	**Below threshold?**	**Summary**	**Adjusted P Value**
**OLP vs. HYB**	105548	83634 to 127461	Yes	****	<0.0001
**OLP vs. CMV**	-17852	-47200 to 11496	No	ns	0.2278
**OLP vs. Equalizer-L**	127509	104780 to 150238	Yes	****	<0.0001
**HYB vs. CMV**	-123400	-150848 to -95951	Yes	****	<0.0001
**HYB vs. Equalizer-L**	21961	14564 to 29359	Yes	***	0.0002
**CMV vs. Equalizer-L**	145361	116349 to 174373	Yes	****	<0.0001

Supp. Fig. 14i

Sample size (independent transfections)

	**CMV**	**PGK**	**Equalizer-L**
**n**	6	6	6

Normality test

Normality was assumed.

Brown-Forsythe test

**Brown-Forsythe test**	
**F (DFn, DFd)**	193.3 (2, 15)
**P value**	<0.0001
**P value summary**	****
**Are SDs significantly different (P < 0.05)?**	Yes

Welch's ANOVA test

Welch's ANOVA test	
W (DFn, DFd)	23.16 (2.000, 6.727)
P value	0.0010
P value summary	***
Significant diff. among means (P < 0.05)?	Yes
Data summary	
Number of treatments (columns)	3
Number of values (total)	18

Post- hoc Dunnett's tests

Dunnett's T3 multiple comparisons test	Mean Diff.	95.00% CI of diff.	Below threshold?	Summary	Adjusted P Value
CMV vs. PGK	84780	13228 to 156332	Yes	*	0.0125
CMV vs. Equalizer-L	87162	15635 to 158689	Yes	*	0.0111
PGK vs. Equalizer-L	2382	479.4 to 4284	Yes	**	0.0099

Supp. Fig. 14j

Sample size (independent transfections)

	OLP	HYB
1 ng	6	6
5 ng	6	6
10 ng	6	6
50 ng	6	6
100 ng	6	6
200 ng	6	6

Normality test

Normality was assumed.

Spearman's test for heteroscedasticity

Spearman's test for heteroscedasticity	
Rs of predicted Y vs. |residual|	0.6201
P value (one tailed)	<0.0001
Passed (P > 0.05)?	No

Two-way ANOVA

Two-way ANOVA	Ordinary				
Alpha	0.05				
Source of Variation	% of total variation	P value	P value summary	Significant?	

**Interaction**	22.07	<0.0001	****	Yes	
**Plasmid dose**	36.47	<0.0001	****	Yes	
**Circuit type**	39.41	<0.0001	****	Yes	
**ANOVA table**	SS	DF	MS	F (DFn, DFd)	P value
**Interaction**	45124211398	5	9024842280	F (5, 60) = 129.1	P<0.0001
**Plasmid dose**	74540547069	5	14908109414	F (5, 60) = 213.2	P<0.0001
**Circuit type**	80554357036	1	80554357036	F (1, 60) = 1152	P<0.0001
**Residual**	4195631057	60	69927184		
**Data summary**					
**Number of columns (Circuit type)**	2				
**Number of rows (Plasmid dose)**	6				
**Number of values**	72				

Post- hoc Sidak tests

**Šídák's multiple comparisons test**	**Mean Diff.**	**95.00% CI of diff.**	**Below threshold?**	**Summary**	**Adjusted P Value**
**OLP - HYB**					
**Row 1 (1 ng)**	9039	-4096 to 22174	No	ns	0.3364
**Row 2 (5 ng)**	18982	5847 to 32117	Yes	**	0.0013
**Row 3 (10 ng)**	28297	15162 to 41432	Yes	****	<0.0001
**Row 4 (50 ng)**	94959	81824 to 108094	Yes	****	<0.0001
**Row 5 (100 ng)**	111707	98572 to 124842	Yes	****	<0.0001
**Row 6 (200 ng)**	138400	125265 to 151535	Yes	****	<0.0001

Supp. Fig. 14j - continued

Sample size (independent transfections)

	**CMV**	**PGK**	**Equalizer-L**
**1 ng**	6	6	6
**5 ng**	6	6	6
**10 ng**	6	6	6
**50 ng**	6	6	6
**100 ng**	6	6	6
**200 ng**	6	6	6

Normality test

*N*ormality was assumed.

Spearman's test for heteroscedasticity

**Spearman's test for heteroscedasticity**	
**Rs of predicted Y vs. |residual|**	0.9633
**P value (one tailed)**	<0.0001
**Passed (P > 0.05)?**	No

Two-way ANOVA

**Two-way ANOVA**	**Ordinary**				
**Alpha**	0.05				
**Source of Variation**	% of total variation	P value	P value summary	Significant?	
**Interaction**	19.79	<0.0001	****	Yes	
**Plasmid dose**	11.48	<0.0001	****	Yes	
**Circuit type**	57.98	<0.0001	****	Yes	
**ANOVA table**	SS	DF	MS	F (DFn, DFd)	P value
**Interaction**	6240406186	10	624040619	F (10, 90) = 16.59	P<0.0001
**Plasmid dose**	3620390594	5	724078119	F (5, 90) = 19.24	P<0.0001
**Circuit type**	18280470365	2	9140235183	F (2, 90) = 242.9	P<0.0001
**Residual**	3386189801	90	37624331		
**Data summary**					
**Number of columns (Circuit type)**	3				
**Number of rows (Plasmid dose)**	6				
**Number of values**	108				

Post- hoc Sidak tests

**Tukey's multiple comparisons test**	**Mean Diff.**	**95.00% CI of diff.**	**Below threshold?**	**Summary**	**Adjusted P Value**
**Row 1 (1 ng)**					
**CMV vs. PGK**	4770	-3669 to 13210	No	ns	0.3732
**CMV vs. Equalizer-L**	4202	-4238 to 12641	No	ns	0.4643
**PGK vs. Equalizer-L**	-568.7	-9008 to 7871	No	ns	0.9859
**Row 2 (5 ng)**					
**CMV vs. PGK**	14392	5953 to 22831	Yes	***	0.0003
**CMV vs. Equalizer-L**	13730	5291 to 22170	Yes	***	0.0006
**PGK vs. Equalizer-L**	-661.7	-9101 to 7778	No	ns	0.9809
**Row 3 (10 ng)**					

**CMV vs. PGK**	19477	11038 to 27917	Yes	****	<0.0001
**CMV vs. Equalizer-L**	18819	10380 to 27259	Yes	****	<0.0001
**PGK vs. Equalizer-L**	-658.2	-9098 to 7781	No	ns	0.9811
**Row 4 (50 ng)**					
**CMV vs. PGK**	35840	27400 to 44279	Yes	****	<0.0001
**CMV vs. Equalizer-L**	35129	26689 to 43568	Yes	****	<0.0001
**PGK vs. Equalizer-L**	-711.2	-9151 to 7728	No	ns	0.9780
**Row 5 (100 ng)**					
**CMV vs. PGK**	43573	35134 to 52013	Yes	****	<0.0001
**CMV vs. Equalizer-L**	42921	34482 to 51360	Yes	****	<0.0001
**PGK vs. Equalizer-L**	-652.2	-9092 to 7787	No	ns	0.9815
**Row 6 (200 ng)**					
**CMV vs. PGK**	49204	40765 to 57644	Yes	****	<0.0001
**CMV vs. Equalizer-L**	49074	40635 to 57514	Yes	****	<0.0001
**PGK vs. Equalizer-L**	-130.0	-8569 to 8309	No	ns	0.9993

Supp. Fig. 15b

Sample size (independent transfections)

	**Equalizer-L**	**Multi-promoter Equalizer-L**
**n**	6	6

Normality test

Normality was assumed.

Spearman's test for heteroscedasticity

**Spearman's test for heteroscedasticity**	
**Rs of predicted Y vs. |residual|**	0.6115
**P value (one tailed)**	<0.0001
**Passed (P > 0.05)?**	No

Two-way ANOVA

**Two-way ANOVA**	**Ordinary**				
**Alpha**	0.05				
**Source of Variation**	% of total variation	P value	P value summary	Significant?	
**Interaction**	7.289	0.0033	**	Yes	

**Doxycycline concentration**	39.20	<0.0001	****	Yes	
**Circuit type**	30.42	<0.0001	****	Yes	
**ANOVA table**	SS (Type III)	DF	MS	F (DFn, DFd)	P value
**Interaction**	3786	6	631.0	F (6, 69) = 3.649	P=0.0033
**Doxycycline concentration**	20363	6	3394	F (6, 69) = 19.63	P<0.0001
**Circuit type**	15802	1	15802	F (1, 69) = 91.38	P<0.0001
**Residual**	11932	69	172.9		
**Data summary**					
**Number of columns (Circuit type)**	2				
**Number of rows (Doxycycline concentration)**	7				
**Number of values**	83				

Post- hoc Sidak tests

**Šídák's multiple comparisons test**	**Predicted (LS) mean diff.**	**95.00% CI of diff.**	**Below threshold?**	**Summary**	**Adjusted P Value**
**Equalizer-L - Multi-promoter Equalizer-L**					
**Row 1 (0 ng/mL)**	-1.383	-22.37 to 19.61	No	ns	>0.9999
**Row 2 (1 ng/mL)**	-20.85	-41.84 to 0.1408	No	ns	0.0526
**Row 3 (3 ng/mL)**	-22.97	-43.96 to -1.976	Yes	*	0.0242
**Row 4 (5 ng/mL)**	-30.37	-51.36 to -9.376	Yes	**	0.0011
**Row 5 (7 ng/mL)**	-32.52	-54.53 to -10.50	Yes	***	0.0008
**Row 6 (10 ng/mL)**	-39.12	-60.11 to -18.13	Yes	****	<0.0001
**Row 7 (30 ng/mL)**	-46.18	-67.17 to -25.19	Yes	****	<0.0001

Supp. Fig. 20a

Sample size (independent transfections or culture)

	**CMV episome**	**PGK episome**	**Equalizer-L episome**	**CMV cell line**
**n**	4	4	4	4

Normality test

Normality was assumed.

Spearman's test for heteroscedasticity

Spearman's test for heteroscedasticity	
Rs of predicted Y vs. |residual|	0.7617
P value (one tailed)	<0.0001
Passed (P > 0.05)?	No

Two-way ANOVA

Two-way ANOVA	Ordinary				
Alpha	0.05				
Source of Variation	% of total variation	P value	P value summary	Significant?	
Interaction	14.90	<0.0001	****	Yes	
Days after transfection	7.850	<0.0001	****	Yes	
Circuit type	74.53	<0.0001	****	Yes	
ANOVA table	SS	DF	MS	F (DFn, DFd)	P value
Interaction	6564892483	12	547074374	F (12, 60) = 27.33	P<0.0001
Days after transfection	3459826087	4	864956522	F (4, 60) = 43.22	P<0.0001
Circuit type	32847417131	3	10949139044	F (3, 60) = 547.1	P<0.0001
Residual	1200855069	60	20014251		
Data summary					
Number of columns (Circuit type)	4				
Number of rows (Days after transfection)	5				
Number of values	80				

Post- hoc Tukey tests

Tukey's multiple comparisons test	Mean Diff.	95.00% CI of diff.	Below threshold?	Summary	Adjusted P Value
Row 1					
CMV episome vs. PGK episome	58915	50556 to 67274	Yes	****	<0.0001
CMV episome vs. Equalizer-L episome	67961	59601 to 76320	Yes	****	<0.0001
CMV episome vs. CMV cell line	13467	5108 to 21826	Yes	***	0.0004
PGK episome vs. Equalizer-L episome	9046	686.5 to 17405	Yes	*	0.0290
PGK episome vs. CMV cell line	-45448	-53807 to -37089	Yes	****	<0.0001
Equalizer-L episome vs. CMV cell line	-54494	-62853 to -46134	Yes	****	<0.0001
Row 2					
CMV episome vs. PGK episome	10909	2549 to 19268	Yes	**	0.0056

**CMV episome vs. Equalizer-L episome**	14989	6629 to 23348	Yes	****	<0.0001
**CMV episome vs. CMV cell line**	-39498	-47857 to -31139	Yes	****	<0.0001
**PGK episome vs. Equalizer-L episome**	4080	-4279 to 12439	No	ns	0.5730
**PGK episome vs. CMV cell line**	-50407	-58766 to -42047	Yes	****	<0.0001
**Equalizer-L episome vs. CMV cell line**	-54487	-62846 to -46127	Yes	****	<0.0001
**Row 3**					
**CMV episome vs. PGK episome**	10608	2248 to 18967	Yes	**	0.0074
**CMV episome vs. Equalizer-L episome**	13696	5337 to 22056	Yes	***	0.0003
**CMV episome vs. CMV cell line**	-34603	-42962 to -26243	Yes	****	<0.0001
**PGK episome vs. Equalizer-L episome**	3089	-5271 to 11448	No	ns	0.7634
**PGK episome vs. CMV cell line**	-45210	-53570 to -36851	Yes	****	<0.0001
**Equalizer-L episome vs. CMV cell line**	-48299	-56658 to -39939	Yes	****	<0.0001
**Row 4**					
**CMV episome vs. PGK episome**	10344	1985 to 18703	Yes	**	0.0094
**CMV episome vs. Equalizer-L episome**	12783	4423 to 21142	Yes	***	0.0009
**CMV episome vs. CMV cell line**	-32677	-41036 to -24318	Yes	****	<0.0001
**PGK episome vs. Equalizer-L episome**	2439	-5920 to 10798	No	ns	0.8672
**PGK episome vs. CMV cell line**	-43021	-51380 to -34661	Yes	****	<0.0001
**Equalizer-L episome vs. CMV cell line**	-45460	-53819 to -37100	Yes	****	<0.0001
**Row 5**					
**CMV episome vs. PGK episome**	8920	560.3 to 17279	Yes	*	0.0321
**CMV episome vs. Equalizer-L episome**	11404	3045 to 19763	Yes	**	0.0035
**CMV episome vs. CMV cell line**	-42084	-50443 to -33725	Yes	****	<0.0001
**PGK episome vs. Equalizer-L episome**	2485	-5875 to 10844	No	ns	0.8608
**PGK episome vs. CMV cell line**	-51004	-59363 to -42644	Yes	****	<0.0001
**Equalizer-L episome vs. CMV cell line**	-53488	-61848 to -45129	Yes	****	<0.0001

Supp. Fig. 20b

Sample size (independent transfections or culture)

	CMV episome	PGK episome	Equalizer-L episome	CMV cell line
**n**	4	4	4	4

Normality test

Normality was assumed.

Spearman's test for heteroscedasticity

Spearman's test for heteroscedasticity	
**Rs of predicted Y vs. |residual|**	0.3811
**P value (one tailed)**	0.0013
**Passed (P > 0.05)?**	No

Two-way ANOVA

Two-way ANOVA	Ordinary				
**Alpha**	0.05				
**Source of Variation**	% of total variation	P value	P value summary	Significant?	
**Interaction**	16.41	<0.0001	****	Yes	
**Days after transfection**	54.06	<0.0001	****	Yes	
**Circuit type**	26.65	<0.0001	****	Yes	
**ANOVA table**	SS	DF	MS	F (DFn, DFd)	P value
**Interaction**	16126727	8	2015841	F (8, 45) = 32.09	P<0.0001
**Days after transfection**	53117086	4	13279271	F (4, 45) = 211.4	P<0.0001
**Circuit type**	26186452	2	13093226	F (2, 45) = 208.4	P<0.0001
**Residual**	2827000	45	62822		
**Data summary**					
**Number of columns (Circuit type)**	3				
**Number of rows (Days after transfection)**	5				
**Number of values**	60				

Post- hoc Tukey tests

Tukey's multiple comparisons test	Mean Diff.	95.00% CI of diff.	Below threshold?	Summary	Adjusted P Value
**Row 1 (Day 9)**					
**CMV episome vs. PGK episome**	-3582	-4011 to -3152	Yes	****	<0.0001
**CMV episome vs. Equalizer-L episome**	-3274	-3703 to -2844	Yes	****	<0.0001
**PGK episome vs. Equalizer-L episome**	307.9	-121.7 to 737.4	No	ns	0.2027

**Row 2 (Day 16)**					
**CMV episome vs. PGK episome**	-1294	-1723 to -864.0	Yes	****	<0.0001
**CMV episome vs. Equalizer-L episome**	-1679	-2109 to -1250	Yes	****	<0.0001
**PGK episome vs. Equalizer-L episome**	-385.8	-815.3 to 43.79	No	ns	0.0863
**Row 3 (Day 23)**					
**CMV episome vs. PGK episome**	-721.0	-1151 to -291.5	Yes	***	0.0005
**CMV episome vs. Equalizer-L episome**	-993.9	-1423 to -564.3	Yes	****	<0.0001
**PGK episome vs. Equalizer-L episome**	-272.9	-702.4 to 156.7	No	ns	0.2824
**Row 4 (Day 40)**					
**CMV episome vs. PGK episome**	-641.1	-1071 to -211.6	Yes	**	0.0021
**CMV episome vs. Equalizer-L episome**	-1017	-1447 to -587.8	Yes	****	<0.0001
**PGK episome vs. Equalizer-L episome**	-376.3	-805.8 to 53.29	No	ns	0.0965
**Row 5 (Day 60)**					
**CMV episome vs. PGK episome**	-371.1	-800.7 to 58.42	No	ns	0.1025
**CMV episome vs. Equalizer-L episome**	-383.3	-812.8 to 46.29	No	ns	0.0889
**PGK episome vs. Equalizer-L episome**	-12.13	-441.7 to 417.4	No	ns	0.9974

Supp. Fig. 20c

Sample size (independent transfections or culture)

	**CMV episome**	**PGK episome**	**Equalizer-L episome**
**n**	4	4	4

Normality test

Normality was assumed.

Spearman's test for heteroscedasticity

**Spearman's test for heteroscedasticity**	
**Rs of predicted Y vs. |residual|**	-0.3856
**P value (one tailed)**	0.0012
**Passed (P > 0.05)?**	No

Two-way ANOVA

**Two-way ANOVA**	**Ordinary**				
**Alpha**	0.05				

Source of Variation	% of total variation	P value	P value summary	Significant?	
**Interaction**	2.982	0.0017	**	Yes	
**Days after transfection**	87.28	<0.0001	****	Yes	
**Circuit type**	5.351	<0.0001	****	Yes	
**ANOVA table**	SS	DF	MS	F (DFn, DFd)	P value
**Interaction**	0.1413	8	0.01767	F (8, 45) = 3.822	P=0.0017
**Days after transfection**	4.136	4	1.034	F (4, 45) = 223.7	P<0.0001
**Circuit type**	0.2536	2	0.1268	F (2, 45) = 27.43	P<0.0001
**Residual**	0.2080	45	0.004623		
**Data summary**					
**Number of columns (Circuit type)**	3				
**Number of rows (Days after transfection)**	5				
**Number of values**	60				

<u>Post- hoc Tukey tests</u>

Tukey's multiple comparisons test	Mean Diff.	95.00% CI of diff.	Below threshold?	Summary	Adjusted P Value
**Row 1 (Day 9)**					
**CMV episome vs. PGK episome**	0.000	-0.1165 to 0.1165	No	ns	>0.9999
**CMV episome vs. Equalizer-L episome**	0.000	-0.1165 to 0.1165	No	ns	>0.9999
**PGK episome vs. Equalizer-L episome**	0.000	-0.1165 to 0.1165	No	ns	>0.9999
**Row 2 (Day 16)**					
**CMV episome vs. PGK episome**	0.1425	0.02599 to 0.2590	Yes	*	0.0132
**CMV episome vs. Equalizer-L episome**	0.02172	-0.09480 to 0.1382	No	ns	0.8939
**PGK episome vs. Equalizer-L episome**	-0.1208	-0.2373 to -0.004271	Yes	*	0.0407
**Row 3 (Day 23)**					
**CMV episome vs. PGK episome**	0.2080	0.09146 to 0.3245	Yes	***	0.0002
**CMV episome vs. Equalizer-L episome**	0.1222	0.005684 to 0.2387	Yes	*	0.0379
**PGK episome vs. Equalizer-L episome**	-0.08577	-0.2023 to 0.03074	No	ns	0.1864
**Row 4 (Day 40)**					
**CMV episome vs. PGK episome**	0.1538	0.03731 to 0.2703	Yes	**	0.0070

**CMV episome vs. Equalizer-L episome**	0.04023	-0.07629 to 0.1567	No	ns	0.6824
**PGK episome vs. Equalizer-L episome**	-0.1136	-0.2301 to 0.002924	No	ns	0.0574
**Row 5 (Day 60)**					
**CMV episome vs. PGK episome**	0.2891	0.1726 to 0.4056	Yes	****	<0.0001
**CMV episome vs. Equalizer-L episome**	0.2705	0.1540 to 0.3870	Yes	****	<0.0001
**PGK episome vs. Equalizer-L episome**	-0.01861	-0.1351 to 0.09791	No	ns	0.9209

Supp. Fig. 20d

Sample size (independent transfections or culture)

	CMV episome	PGK episome	Equalizer-L episome	CMV cell line
**n**	4	4	4	4

Normality test

Normality was assumed.

Spearman's test for heteroscedasticity

Spearman's test for heteroscedasticity	
**Rs of predicted Y vs. |residual|**	0.5514
**P value (one tailed)**	<0.0001
**Passed (P > 0.05)?**	No

Two-way ANOVA

Two-way ANOVA	Ordinary				
**Alpha**	0.05				
**Source of Variation**	% of total variation	P value	P value summary	Significant?	
**Interaction**	24.97	<0.0001	****	Yes	
**Days after transfection**	14.67	<0.0001	****	Yes	
**Circuit type**	55.18	<0.0001	****	Yes	
**ANOVA table**	SS	DF	MS	F (DFn, DFd)	P value
**Interaction**	96926	12	8077	F (12, 60) = 24.10	P<0.0001
**Days after transfection**	56943	4	14236	F (4, 60) = 42.48	P<0.0001
**Circuit type**	214192	3	71397	F (3, 60) = 213.1	P<0.0001
**Residual**	20106	60	335.1		
**Data summary**					

**Number of columns (Circuit type)**	4				
**Number of rows (Days after transfection)**	5				
**Number of values**	80				

Post- hoc Tukey tests

**Tukey's multiple comparisons test**	**Mean Diff.**	**95.00% CI of diff.**	**Below threshold?**	**Summary**	**Adjusted P Value**
**Row 1 (Day 9)**					
**CMV episome vs. PGK episome**	70.50	36.30 to 104.7	Yes	****	<0.0001
**CMV episome vs. Equalizer-L episome**	242.2	208.0 to 276.4	Yes	****	<0.0001
**CMV episome vs. CMV cell line**	239.3	205.1 to 273.5	Yes	****	<0.0001
**PGK episome vs. Equalizer-L episome**	171.7	137.5 to 205.9	Yes	****	<0.0001
**PGK episome vs. CMV cell line**	168.8	134.6 to 203.0	Yes	****	<0.0001
**Equalizer-L episome vs. CMV cell line**	-2.838	-37.04 to 31.37	No	ns	0.9962
**Row 2 (Day 16)**					
**CMV episome vs. PGK episome**	-37.75	-71.95 to -3.545	Yes	*	0.0250
**CMV episome vs. Equalizer-L episome**	94.51	60.31 to 128.7	Yes	****	<0.0001
**CMV episome vs. CMV cell line**	101.4	67.20 to 135.6	Yes	****	<0.0001
**PGK episome vs. Equalizer-L episome**	132.3	98.06 to 166.5	Yes	****	<0.0001
**PGK episome vs. CMV cell line**	139.2	104.9 to 173.4	Yes	****	<0.0001
**Equalizer-L episome vs. CMV cell line**	6.888	-27.32 to 41.09	No	ns	0.9509
**Row 3 (Day 23)**					
**CMV episome vs. PGK episome**	-85.98	-120.2 to -51.77	Yes	****	<0.0001
**CMV episome vs. Equalizer-L episome**	42.44	8.233 to 76.64	Yes	**	0.0092
**CMV episome vs. CMV cell line**	49.84	15.63 to 84.04	Yes	**	0.0016
**PGK episome vs. Equalizer-L episome**	128.4	94.21 to 162.6	Yes	****	<0.0001
**PGK episome vs. CMV cell line**	135.8	101.6 to 170.0	Yes	****	<0.0001
**Equalizer-L episome vs. CMV cell line**	7.400	-26.80 to 41.60	No	ns	0.9401
**Row 4 (Day 40)**					
**CMV episome vs. PGK episome**	-49.85	-84.05 to -15.65	Yes	**	0.0016

**CMV episome vs. Equalizer-L episome**	39.34	5.133 to 73.54	Yes	*	0.0180
**CMV episome vs. CMV cell line**	39.01	4.808 to 73.22	Yes	*	0.0192
**PGK episome vs. Equalizer-L episome**	89.19	54.98 to 123.4	Yes	****	<0.0001
**PGK episome vs. CMV cell line**	88.86	54.66 to 123.1	Yes	****	<0.0001
**Equalizer-L episome vs. CMV cell line**	-0.3250	-34.53 to 33.88	No	ns	>0.9999
**Row 5 (Day 60)**					
**CMV episome vs. PGK episome**	-25.40	-59.60 to 8.805	No	ns	0.2138
**CMV episome vs. Equalizer-L episome**	30.94	-3.267 to 65.14	No	ns	0.0899
**CMV episome vs. CMV cell line**	11.29	-22.92 to 45.49	No	ns	0.8193
**PGK episome vs. Equalizer-L episome**	56.34	22.13 to 90.54	Yes	***	0.0003
**PGK episome vs. CMV cell line**	36.69	2.483 to 70.89	Yes	*	0.0309
**Equalizer-L episome vs. CMV cell line**	-19.65	-53.85 to 14.55	No	ns	0.4333

Supp. Fig. 20e

Sample size (independent transfections or culture)

	CMV episome	PGK episome	Equalizer-L episome	CMV cell line
**n**	4	4	4	4

Normality test

Normality was assumed.

Spearman's test for heteroscedasticity

**Spearman's test for heteroscedasticity**	
**Rs of predicted Y vs. |residual|**	0.02186
**P value (one tailed)**	0.4247
**Passed (P > 0.05)?**	Yes

Two-way ANOVA

**Two-way ANOVA**	**Ordinary**				
**Alpha**	0.05				
**Source of Variation**	% of total variation	P value	P value summary	Significant?	
**Interaction**	15.07	0.0010	***	Yes	
**Days after transfection**	13.37	<0.0001	****	Yes	

**Circuit type**	49.57	<0.0001	****	Yes	
**ANOVA table**	SS (Type III)	DF	MS	F (DFn, DFd)	P value
**Interaction**	1.335	12	0.1113	F (12, 58) = 3.346	P=0.0010
**Days after transfection**	1.184	4	0.2961	F (4, 58) = 8.905	P<0.0001
**Circuit type**	4.391	3	1.464	F (3, 58) = 44.01	P<0.0001
**Residual**	1.929	58	0.03325		
**Data summary**					
**Number of columns (Circuit type)**	4				
**Number of rows (Days after transfection)**	5				
**Number of values**	78				

<u>Post- hoc Tukey tests</u>

**Tukey's multiple comparisons test**	**Predicted (LS) mean diff.**	**95.00% CI of diff.**	**Below threshold?**	**Summary**	**Adjusted P Value**
**Row 1 (Day 9)**					
**CMV episome vs. PGK episome**	0.000	-0.3411 to 0.3411	No	ns	>0.9999
**CMV episome vs. Equalizer-L episome**	0.000	-0.3411 to 0.3411	No	ns	>0.9999
**CMV episome vs. CMV cell line**	0.000	-0.3411 to 0.3411	No	ns	>0.9999
**PGK episome vs. Equalizer-L episome**	0.000	-0.3411 to 0.3411	No	ns	>0.9999
**PGK episome vs. CMV cell line**	0.000	-0.3411 to 0.3411	No	ns	>0.9999
**Equalizer-L episome vs. CMV cell line**	0.000	-0.3411 to 0.3411	No	ns	>0.9999
**Row 2 (Day 16)**					
**CMV episome vs. PGK episome**	0.09440	-0.2467 to 0.4355	No	ns	0.8838
**CMV episome vs. Equalizer-L episome**	-0.6296	-0.9707 to -0.2885	Yes	****	<0.0001
**CMV episome vs. CMV cell line**	-0.4752	-0.8163 to -0.1341	Yes	**	0.0028
**PGK episome vs. Equalizer-L episome**	-0.7240	-1.065 to -0.3829	Yes	****	<0.0001
**PGK episome vs. CMV cell line**	-0.5696	-0.9107 to -0.2285	Yes	***	0.0003
**Equalizer-L episome vs. CMV cell line**	0.1544	-0.1867 to 0.4955	No	ns	0.6309
**Row 3 (Day 23)**					
**CMV episome vs. PGK episome**	0.2266	-0.1145 to 0.5677	No	ns	0.3042
**CMV episome vs. Equalizer-L episome**	-0.4896	-0.8306 to -0.1485	Yes	**	0.0020

**CMV episome vs. CMV cell line**	-0.3462	-0.6873 to -0.005129	Yes	*	0.0454
**PGK episome vs. Equalizer-L episome**	-0.7162	-1.057 to -0.3751	Yes	****	<0.0001
**PGK episome vs. CMV cell line**	-0.5728	-0.9139 to -0.2318	Yes	***	0.0002
**Equalizer-L episome vs. CMV cell line**	0.1434	-0.1977 to 0.4844	No	ns	0.6838
**Row 4 (Day 40)**					
**CMV episome vs. PGK episome**	0.2327	-0.1084 to 0.5738	No	ns	0.2815
**CMV episome vs. Equalizer-L episome**	-0.5196	-0.8607 to -0.1785	Yes	***	0.0009
**CMV episome vs. CMV cell line**	-0.3431	-0.6842 to -0.002059	Yes	*	0.0481
**PGK episome vs. Equalizer-L episome**	-0.7523	-1.093 to -0.4112	Yes	****	<0.0001
**PGK episome vs. CMV cell line**	-0.5759	-0.9169 to -0.2348	Yes	***	0.0002
**Equalizer-L episome vs. CMV cell line**	0.1764	-0.1646 to 0.5175	No	ns	0.5240
**Row 5 (Day 60)**					
**CMV episome vs. PGK episome**	0.1666	-0.1745 to 0.5077	No	ns	0.5717
**CMV episome vs. Equalizer-L episome**	-0.4199	-0.7609 to -0.07878	Yes	**	0.0099
**CMV episome vs. CMV cell line**	-0.7088	-1.127 to -0.2911	Yes	***	0.0002
**PGK episome vs. Equalizer-L episome**	-0.5865	-0.9275 to -0.2454	Yes	***	0.0002
**PGK episome vs. CMV cell line**	-0.8754	-1.293 to -0.4577	Yes	****	<0.0001
**Equalizer-L episome vs. CMV cell line**	-0.2889	-0.7067 to 0.1288	No	ns	0.2702

Supp. Fig. 22a

Sample size (independent transfections)

	PGK	PGK+
**n**	6	6

Normality test

Normality was assumed.

F-test

**F test to compare variances**	
**F, DFn, Dfd**	1.101, 5, 5
**P value**	0.9187
**P value summary**	ns
**Significantly different (P < 0.05)?**	No

Unpaired t-test

**Unpaired t test**	
**P value**	0.5384
**P value summary**	ns
**Significantly different (P < 0.05)?**	No
**One- or two-tailed P value?**	Two-tailed
**t, df**	t=0.6370, df=10

Supp. Fig. 22b

Sample size (independent transfections)

	**PGK**	**PGK+**
**n**	6	6

Normality test

Normality was assumed.

F-test

**F test to compare variances**	
**F, DFn, Dfd**	7.638, 5, 5
**P value**	0.0435
**P value summary**	*
**Significantly different (P < 0.05)?**	Yes

Unpaired t-test

**Unpaired t test with Welch's correction**	
**P value**	0.0427
**P value summary**	*
**Significantly different (P < 0.05)?**	Yes
**One- or two-tailed P value?**	Two-tailed
**Welch-corrected t, df**	t=2.534, df=6.287
