# Supplementary figures and images for: A synthetic circuit for buffering gene dosage variation between individual mammalian cells
Source: Nat Commun. 2021 Jul 5;12:4132. doi: 10.1038/s41467-021-23889-0 (PMC8257781; doi:10.1038/s41467-021-23889-0)

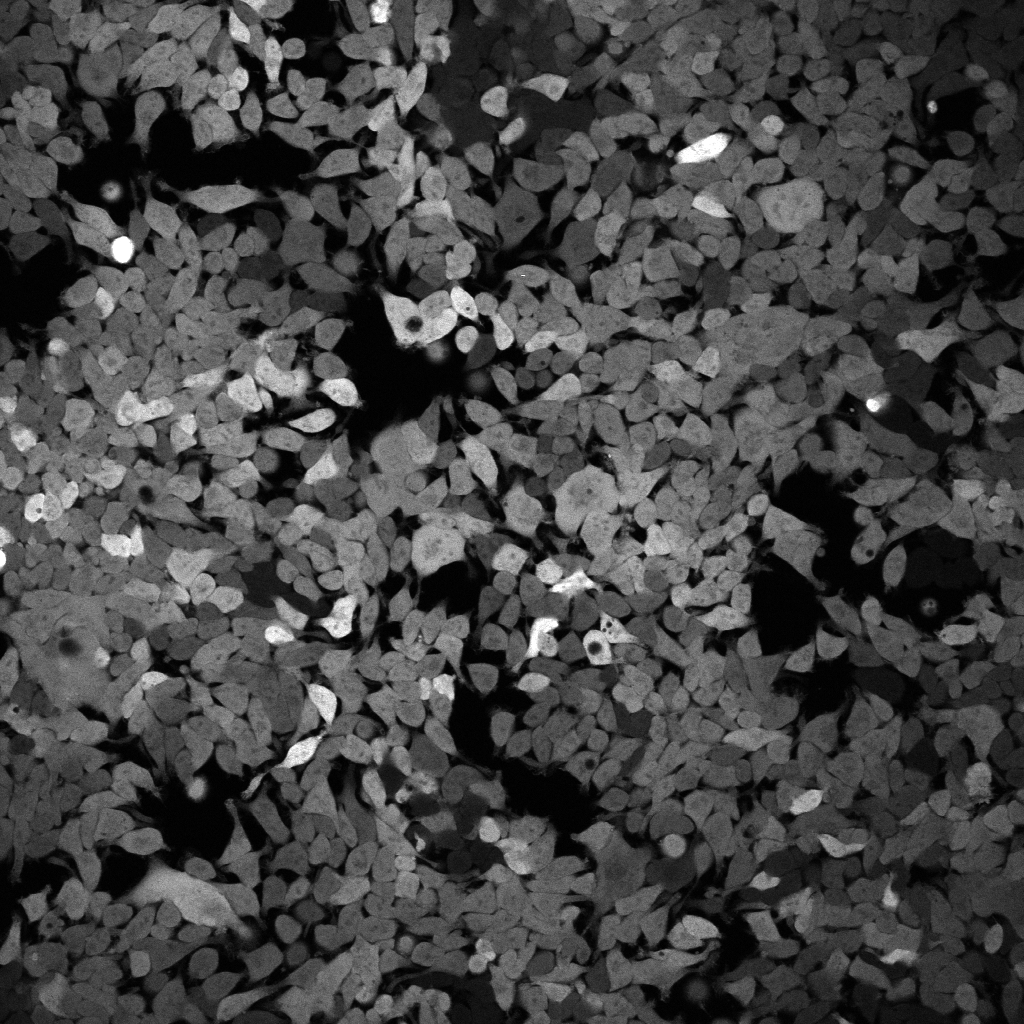

Supplement: Supplementary file 3 — Source Data [file 41467_2021_23889_MOESM3_ESM.zip › Raw microscopy images/Figure 6 and Supp Fig 18/Day 16_CMV cell line.tif]

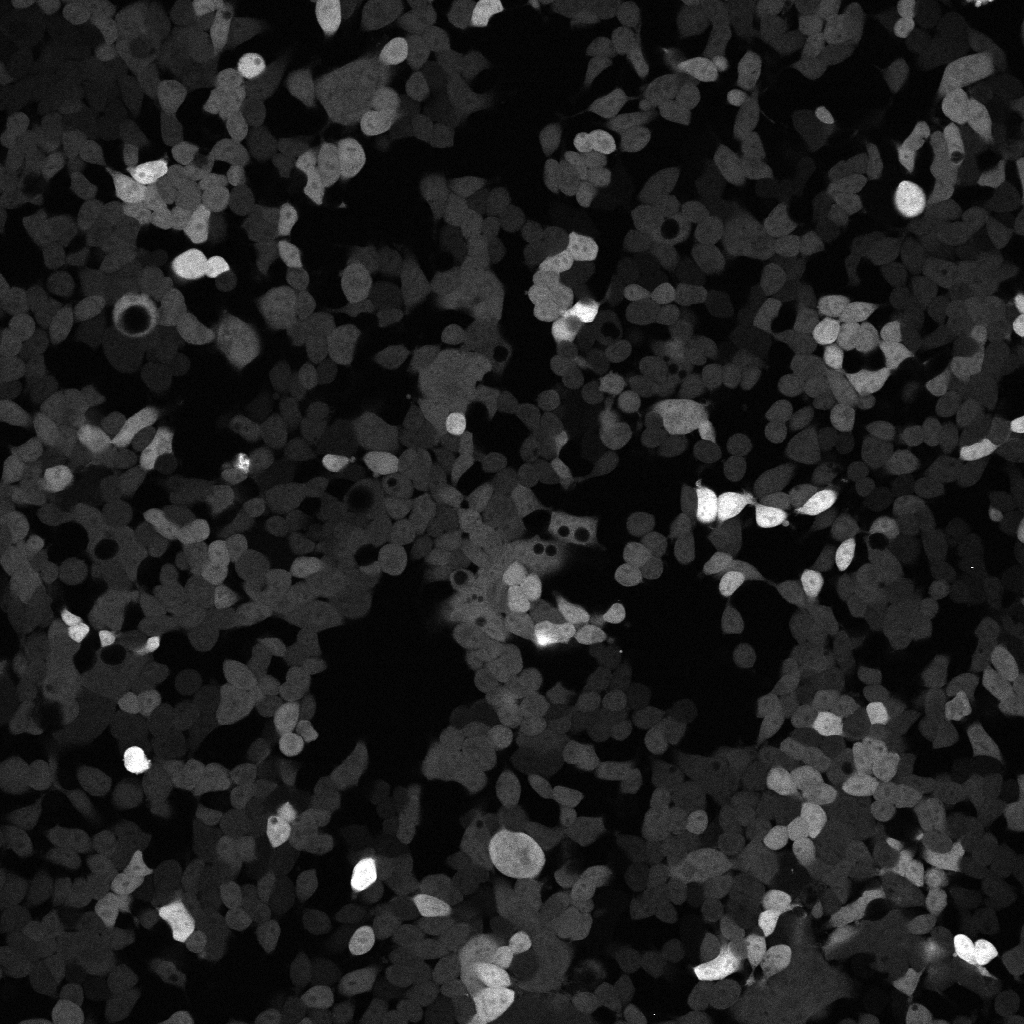

Supplement: Supplementary file 3 — Source Data [file 41467_2021_23889_MOESM3_ESM.zip › Raw microscopy images/Figure 6 and Supp Fig 18/Day 16_CMV episome.tif]

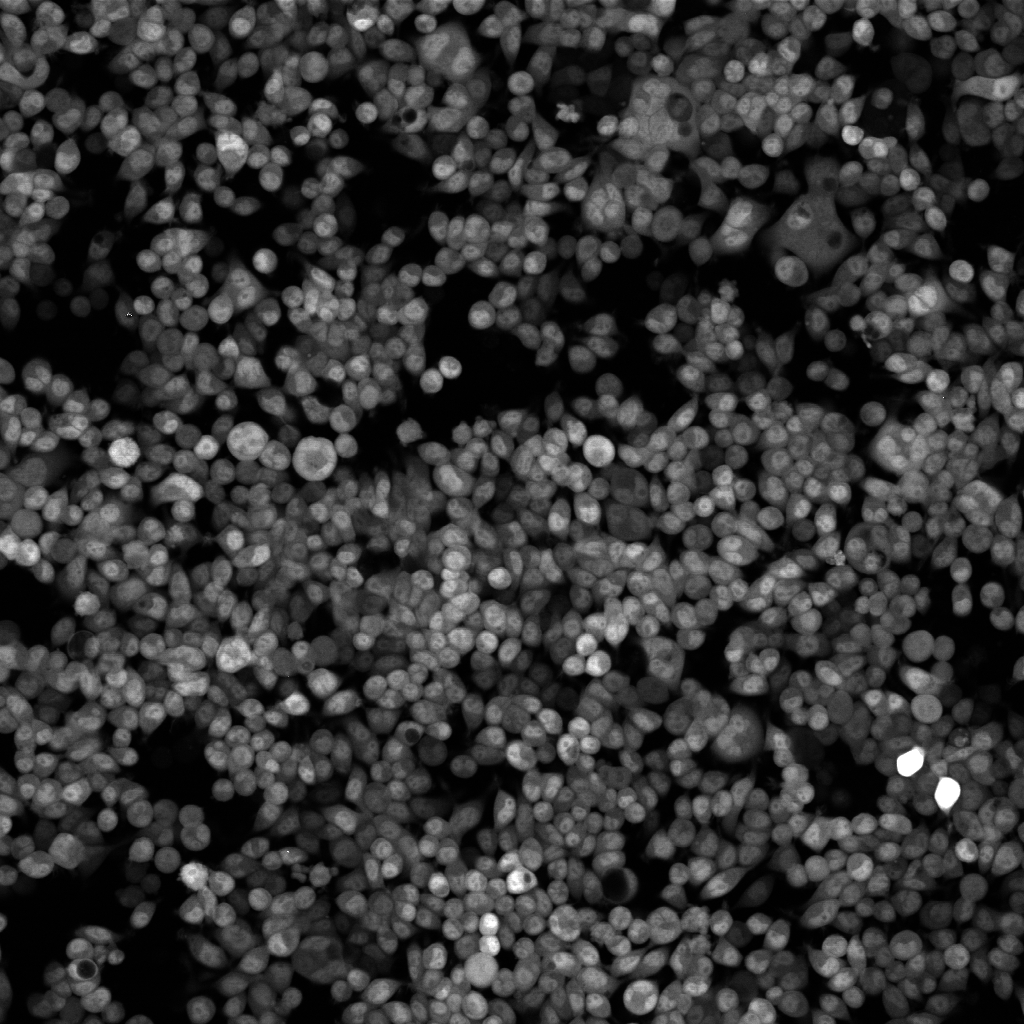

Supplement: Supplementary file 3 — Source Data [file 41467_2021_23889_MOESM3_ESM.zip › Raw microscopy images/Figure 6 and Supp Fig 18/Day 16_Equalizer-L episome.tif]

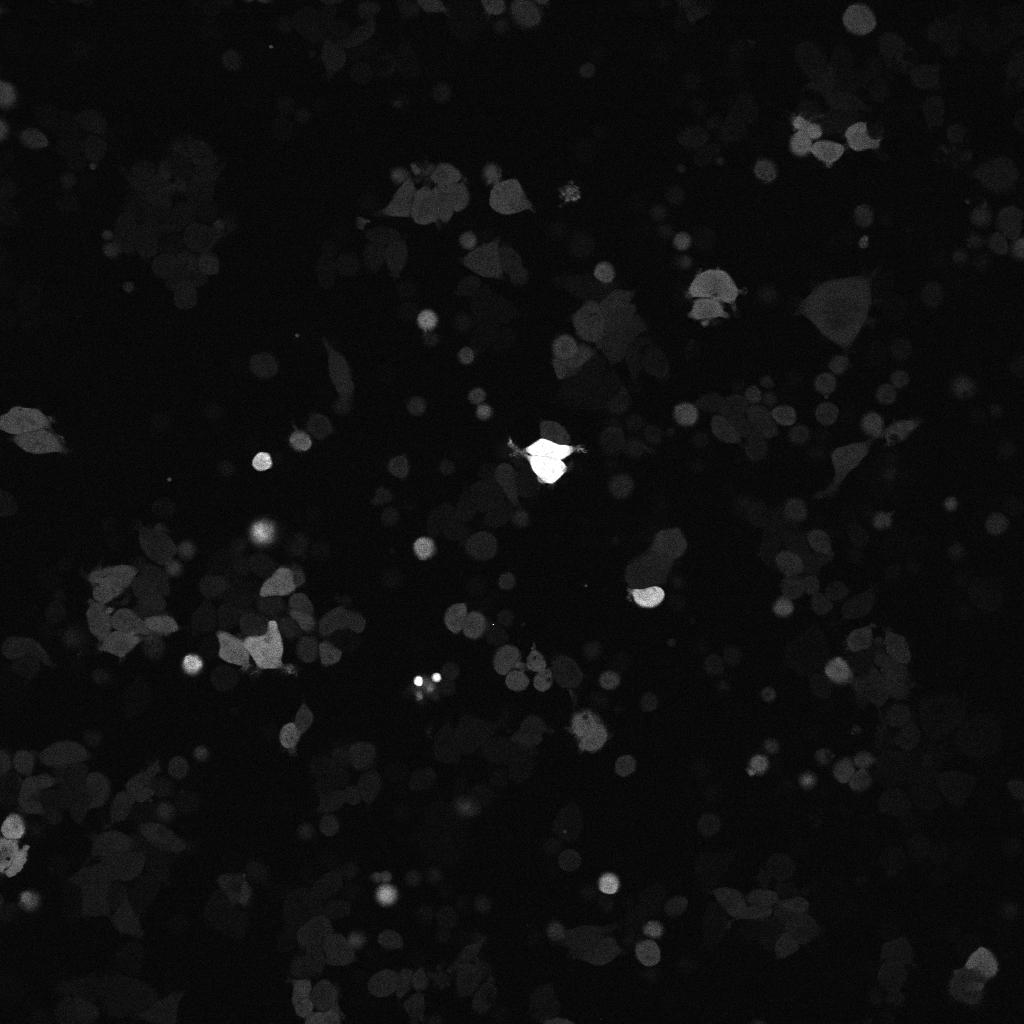

Supplement: Supplementary file 3 — Source Data [file 41467_2021_23889_MOESM3_ESM.zip › Raw microscopy images/Figure 6 and Supp Fig 18/Day 16_PGK episome.tif]

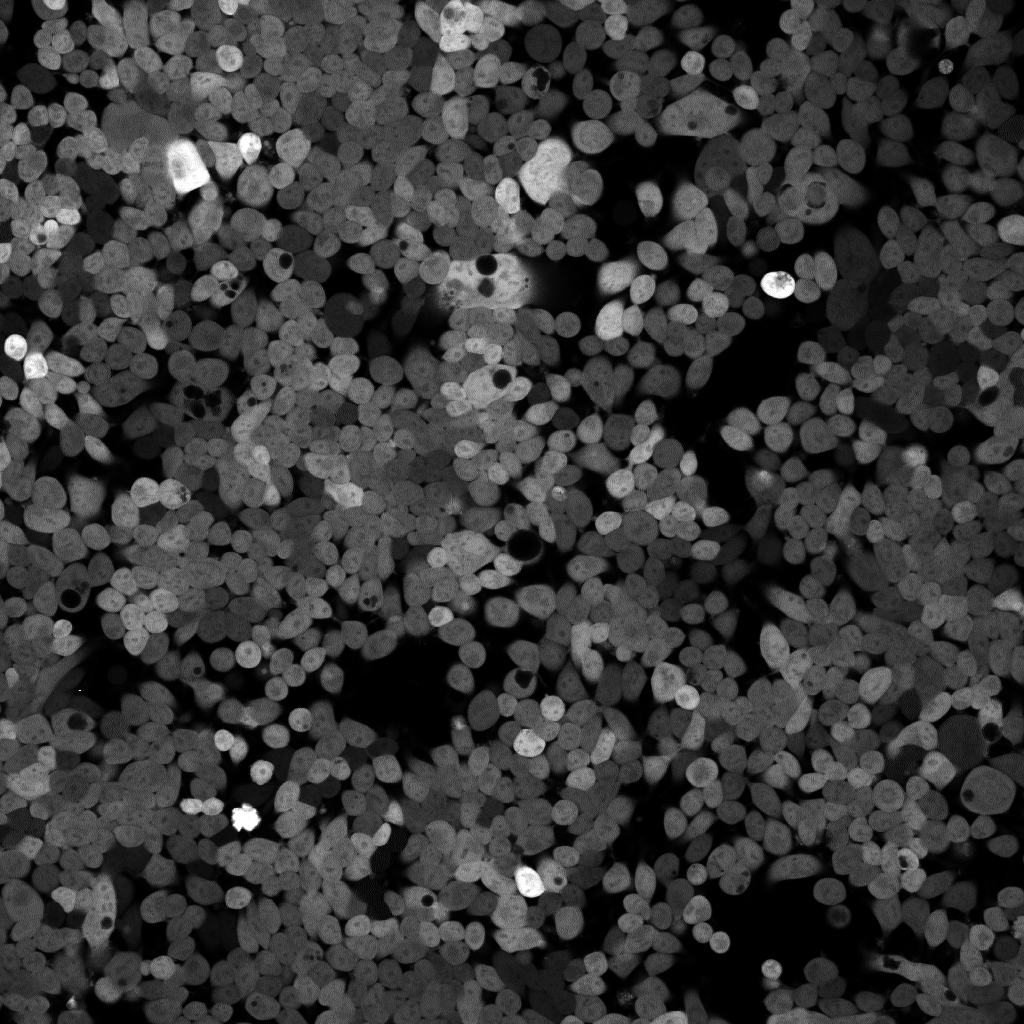

Supplement: Supplementary file 3 — Source Data [file 41467_2021_23889_MOESM3_ESM.zip › Raw microscopy images/Figure 6 and Supp Fig 18/Day 2_CMV cell line.tif]

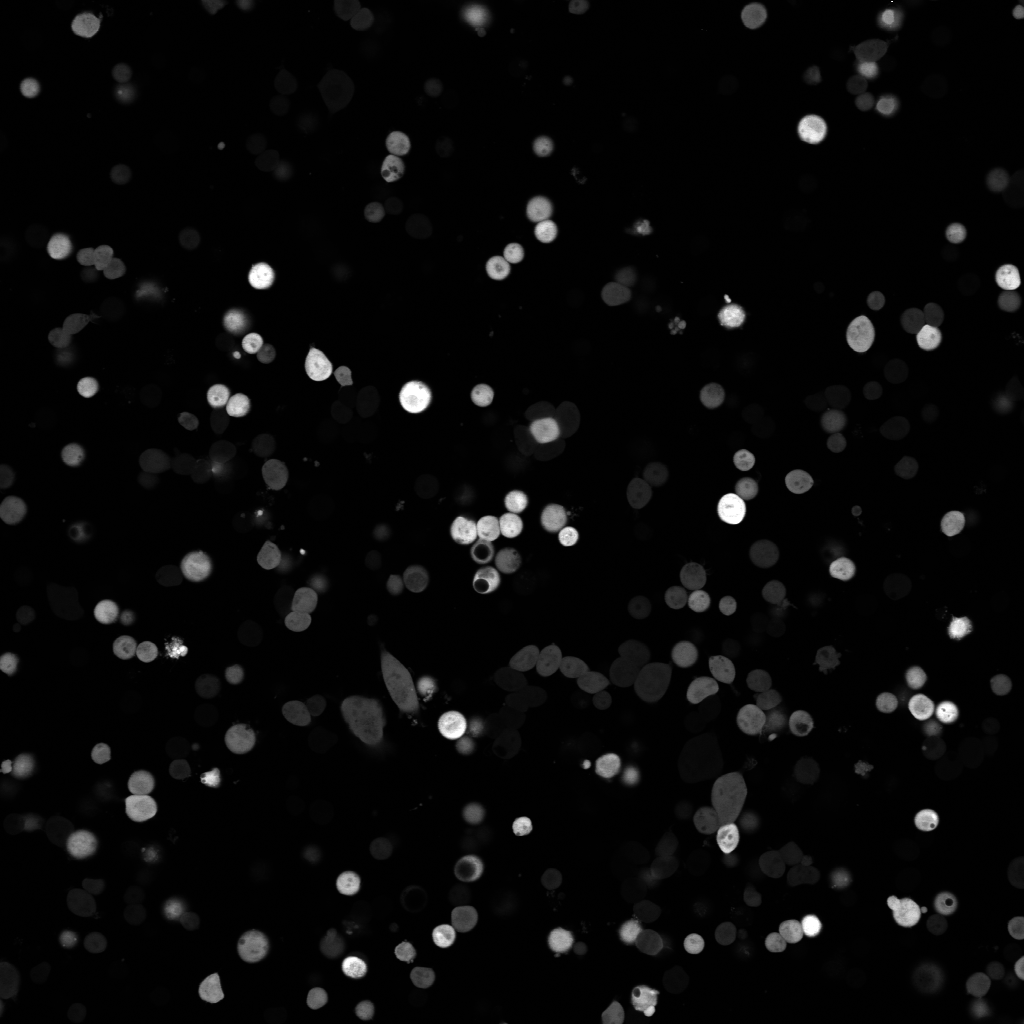

Supplement: Supplementary file 3 — Source Data [file 41467_2021_23889_MOESM3_ESM.zip › Raw microscopy images/Figure 6 and Supp Fig 18/Day 2_CMV episome.tif]

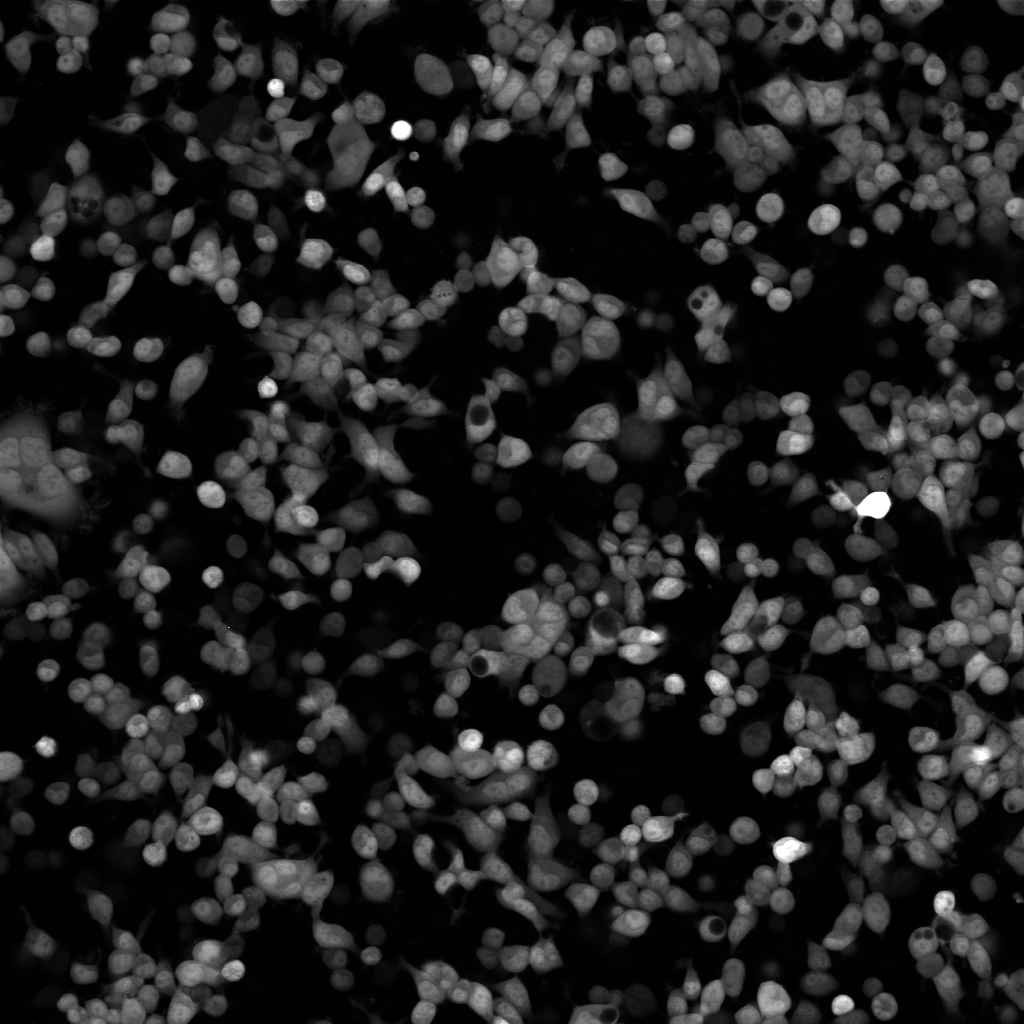

Supplement: Supplementary file 3 — Source Data [file 41467_2021_23889_MOESM3_ESM.zip › Raw microscopy images/Figure 6 and Supp Fig 18/Day 2_Equalizer-L episome.tif]

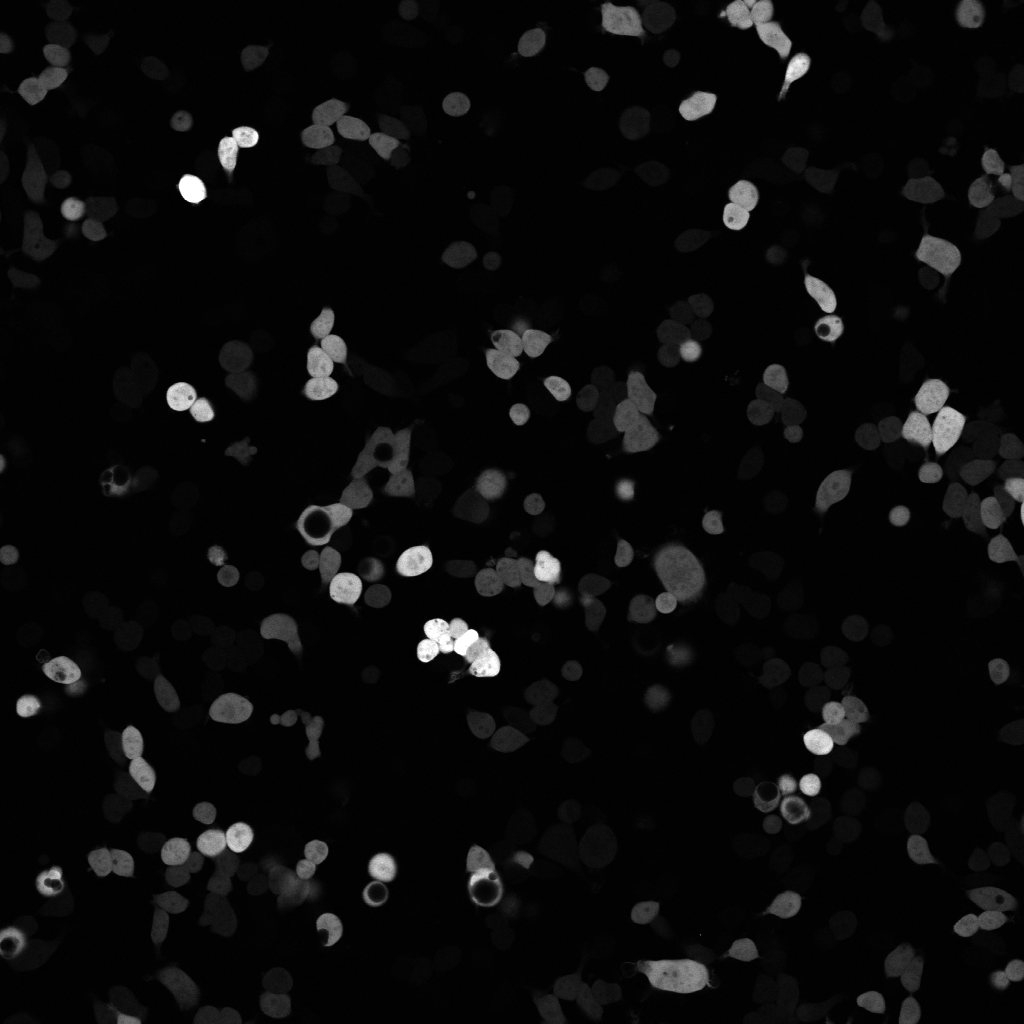

Supplement: Supplementary file 3 — Source Data [file 41467_2021_23889_MOESM3_ESM.zip › Raw microscopy images/Figure 6 and Supp Fig 18/Day 2_PGK episome.tif]

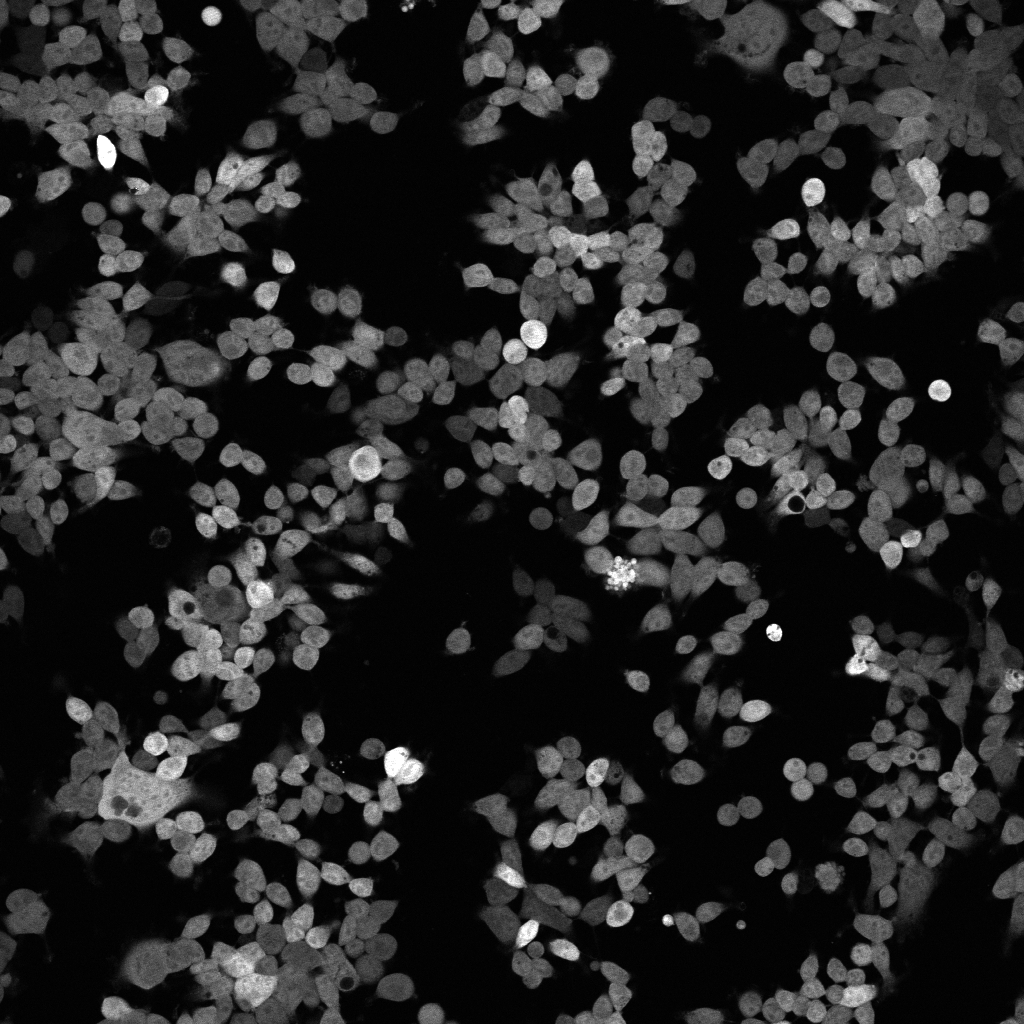

Supplement: Supplementary file 3 — Source Data [file 41467_2021_23889_MOESM3_ESM.zip › Raw microscopy images/Figure 6 and Supp Fig 18/Day 23_CMV cell line.tif]

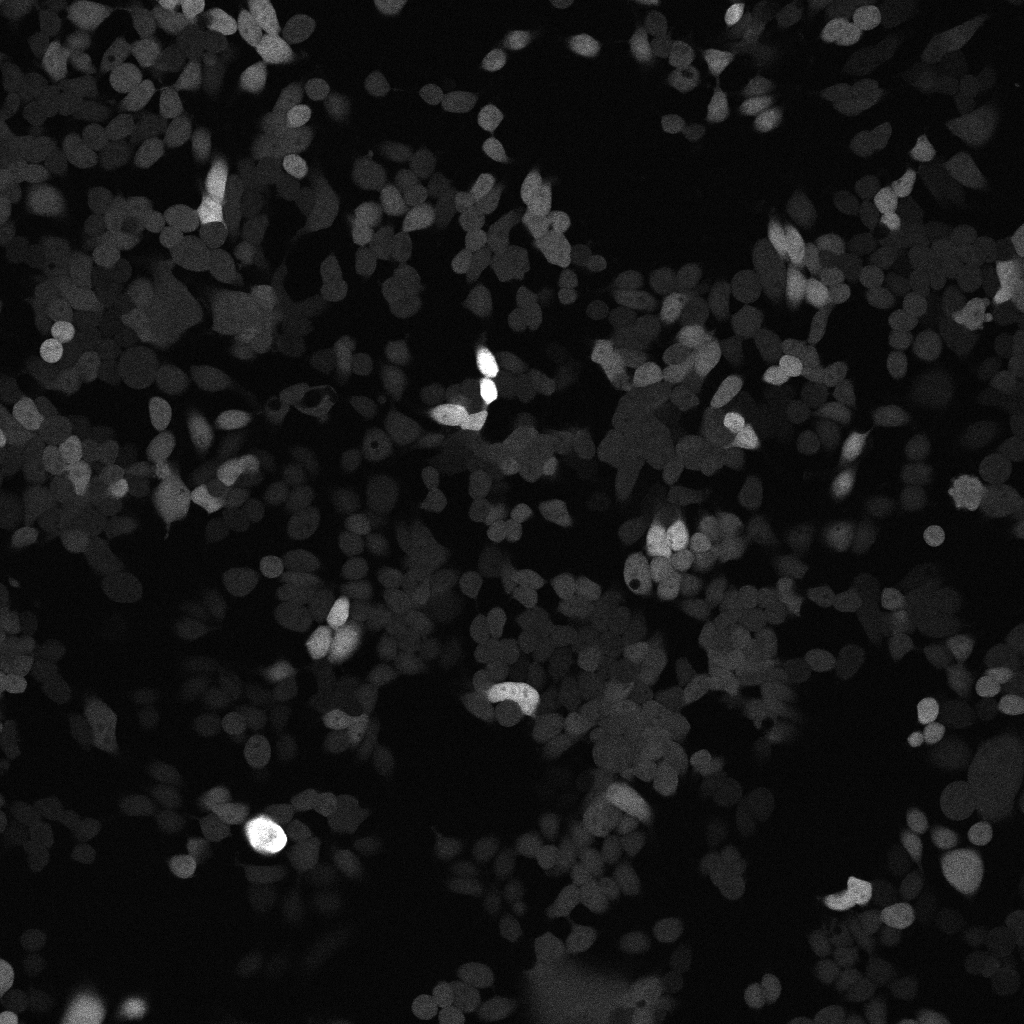

Supplement: Supplementary file 3 — Source Data [file 41467_2021_23889_MOESM3_ESM.zip › Raw microscopy images/Figure 6 and Supp Fig 18/Day 23_CMV episome.tif]

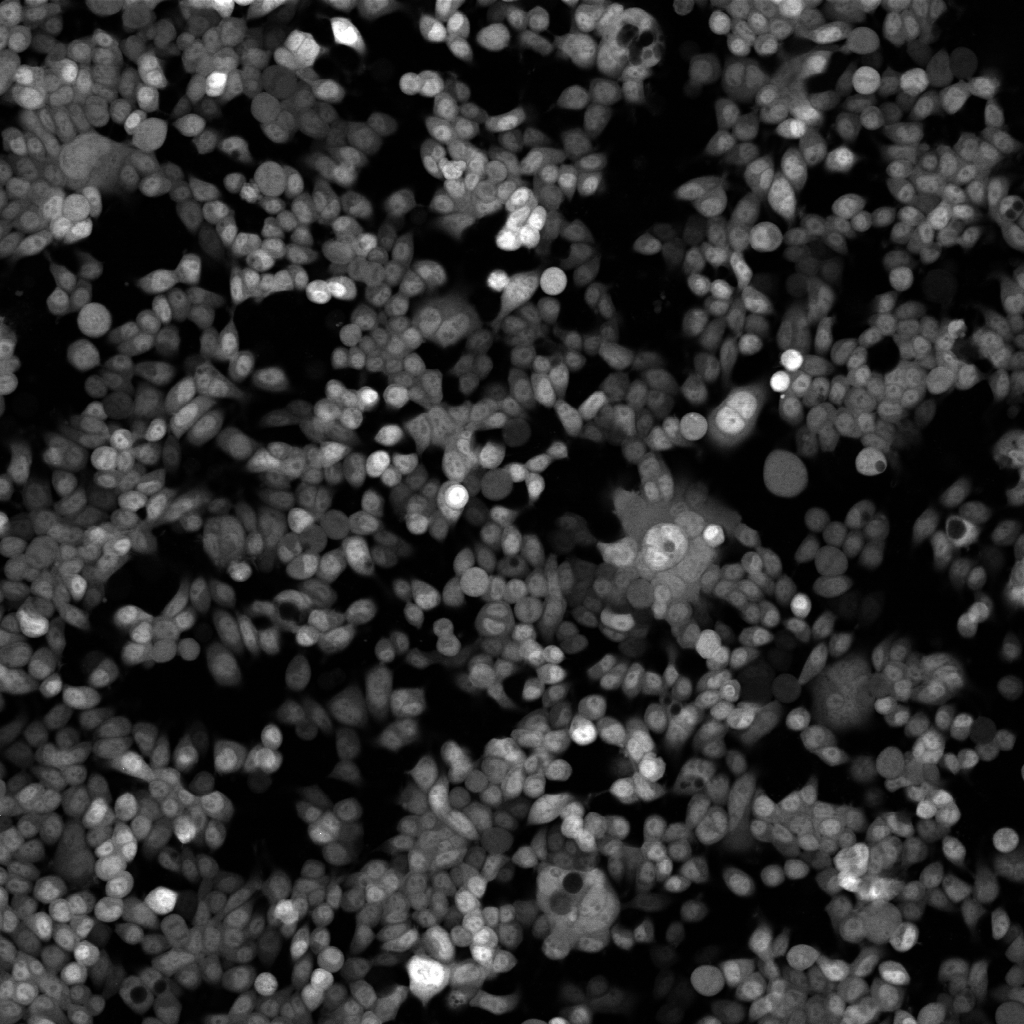

Supplement: Supplementary file 3 — Source Data [file 41467_2021_23889_MOESM3_ESM.zip › Raw microscopy images/Figure 6 and Supp Fig 18/Day 23_Equalizer-L episome.tif]

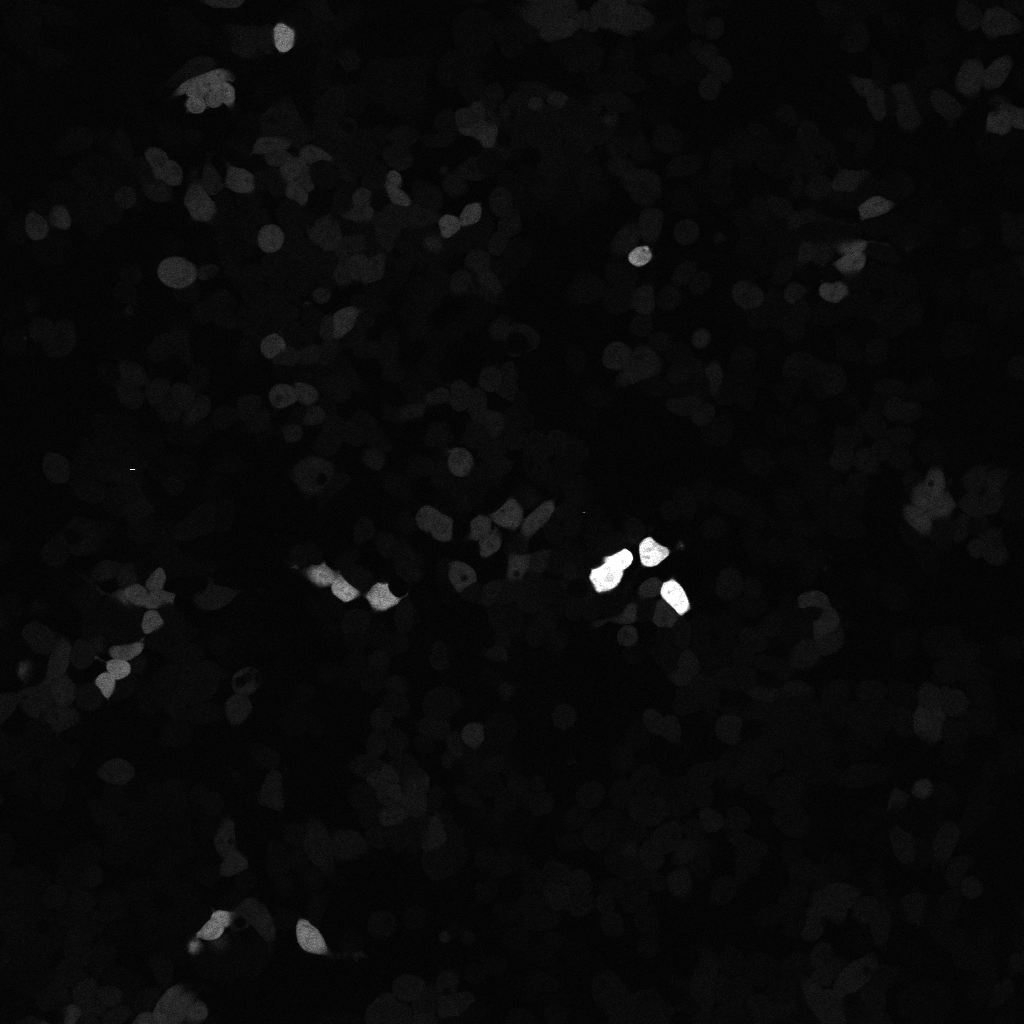

Supplement: Supplementary file 3 — Source Data [file 41467_2021_23889_MOESM3_ESM.zip › Raw microscopy images/Figure 6 and Supp Fig 18/Day 23_PGK episome.tif]

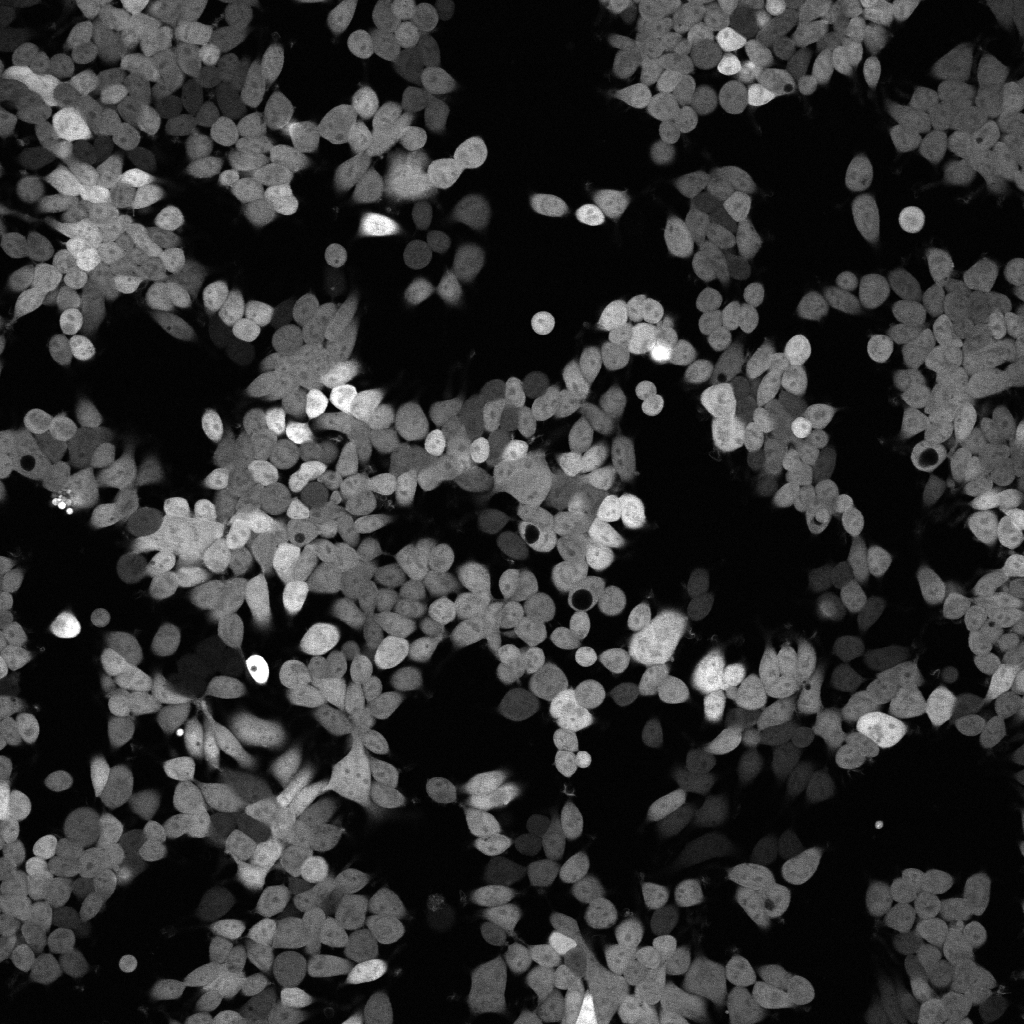

Supplement: Supplementary file 3 — Source Data [file 41467_2021_23889_MOESM3_ESM.zip › Raw microscopy images/Figure 6 and Supp Fig 18/Day 40_CMV cell line.tif]

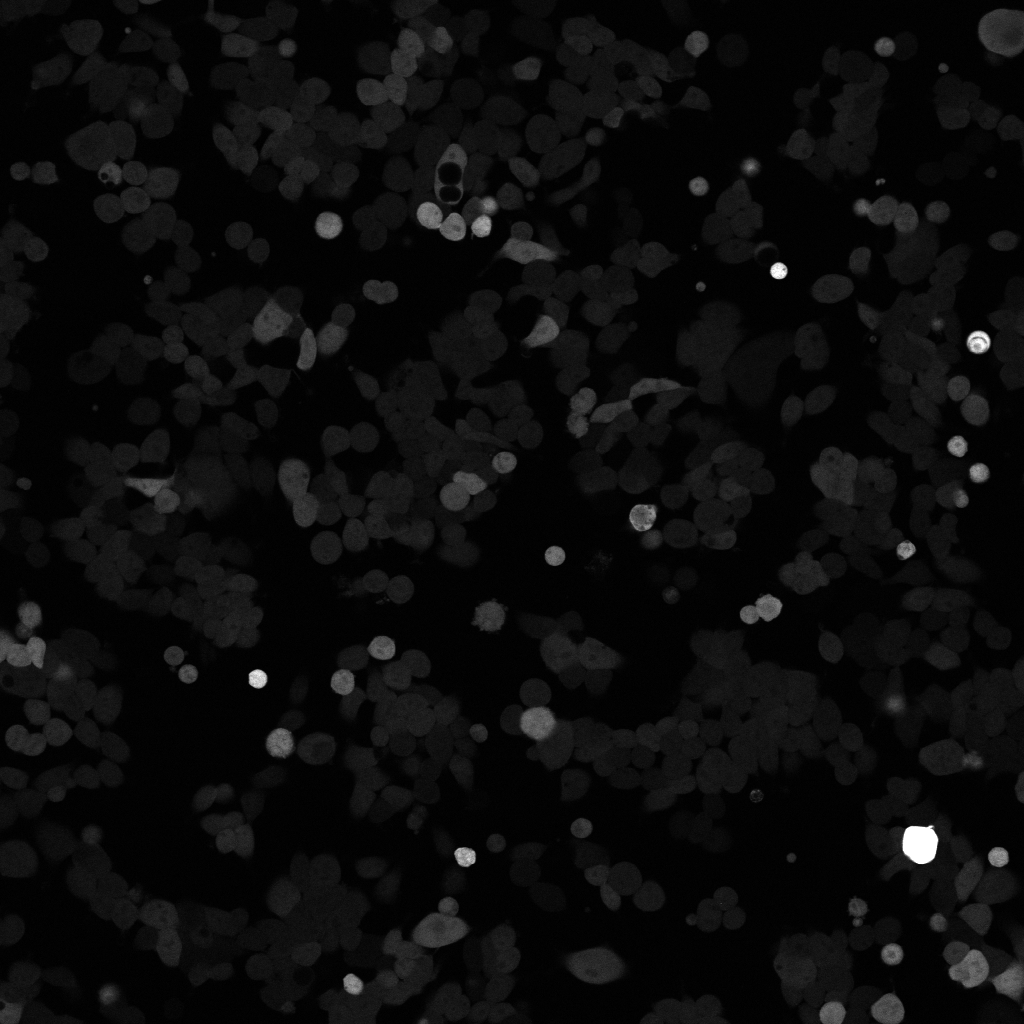

Supplement: Supplementary file 3 — Source Data [file 41467_2021_23889_MOESM3_ESM.zip › Raw microscopy images/Figure 6 and Supp Fig 18/Day 40_CMV episome.tif]

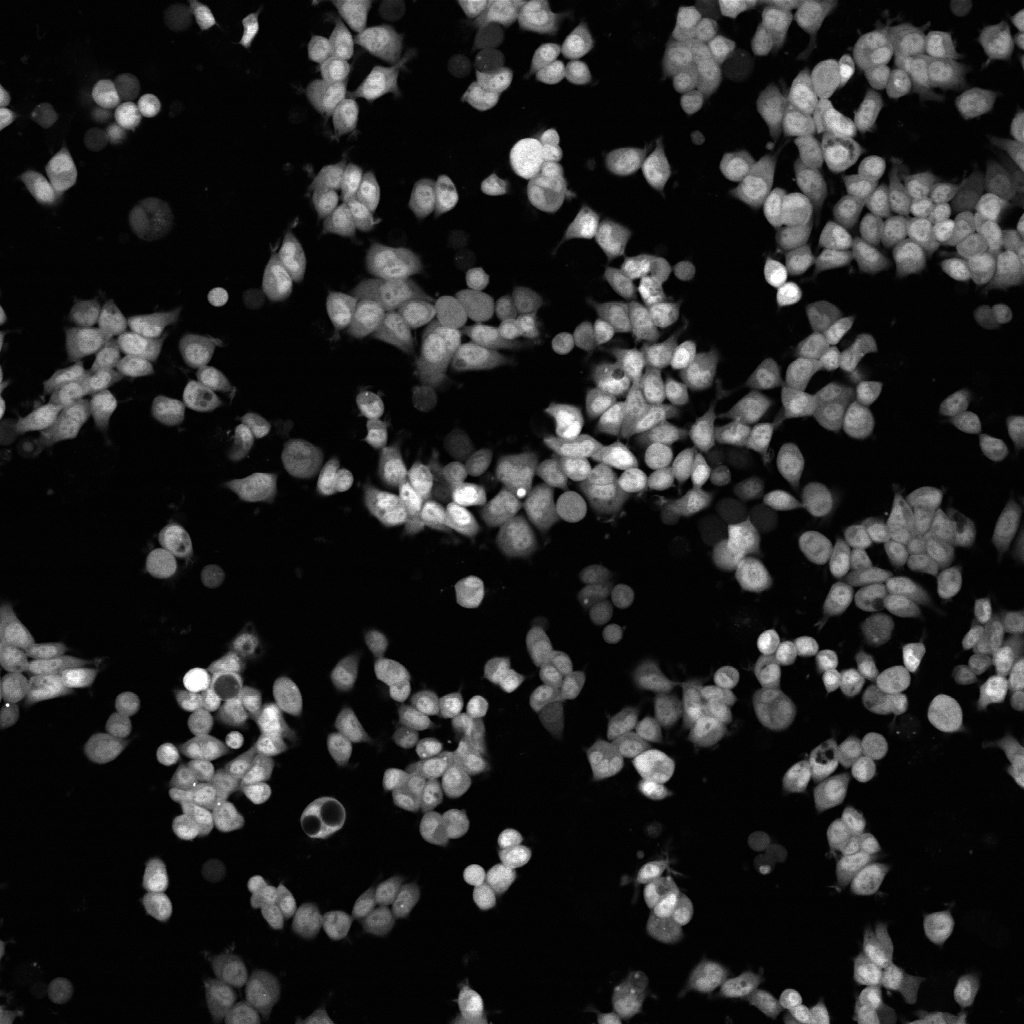

Supplement: Supplementary file 3 — Source Data [file 41467_2021_23889_MOESM3_ESM.zip › Raw microscopy images/Figure 6 and Supp Fig 18/Day 40_Equalizer-L episome.tif]

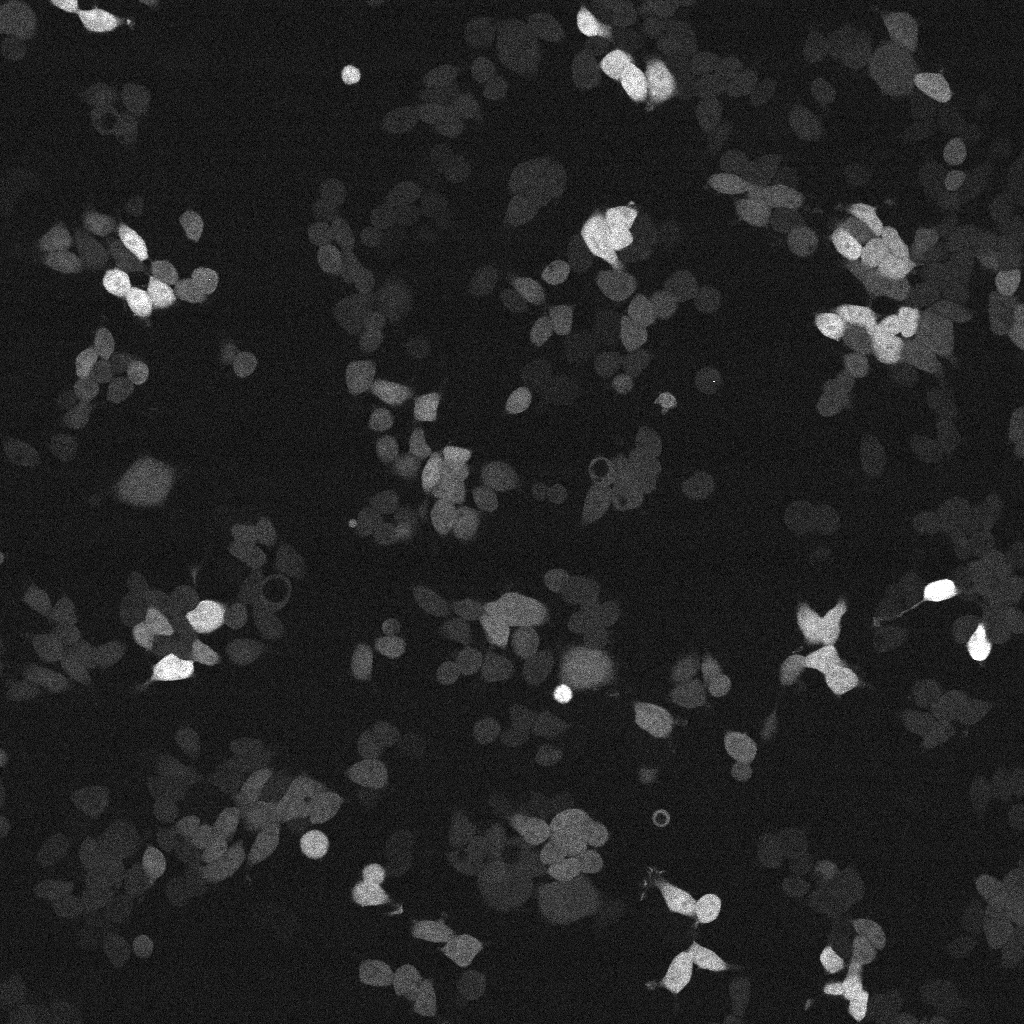

Supplement: Supplementary file 3 — Source Data [file 41467_2021_23889_MOESM3_ESM.zip › Raw microscopy images/Figure 6 and Supp Fig 18/Day 40_PGK episome.tif]

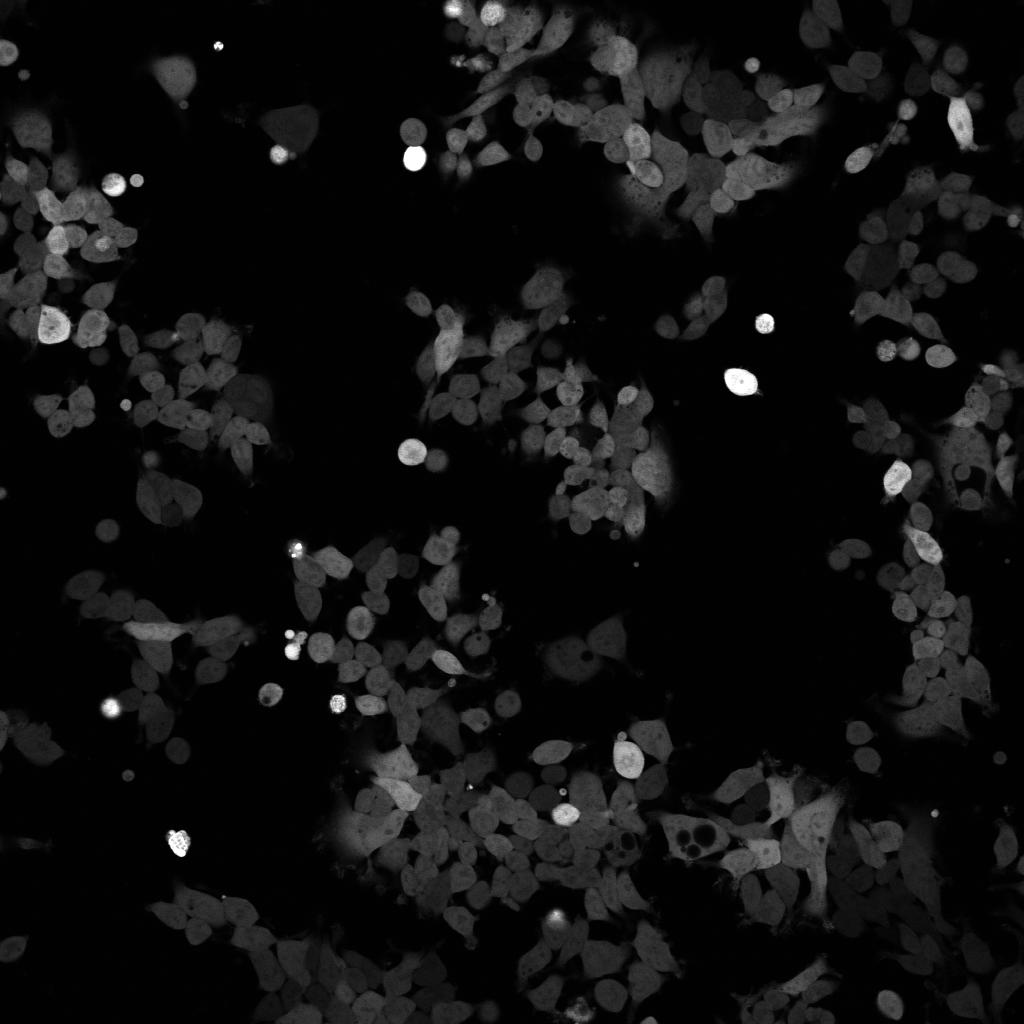

Supplement: Supplementary file 3 — Source Data [file 41467_2021_23889_MOESM3_ESM.zip › Raw microscopy images/Figure 6 and Supp Fig 18/Day 60_CMV cell line.tif]

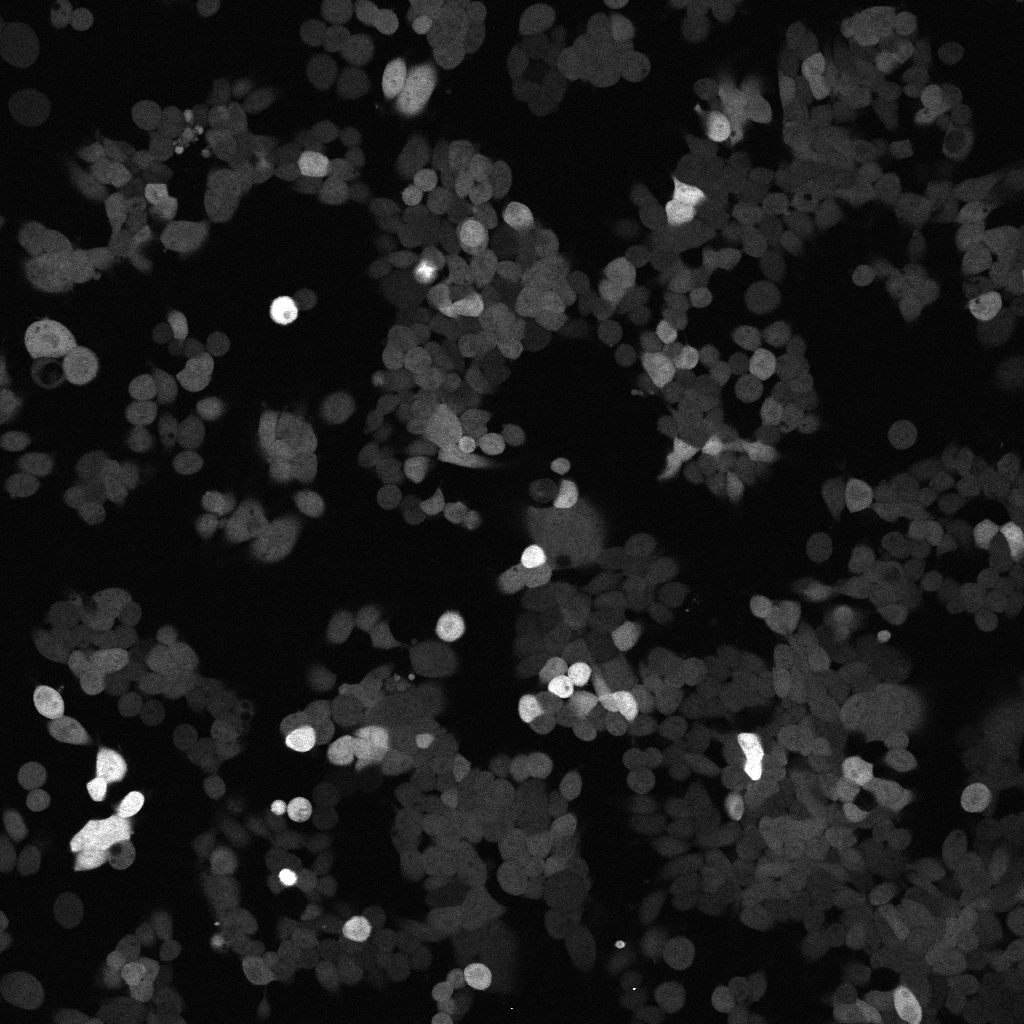

Supplement: Supplementary file 3 — Source Data [file 41467_2021_23889_MOESM3_ESM.zip › Raw microscopy images/Figure 6 and Supp Fig 18/Day 60_CMV episome.tif]

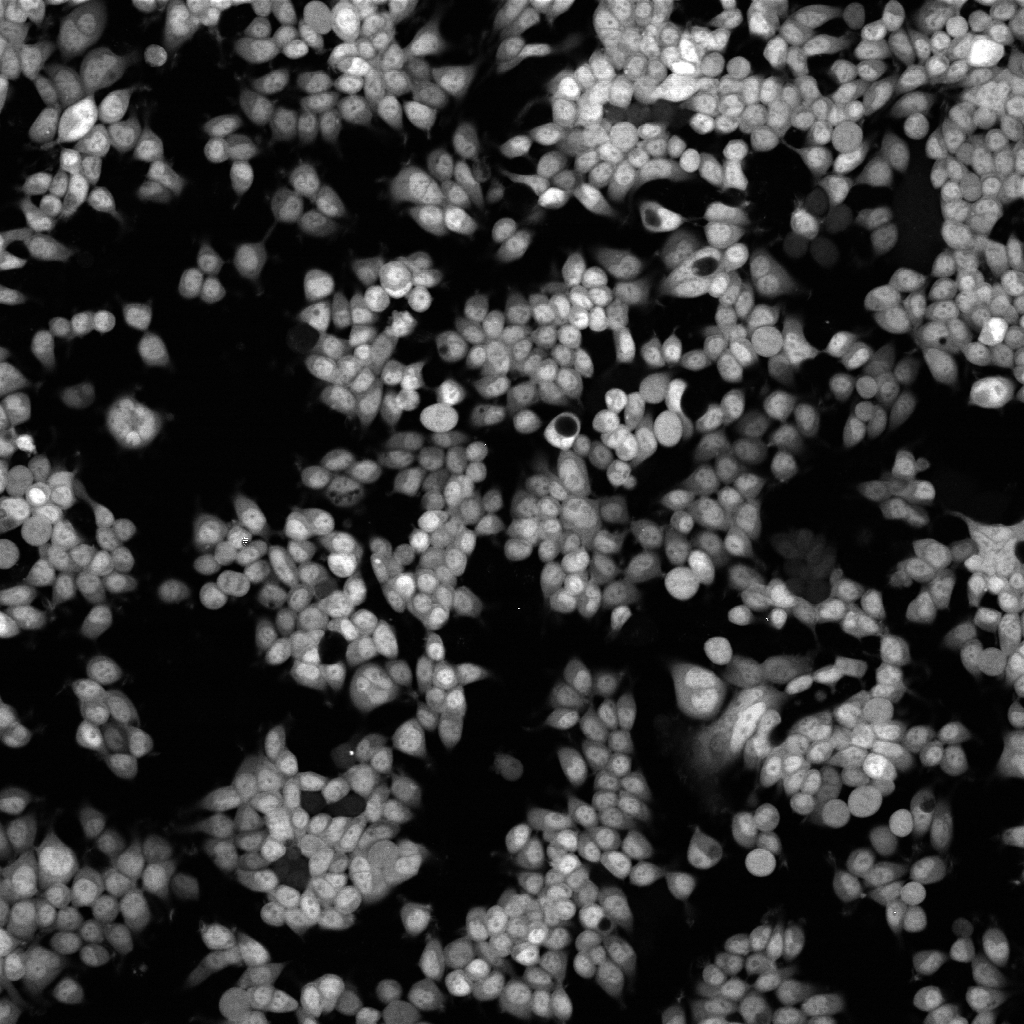

Supplement: Supplementary file 3 — Source Data [file 41467_2021_23889_MOESM3_ESM.zip › Raw microscopy images/Figure 6 and Supp Fig 18/Day 60_Equalizer-L episome.tif]

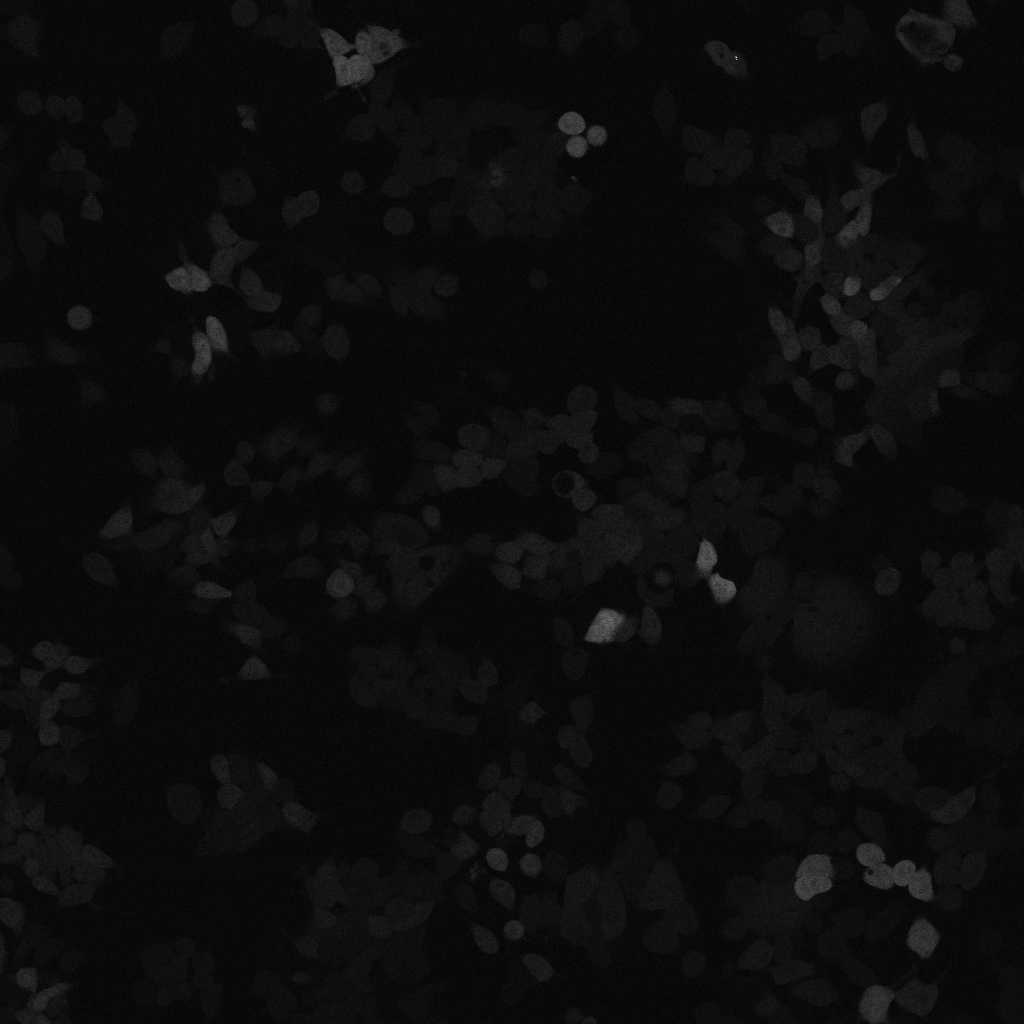

Supplement: Supplementary file 3 — Source Data [file 41467_2021_23889_MOESM3_ESM.zip › Raw microscopy images/Figure 6 and Supp Fig 18/Day 60_PGK episome.tif]

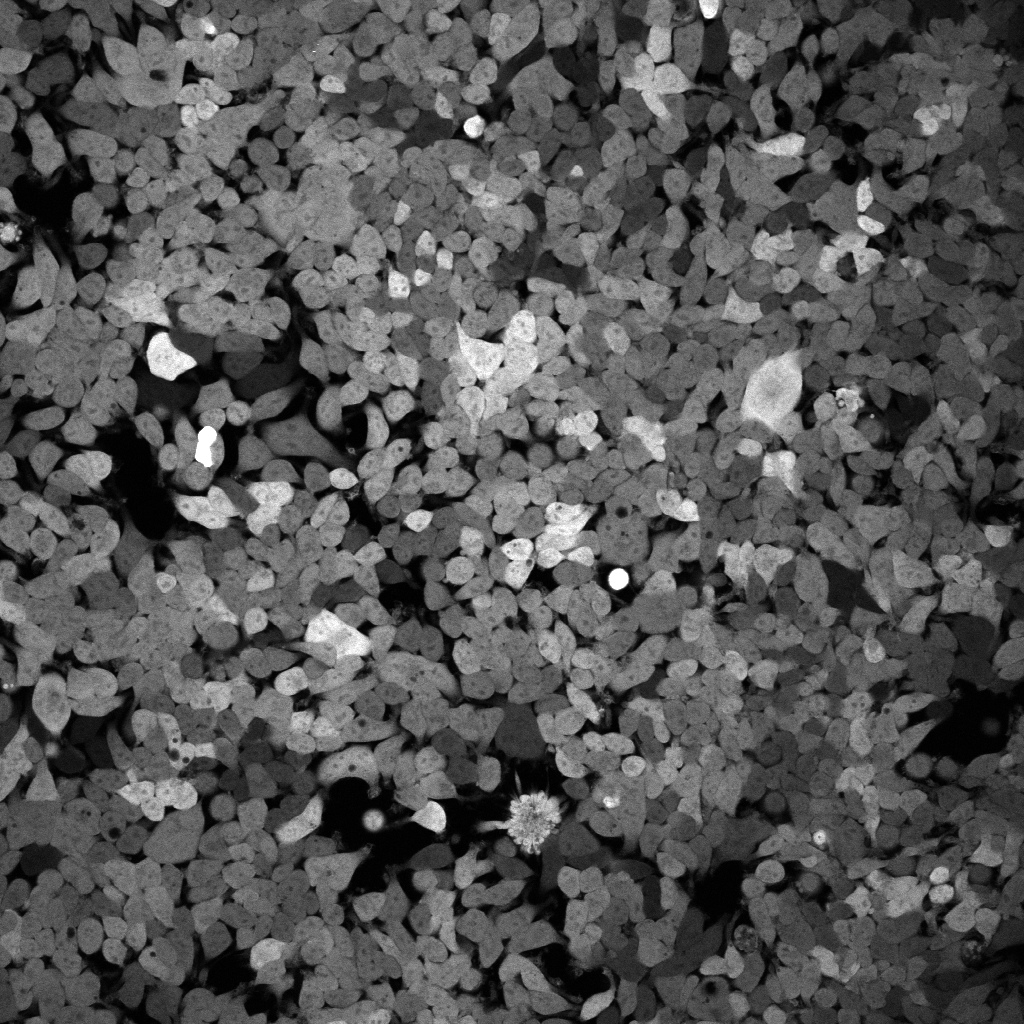

Supplement: Supplementary file 3 — Source Data [file 41467_2021_23889_MOESM3_ESM.zip › Raw microscopy images/Figure 6 and Supp Fig 18/Day 9_CMV cell line.tif]

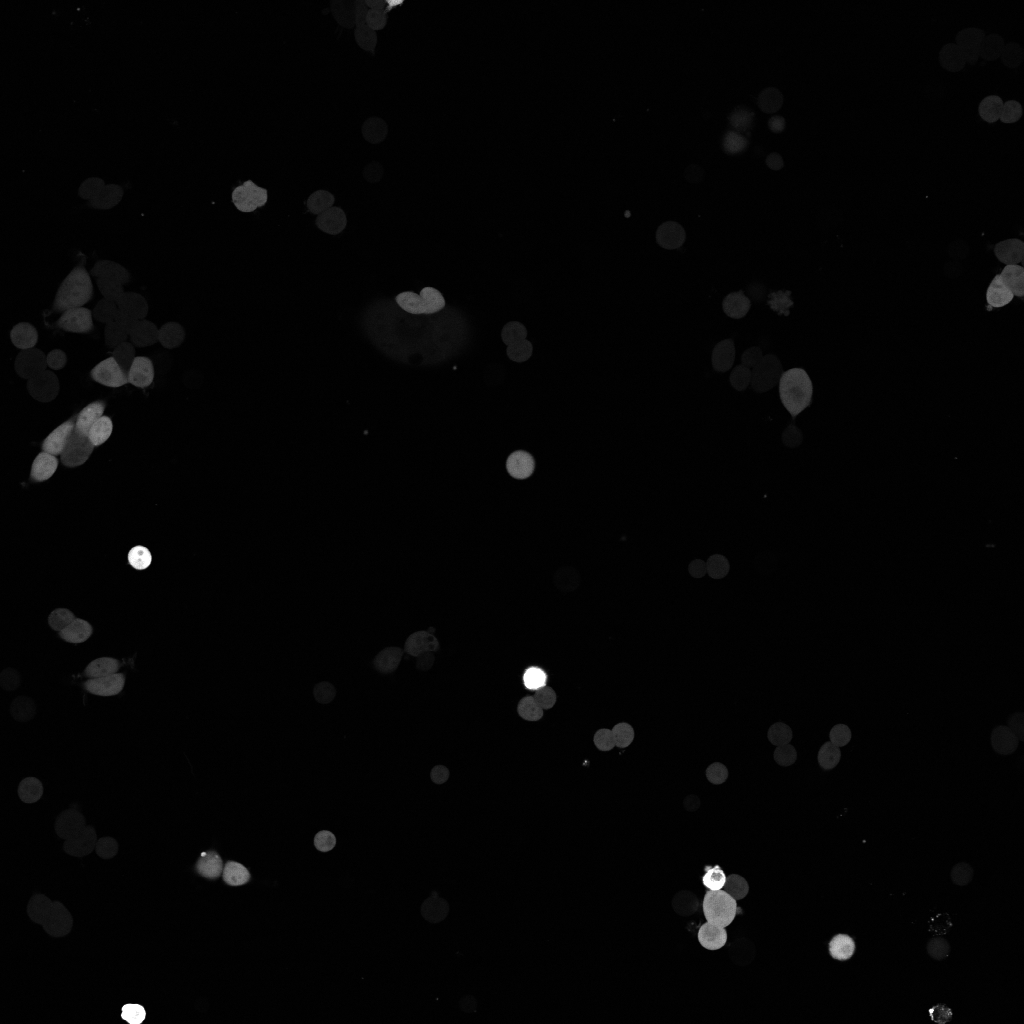

Supplement: Supplementary file 3 — Source Data [file 41467_2021_23889_MOESM3_ESM.zip › Raw microscopy images/Figure 6 and Supp Fig 18/Day 9_CMV episome.tif]

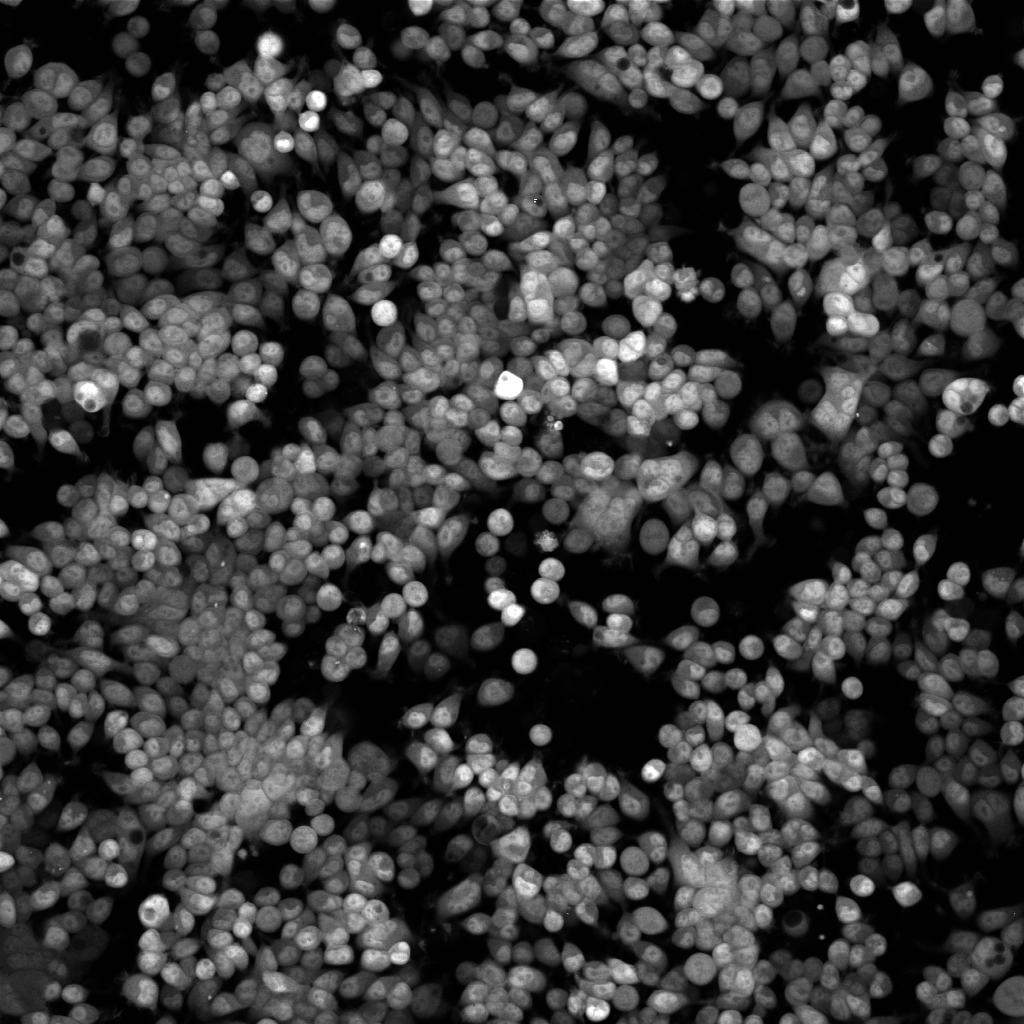

Supplement: Supplementary file 3 — Source Data [file 41467_2021_23889_MOESM3_ESM.zip › Raw microscopy images/Figure 6 and Supp Fig 18/Day 9_Equalizer-L episome.tif]

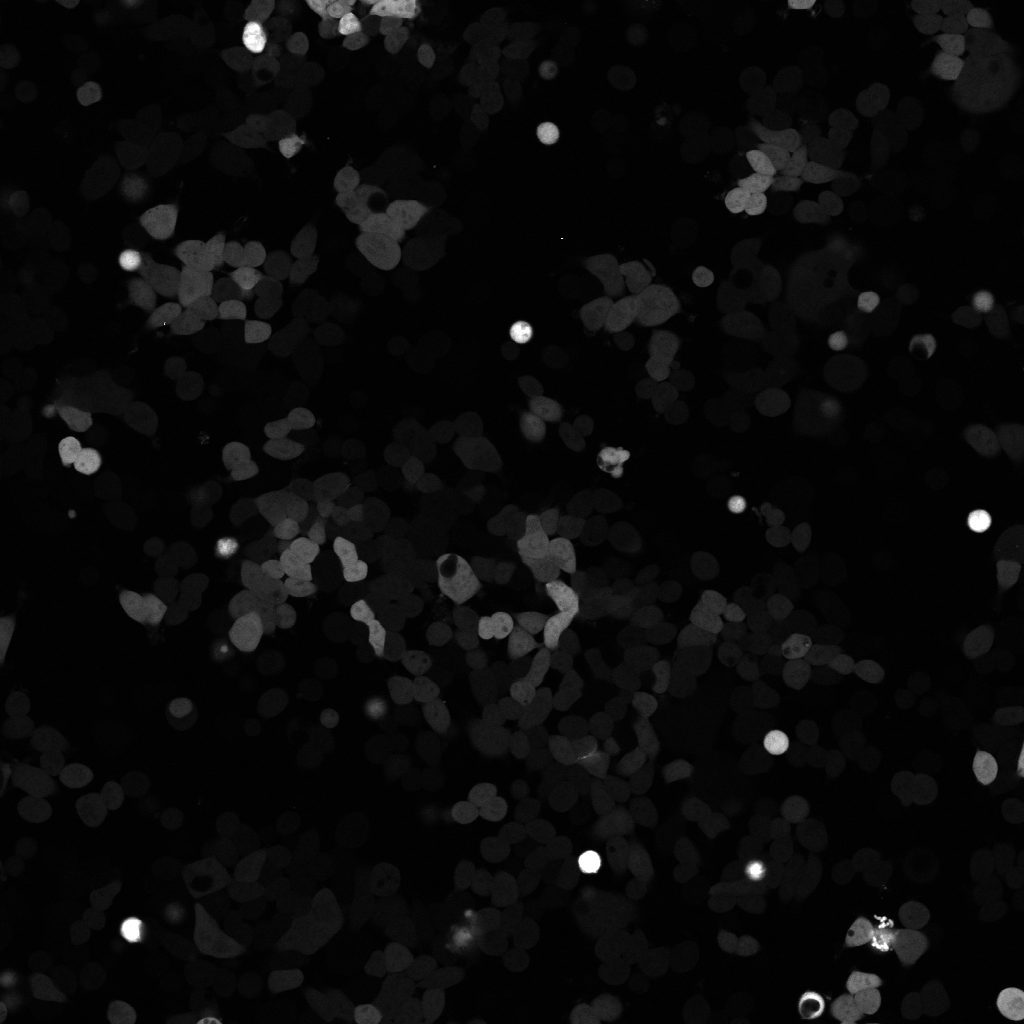

Supplement: Supplementary file 3 — Source Data [file 41467_2021_23889_MOESM3_ESM.zip › Raw microscopy images/Figure 6 and Supp Fig 18/Day 9_PGK episome.tif]

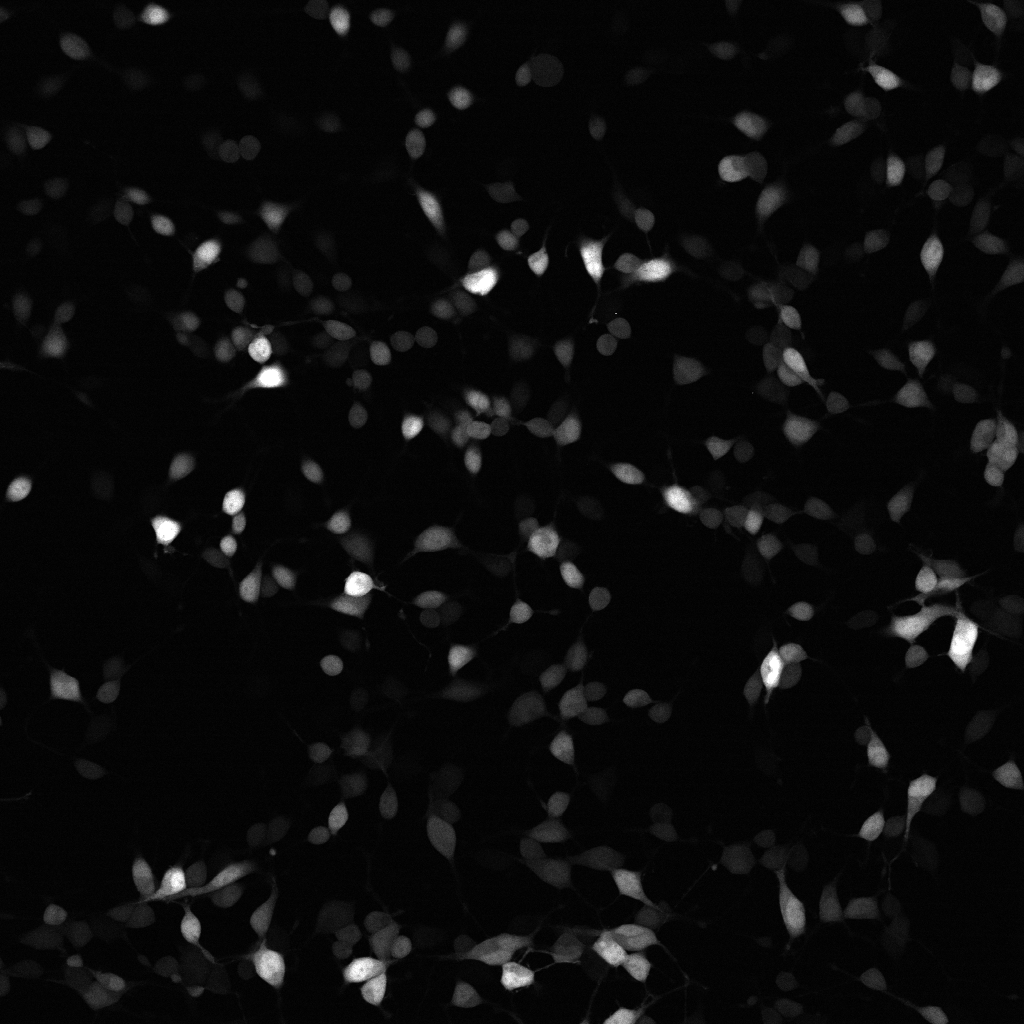

Supplement: Supplementary file 3 — Source Data [file 41467_2021_23889_MOESM3_ESM.zip › Raw microscopy images/Supp Fig 3/No induction.tif]

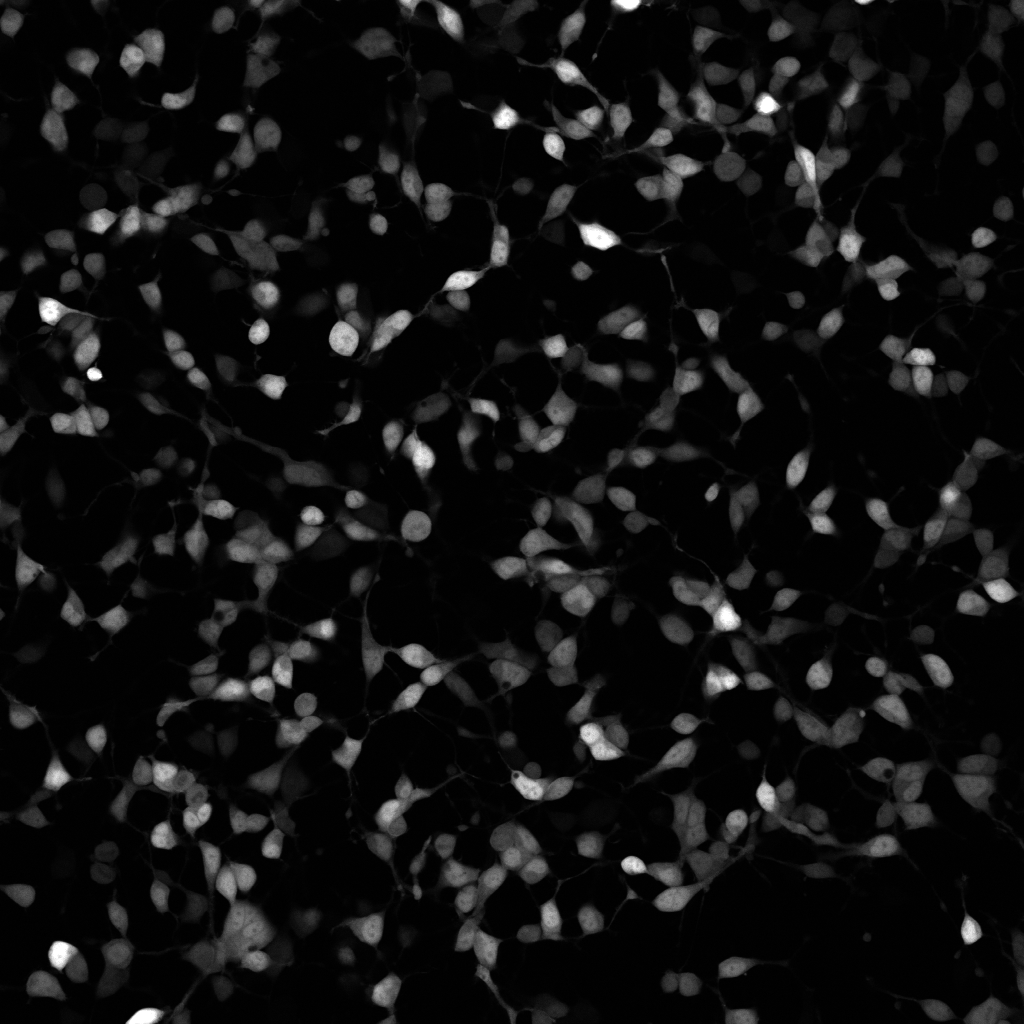

Supplement: Supplementary file 3 — Source Data [file 41467_2021_23889_MOESM3_ESM.zip › Raw microscopy images/Supp Fig 3/Saturated induction.tif]
